# Supplementary material for: Bazedoxifene reverses sexually dimorphic autistic-like abnormalities in biallelic MDGA1-mutant mice
Source: EMBO Mol Med. 2026 Mar 20;18(4):1358–98. doi: 10.1038/s44321-026-00402-y (PMC13084050; doi:10.1038/s44321-026-00402-y)
Supplement: Supplementary file 1 — Table EV1 [file 44321_2026_402_MOESM1_ESM.doc]

**Table EV1. Description of patients with the ASD-associated *MDGA1* variants described in the current study and the functional effects of these variants predicted using four different bioinformatics tools**

| **Variant** | **First Family** | | | **Second Family** | |
| --- | --- | --- | --- | --- | --- |
| Position | | g.37612408C>G | g.37615091T>C | g.37626057C>T | g.37614135G>A |
| DNA change | | c.2266 G>C | c.1904 A>G | c.346 G>A | c.2063 C>T |
| Amino acid change | | p.Glu756Gln | p.Tyr635Cys | p.Val116Met | p.Ala688Val |
| Exon | | 13 | 15 | 3 | 11 |
| ACMG Classification | | Uncertain Significance | Uncertain Significance | Uncertain Significance | Likely Benign |
| Gnomad Frequency | | ≈0.07% | ≈0.001% | < 0.001% | ≈0.07% |
| Healthy Homozygotes | | No | No | No | No |
| CADD score | | 32 | 27.2 | 23.6 | 21.3 |

Abbreviations: N.D., not determined
