# Supplementary material for: Bazedoxifene reverses sexually dimorphic autistic-like abnormalities in biallelic MDGA1-mutant mice
Source: EMBO Mol Med. 2026 Mar 20;18(4):1358–98. doi: 10.1038/s44321-026-00402-y (PMC13084050; doi:10.1038/s44321-026-00402-y)
Supplement: Supplementary file 2 — Table EV2 [file 44321_2026_402_MOESM2_ESM.doc]

**Table EV2. Stability analysis of ASD-associated *MDGA1* mutations**

The free energy of folding (stability) was calculated using FoldX5.

| **Model** | **Energy (kcal.mol-1)** |
| --- | --- |
| MDGA1 (Q19-P742) WT | 0.04 |
| MDGA1 (Q19-P742) V116M | 0.04 |
| MDGA1 (Q19-P742) A688V | 1.89 |
| MDGA1 (Q19-P742) Y635C | 7.02 |

Abbreviations: WT, wild type
