# Supplementary material for: Bazedoxifene reverses sexually dimorphic autistic-like abnormalities in biallelic MDGA1-mutant mice
Source: EMBO Mol Med. 2026 Mar 20;18(4):1358–98. doi: 10.1038/s44321-026-00402-y (PMC13084050; doi:10.1038/s44321-026-00402-y)
Supplement: Supplementary file 3 — Table EV3 [file 44321_2026_402_MOESM3_ESM.doc]

**Table EV3. Analyses of potential association between *MDGA1* variants and neurodevelopmental/neuropsychiatric diseases in major gene/disease databases**

| **Database** | **Disease**  **/Phenotype** | **Variant Type** | **Association/Evidence** | **Note / Link** |
| --- | --- | --- | --- | --- |
| SFARI GENE | Autism spectrum disorder (ASD) | - | Not listed | <https://gene.sfari.org/database/human-gene/MDGA1> |
| OMIM | Neurodevelopmental/neuropsychiatric | - | No direct disease association | <https://www.omim.org/entry/613126> |
| ClinGen | ASD, neurodevelopmental disorders | - | No dosage pathogenicity  /association | <https://search.clinicalgenome.org/kb/genes/hgnc/MDGA1> |
| gnomAD v4 | - | pLI = 0 (No LoF constraint) | Multiple PTVs observed | [gnomAD v4](https://gnomad.broadinstitute.org/gene/ENSG00000112139?dataset=gnomad_r4) |
| DECIPHER | Neurodevelopmental disorders | CNV (triplication, VUS) | Patient #360922: MDGA1 duplication (VUS) | [DECIPHER](https://www.deciphergenomics.org/gene/MDGA1/patient-overlap/cnvs) |
