# Supplementary material for: Bazedoxifene reverses sexually dimorphic autistic-like abnormalities in biallelic MDGA1-mutant mice
Source: EMBO Mol Med. 2026 Mar 20;18(4):1358–98. doi: 10.1038/s44321-026-00402-y (PMC13084050; doi:10.1038/s44321-026-00402-y)
Supplement: Supplementary file 4 — Appendix [file 44321_2026_402_MOESM4_ESM.pdf]

Bazedoxifene reverses sexually dimorphic autistic-like abnormalities in biallelic MDGA1-mutant mice

Seungjoon Kim[1,2,*], Hyeonho Kim[1,2,*], Javier Porta Pelayo[3,*], Sara Alvarez[4,*], Gyubin Jang[1,2], Jinhu Kim[1,2], Byeongchan Kim[1], Victoria M. Hoelscher[5], Beatriz Calleja-Pérez[6], Hyunsu Jung[7], Yeji Yang[8,9], Hea Ji Lee[8], Jihae Lee[10], Seoyeon Kim[11,12,13], Mar Jiménez de la Peña[14,15], Yelin Lee[1], Sohye Kim[1], Ah-reum Han[16], Dong Sun Lee[16], Sangho Ji[1], Wookyung Yu[1,2], Ho Min Kim[9,16], Joon-Yong An[10,11,12,13], Won Chan Oh[5], Seok-Kyu Kwon[7,17], Jin Young Kim[8,18], Ji Won Um[1,2], Alberto Fernández-Jaén[18,19,#] & Jaewon Ko[1,2,#]

[1]Department of Brain Sciences, Daegu Gyeongbuk Institute of Science and Technology (DGIST), Daegu 42988, Korea
[2]Center for Synapse Diversity and Specificity, DGIST, Daegu 42988, Korea
[3]Genomics, Genologica, Málaga, Spain
[4]Genomics, NimGenetics, Madrid, Spain
[5]Department of Pharmacology, University of Colorado School of Medicine, Anschutz Medical Campus, Aurora, CO 80045, USA
[6]Pediatric Primary Care, C.S. Doctor Cirajas, Madrid, Spain
[7]Brain Science Institute, Korea Institute of Science and Technology (KIST), Seoul 02792, Korea
[8]Digital Omics Research Center, Korea Basic Science Institute (KBSI), Ochang 28119, Korea
[9]Department of Biological Sciences, Korea Advanced Institute of Science and Technology (KAIST), Daejeon 34141, Korea
[10]School of Biosystem and Biomedical Science, College of Health Science, Korea University, Seoul 02841, Korea
[11]Department of Integrated Biomedical and Life Science, Korea University, Seoul 02841, Korea
[12]BK21 Four R&E Center for Learning Health Systems, Korea University, Seoul 02841, Korea
[13]L-HOPE Program for Community-Based Total Learning Health Systems, Korea University, Seoul 02841, Korea
[14]Neuroimaging, Hospital Universitario Quirónsalud, Madrid, Spain
[15]Universidad Europea de Madrid, Spain
[16]Center for Biomolecular & Cellular Structure, Institute for Basic Science (IBS), Daejeon 34126, Korea
[17]Dividion of Bio-Medical Science & Technology, KIST School, Korea University of Science and Technology (UST), Daejeon 34113, Korea
[18]College of Pharmacy, Chung-Ang University, Seoul 06974, Korea
[19]Department of Pediatric Neurology, Hospital Universitario Quirónsalud, Madrid, Spain

*These authors contributed equally
[#]Corresponding author: Alberto Fernández-Jaén: aferjaen@telefonica.net; Jaewon Ko: jaewonko@dgist.ac.kr

APPENDIX TABLES & FIGURES

Appendix Table of Contents

Appendix Figure S1. Diffusion tensor 2D-map and 3D-tractography reconstruction--------Pg4
Appendix Figure S2. Analysis of MDGA1 expression in developing human neocortex-----Pg5
Appendix Figure S3. MDGA1 expression in individuals with ASD versus control subjects---Pg6
Appendix Figure S4. Sequence comparison and analysis of MDGA1 across different species---Pg7
Appendix Figure S5. Negative-stain electron microscopy of the full ecto-domain of hMDGA1 WT and hMDGA1 Y635C/E756Q protein---Pg8
Appendix Figure S6. Analysis of the synapse-suppressing activities of the ASD-associated MDGA1 variants--Pg9
Appendix Figure S7. Effects of overexpression of the ASD-associated MDGA1 variants on GABAergic synapses in cultured hippocampal neurons--Pg10
Appendix Figure S8. Effects of overexpression of MDGA2 variants (equivalent MDGA1 residues associated with ASDs were introduced) on glutamatergic synapses in cultured hippocampal neurons---Pg11
Appendix Figure S9. Effects of overexpression of the ASD-associated MDGA1 variants on GABAergic synapses in adult mPFC layer II/III pyramidal neurons---------------------------Pg12
Appendix Figure S10. Effects of overexpression of the ASD-associated MDGA1 variants on asynchronous evoked GABAergic transmission in adult hippocampal CA1 pyramidal neurons---Pg14
Appendix Figure S11. Effects of overexpression of the ASD-associated MDGA1 variants on glutamatergic synapses in cultured hippocampal neurons---------------------------------------Pg15
Appendix Figure S12. Analysis of behaviors of adult male *Mdga1*-cKO mice----------------Pg16
Appendix Figure S13. Analysis of body weight, brain weight and the survival curve for *Mdga1*-cKO and *Mdga1*$^{Y636C/E751Q}$ KI mice--Pg17
Appendix Figure S14. Analysis of behaviors of adult male *Mdga1*$^{Y636C/E751Q}$ KI mice---------Pg18
Appendix Figure S15. Analysis of expression levels of *Mdga* mRNAs and MDGA proteins as well as expression levels of other synaptic proteins in the hippocampus of male *Mdga1*-cKO and male *Mdga1*$^{Y636C/E751Q}$ KI mice--Pg19
Appendix Figure S16. Analysis of glutamatergic and GABAergic synaptic puncta in the hippocampus of adult male *Mdga1*-cKO mice---Pg20
Appendix Figure S17. Analysis of glutamatergic and GABAergic synaptic puncta in the hippocampus of adult female *Mdga1*-cKO mice---Pg21
Appendix Figure S18. Analysis of glutamatergic and GABAergic synaptic puncta in the mPFC of adult male *Mdga1*-cKO mice---Pg22
Appendix Figure S19. Analysis of glutamatergic and GABAergic synaptic puncta in the mPFC of adult female *Mdga1*-cKO mice---Pg23
Appendix Figure S20. Analysis of glutamatergic and GABAergic synaptic puncta in the hippocampus of adult male *Mdga1*$^{Y636C/E751Q}$ KI mice---Pg24

Appendix Figure S21. Analysis of glutamatergic and GABAergic synaptic puncta in the hippocampus of adult female *Mdga1*$^{Y636C/E751Q}$ KI mice--Pg25
Appendix Figure S22. Analysis of glutamatergic and GABAergic synaptic puncta in the mPFC of adult male *Mdga1*$^{Y636C/E751Q}$ KI mice---Pg26
Appendix Figure S23. Analysis of glutamatergic and GABAergic synaptic puncta in the mPFC of adult female *Mdga1*$^{Y636C/E751Q}$ KI mice---Pg27
Appendix Figure S24. Analysis of asynchronous GABAergic evoked synaptic transmission in the hippocampal CA1 pyramidal neurons of adult male *Mdga1*-cKO and *Mdga1*$^{Y636C/E751Q}$ KI mice--Pg28
Appendix Figure S25. Analysis of density of interneurons and c-FOS+ neurons in the hippocampus of adult *Mdga1*-cKO and *Mdga1*$^{Y636C/E751Q}$ KI mice---------------------------------Pg29
Appendix Figure S26. Analysis of density of neurons, astrocytes and microglia across brain areas of adult *Mdga1*$^{Y636C/E751Q}$ KI mice--Pg30
Appendix Figure S27. Analysis of cortical neuron migration for adult male *Mdga1*-cKO mice---Pg31
Appendix Figure S28. Analysis of cortical neuron migration for adult male *Mdga1*$^{Y636C/E751Q}$ KI mice--Pg32
Appendix Figure S29. Volcano plot of proteins identified from proteomic analyses of hippocampi from adult *Mdga1*-cKO and *Mdga1*$^{Y636C/E751Q}$ KI mice--------------------------------Pg33
Appendix Figure S30. Analysis of phosphorylation sites that are altered in adult male *Mdga1*$^{Y636C/E751Q}$ KI mice---Pg34
Appendix Figure S31. Phosphoproteomic analysis of hippocampi from embryos of *Mdga1*-cKO and *Mdga1*$^{Y636C/E751Q}$ KI mice--Pg35
Appendix Figure S32. Estrogen receptor-associated protein abundance in hippocampi from embryonic and adult male *Mdga1*-cKO and *Mdga1*$^{Y636C/E751Q}$ KI mice---------------------------Pg36
Appendix Figure S33. Measurement of estradiol e2 levels from juvenile and adult male *Mdga1*$^{Y636C/E751Q}$ KI mice---Pg37
Appendix Figure S34. Effect of Fulvestrant administration into adult male *Mdga1*$^{Y636C/E751Q}$ KI mice on evoked GABAergic synaptic strength and GABA release probability---------------Pg38
Appendix Figure S35. Effect of conformational changes in MDGA1 extracellular regions on GABAergic synapses in cultured hippocampal neurons--Pg39

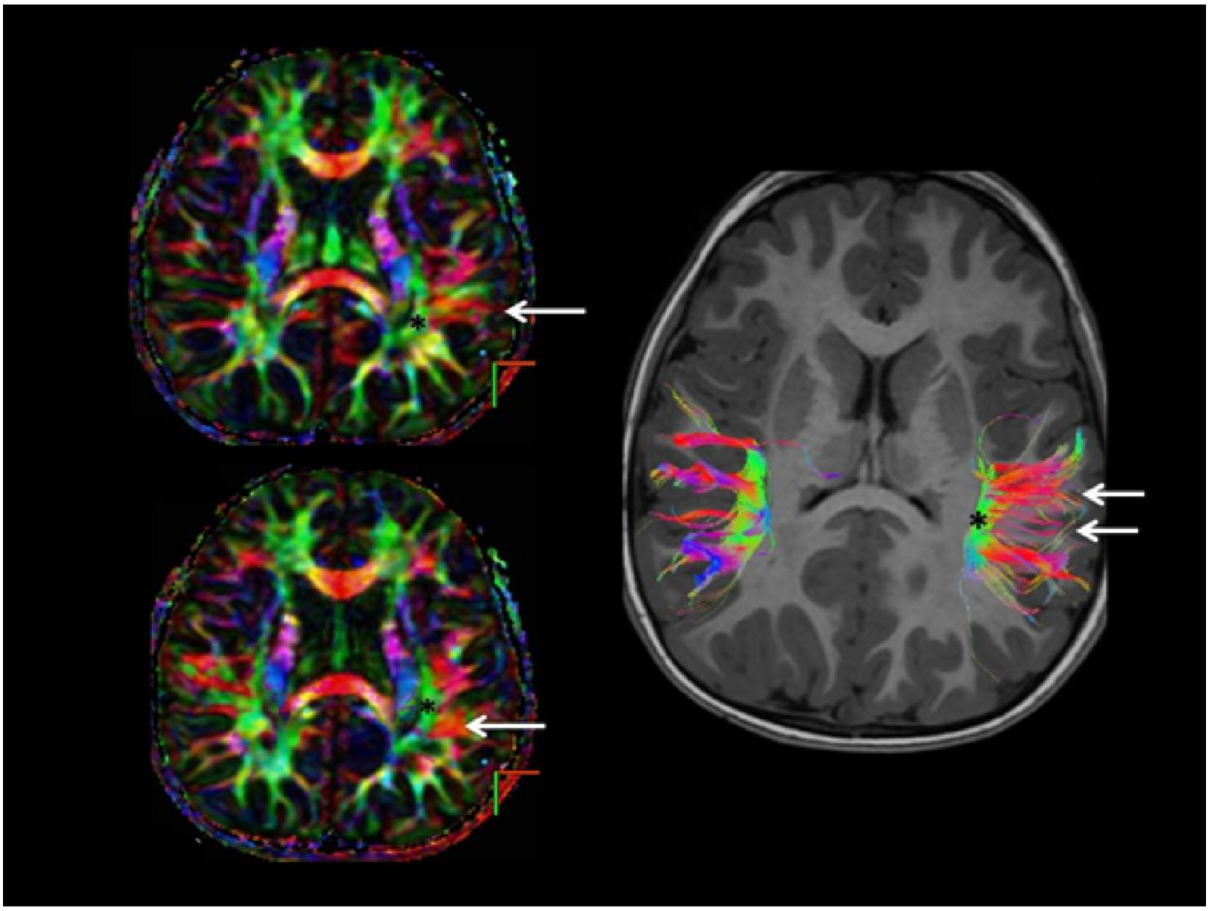

Appendix Figure S1. Diffusion tensor 2D-map and 3D-tractography reconstruction. Although a mild asymmetry of the anteroposterior component of the arcuate fascicle (asterisk) is visible on the 2D-fiber tracking image, the main finding is the clustering of the horizontal subcortical connections (white arrows) with the left inferior parietal lobe.

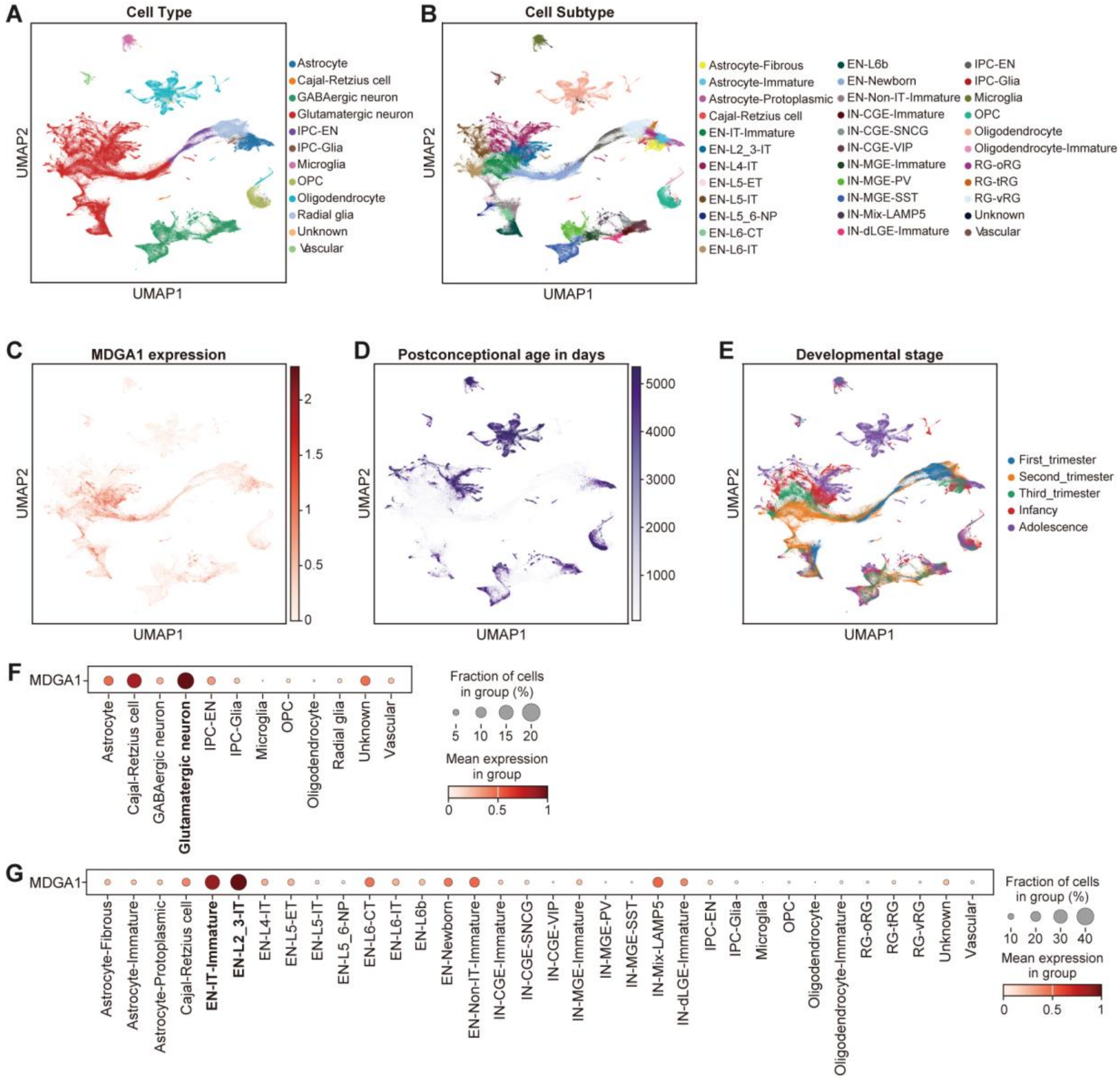

Appendix Figure S2. Analysis of MDGA1 expression in developing human neocortex. (**A–E**) UMAP visualization of the single-nucleus RNA-sequencing dataset from the developing human neocortex multiome atlas, showing major cell populations (**A**), granular neural cell subtypes (**B**), *MDGA1* expression (**C**), postconceptional age of cells in days (**D**), and developmental stages (**E**). (**F** and **G**) Dot plots quantifying MDGA1 expression across major cell types (**F**) and detailed neural subtypes (**G**).

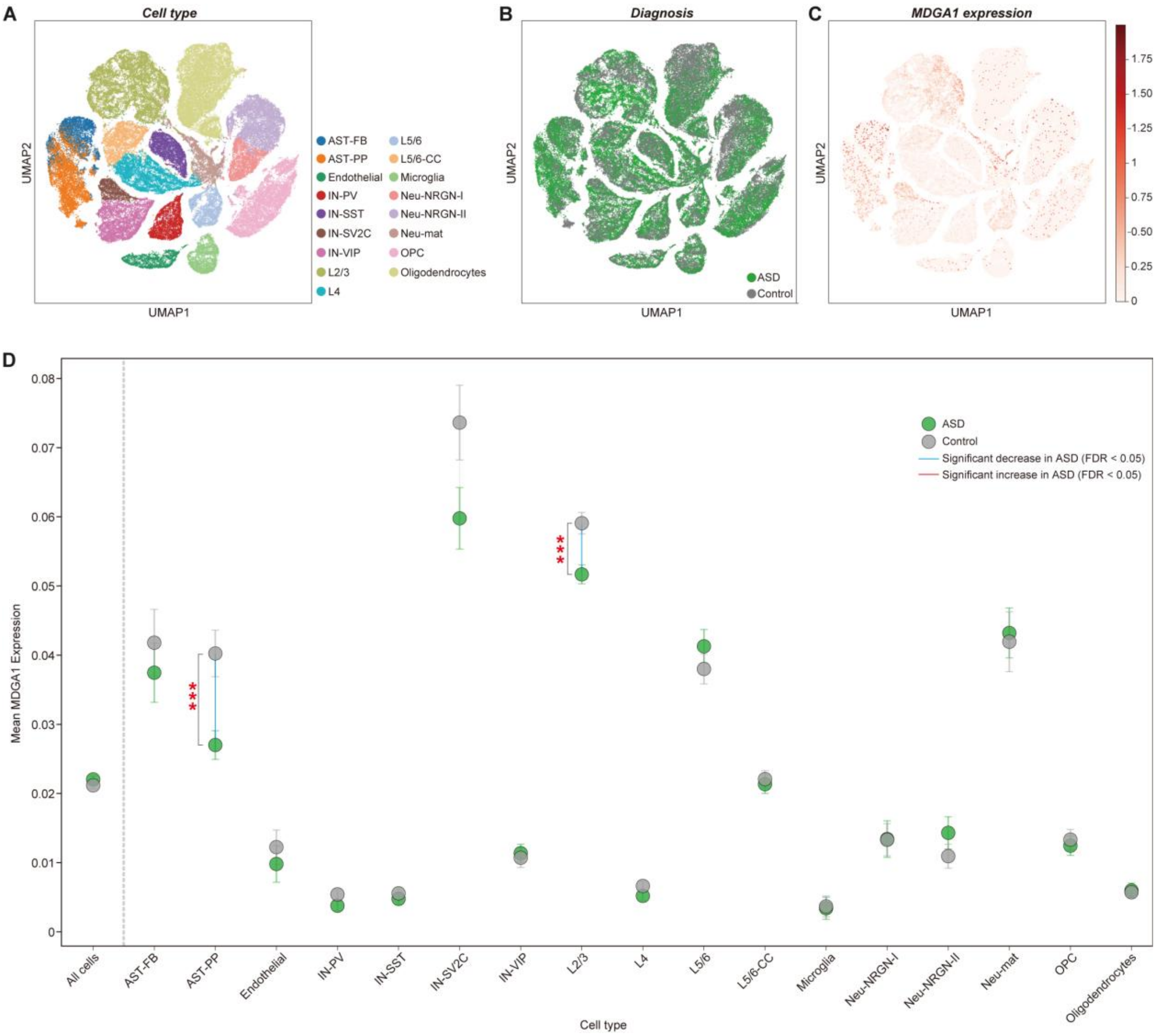

Appendix Figure S3. ***MDGA1*** **expression in individuals with ASD versus control subjects.** (**A–C**) UMAP visualization of single-nucleus RNA sequencing data, showing cell-type classification (**A**), diagnosis status (**B**), and *MDGA1* expression levels (**C**).

(**D**) Dot plots comparing mean MDGA1 expression levels across various cell types between ASD (green) and control (gray) samples. Error bars represent SEMs. Statistical significance is indicated by colored lines (blue: significantly downregulated in ASD; red: significantly upregulated in ASD; FDR < 0.05). *** indicates highly significant differences (FDR < 0.01).

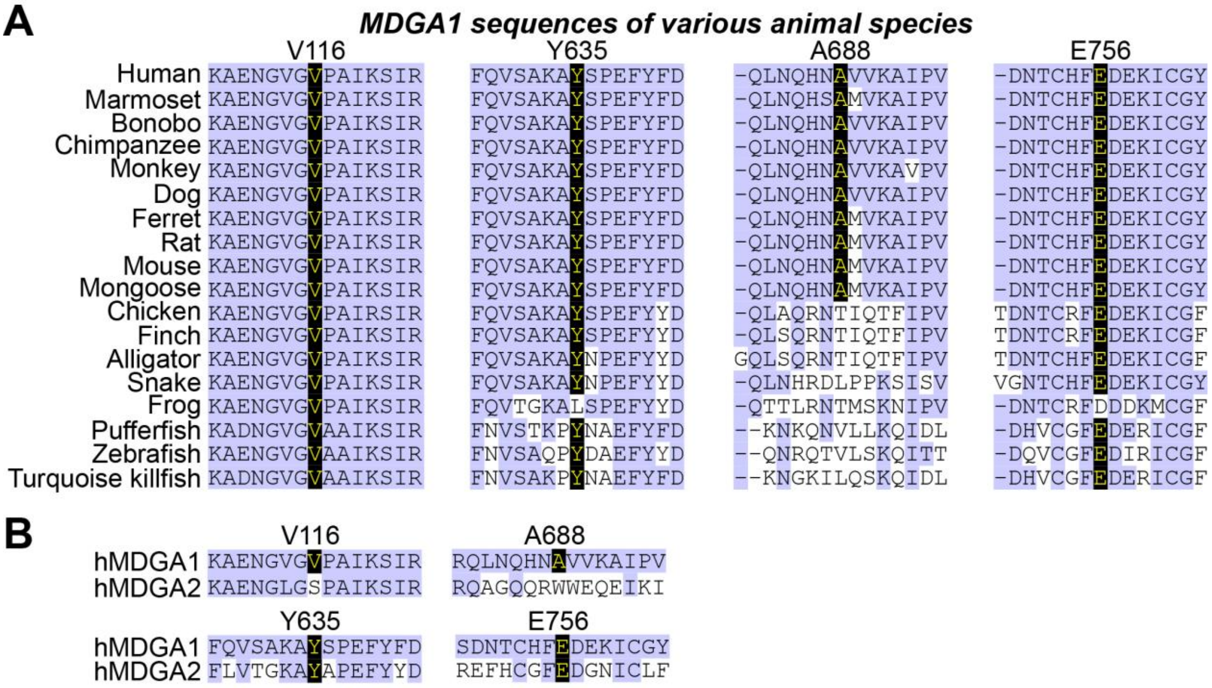

Appendix Figure S4. Sequence comparison and analysis of MDGA1 across different species.

(**A**) Multiple sequence alignment of MDGA1 from 18 vertebrate species. The sequences are compared for the presence of conserved and variable regions. Specific amino acid residues of interest in this study (V116, Y635, A688 and E756) are highlighted across species, including human, mouse, rat, dog, zebrafish, chicken, alligator, marmoset, finch, ferret, turquoise killifish, bonobo, chimpanzee, snake, mongoose, pufferfish, monkey, and frog.

(**B**) Pairwise comparison of MDGA1 and MDGA2 sequences. The sequences are aligned to show conserved and divergent regions between the two MDGA family proteins, with specific attention given to the amino acids V116, Y635, A688 and E756. Notably, Y635 and E756 are shared between the paralogs, whereas V116 and A688 show divergence, indicating that the latter residues may have subtype-specific roles in synaptic function.

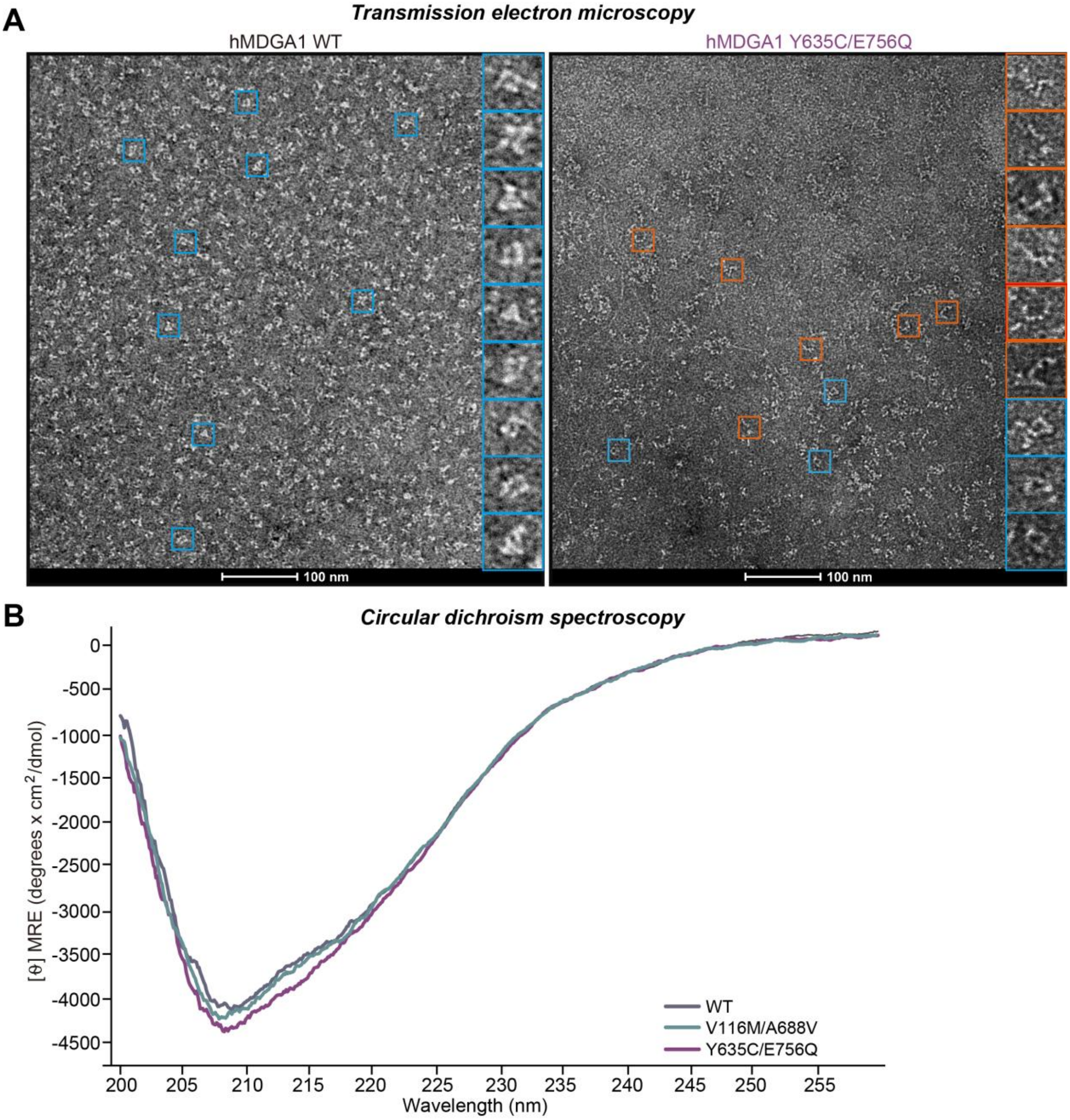

Appendix Figure S5. Negative-stain electron microscopy of the full ecto-domain of hMDGA1 WT and hMDGA1 Y635C/E756Q protein.

(**A**) Representative negative-stained electron microscopy images of the full ectodomain of human MDGA1 WT (**left**) and Y635C/E756Q mutant (**right**). The right insets show close-up views corresponding to the square boxes in the raw micrographs. The closed triangular shape of MDGA1 WT/mutant and the linear shape of MDGA1 mutant are indicated with blue and red boxes, respectively. While the majority of WT proteins exhibited a compact triangular conformation, the mutant population displayed both similar triangular structures and a subset of elongated, linear conformations, suggesting that the mutations introduced structural heterogeneity. Scale bar, 100 nm. (**B**) Far-UV circular dichroism (CD) spectra of MDGA1 WT and the ASD-associated MDGA1 mutant proteins. The CD spectra are similar, indicating that the secondary structure content of the MDGA1 variants is comparable to that of MDGA1 WT.

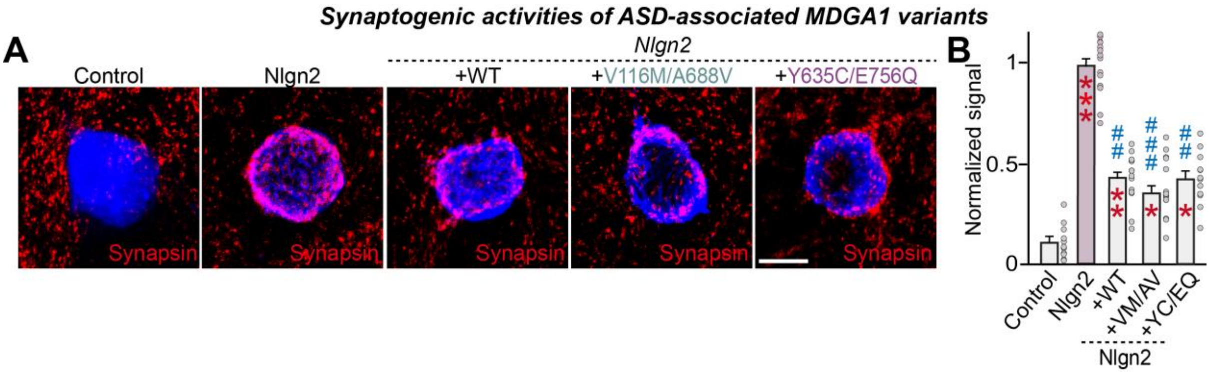

Appendix Figure S6. Analysis of the synapse-suppressing activities of the ASD-associated MDGA1 variants.

(**A** and **B**) Effects of MDGA1 WT or its variants on the synaptogenic activities of Nlgn2. HEK293T cells expressing the indicated proteins were co-cultured with hippocampal neurons without or with coexpression of the indicated MDGA1 WT or its splice variants. Representative images (**A**) of co-cultures immunostained with antibodies against EGFP or HA (blue) and synapsin I (red). Scale bar, 10 μm (applies to all images). Quantitation (**B**) of heterologous synapse-formation assays, determined by calculating the ratio of synapsin to EGFP/HA fluorescence signals. Data are means ± SEMs (**p* < 0.05, ***p* < 0.01, ****p* < 0.001, ##*p* < 0.01, ###*p* < 0.001; # indicates statistical comparisons with their counterparts; nonparametric Kruskal-Wallis test with Dunn's *post hoc* test; n = 11–14 cells/group).

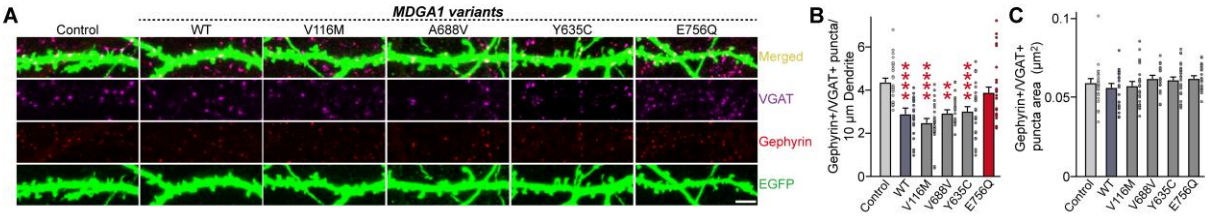

Appendix Figure S7. Effects of overexpression of the ASD-associated MDGA1 variants on GABAergic synapses in cultured hippocampal neurons.

(**A**) Representative confocal images of hippocampal neurons transfected at DIV7 with EGFP (green) and either MDGA1 WT or the indicated MDGA1 variants (V116M, Y635C, A688V and E756Q). Neurons were immunostained at DIV14 for VGAT (magenta) and gephyrin (red). EGFP (green) marks the transfected neurons. Scale bar, 10 µm.

(**B**) Quantification of gephyrin$^+$/VGAT$^+$ puncta density per 10-µm dendritic segment in hippocampal neurons expressing control, MDGA1 WT or the indicated MDGA1 variant. Data are presented as the number of gephyrin$^+$/VGAT$^+$ puncta per 10 µm of dendrite. While MDGA1 WT reduced the density of inhibitory synaptic puncta compared to control levels, the E756Q variant failed to suppress inhibitory synapse density to a similar extent, suggesting that the variant exhibited a partial loss of synapse-suppressing activity. Data are presented as means ± SEMs (n = 19–36 neurons/group; **$p < 0.01$, ****$p < 0.0001$; nonparametric Kruskal-Wallis test with Dunn's *post hoc* test).

(**C**) Quantification of gephyrin$^+$/VGAT$^+$ puncta area in hippocampal neurons expressing control, MDGA1 WT or the indicated MDGA1 variant. No significant differences were observed among groups. Data are presented as means ± SEMs (n = 19–36 neurons/group).

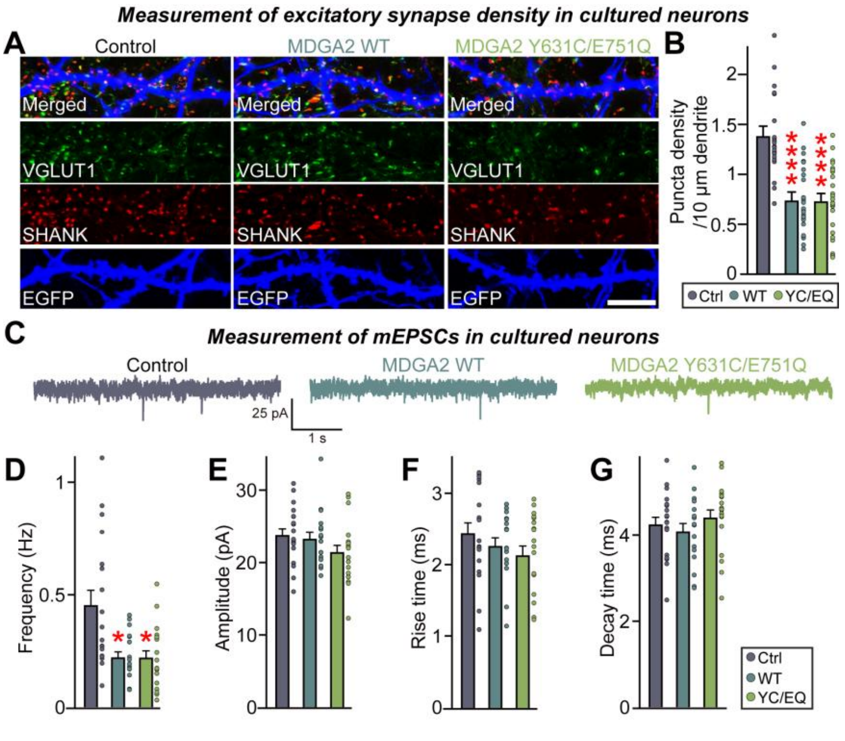

Appendix Figure S8. Effects of overexpression of MDGA2 variants (equivalent MDGA1 residues associated with ASDs were introduced) on glutamatergic synapses in cultured hippocampal neurons.

(**A**) Representative images of hippocampal neurons transfected with EGFP (green) and either MDGA2 WT or MDGA2 Y631C/E751Q variant. Neurons were immunostained for VGLUT1 (magenta) and SHANK (red). EGFP (green) marks the transfected neurons. Merged images show the colocalization of VGLUT1 and Shank. Scale bar, 10 µm.

(**B**) Quantification of VGLUT1$^+$/Shank$^+$ puncta density per 10-µm of dendrite of hippocampal cultured neurons expressing control, MDGA2 WT, and MDGA2 Y631C/E751Q. MDGA2 WT and the Y631C/E751Q mutant significantly reduced excitatory synapse density relative to control levels, with no difference between the WT and mutant groups, suggesting that the introduced mutations do not disrupt the synapse-suppressive function of MDGA2. Data are presented as means ± SEMs (n = 21–25 neurons/group; **** $p < 0.0001$; nonparametric Kruskal-Wallis test with Dunn's *post hoc* test).

(**C–G**) Representative mEPSCs traces (**C**) and quantification of frequency (**D**), amplitude (**E**), rise time (**F**) and decay time (**G**) of mEPSCs from cultured neurons transfected with MDGA2 WT or its variants. No significant difference was detected between WT and the mutants in any examined parameter. Data are presented as means ± SEMs (n = 16–19 neurons/group; *$p < 0.05$; ANOVA with a nonparametric Kruskal-Wallis test).

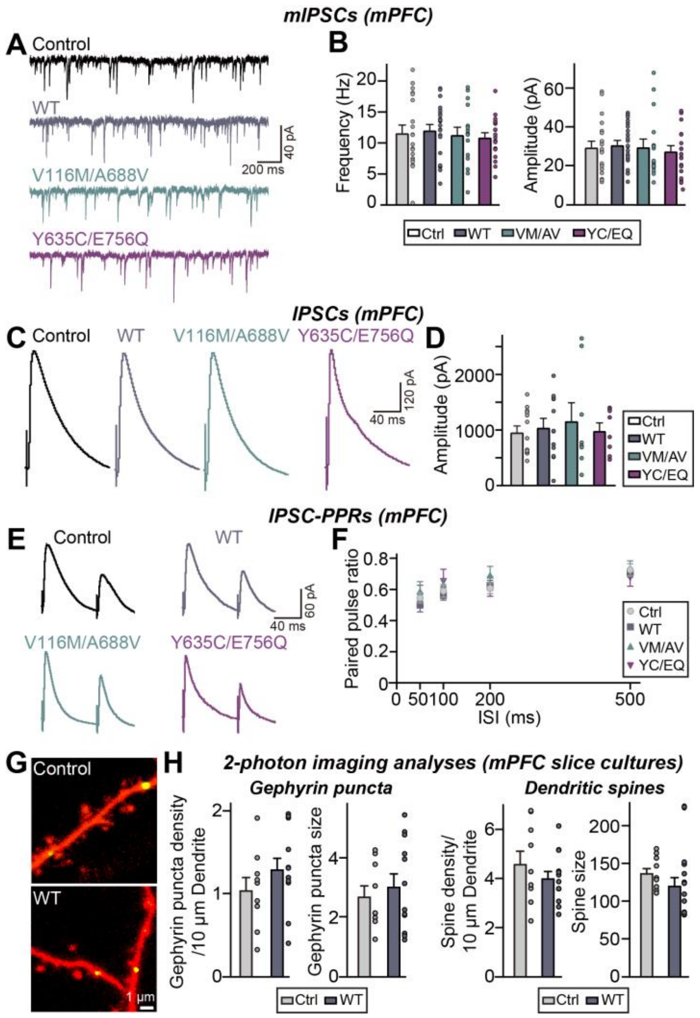

Appendix Figure S9. Effects of overexpression of the ASD-associated MDGA1 variants on GABAergic synapses in adult mPFC layer II/III pyramidal neurons.

(**A** and **B**) Whole-cell recordings of mIPSCs from adult mPFC layer II/III pyramidal neurons expressing control, MDGA1 WT, MDGA1 V116M/A688V or MDGA1 Y635C/E756Q. Representative traces (**A**) and averages of mIPSC frequencies and amplitudes (**B**; control, n = 20/5; WT, n = 22/5; V116M/A688V, n = 16/4; Y635C/E756Q, n = 20/5, where 'n' denotes the number of cells/mice). No significant differences were detected in mIPSC frequency or amplitude across groups. Data are presented as means ± SEMs.

(**C** and **D**) Recordings of eIPSCs from adult mPFC layer II/III pyramidal neurons expressing control, MDGA1 WT, MDGA1 V116M/A688V or MDGA1 Y635C/E756Q. Representative traces (**C**) and average of eIPSC amplitudes (**D**; control, n = 13/4; WT, n = 12/4; V116M/A668V, n = 8/3; Y635C/E756Q, n = 8/3). No significant effects of MDGA1 overexpression or mutations were observed. Data are presented as means ± SEMs.

(**E** and **F**) Recordings of eIPSC-PPRs from adult hippocampal CA1 pyramidal neurons expressing control, MDGA1 WT, MDGA1 V116M/A688V or MDGA1 Y635C/E756Q. Representative traces (**E**) and average of eIPSC-PPRs (**F**; control, n = 13/4; WT, n = 12/4; V116M/A668V, n = 8/3; Y635C/E756Q, n = 8/3). No significant differences in eIPSC-PPRs were observed between experimental groups. Data are presented as means ± SEMs (Kruskal–Wallis test followed by Dunn's multiple comparison test).

(**G**) Two-photon images of dendritic segments from mPFC layer II/III pyramidal neurons co-transfected with tdTomato, gephyrin intrabody-GFP, and control or MDGA1 WT plasmid.
(**H**) Quantitative analysis of gephyrin^{+} puncta density and size (**left**), and dendritic spine density and size (**right**), showing no evidence that structural alterations were induced by MDGA1 WT overexpression in adult mPFC neurons. Data are presented as means ± SEMs (n = 9–12 cells/group).

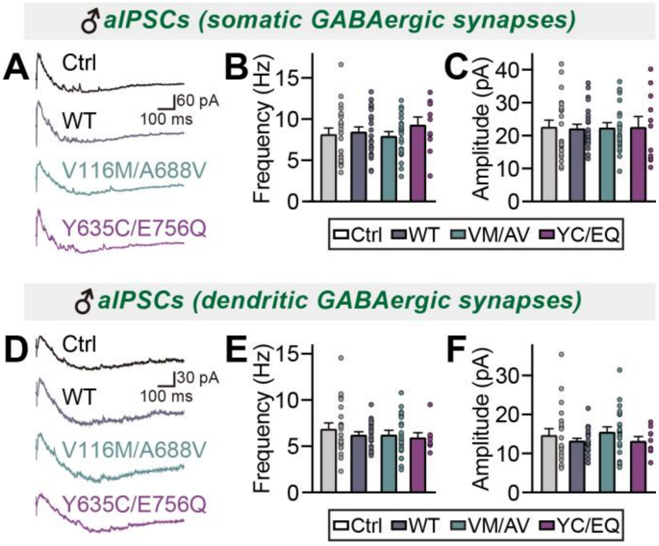

Appendix Figure S10. Effects of overexpression of the ASD-associated MDGA1 variants on asynchronous evoked GABAergic transmission in adult hippocampal CA1 pyramidal neurons.

(**A–C**) Representative traces (**A**) and averages of somatic aIPSC frequencies (**B**) and amplitudes (**C**) from CA1 pyramidal neurons (Control, n = 22/5; WT, n = 25/5; V116M/A688V, n = 23/5; Y635C/E756Q, n = 11/4; 'n' denotes number of cells/mice). No significant differences in aIPSC properties were observed across experimental groups.

(**D–F**) Representative traces (**D**) and averages of dendritic aIPSC frequencies (**E**) and amplitudes (**F**) from CA1 pyramidal neurons (Control, n = 21/5; WT, n = 25/5; V116M/A688V, n = 23/5; Y635C/E756Q, n = 9/4). Paired electric stimulation at 10 Hz was used for measuring aIPSCs. No significant differences were detected across experimental groups. Data are presented as means ± SEMs (Kruskal–Wallis test followed by Dunn's multiple comparison test).

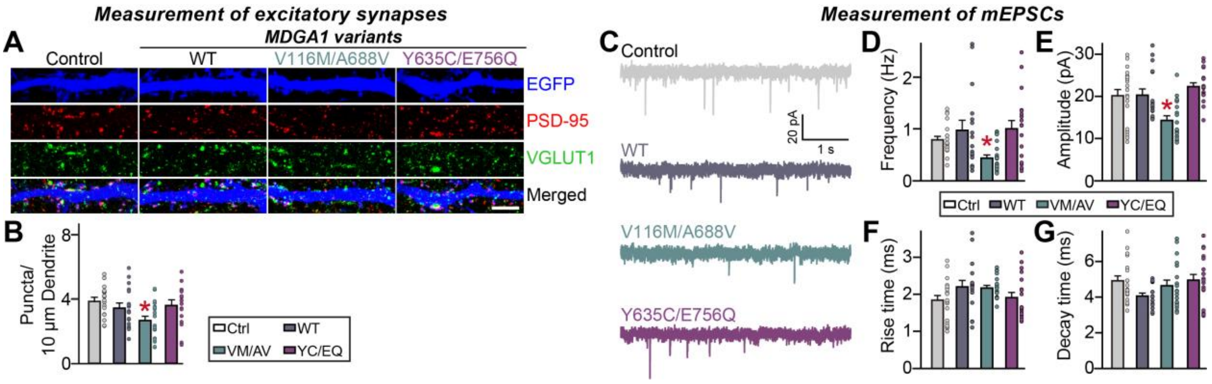

Appendix Figure S11. Effects of overexpression of the ASD-associated MDGA1 variants on glutamatergic synapses in cultured hippocampal neurons.

(**A** and **B**) Representative confocal images (**A**) and summary graphs (**B**) showing the density of glutamatergic synaptic puncta of cultured hippocampal neurons transfected at DIV7 with the indicated full-length MDGA1 expression construct and immunostained at DIV14 with antibodies to VGLUT1 and PSD-95 (excitatory synaptic markers) and EGFP. Notably, only the V116M/A688V variant caused a significant reduction in glutamatergic synaptic puncta density. Data are presented as means ± SEMs (*$p < 0.05$; ANOVA with non-parametric Kruskal-Wallis test; n = 17–20 images/group). Scale bar, 10 μm (applies to all images).

(**C–G**) Representative mEPSCs traces (**C**) and quantification of frequency (**D**), amplitude (**E**), rise time (**F**) and decay time (**G**) of mEPSCs from cultured neurons transfected with MDGA1 or its variants. A significant reduction in mEPSC frequency was observed in neurons expressing the V116M/A688V variant. Data are presented as means ± SEMs (n = 16–21 neurons/group; *$p < 0.05$; ANOVA with nonparametric Kruskal-Wallis test).

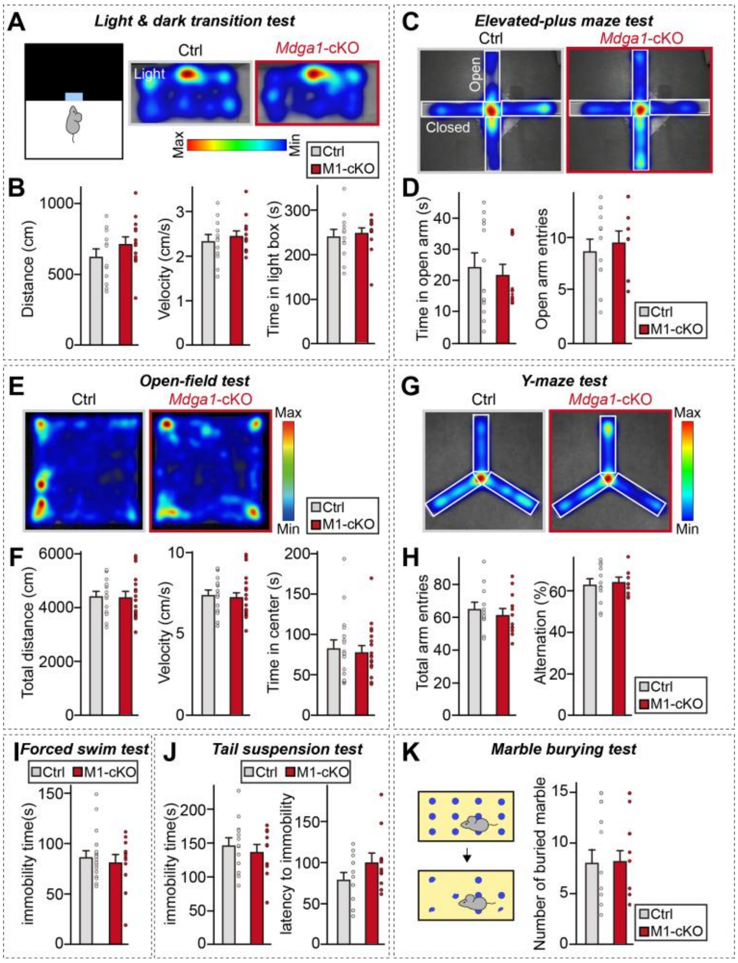

Appendix Figure S12. Analysis of behaviors of adult male *Mdga1*-cKO mice.

(**A** and **B**) Light and dark transition test results showing time spent in the light box, total distance traveled, and velocity for control and *Mdga1*-cKO mice. No significant changes in time spent in the light compartment, total distance traveled, or movement velocity were observed compared with littermate controls. Data are presented as means ± SEMs (n = 12–14 mice/group). (**C** and **D**) Elevated-plus maze test results showing time spent in the open arms and number of open arm entries for control and *Mdga1*-cKO mice. No overt anxiety-like phenotypes were observed in both control and *Mdga1*-cKO mice. Data are presented as means ± SEMs (n = 9–12 mice/group). (**E** and **F**) Open-field test results showing no changes in total distance traveled, time spent in the center, and velocity for control and *Mdga1*-cKO mice, indicating that locomotion and exploratory behaviors were unaltered. Data are presented as means ± SEMs (n = 17–18 mice/group). (**G** and **H**) Y-maze test results showing no changes in total arm entries and alternation percentage for control and *Mdga1*-cKO mice, indicating that intact working memory was observed in all experimental groups. Data are presented as means ± SEMs (n = 12 mice/group).

(**I**) Forced swim test results showing no differences in total immobility time for control and *Mdga1*-cKO mice. Data are presented as means ± SEMs (n = 11–18 mice/group). (**J**) Tail suspension test results showing no changes in the latency to immobility and total immobility time for control and *Mdga1*-cKO mice. Data are presented as means ± SEMs (n = 11–13 mice/group). (**K**) Marble burying test results showing no marked changes in the number of marbles buried by control and *Mdga1*-cKO mice, indicating that there was no alteration of repetitive behaviors. Data are presented as means ± SEMs (n = 13–15 mice/group).

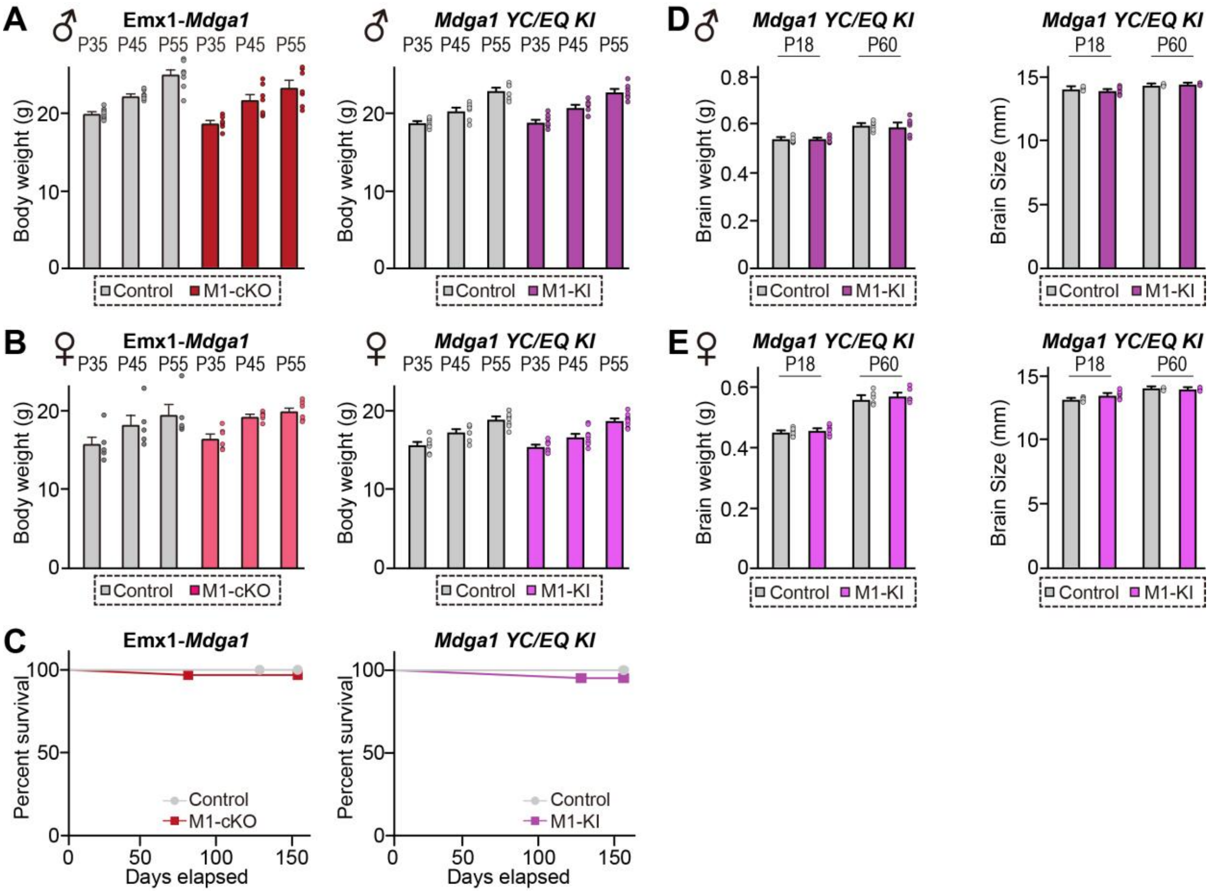

Appendix Figure S13. Analysis of body weight, brain weight, and the survival curve for *Mdga1*-cKO and *Mdga1*$^{Y636C/E751Q}$ KI mice.

(**A**) Body weight measurements of male MDGA1 conditional knockout (cKO) (**left**) and MDGA1 Y636C/E751Q knock-in (KI) (**right**) mice compared to control mice, showing no notable changes. Body weight was recorded at postnatal days P35, P45, and P55. Data are presented as means ± SEMs (n = 7 mice/group).

(**B**) Body weight measurements of female MDGA1 conditional knockout (cKO) (**left**) and MDGA1 Y636C/E751Q KI (**right**) mice compared to control mice, showing no significant differences in body weight compared to controls across the same postnatal time points. Body weight was recorded at postnatal days P35, P45, and P55. Data are presented as means ± SEMs (n =5–9 mice/group).

(**C**) Survival curves showing indistinguishable survival probability between male *Mdga1*-cKO and *Mdga1*$^{Y636C/E751Q}$ KI mice compared to control mice. The survival probability was tracked over time up to 150 days. Survival curves were generated using the Kaplan–Meier method (n = 20–32 mice/group).

(**D**) Quantification of brain weights and size of male control and *Mdga1*$^{Y636C/E751Q}$ KI mice (P18 or P60). Data are means ± SEMs ('n' denotes the number of mice; control and *Mdga1*$^{Y636C/E751Q}$ KI, n = 6).

(**E**) Same as in (**D**), except that female counterparts were analyzed. Data are means ± SEMs ('n' denotes the number of mice; control and *Mdga1*$^{Y636C/E751Q}$ KI, n = 6).

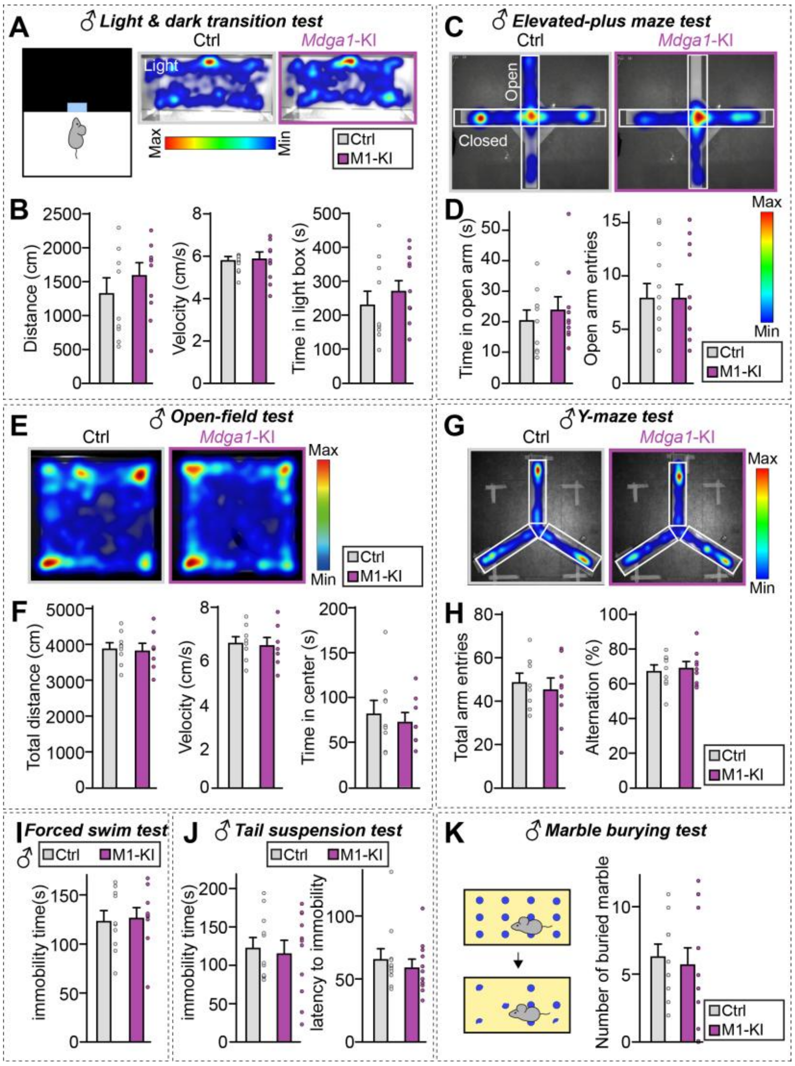

Appendix Figure S14. Analysis of behaviors of adult male *Mdga1*$^{Y636C/E751Q}$ KI mice.

(**A** and **B**) Light and dark transition test results showing no significant differences in time spent in the light box (**A**), total distance traveled, and velocity for male control and *Mdga1*$^{Y636C/E751Q}$ KI mice (**B**). Data are presented as means ± SEMs (n = 9–10 mice/group). (**C** and **D**) Elevated-plus maze test results showing no changes in time spent in the open arms (**C**) and number of open arm entries for male control and *Mdga1*$^{Y636C/E751Q}$ KI mice (**D**). Data are presented as means ± SEMs (n = 10 mice/group). (**E** and **F**) Open-field test results showing no changes in total distance traveled (**E**), time spent in the center, and velocity for male control and *Mdga1*$^{Y636C/E751Q}$ KI mice (**F**). Data are presented as means ± SEMs (n = 8–9 mice/group). (**G** and **H**) Y-maze test results showing no changes in total arm entries (**G**) and alternation percentage for male control and *Mdga1*$^{Y636C/E751Q}$ KI mice (**H**). Data are presented as means ± SEMs (n = 9–10 mice/group). (**I**) Forced swim test results showing no changes in total immobility time for male control and *Mdga1*$^{Y636C/E751Q}$ KI mice. Data are presented as means ± SEMs (n = 7 mice/group). (**J**) Tail suspension test results showing no changes in the latency to immobility and total immobility time for male control and *Mdga1*$^{Y636C/E751Q}$ KI mice. Data are presented as means ± SEMs (n = 7 mice/group). (**K**) Marble burying test results showing no changes in the number of marbles buried by male control and *Mdga1*$^{Y636C/E751Q}$ KI mice. Data are presented as means ± SEMs (n = 11–13 mice/group.)

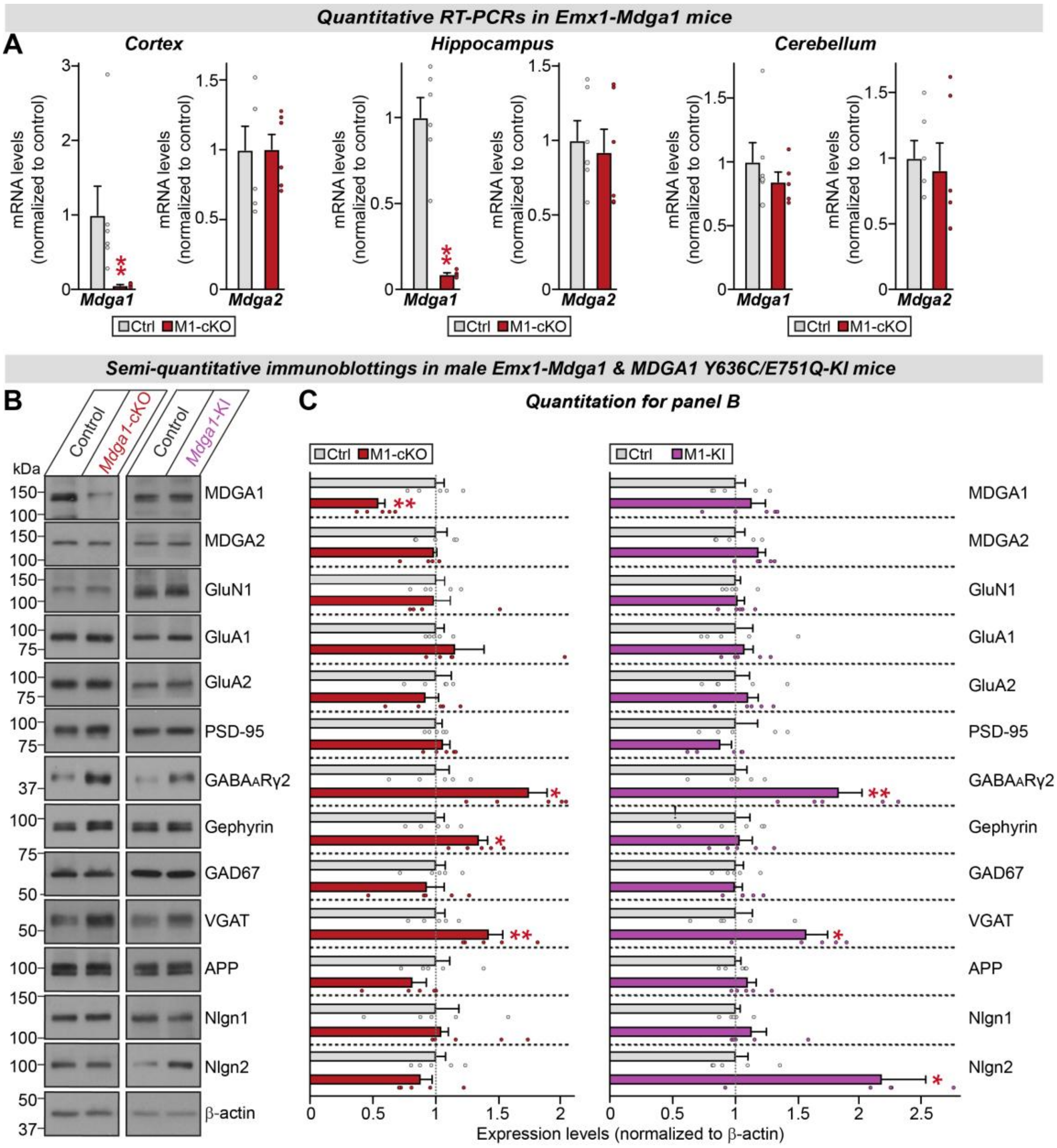

Appendix Figure S15. Analysis of expression levels of *Mdga* mRNAs and MDGA proteins as well as expression levels of other synaptic proteins in the hippocampus of male *Mdga1*-cKO and male *Mdga1*$^{Y636C/E751Q}$ KI mice.

(**A**) Quantitative RT-PCR analysis of *Mdga1* and *Mdga2* mRNA levels in the hippocampus, cortex, and cerebellum of adult male *Mdga1*-cKO mice. Data are normalized to control levels and presented as means ± SEMs (n = 5–6 mice/group; **$p < 0.01$, Mann–Whitney *U* test).

(**B**) Semi-quantitative immunoblotting of various synaptic proteins in the hippocampus of adult male control, *Mdga1*-cKO and *Mdga1*$^{Y636C/E751Q}$ KI mice. β-actin was used as a loading control for normalization.

(**C**) Semi-quantitative analysis of immunoblotting results shown in panel (**B**). Expression levels of various proteins are normalized to β-actin and presented as fold changes relative to control mice. Data are presented as means ± SEMs (n = 5 mice/group; *$p < 0.05$, **$p < 0.01$; Mann–Whitney *U* test).

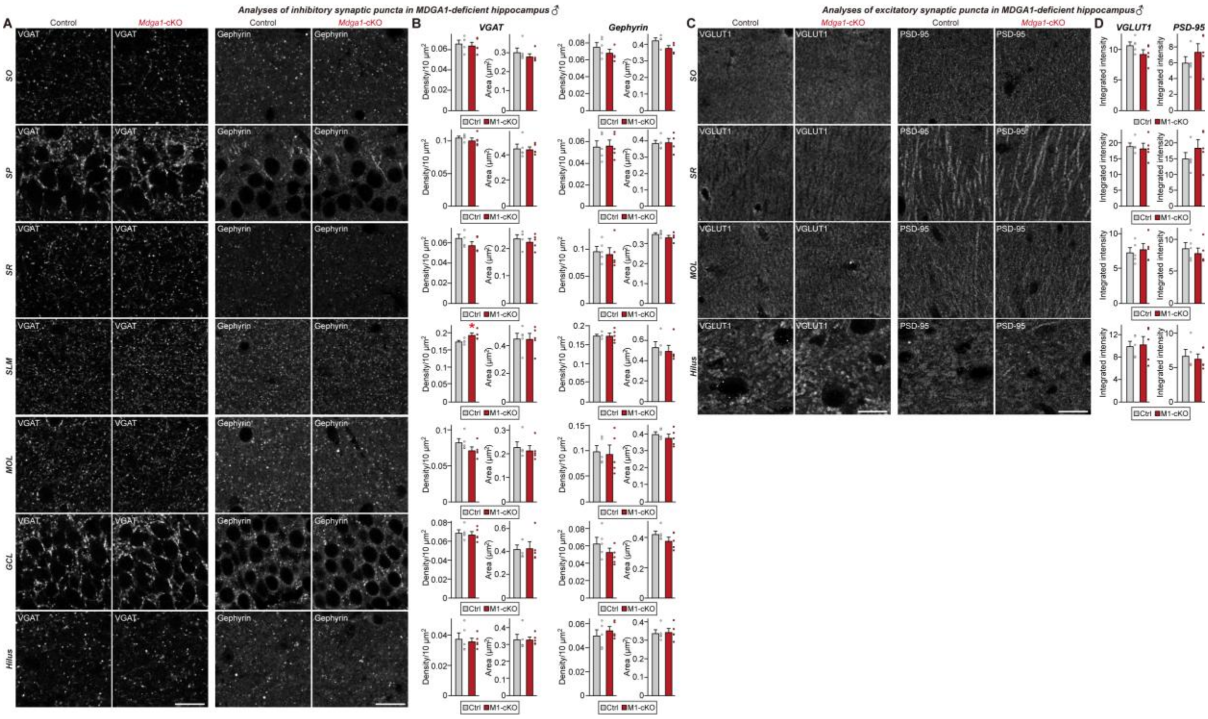

Appendix Figure S16. Analysis of glutamatergic and GABAergic synaptic puncta in the hippocampus of adult male *Mdga1*-cKO mice.

(**A**) Representative images of GABAergic synaptic puncta in the hippocampus of male control and *Mdga1*-cKO mice. Neurons were immunostained for VGAT and gephyrin in various hippocampal layers. Abbreviation: SO, *stratum oriens*; SR, *stratum radiatum*; SLM, *stratum lacunosum moleculare*; SP, *stratum pyramidale*; MOL, molecular layer; and GCL, granule cell layer. Scale bar, 10 μm (applies to all images).

(**B**) Quantification of GABAergic synaptic puncta density and area in the hippocampus of male control and *Mdga1*-cKO mice. Data are presented as means ± SEMs (n = 5 mice/group; **p* < 0.05; Mann–Whitney *U* test).

(**C**) Representative images of glutamatergic synaptic puncta in the hippocampus of male control and *Mdga1*-cKO mice. Neurons were immunostained for VGLUT1 and PSD-95 in various hippocampal layers. Scale bar, 10 μm (applies to all images).

(**D**) Quantification of glutamatergic synaptic puncta density and area in the hippocampus of male control and *Mdga1*-cKO mice. Data are presented as means ± SEMs (n = 5 mice/group).

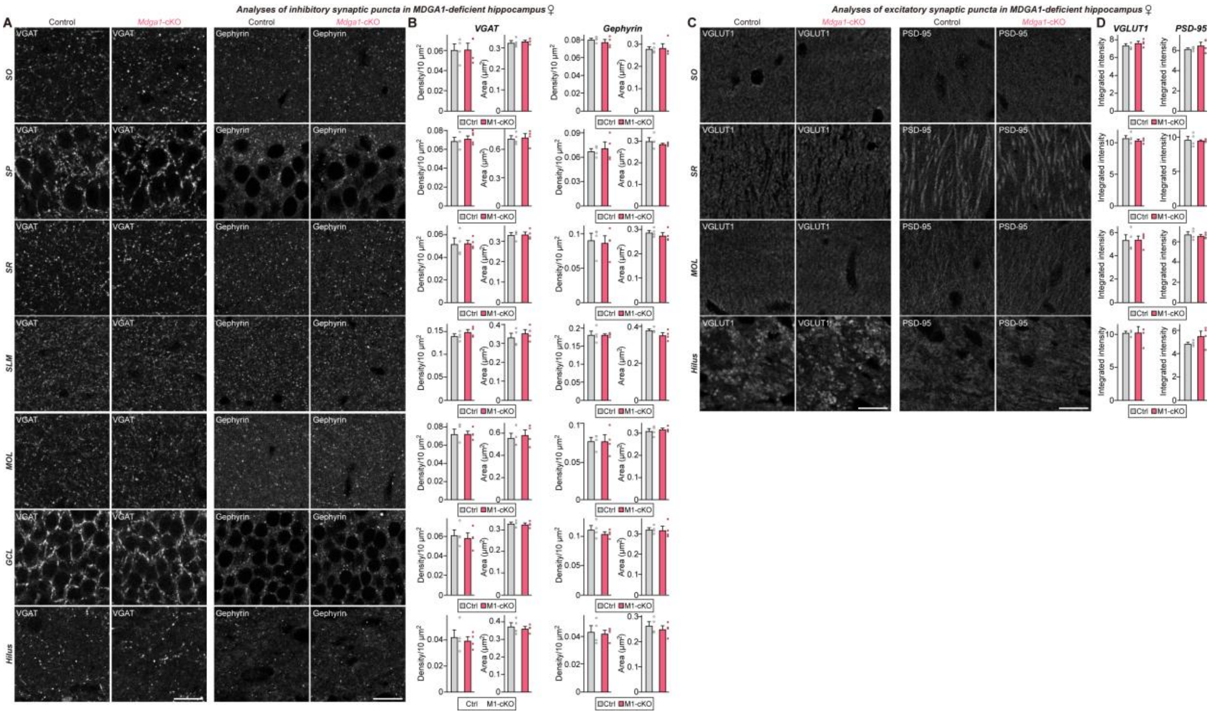

Appendix Figure S17. Analysis of glutamatergic and GABAergic synaptic puncta in the hippocampus of adult female *Mdga1*-cKO mice.

(**A**) Representative images of GABAergic synaptic puncta in the hippocampus of female control and *Mdga1*-cKO mice. Scale bar, 10 μm (applies to all images).

(**B**) Quantification of GABAergic synaptic puncta density and area in the hippocampus of female control and *Mdga1*-cKO mice. Data are presented as means ± SEMs (n = 4 mice/group).

(**C**) Representative images of glutamatergic synaptic puncta in the hippocampus of female control and *Mdga1*-cKO mice. Neurons were immunostained for VGLUT1 and PSD-95 in various hippocampal layers. Scale bar, 10 μm (applies to all images).

(**D**) Quantification of glutamatergic synaptic puncta density and area in the hippocampus of female control and *Mdga1*-cKO mice. Data are presented as means ± SEMs (n = 4 mice/group).

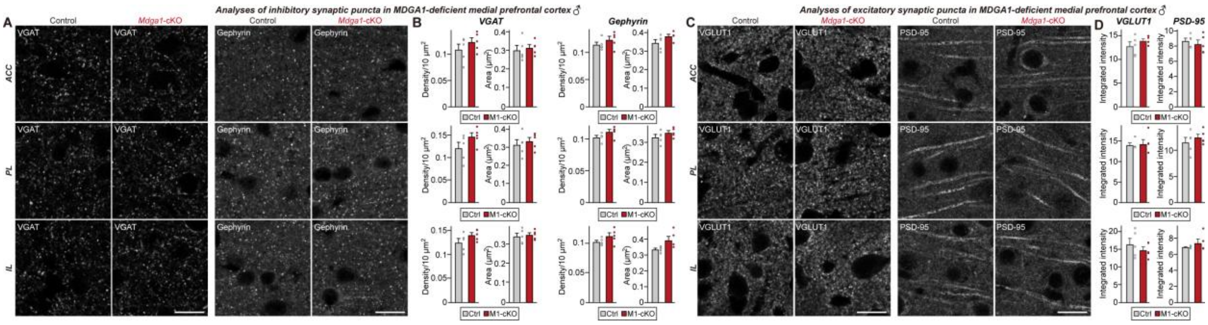

Appendix Figure S18. Analysis of glutamatergic and GABAergic synaptic puncta in the mPFC of adult male *Mdga1*-cKO mice.

(**A**) Representative images of GABAergic synaptic puncta in the mPFC of male control and *Mdga1*-cKO mice. Neurons were immunostained for VGAT and gephyrin in different layers of the mPFC. Abbreviations: ACC, anterior cingulate cortex; IL, infralimbic cortex; PL, prelimbic cortex. Scale bar, 10 μm (applies to all images).

(**B**) Quantification of GABAergic synaptic puncta density and area in the mPFC of male control and *Mdga1*-cKO mice. Data are presented as means ± SEMs (n = 5 mice/group).

(**C**) Representative images of glutamatergic synaptic puncta in the mPFC of male control and *Mdga1*-cKO mice. Neurons were immunostained for VGLUT1 and PSD-95 in different layers of the mPFC. Scale bar, 10 μm (applies to all images).

(**D**) Quantification of glutamatergic synaptic puncta density and area in the mPFC of male control and *Mdga1*-cKO mice. Data are presented as means ± SEMs (n = 5 mice/group).

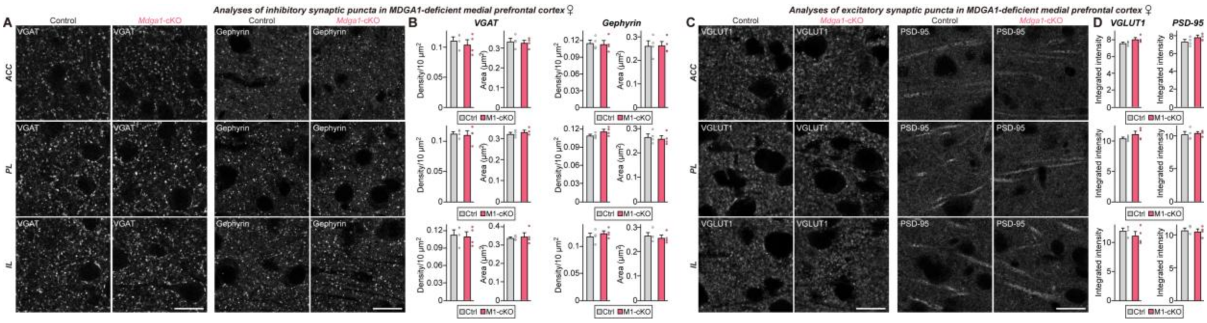

Appendix Figure S19. Analysis of glutamatergic and GABAergic synaptic puncta in the mPFC of adult female *Mdga1*-cKO mice.

(**A**) Representative images of GABAergic synaptic puncta in the mPFC of female control and *Mdga1*-cKO mice. Neurons were immunostained for VGAT and gephyrin in different layers of the mPFC. Scale bar, 10 μm (applies to all images).

(**B**) Quantification of GABAergic synaptic puncta density and area in the mPFC of female control and *Mdga1*-cKO mice. Data are presented as means ± SEMs (n = 4 mice/group).

(**C**) Representative images of glutamatergic synaptic puncta in the mPFC of female control and *Mdga1*-cKO mice. Neurons were immunostained for VGLUT1 and PSD-95 in different layers of the mPFC. Scale bar, 10 μm (applies to all images).

(**D**) Quantification of glutamatergic synaptic puncta density and area in the mPFC of female control and *Mdga1*-cKO mice. Data are presented as means ± SEMs (n = 4 mice/group).

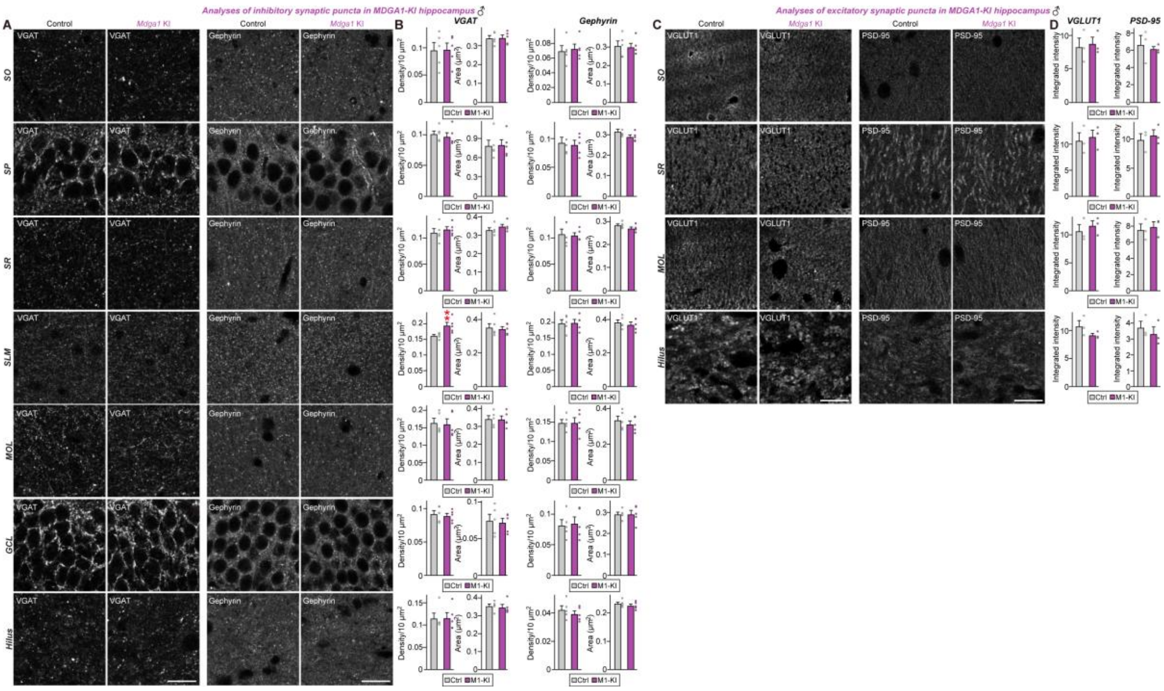

Appendix Figure S20. Analysis of glutamatergic and GABAergic synaptic puncta in the hippocampus of adult male *Mdga1*$^{\mathrm{Y636C/E751Q}}$ KI mice.

(**A**) Representative images of GABAergic synaptic puncta in the hippocampus of male control and *Mdga1*$^{\mathrm{Y636C/E751Q}}$ KI mice. Neurons were immunostained for VGAT and gephyrin in various hippocampal layers. Scale bar, 10 µm (applies to all images).

(**B**) Quantification of GABAergic synaptic puncta density and area in the hippocampus of male control and *Mdga1*$^{\mathrm{Y636C/E751Q}}$ KI mice. Data are presented as means ± SEMs (n = 5 mice/group; $^{**}p < 0.01$; Mann–Whitney *U* test).

(**C**) Representative images of glutamatergic synaptic puncta in the hippocampus of male control and *Mdga1*$^{\mathrm{Y636C/E751Q}}$ KI mice. Neurons were immunostained for VGLUT1 and PSD-95 in various hippocampal layers. Scale bar, 10 µm (applies to all images).

(**D**) Quantification of glutamatergic synaptic puncta density and area in the hippocampus of male control and *Mdga1*$^{\mathrm{Y636C/E751Q}}$ KI mice. Data are presented as means ± SEMs (n = 5 mice/group).

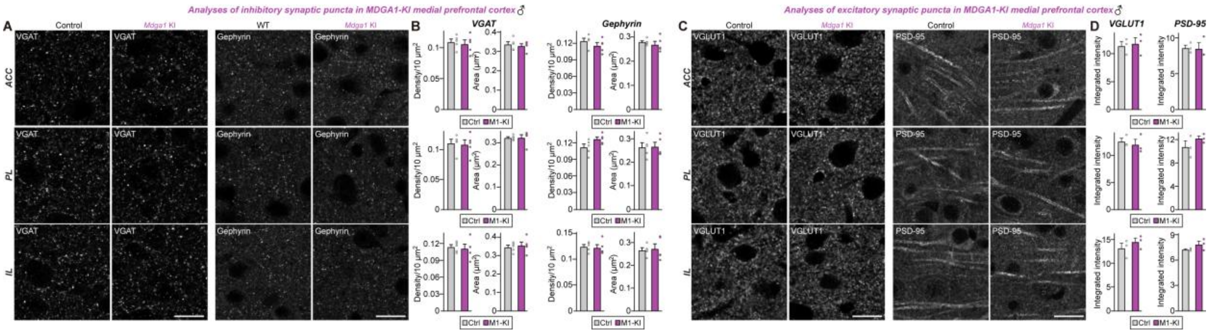

Appendix Figure S21. Analysis of glutamatergic and GABAergic synaptic puncta in the mPFC of adult male *Mdga1*$^{Y636C/E751Q}$ KI mice.

(**A**) Representative images of GABAergic synaptic puncta in the hippocampus of female control and *Mdga1*$^{Y636C/E751Q}$ KI mice. Neurons were immunostained for VGAT and gephyrin in various hippocampal layers. Scale bar, 10 μm (applies to all images).

(**B**) Quantification of GABAergic synaptic puncta density and area in the hippocampus of female control and *Mdga1*$^{Y636C/E751Q}$ KI mice. Data are presented as means ± SEMs (n = 5 mice/group).

(**C**) Representative images of glutamatergic synaptic puncta in the hippocampus of female control and *Mdga1*$^{Y636C/E751Q}$ KI mice. Neurons were immunostained for VGLUT1 and PSD-95 in various hippocampal layers. Scale bar, 10 μm (applies to all images).

(**D**) Quantification of glutamatergic synaptic puncta density and area in the hippocampus of female control and *Mdga1*$^{Y636C/E751Q}$ KI mice. Data are presented as means ± SEMs (n = 5 mice/group).

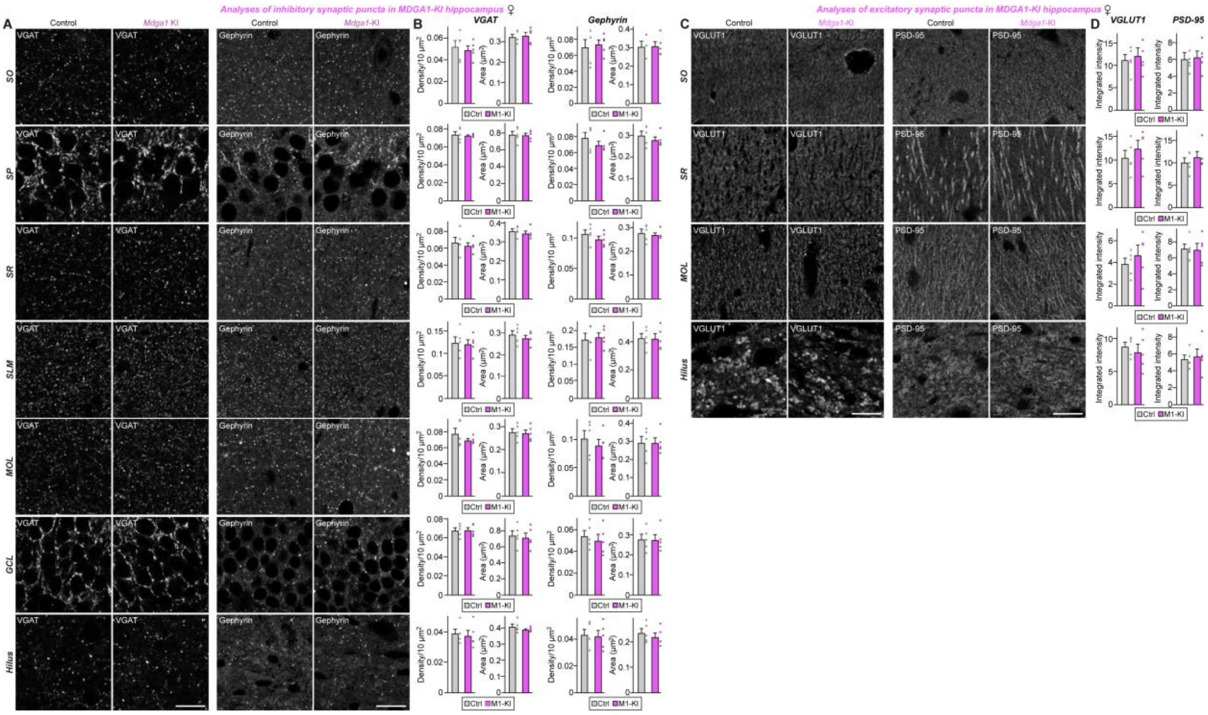

Appendix Figure S22. Analysis of glutamatergic and GABAergic synaptic puncta in the hippocampus of adult female *Mdga1*$^{Y636C/E751Q}$ KI mice.

(**A**) Representative images of GABAergic synaptic puncta in the mPFC of male control and *Mdga1*$^{Y636C/E751Q}$ KI mice. Neurons were immunostained for VGAT and gephyrin in different layers of the mPFC. Scale bar: 10 µm (applies to all images).

(**B**) Quantification of GABAergic synaptic puncta density and area in the mPFC of male control and *Mdga1*$^{Y636C/E751Q}$ KI mice. Data are presented as means ± SEMs (n = 5 mice/group).

(**C**) Representative images of glutamatergic synaptic puncta in the mPFC of male control and *Mdga1*$^{Y636C/E751Q}$ KI mice. Neurons were immunostained for VGLUT1 and PSD-95 in different layers of the mPFC. Scale bar, 10 µm (applies to all images).

(**D**) Quantification of glutamatergic synaptic puncta density and area in the mPFC of male control and *Mdga1*$^{Y636C/E751Q}$ KI mice. Data are presented as means ± SEMs (n = 5 mice/group).

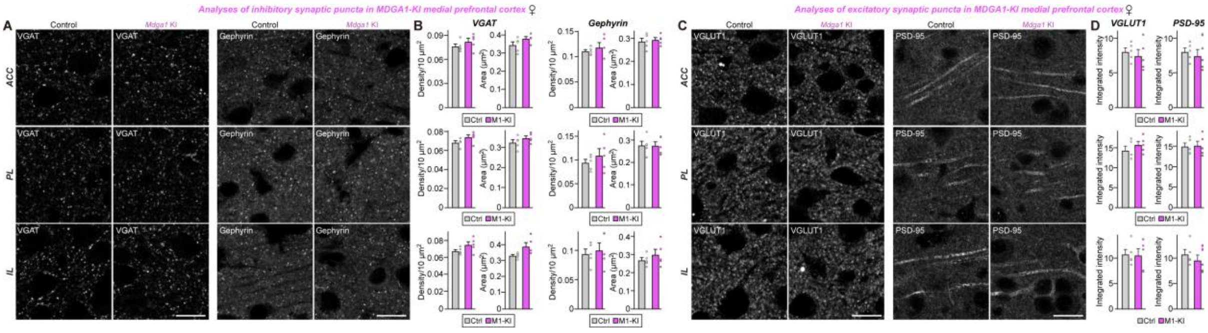

Appendix Figure S23. Analysis of glutamatergic and GABAergic synaptic puncta in the mPFC of adult female *Mdga1*$^{Y636C/E751Q}$ KI mice.

(**A**) Representative images of GABAergic synaptic puncta in the mPFC of female control and *Mdga1*$^{Y636C/E751Q}$ KI mice. Neurons were immunostained for VGAT and gephyrin in different layers of the mPFC. Scale bar, 10 μm (applies to all images).

(**B**) Quantification of GABAergic synaptic puncta density and area in the mPFC of female control and *Mdga1*$^{Y636C/E751Q}$ KI mice. Data are presented as means ± SEMs (n = 5 mice/group).

(**C**) Representative images of excitatory synaptic puncta in the mPFC of female control and *Mdga1*$^{Y636C/E751Q}$ KI mice. Neurons were immunostained for VGLUT1 and PSD-95 in different layers of the mPFC. Scale bar, 10 μm (applies to all images).

(**D**) Quantification of glutamatergic synaptic puncta density and area in the mPFC of female control and *Mdga1*$^{Y636C/E751Q}$ KI mice. Data are presented as means ± SEMs (n = 5 mice/group).

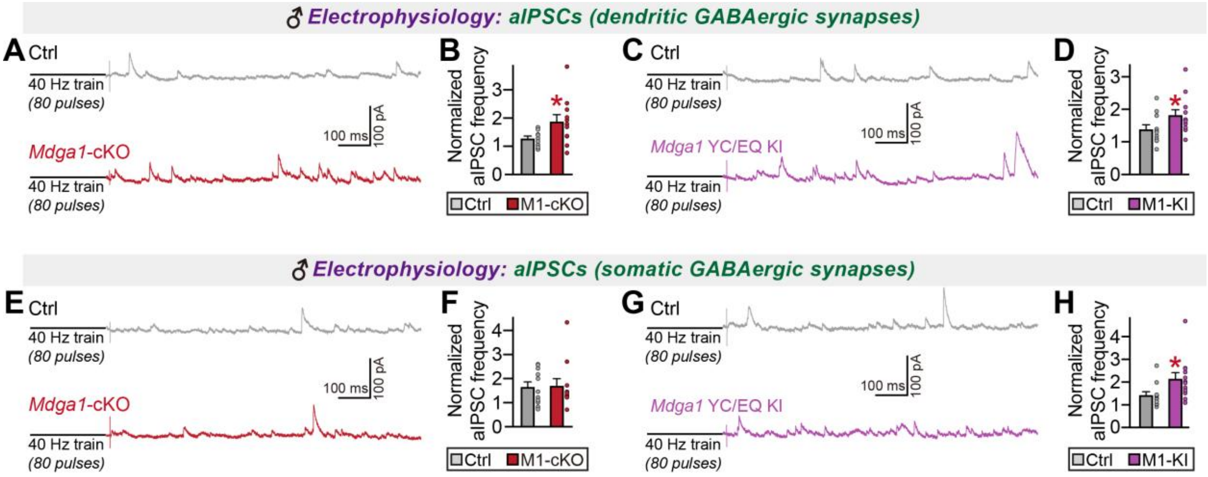

Appendix Figure S24. Analysis of asynchronous GABAergic evoked synaptic transmission in the hippocampal CA1 pyramidal neurons of adult male *Mdga1*-cKO and *Mdga1*$^{Y636C/E751Q}$ KI mice.

(**A** and **B**) Representative traces (**A**) and quantification of normalized asynchronous inhibitory postsynaptic currents (aIPSCs) frequency (**B**) recorded from dendritic GABAergic synapses in male control and *Mdga1*-cKO mice. *Mdga1*-cKO mice showed significantly increased aIPSC frequencies. Data are presented as means ± SEMs (n = 11 cells/group; *$p < 0.05$; Mann–Whitney *U* test).

(**C** and **D**) Representative traces (**C**) and quantification of normalized aIPSC frequency (**D**) in dendritic GABAergic synapses in male control and *Mdga1*$^{Y636C/E751Q}$ KI mice. *Mdga1*$^{Y636C/E751Q}$ KI exhibited significantly elevated aIPSC frequencies. Data are presented as means ± SEMs (n = 11–12 cells/group; *$p < 0.05$; Mann–Whitney *U* test).

(**E** and **F**) Representative traces (**E**) and quantification of normalized aIPSCs frequency (**F**) recorded from somatic GABAergic synapses in male control and *Mdga1*-cKO mice. No significant differences were detected. Data are presented as means ± SEMs (n = 11 cells/group).

(**G** and **H**) Representative traces (**G**) and quantification of normalized aIPSC frequency (**H**) in somatic GABAergic synapses in male control and *Mdga1*$^{Y636C/E751Q}$ KI mice. *Mdga1*$^{Y636C/E751Q}$ KI displayed significantly increased aIPSC frequencies. Data are presented as means ± SEMs (n = 11–12 cells/group; *$p < 0.05$; Mann–Whitney *U* test).

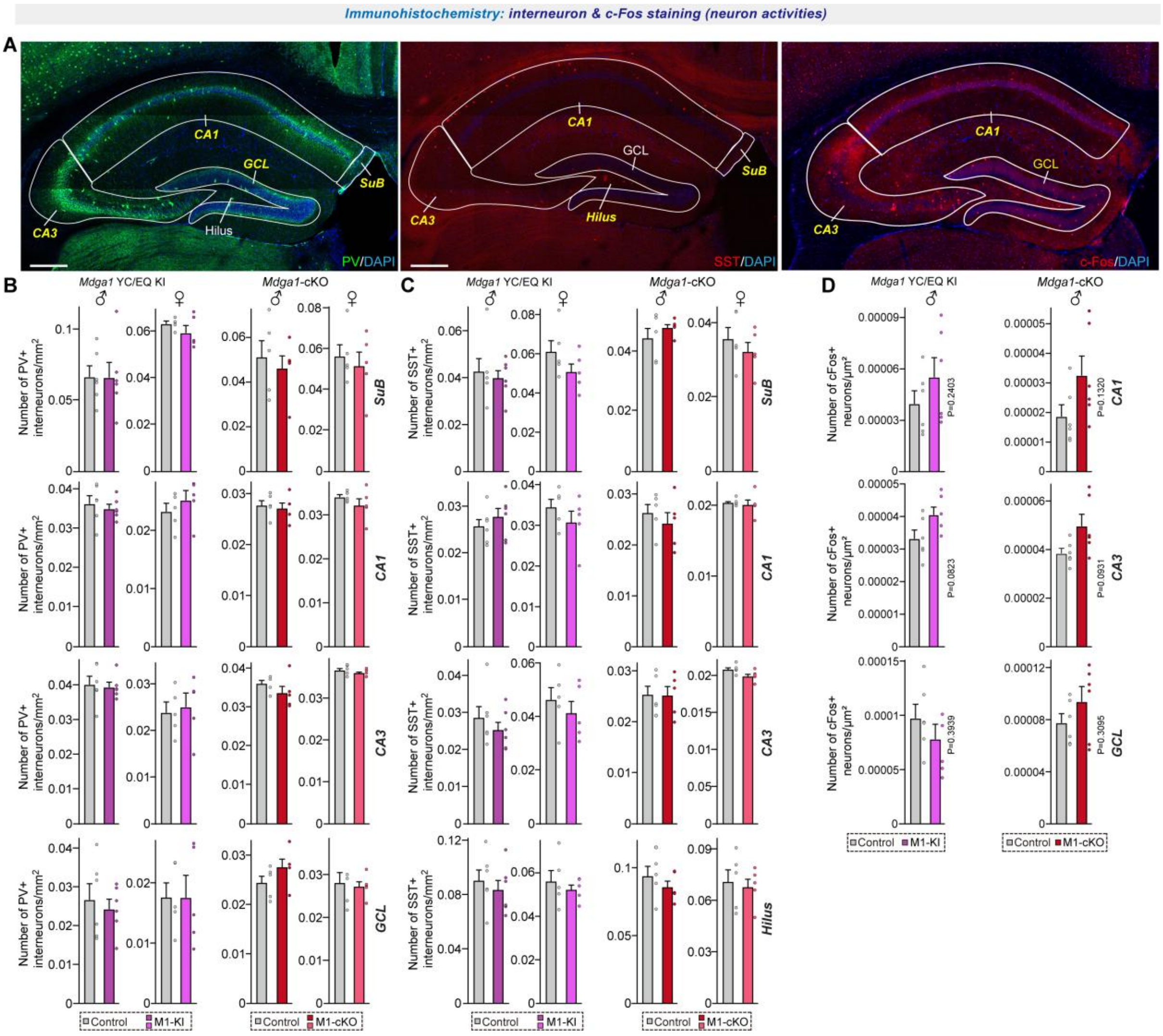

Appendix Figure S25. Analysis of density of interneurons and c-Fos⁺ neurons in the hippocampus of adult *Mdga1*-cKO and *Mdga1*$^{Y636C/E751Q}$ KI mice.

(**A**) Representative images of parvalbumin (PV)⁺ (**left**), somatostatin (SST)⁺ (**middle**), and c-Fos⁺ neurons (**right**) in various hippocampal layers of control, *Mdga1*-cKO and *Mdga1*$^{Y636C/E751Q}$ KI mice. Scale bar, 200 μm (applies to all images).

(**B**) Quantification of PV⁺ interneuron density in the hippocampal layer of control, *Mdga1*-cKO and *Mdga1*$^{Y636C/E751Q}$ KI mice, separated by sex (male and female), showing no differences between experimental groups. Data are presented as means ± SEMs (n = 5–6 mice/group). Abbreviations: SuB, subiculum; GCL, granular cell layer.

(**C**) Quantification of SST⁺ interneuron density in hippocampal layers of control, *Mdga1*-cKO and *Mdga1*$^{Y636C/E751Q}$ KI mice, separated by sex (male and female), showing no differences between experimental groups. Data are presented as means ± SEMs (n = 5–6 mice/group).

(**D**) Quantification of c-Fos⁺ neuron density (c-Fos/DAPI) in various hippocampal layers of male control, *Mdga1*-cKO and *Mdga1*$^{Y636C/E751Q}$ KI mice, showing no significant differences between experimental groups. Data are presented as means ± SEMs (n = 6 mice/group).

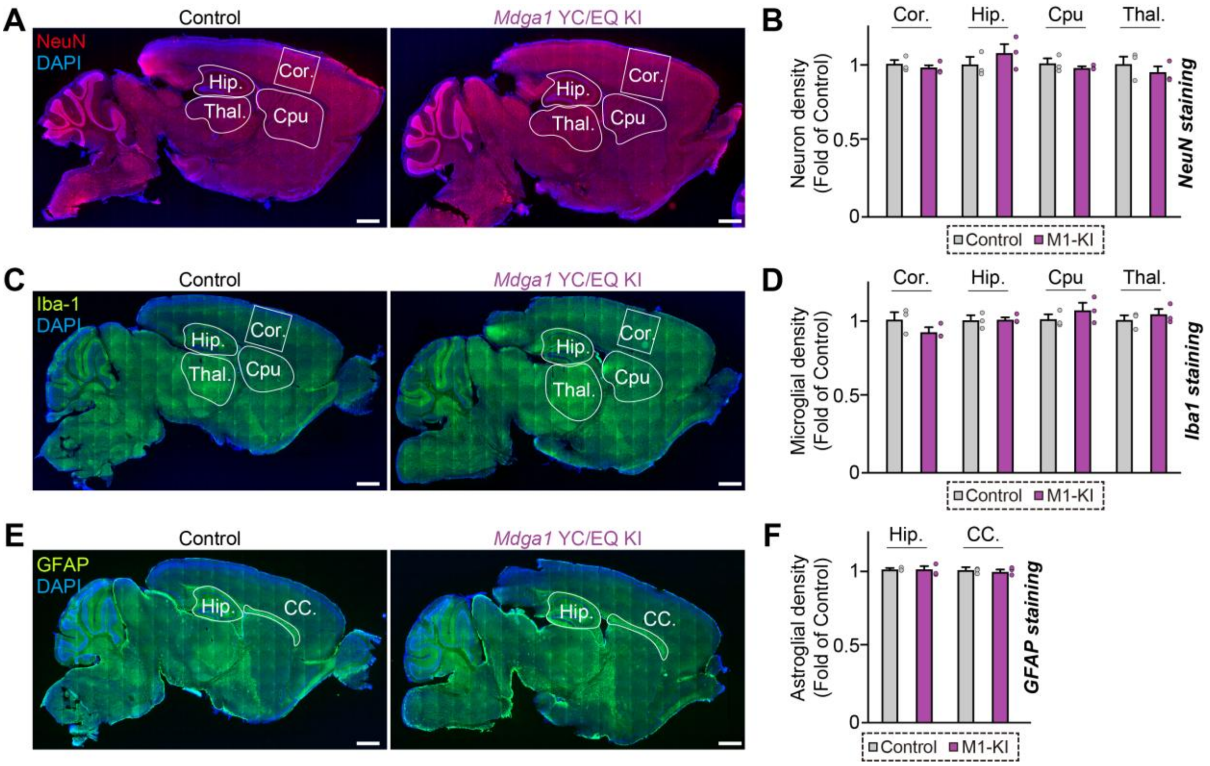

Appendix Figure S26. Analysis of densities of neurons, astrocytes and microglia across brain areas of adult male *Mdga1*$^{Y636C/E751Q}$ KI mice.

(**A**) Representative images of NeuN (a neuronal marker) staining in the adult male *Mdga1*$^{Y636C/E751Q}$ KI brain. Scale bar: 1 mm. Abbreviations: Cor., cortex; Cpu, caudate putamen; Hip., hippocampus; Thal., thalamus.

(**B**) Summary graphs for neuron density. Data are means ± SEMs ('n' denotes the number of mice; control and *Mdga1*$^{Y636C/E751Q}$ KI, n = 3; two-tailed Mann–Whitney *U* test).

(**C**) Representative images of Iba-1 (a microglia marker) staining in the adult male *Mdga1*$^{Y636C/E751Q}$ KI brain. Scale bar: 1 mm. Abbreviations: Cor., cortex; Cpu, caudate putamen; Hip., hippocampus; Thal., thalamus.

(**D**) Summary graphs for microglia density. Data are means ± SEMs ('n' denotes the number of mice; control and *Mdga1*$^{Y636C/E751Q}$ KI, n = 3; two-tailed Mann–Whitney *U* test).

(**E**) Representative images of GFAP (an astrocyte marker) staining in the adult male *Mdga1*$^{Y636C/E751Q}$ KI brain. Scale bar: 1 mm. Abbreviations: CC, corpus callosum; Hip., hippocampus.

(**F**) Summary graphs for astrocyte density. Data are means ± SEMs ('n' denotes the number of mice; control and *Mdga1*$^{Y636C/E751Q}$ KI, n = 3; two-tailed Mann–Whitney *U* test).

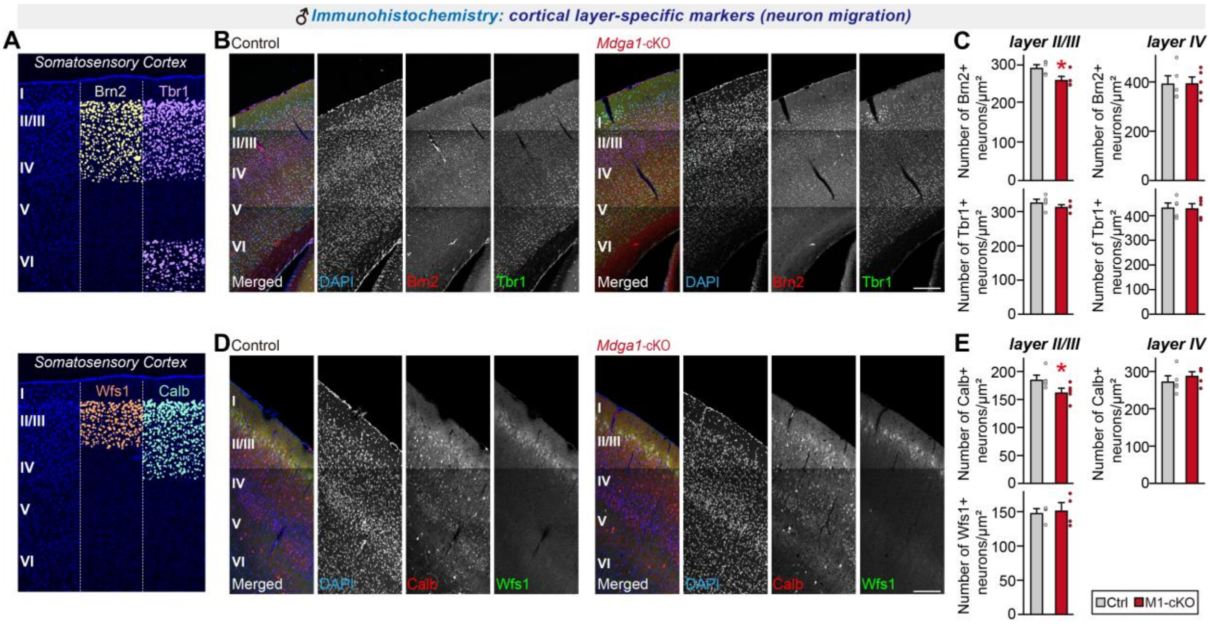

Appendix Figure S27. Analysis of cortical neuron migration for adult male *Mdga1*-cKO mice.

(**A**) Schematic illustration of cortical layer-specific markers used to assess cortical neuron distribution in the current study.

(**B**) Representative images of Brn2- and Tbr1-labeled neurons in the somatosensory cortex of adult male control and *Mdga1*-cKO mice. Neurons were immunostained with antibodies against Brn2 (red), Tbr1 (green) and DAPI (blue). Brn2 and Tbr1 are cortical layer-specific markers (Brn2 for layers II–IV; Tbr1 for layers II/III and primarily VI). Scale bar, 200 µm (applies to all images).

(**C**) Quantification of Brn2⁺ and Tbr1⁺ neuron density in cortical layers (layers II/III and IV) of adult male control and *Mdga1*-cKO mice. *Mdga1*-cKO mice exhibited a mild but significant reduction in Brn2⁺ neurons compared to controls. Data are presented as means ± SEMs (n = 5 mice/group; *$p < 0.05$; Mann–Whitney *U* test).

(**D**) Representative images of calbindin- and Wfs1-labeled neurons in the somatosensory cortex of adult male control and *Mdga1*-cKO mice. Neurons were immunostained with antibodies against calbindin (Calb; red), Wfs1 (green) and DAPI (blue). Calb and Wfs1 are cortical layer-specific markers (Calb for layers II–IV; Wfs1 for layers II/III). Scale bar, 200 µm (applies to all images).

(**E**) Quantification of calbindin⁺ and Wfs1⁺ neuron density in cortical layers II/III and IV of adult male control and *Mdga1*-cKO mice. Calb⁺ neuron density was significantly reduced in *Mdga1*-cKO mice, whereas Wfs1⁺ neurons were unaffected. Data are presented as means ± SEMs (n = 5 mice/group; *$p < 0.05$; Mann–Whitney *U* test).

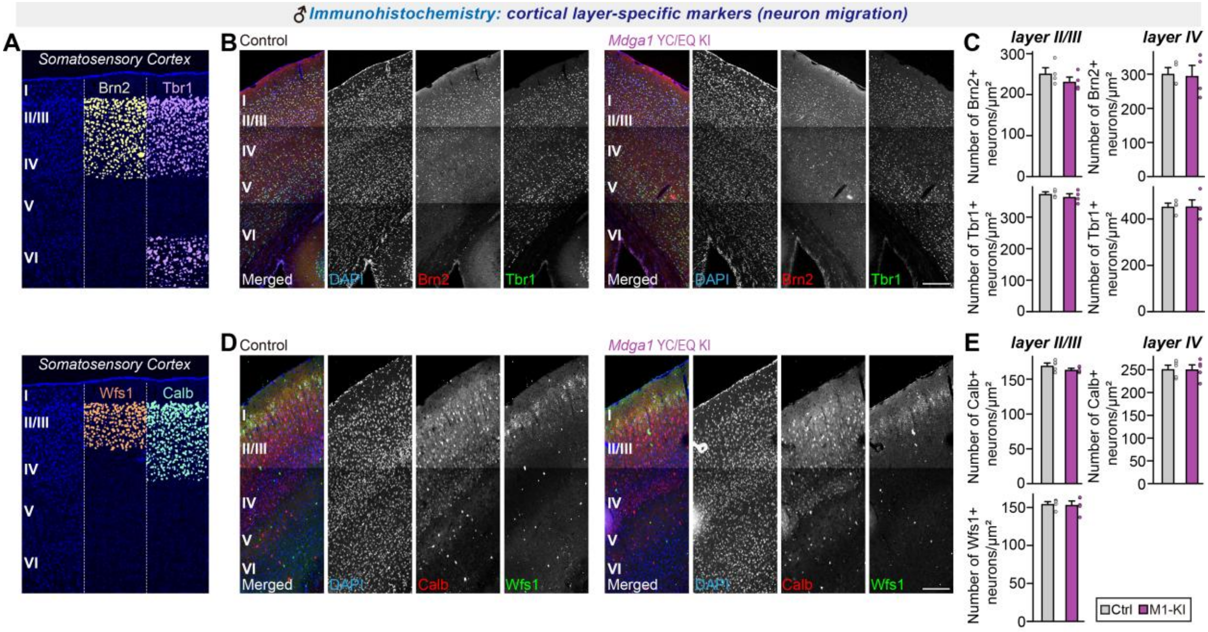

Appendix Figure S28. Analysis of cortical neuron migration for adult male *Mdga1*$^{Y636C/E751Q}$ KI mice.

(**A**) Schematic illustration of cortical layer-specific markers used to assess cortical neuron distribution in the current study.

(**B**) Representative images of Brn2- and Tbr1-labeled neurons in the somatosensory cortex of adult male control and *Mdga1*$^{Y636C/E751Q}$ KI mice. Neurons were immunostained with antibodies against Brn2 (red), Tbr1 (green) and DAPI (blue). Brn2 and Tbr1 are cortical layer-specific markers (Brn2 for layers II–IV; Tbr1 for layers II/III and primarily VI). Scale bar, 200 μm (applies to all images).

(**C**) Quantification of Brn2^{+} and Tbr1^{+} neuron density in cortical layers (layers II/III and IV) of adult male control and *Mdga1*$^{Y636C/E751Q}$ KI mice, showing no differences between genotypes. Data are presented as means ± SEMs (n = 5 mice/group; Mann–Whitney *U* test).

(**D**) Representative images of calbindin- and Wfs1-labeled neurons in the somatosensory cortex of adult male control and *Mdga1*$^{Y636C/E751Q}$ KI mice. Neurons were immunostained with antibodies against calbindin (Calb; red), Wfs1 (green) and DAPI (blue). Calb and Wfs1 are cortical layer-specific markers (Calb for layers II–IV; Wfs1 for layers II/III). Scale bar, 200 μm (applies to all images).

(**E**) Quantification of calbindin^{+} and Wfs1^{+} neuron density in cortical layers II/III and IV of adult male control and *Mdga1*$^{Y636C/E751Q}$ KI mice, showing no significant differences between genotypes. Data are presented as means ± SEMs (n = 5 mice/group; Mann–Whitney *U* test).

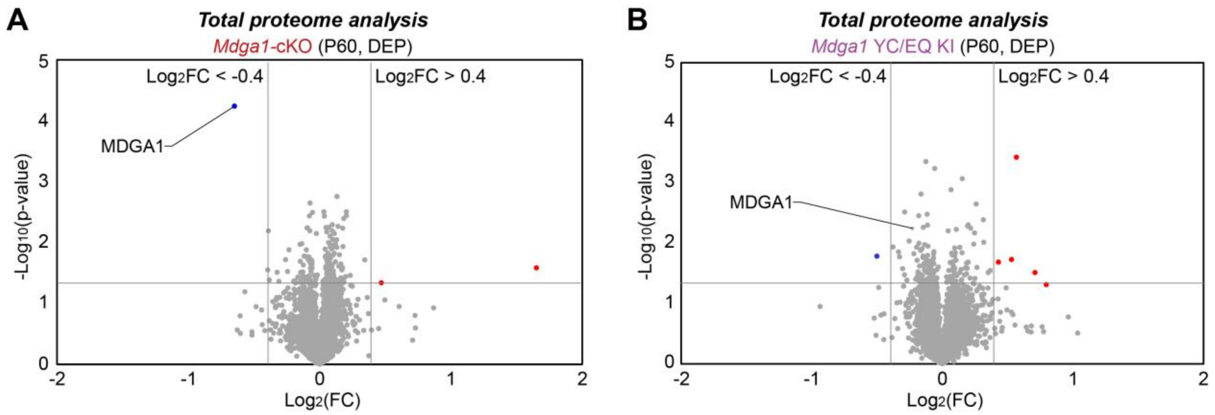

Appendix Figure S29. Volcano plot of proteins identified from proteomic analyses of hippocampi from adult *Mdga1*-cKO and *Mdga1*$^{Y636C/E751Q}$ KI mice.

(**A**) Volcano plot displaying differentially expressed proteins (DEPs) identified from hippocampi of adult male *Mdga1*-cKO mice compared to controls. Only a limited number of proteins exhibited significant differential expression (Log_2FC) < -0.4 or > 0.4, **p* < 0.05), with downregulated DEPs shown in blue and upregulated DEPs in red. MDGA1 is highlighted among the few significantly downregulated proteins.

(**B**) Same as **A**, except showing results obtained from adult male *Mdga1*$^{Y636C/E751Q}$ KI mice compared to controls. In contrast to *Mdga1*-cKO mice, *Mdga1*$^{Y636C/E751Q}$ KI mice exhibited a broader distribution of DEPs, suggesting that they experienced more pronounced proteomic impacts. Each dot represents an individual protein, with significance represented by $-Log_{10}$(*p*-value) on the y-axis and Log_2FC on the x-axis.

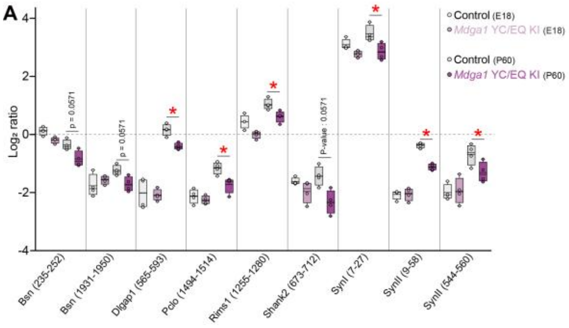

B

Protein name	Sequence
Bsn	(235-252) SQQQLHSPALSPAHSPAK; 2 phosphorylation
Bsn	(1931-1950) SVTDTALPGQSSGPFYSPR; 1 phosphorylation
Dlgap1	(565-593) TTTKPFISITAQSSTESAQDAYMDGQGQR; 1 phosphorylation
Pclo	(1494-1514) QIIEMSADEDASGSEDEEFIR; 1 phosphorylation
Rims1	(1255-1280) QPSRESTDGSINSYSSEGNLIFPGVR; 1 phosphorylation
Shank2	(673-712) SSQGSSMEIDPQATEPGQLRPDDSLTVSSPFAAAIAGAVR; 1 phosphorylation
SynI	(7-27) RLSDSNFMANLPNGYMDLQR; 1 phosphorylation
SynII	(9-58) LSDSSFIANLPNGYMTDLQRPEPQQPPPAPGPGAATASAATSAASPGPER; 1 phosphorylation
SynII	(544-560) SQSLTNAFSFSESSFFR; 1 phosphorylation

Appendix Figure S30. Analysis of phosphorylation sites that are altered in adult male *Mdga1*$^{Y636C/E751Q}$ KI mice.

(**A**) Quantitative comparison of Log_2-transformed phosphopeptide intensities for key synaptic proteins, including synapsin I (SynI), synapsin II (SynII), Piccolo (Pclo), Bassoon (Bsn), Shank2, RIMS1, and Dlgap1, in hippocampal lysates from embryonic (E18) and adult (P60) *Mdga1*$^{Y636C/E751Q}$ KI mice relative to control littermates. Notably, the phosphorylation levels of several presynaptic scaffolding proteins were significantly altered in a stage-specific manner. Data are presented as means ± SEMs (n = 4 mice/group; *$p < 0.05$; two-way ANOVA followed by Tukey's *post hoc* test).

(**B**) Table summarizing the phosphopeptides corresponding to each synaptic protein listed in panel **(A)**, including peptide sequence, phosphorylated residues, and associated fold-change values in the E18 and P60 samples.

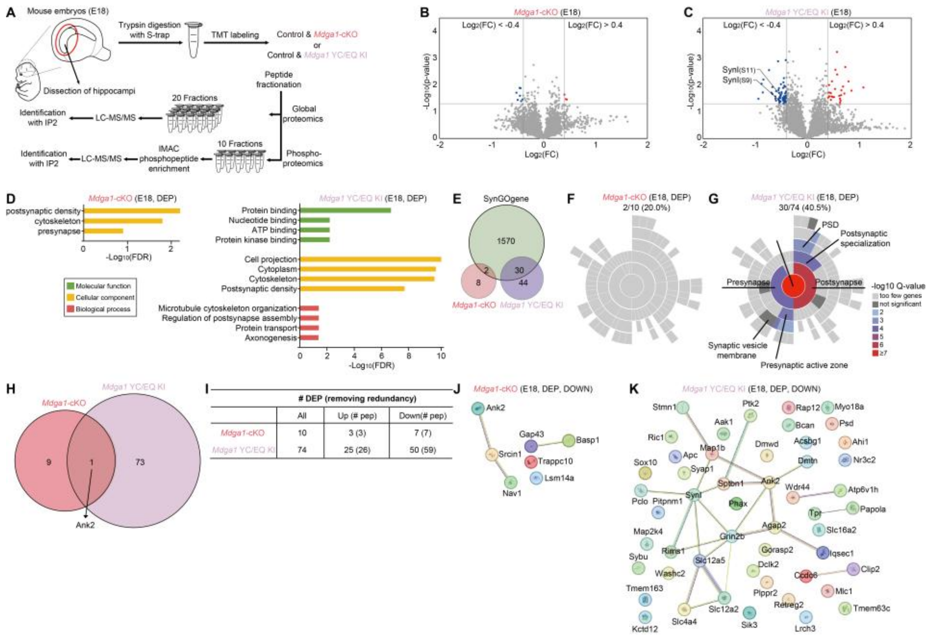

Appendix Figure S31. Phosphoproteomic analysis of hippocampi from embryos of *Mdga1*-cKO and *Mdga1*$^{Y636C/E751Q}$ KI mice.

(**A**) Schematic diagram of the paradigm for the phosphoproteomic analysis of hippocampal lysates from embryos of *Mdga1*-cKO, *Mdga1*$^{Y636C/E751Q}$ KI, and littermate control mice. A total of four biological replicates were used per group (n = 4/group).

(**B**) Volcano plot of 4,338 phosphopeptides identified from hippocampal lysates of *Mdga1*-cKO embryos. Differentially expressed phosphopeptides (DEPPs) with significant increases or decreases ($Log_2FC < -0.4$ or > 0.4; $p < 0.05$; Welch's T-test) are shown in red (3 phosphopeptides) and blue (7 phosphopeptides), respectively.

(**C**) Volcano plot of 5,124 phosphopeptides identified from hippocampal lysates of *Mdga1*$^{Y636C/E751Q}$ KI embryos. DEPPs with significant increases or decreases ($Log_2FC < -0.4$ or > 0.4; $p < 0.05$; Welch's T-test) are shown in red (26 phosphopeptides) and blue (59 phosphopeptides), respectively.

(**D**) Gene ontology (GO) analysis was performed on differentially expressed phosphoproteins (DEPs) identified from the phosphoproteomic analyses of *Mdga1*-cKO (**left**) and *Mdga1*$^{Y636C/E751Q}$ KI (**right**) embryos. The bar charts depict the significantly enriched GO terms in the Molecular Function (green), Cellular Component (yellow), and Biological Process (red) categories, represented as $-log_{10}$(FDR) values.

(**E**) Ven diagram visualization of SynGO category proteins.

(**F**) Localization annotations of SynGO category proteins from *Mdga1*-cKO embryos.

(**G**) Localization annotations of SynGO category proteins from *Mdga1*$^{Y636C/E751Q}$ KI embryos.

(**H**) Venn diagram showing the overlap of DEPs between *Mdga1*-cKO and *Mdga1*$^{Y636C/E751Q}$ KI embryos. Ank2 is shared between the conditions; 9 DEPs are unique to *Mdga1*-cKO embryos and 73 are unique to *Mdga1*$^{Y636C/E751Q}$ KI embryos.

(**I**) Summary table of DEPs after removal of redundancy. The table shows the total number DEPs with a breakdown of the upregulated and downregulated proteins in *Mdga1*-cKO and *Mdga1*$^{Y636C/E751Q}$ KI embryos, together with the number of unique peptides (# pep).

(**J**) STRING analysis of downregulated DEPs from *Mdga1*-cKO embryos ($p < 0.05$ and $Log_2FC < -0.4$; Welch's T-test).

(**K**) STRING analysis of downregulated DEPs from *Mdga1*$^{Y636C/E751Q}$ KI embryos ($p < 0.05$ and $Log_2FC <$ -0.4).

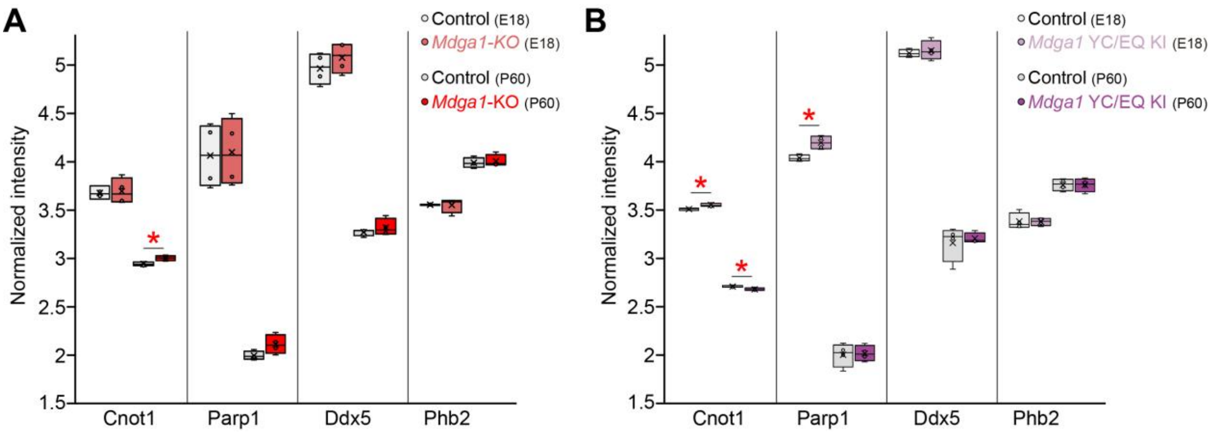

Appendix Figure S32. Estrogen receptor-associated protein abundance in hippocampi from embryonic and adult male *Mdga1*-cKO and *Mdga1*$^{Y636C/E751Q}$ KI mice.

(**A**) Quantitative comparison of $\log_2$-transformed protein intensities for Cnot1, Parp1, Ddx5, and Phb2 in hippocampal lysates from embryonic (E18) and adult (P60) male *Mdga1*-KO mice, relative to control littermates. Proteomic values were normalized using the column-median method. Data are presented as box plots (n = 4 mice/group; **p* < 0.05; two-tailed Student's t-test).

(**B**) Quantitative comparison of $\log_2$-transformed protein intensities for the same four candidates in hippocampal lysates from E18 and P60 male *Mdga1*$^{Y636C/E751Q}$ KI mice, relative to control littermates. Proteomic values were normalized using the column-median method. Data are presented as box plots (n = 4 mice/group; **p* < 0.05; two-tailed Student's t-test).

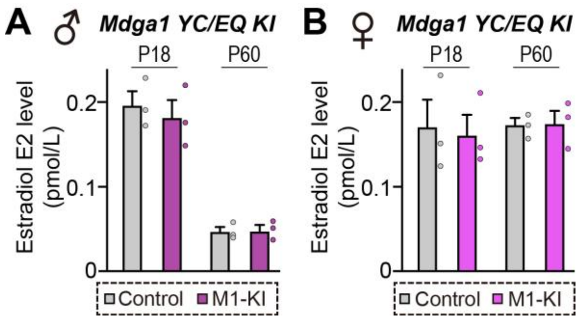

Appendix Figure S33. Measurement of estradiol e2 levels from juvenile and adult *Mdga1*$^{Y636C/E751Q}$ KI mice.

(**A** and **B**) Quantification of estradiol e2 levels of control and *Mdga1*$^{Y636C/E751Q}$ KI male (**A**) or female (**B**) mice at P18 and P60. Data are means ± SEMs ('n' denotes the number of mice; control and *Mdga1*$^{Y636C/E751Q}$ KI, n = 3).

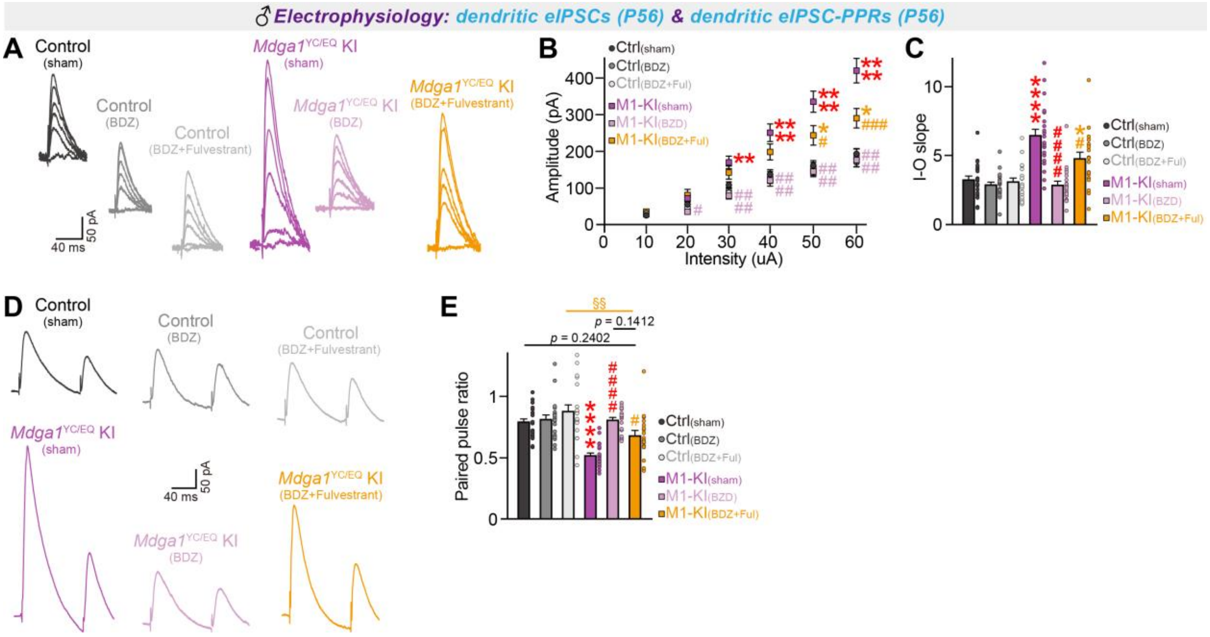

Appendix Figure S34. Effect of Fulvestrant administration into adult male *Mdga1*$^{Y636C/E751Q}$ KI mice on evoked GABAergic synaptic strength and GABA release probability.
Representative traces (**A** and **D**) and quantification of dendritic eIPSCs in the hippocampal CA1 pyramidal neurons from adult control and *Mdga1*$^{Y636C/E751Q}$ KI mice (P56). Input-output (I-O) curves (**B** and **C**) and PPRs (**E**) of dendritic eIPSCs. Data are presented as means ± SEMs (n = 18–23 cells/group; $^{*}p < 0.05$, $^{**}p < 0.01$, $^{****}p < 0.0001$, $^{\#}p < 0.05$, $^{\#\#}p < 0.01$, $^{\#\#\#}p < 0.001$, $^{\#\#\#\#}p < 0.0001$; $^{\S\S}p < 0.01$; # and § indicates statistical comparisons with their counterparts; two-way ANOVA followed by Tukey's *post hoc* test).

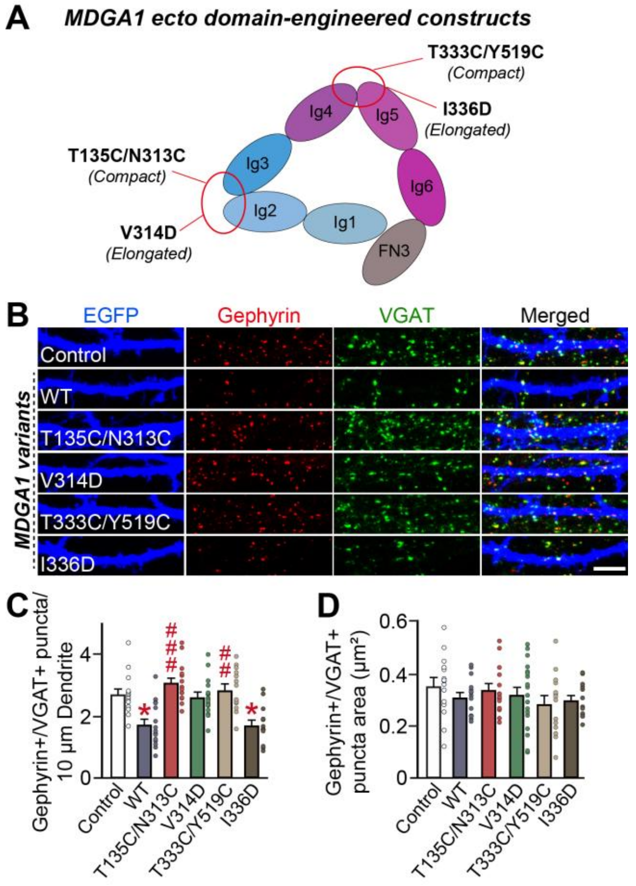

Appendix Figure S35. Effect of conformational changes in MDGA1 extracellular regions on GABAergic synapses in cultured hippocampal neurons.

(**A**) Schematic depicting MDGA1 designer mutants that enable more compact and extended three-dimensional conformations of MDGA1 extracellular domains. Abbreviations: com., compact; ext., extended.

(**B**) Representative confocal images of hippocampal neurons transfected at DIV7 with EGFP (green) and either MDGA1 WT or the indicated MDGA1 variants (T135C/N313C, V314D, T333C/Y519C and I336D). Neurons were immunostained at DIV14 for VGAT (magenta) and gephyrin (red). EGFP (green) marks the transfected neurons. Scale bar, 10 μm.

(**C**) Quantification of gephyrin$^+$/VGAT$^+$ puncta density per 10-μm dendritic segment in hippocampal neurons expressing control, MDGA1 WT or the indicated MDGA1 variant. Data are presented as the number of gephyrin$^+$/VGAT$^+$ puncta per 10 μm of dendrite. While MDGA1 WT reduced the density of inhibitory synaptic puncta compared to control levels, T135C/N313C and T333C/Y519C—but not V314D or I336D—failed to reduce inhibitory synapse density as effectively as WT, suggesting that these variants adopting a more compact MDGA1 conformation exhibited a loss of GABAergic synapse-suppressing activity. Data are presented as means ± SEMs (n = 13–18 neurons/group; $^{**}p < 0.01$ (compared to control), $^{\#\#}p < 0.01$, $^{\#\#\#}p < 0.001$ (compared to MDGA1 WT); nonparametric Kruskal-Wallis test with Dunn's *post hoc* test).

(**D**) Quantification of gephyrin$^+$/VGAT$^+$ puncta area in hippocampal neurons expressing control, MDGA1 WT or the indicated MDGA1 variant. No significant differences were observed among groups. Data are presented as means ± SEMs (n = 13–18 neurons/group).
