# Supplementary figures and images for: Bazedoxifene reverses sexually dimorphic autistic-like abnormalities in biallelic MDGA1-mutant mice (part 1 of 2)
Source: EMBO Mol Med. 2026 Mar 20;18(4):1358–98. doi: 10.1038/s44321-026-00402-y (PMC13084050; doi:10.1038/s44321-026-00402-y)

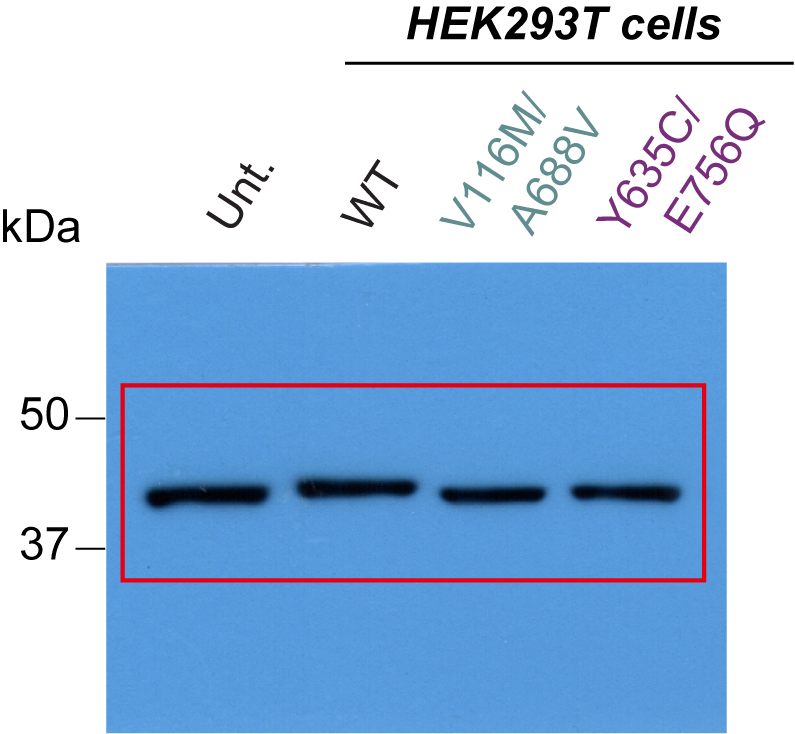

Supplement: Supplementary file 15 — Source data Fig. 1 [file 44321_2026_402_MOESM15_ESM.zip › Panel G/Western Actin.tif]

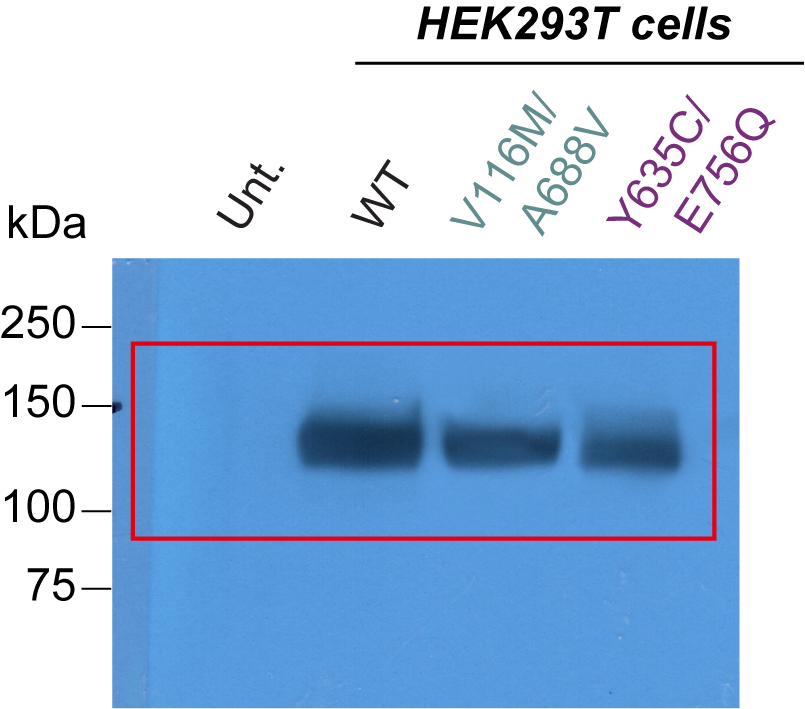

Supplement: Supplementary file 15 — Source data Fig. 1 [file 44321_2026_402_MOESM15_ESM.zip › Panel G/Western HA.tif]

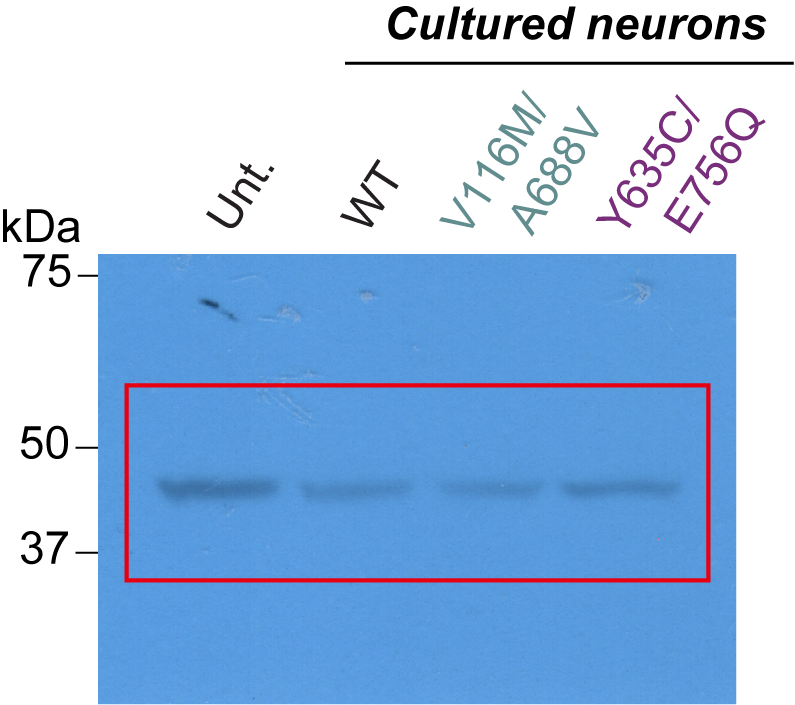

Supplement: Supplementary file 15 — Source data Fig. 1 [file 44321_2026_402_MOESM15_ESM.zip › Panel H/Western Actin.tif]

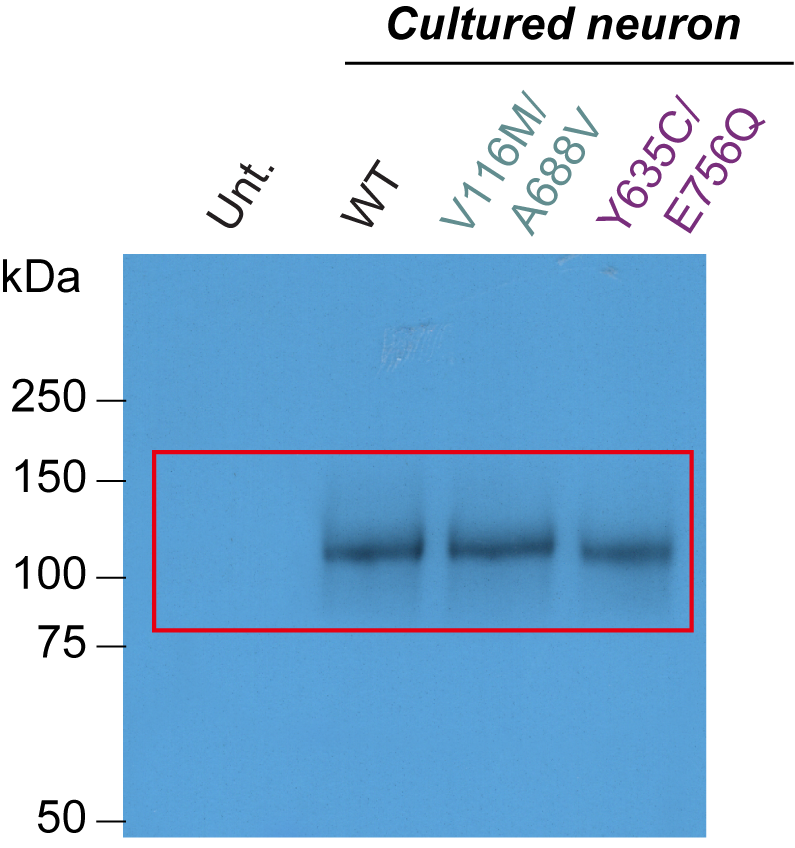

Supplement: Supplementary file 15 — Source data Fig. 1 [file 44321_2026_402_MOESM15_ESM.zip › Panel H/Western HA.tif]

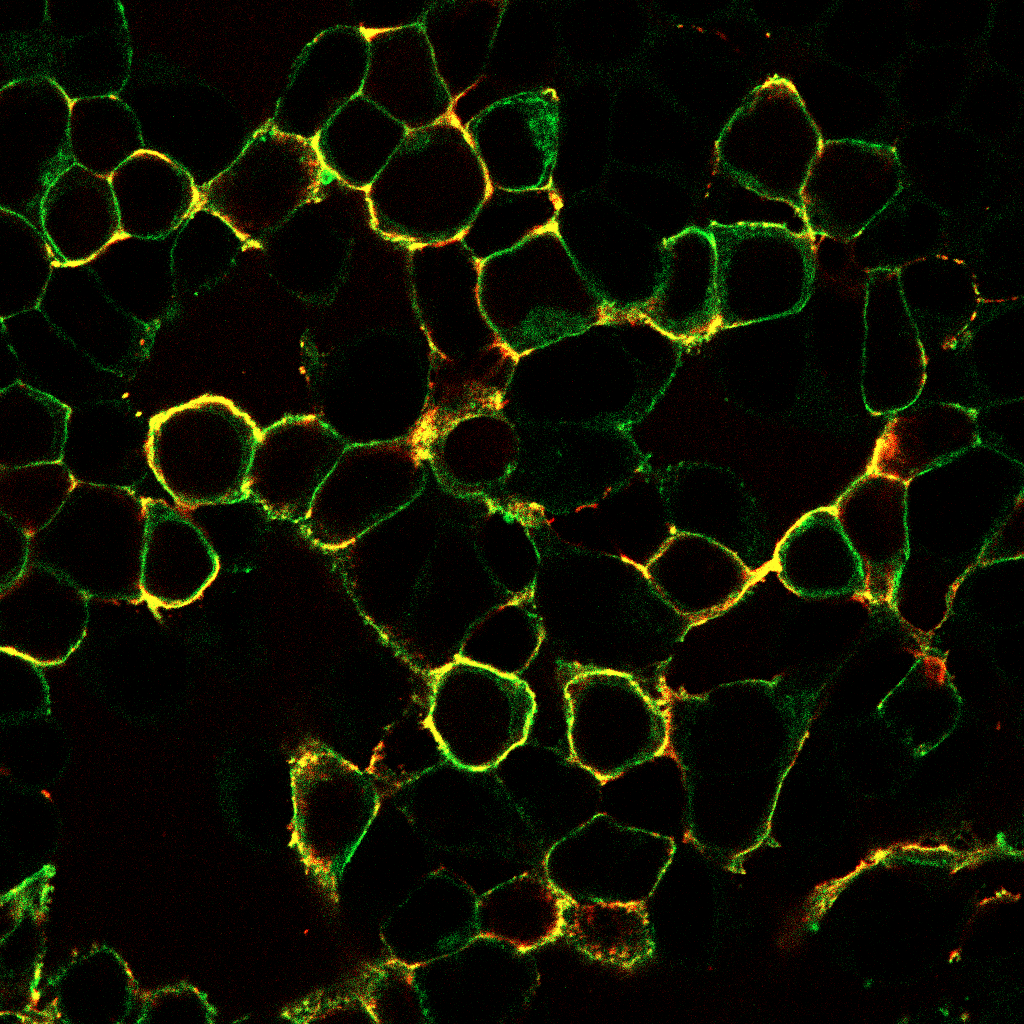

Supplement: Supplementary file 15 — Source data Fig. 1 [file 44321_2026_402_MOESM15_ESM.zip › Panel I and J/MDGA1 V116M, A688V/MDGA1 V116M, A688V (1).tif]

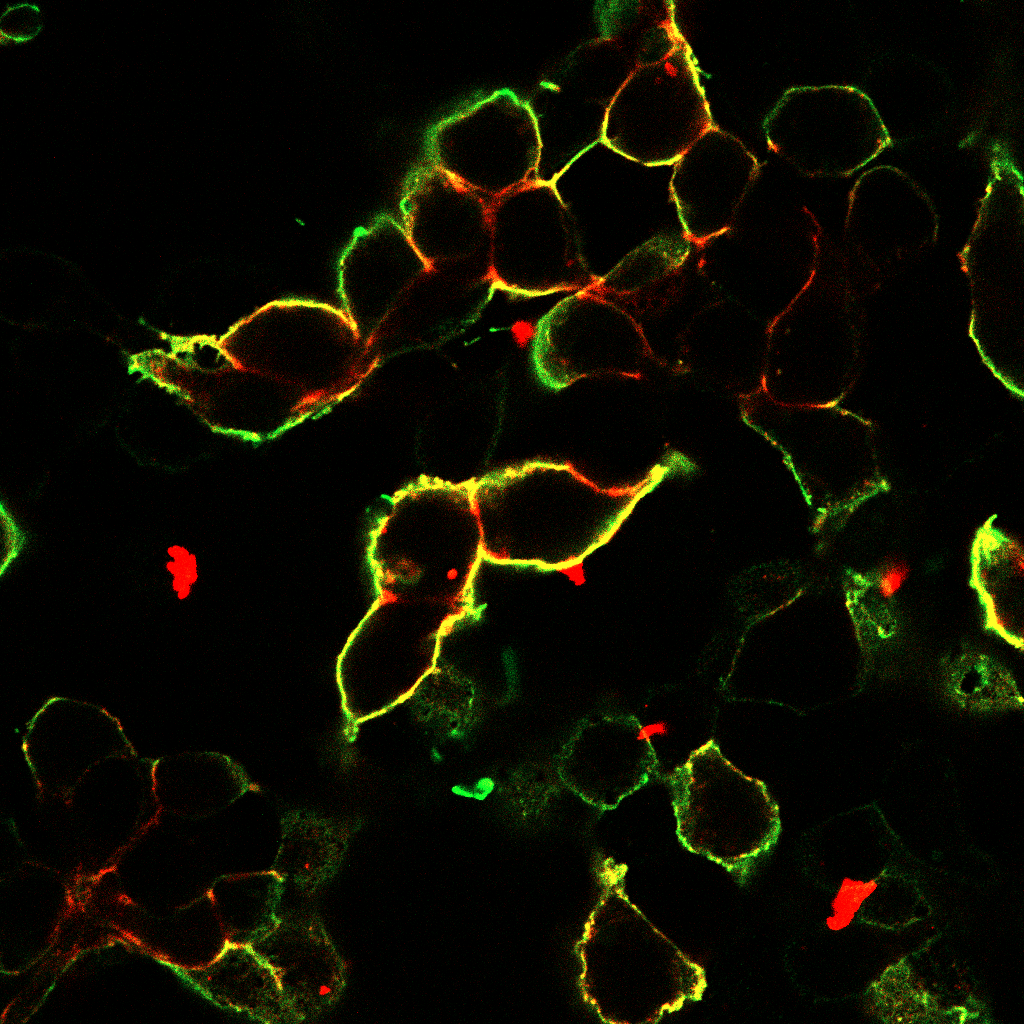

Supplement: Supplementary file 15 — Source data Fig. 1 [file 44321_2026_402_MOESM15_ESM.zip › Panel I and J/MDGA1 V116M, A688V/MDGA1 V116M, A688V (2).tif]

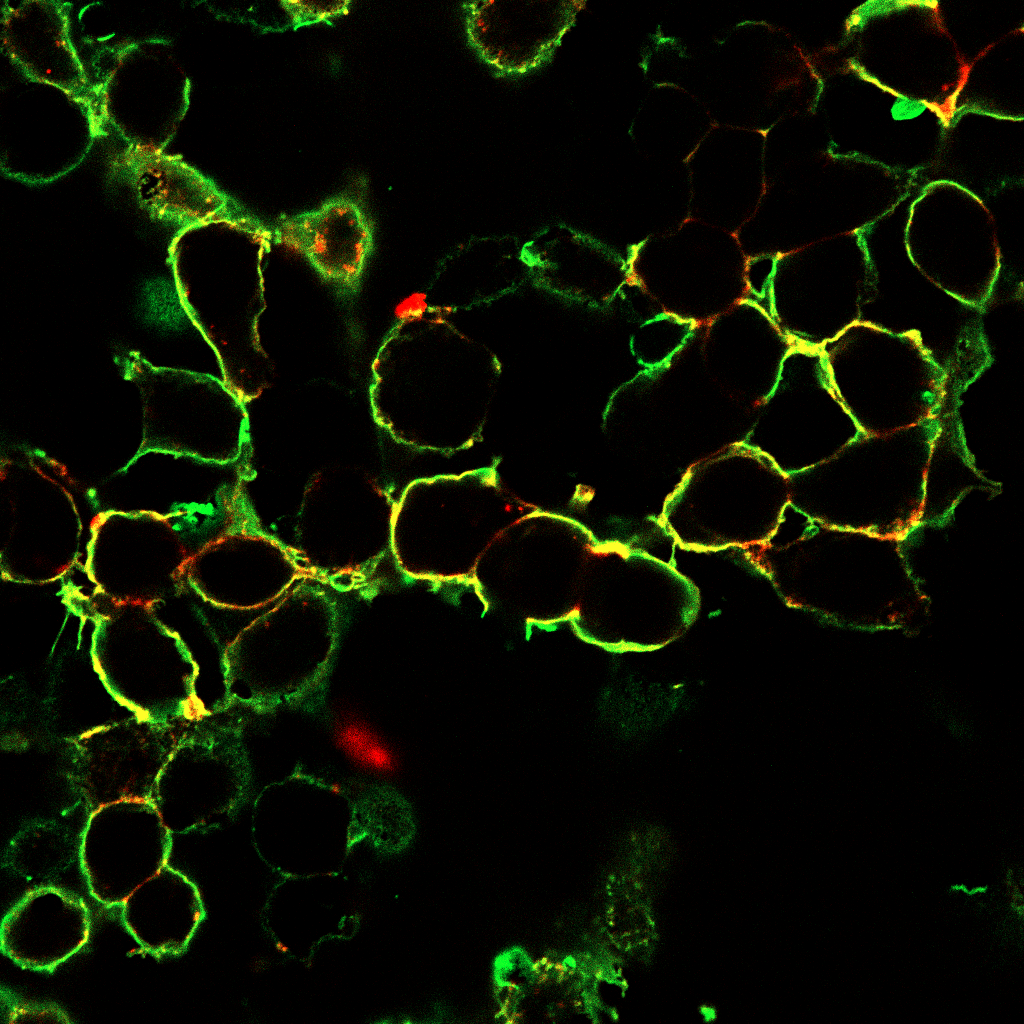

Supplement: Supplementary file 15 — Source data Fig. 1 [file 44321_2026_402_MOESM15_ESM.zip › Panel I and J/MDGA1 V116M, A688V/MDGA1 V116M, A688V (3).tif]

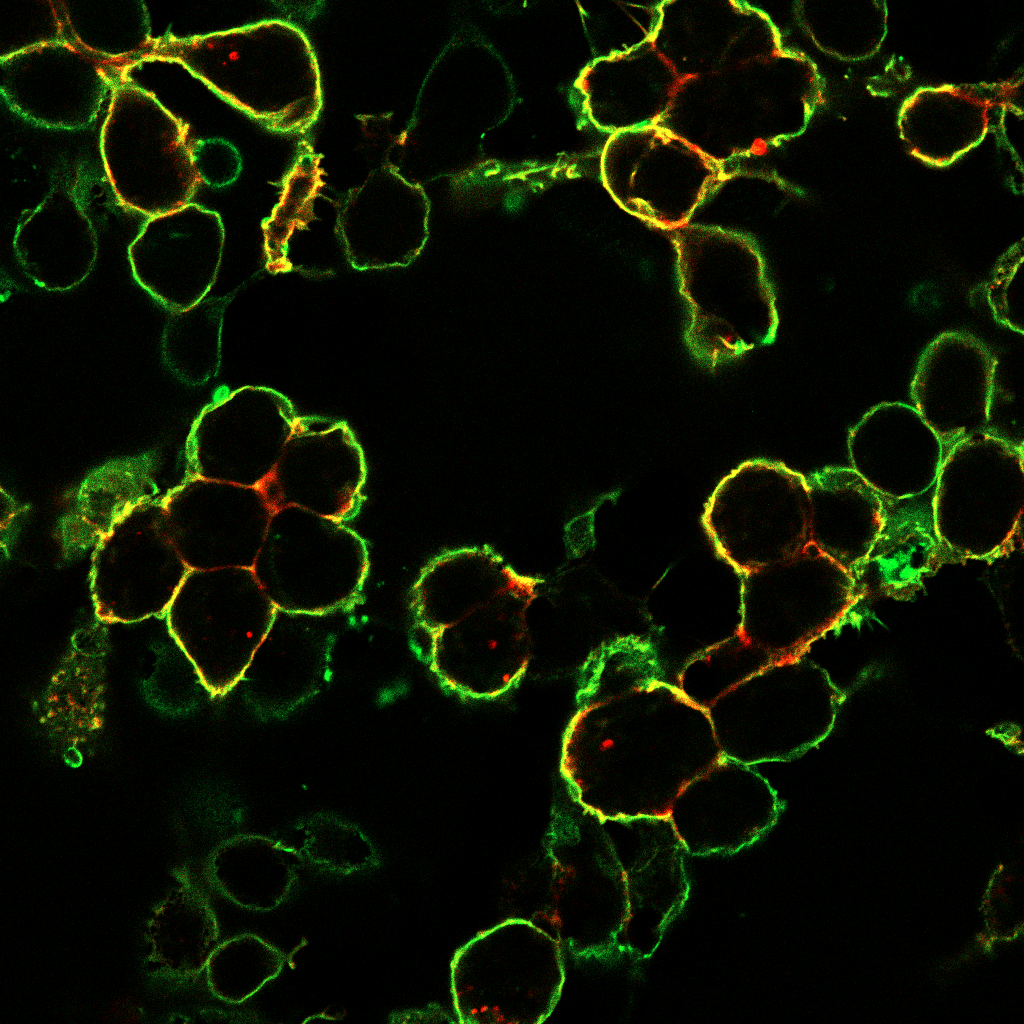

Supplement: Supplementary file 15 — Source data Fig. 1 [file 44321_2026_402_MOESM15_ESM.zip › Panel I and J/MDGA1 V116M, A688V/MDGA1 V116M, A688V (4).tif]

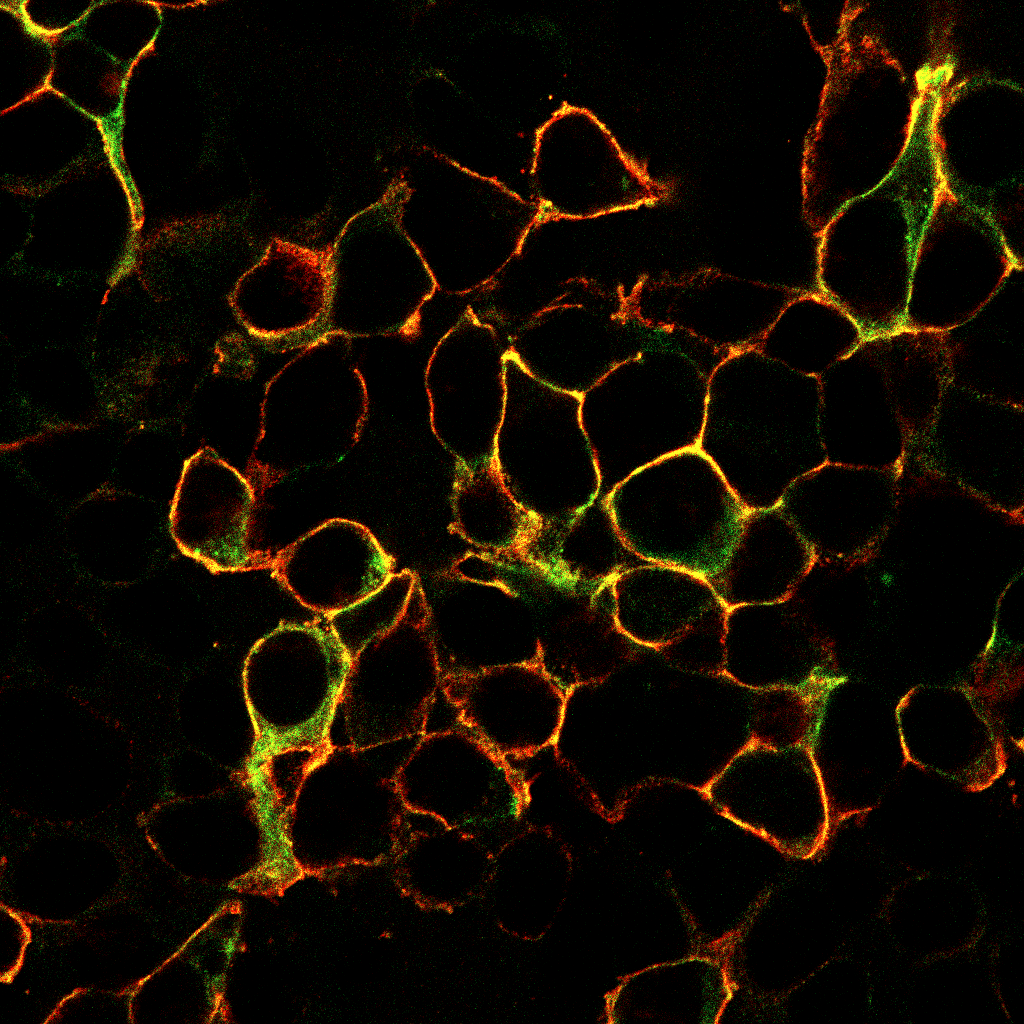

Supplement: Supplementary file 15 — Source data Fig. 1 [file 44321_2026_402_MOESM15_ESM.zip › Panel I and J/MDGA1 V116M, A688V/MDGA1 V116M, A688V (5).tif]

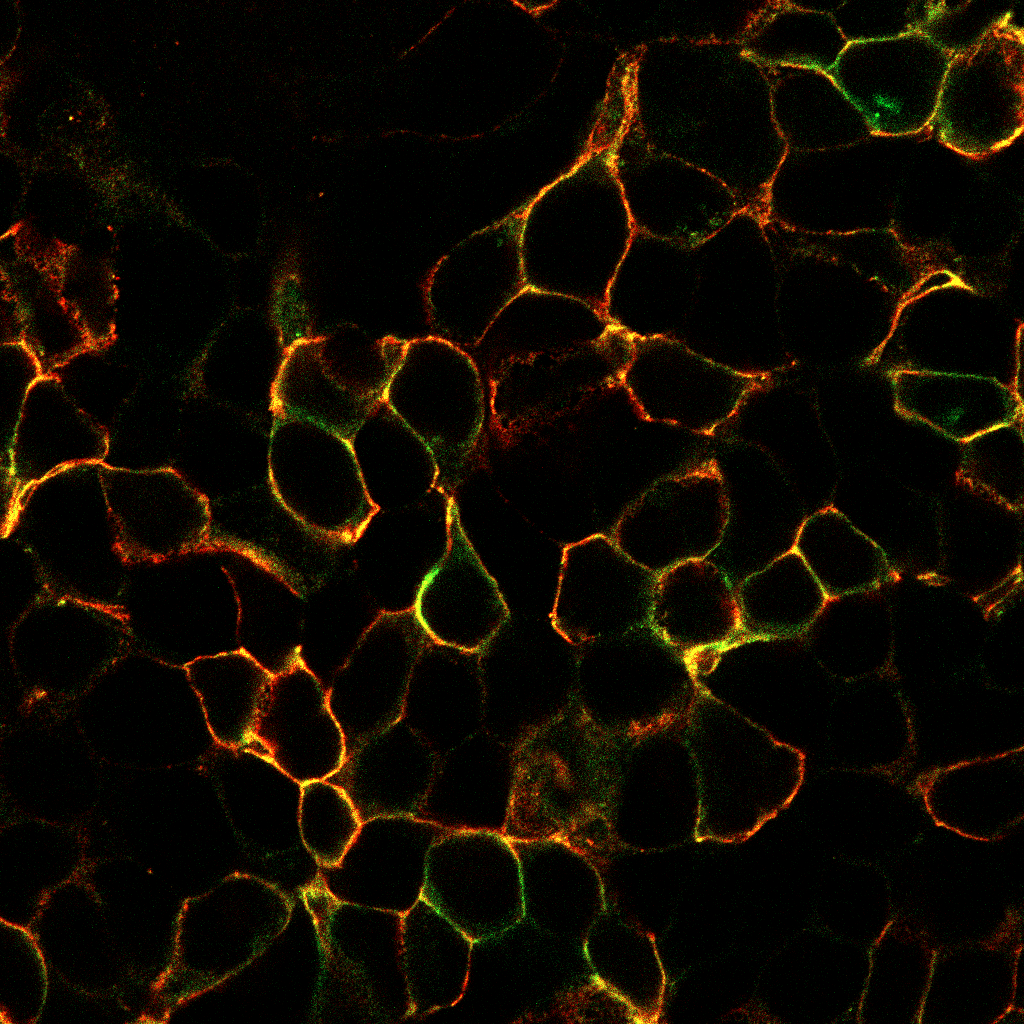

Supplement: Supplementary file 15 — Source data Fig. 1 [file 44321_2026_402_MOESM15_ESM.zip › Panel I and J/MDGA1 V116M, A688V/MDGA1 V116M, A688V (6).tif]

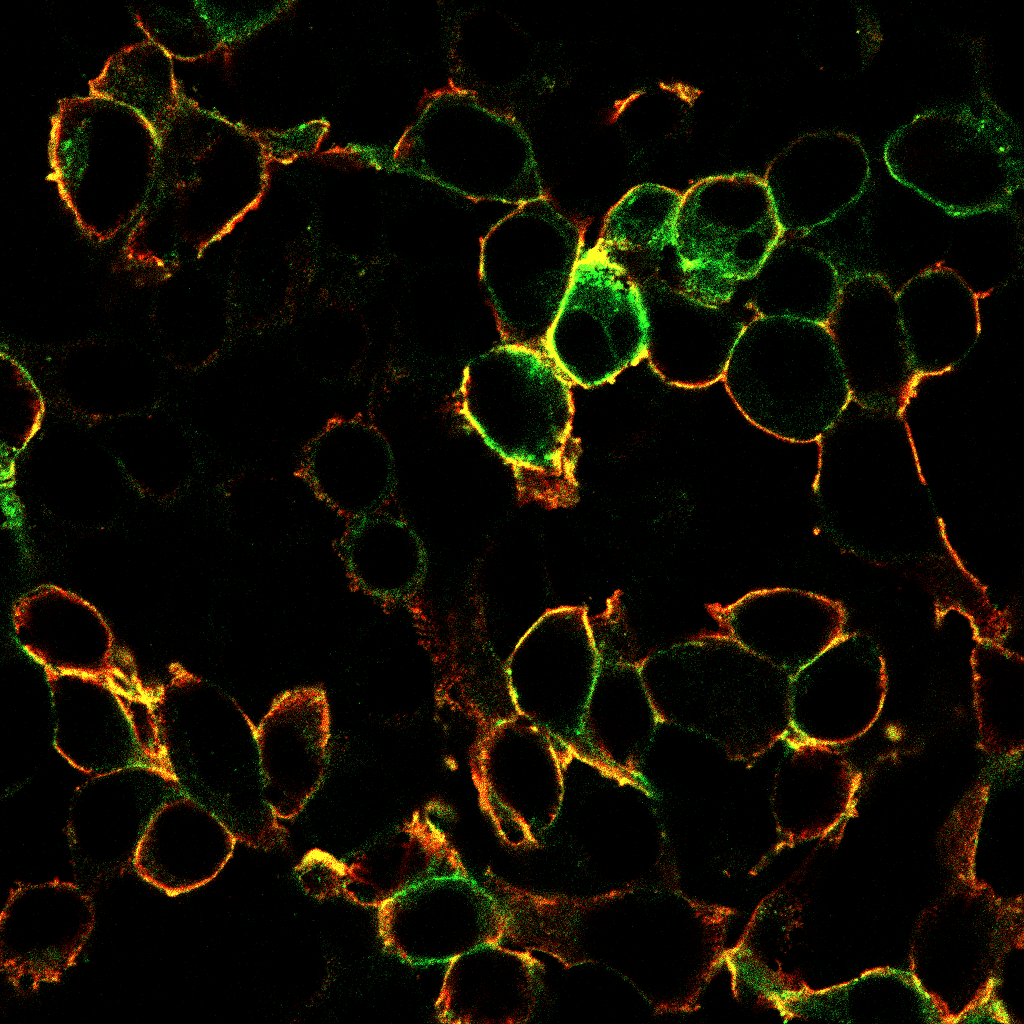

Supplement: Supplementary file 15 — Source data Fig. 1 [file 44321_2026_402_MOESM15_ESM.zip › Panel I and J/MDGA1 V116M, A688V/MDGA1 V116M, A688V (7).tif]

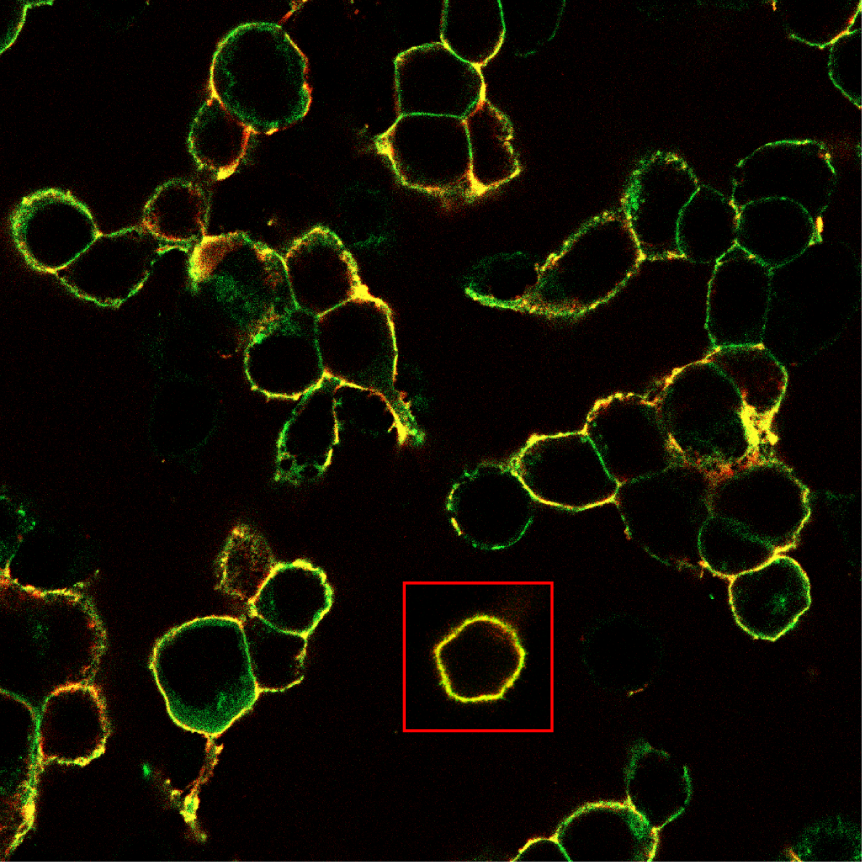

Supplement: Supplementary file 15 — Source data Fig. 1 [file 44321_2026_402_MOESM15_ESM.zip › Panel I and J/MDGA1 V116M, A688V.tif]

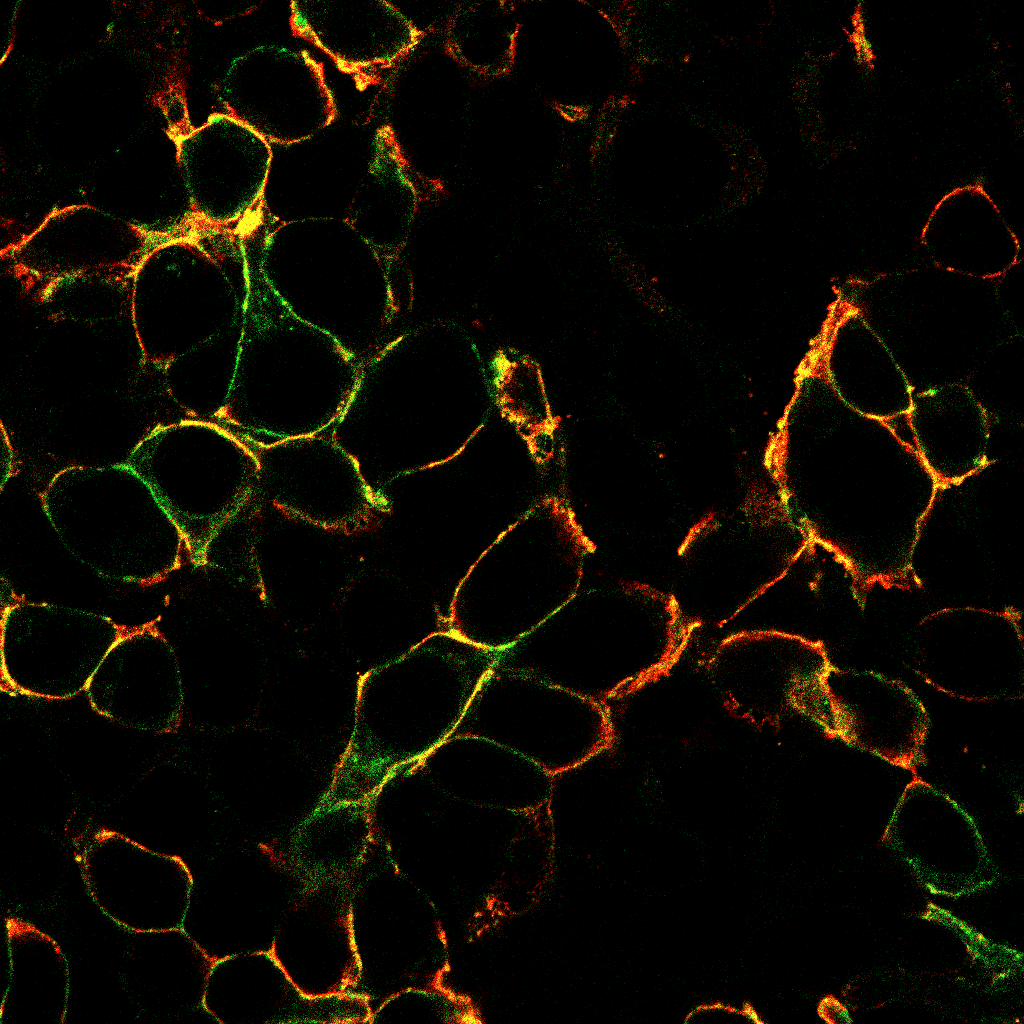

Supplement: Supplementary file 15 — Source data Fig. 1 [file 44321_2026_402_MOESM15_ESM.zip › Panel I and J/MDGA1 WT/MDGA1 WT (1).tif]

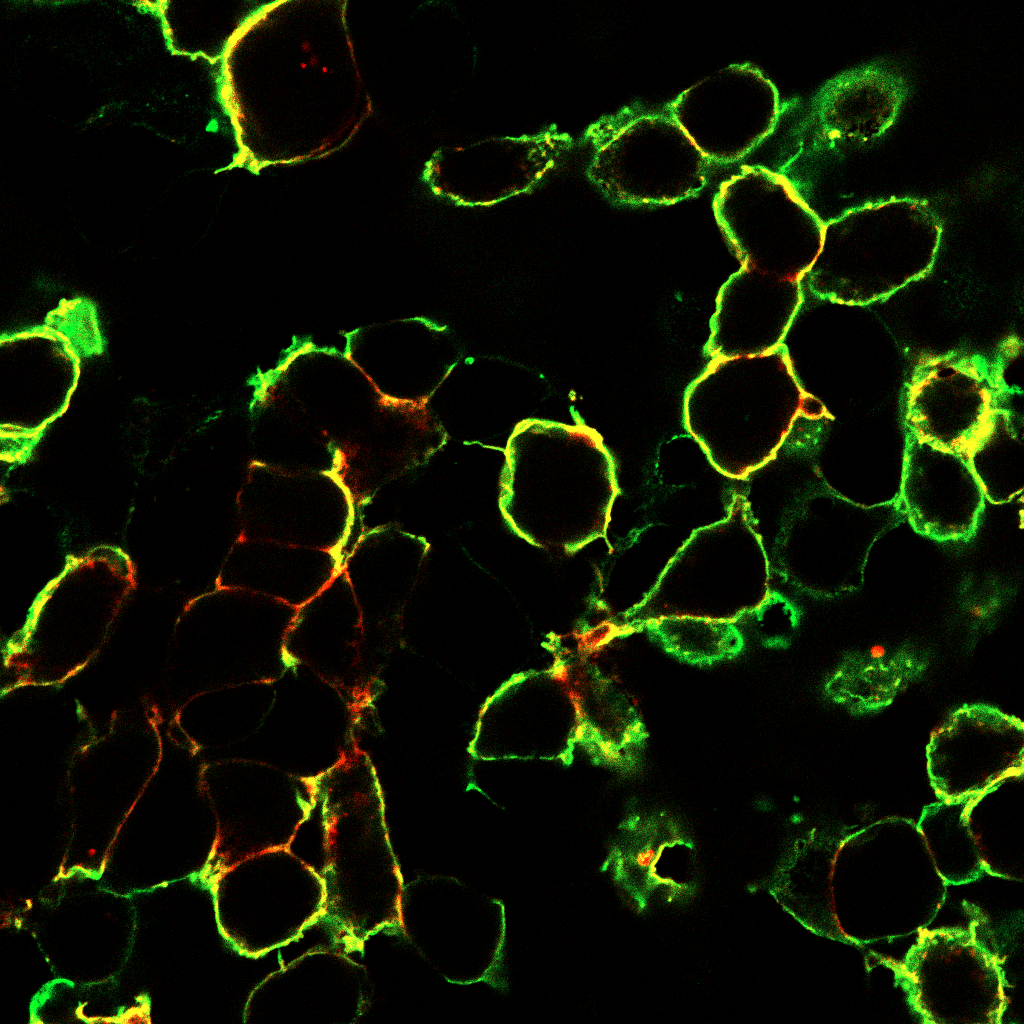

Supplement: Supplementary file 15 — Source data Fig. 1 [file 44321_2026_402_MOESM15_ESM.zip › Panel I and J/MDGA1 WT/MDGA1 WT (2).tif]

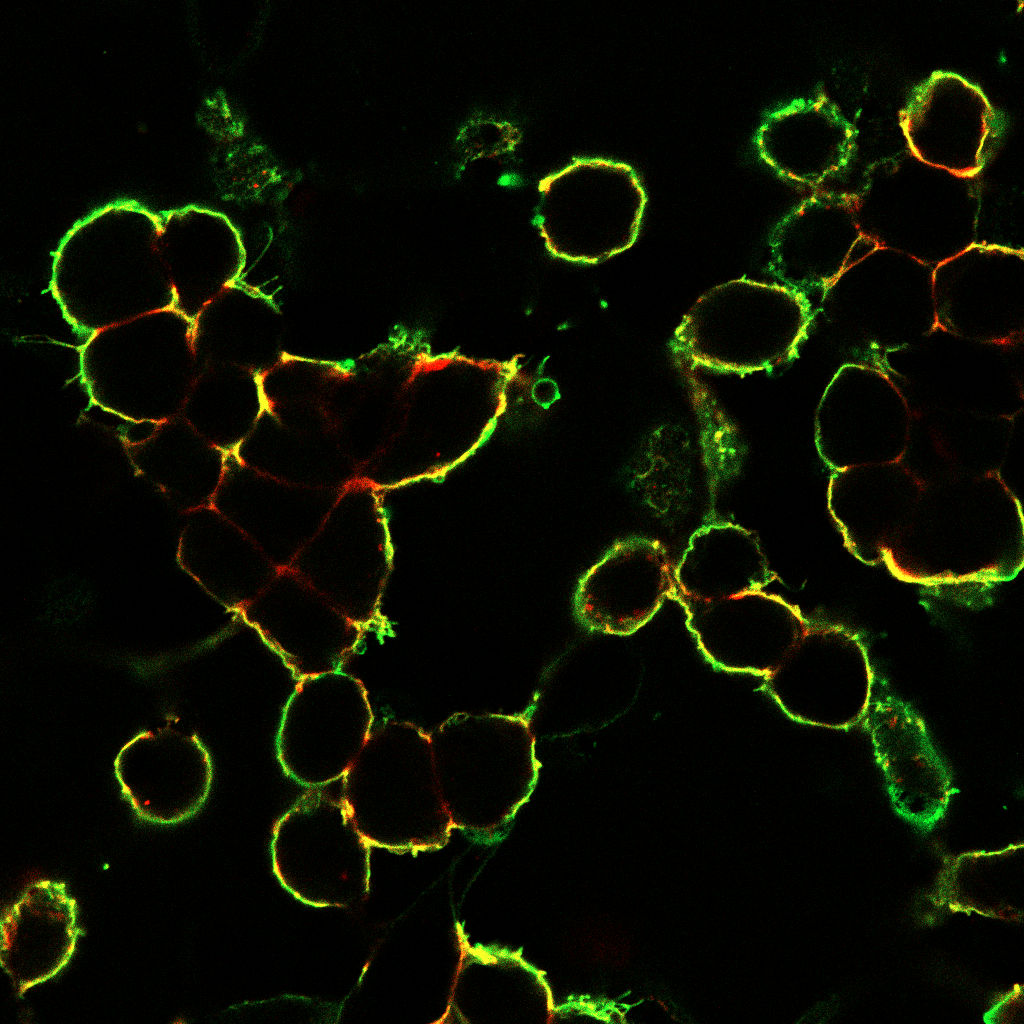

Supplement: Supplementary file 15 — Source data Fig. 1 [file 44321_2026_402_MOESM15_ESM.zip › Panel I and J/MDGA1 WT/MDGA1 WT (3).tif]

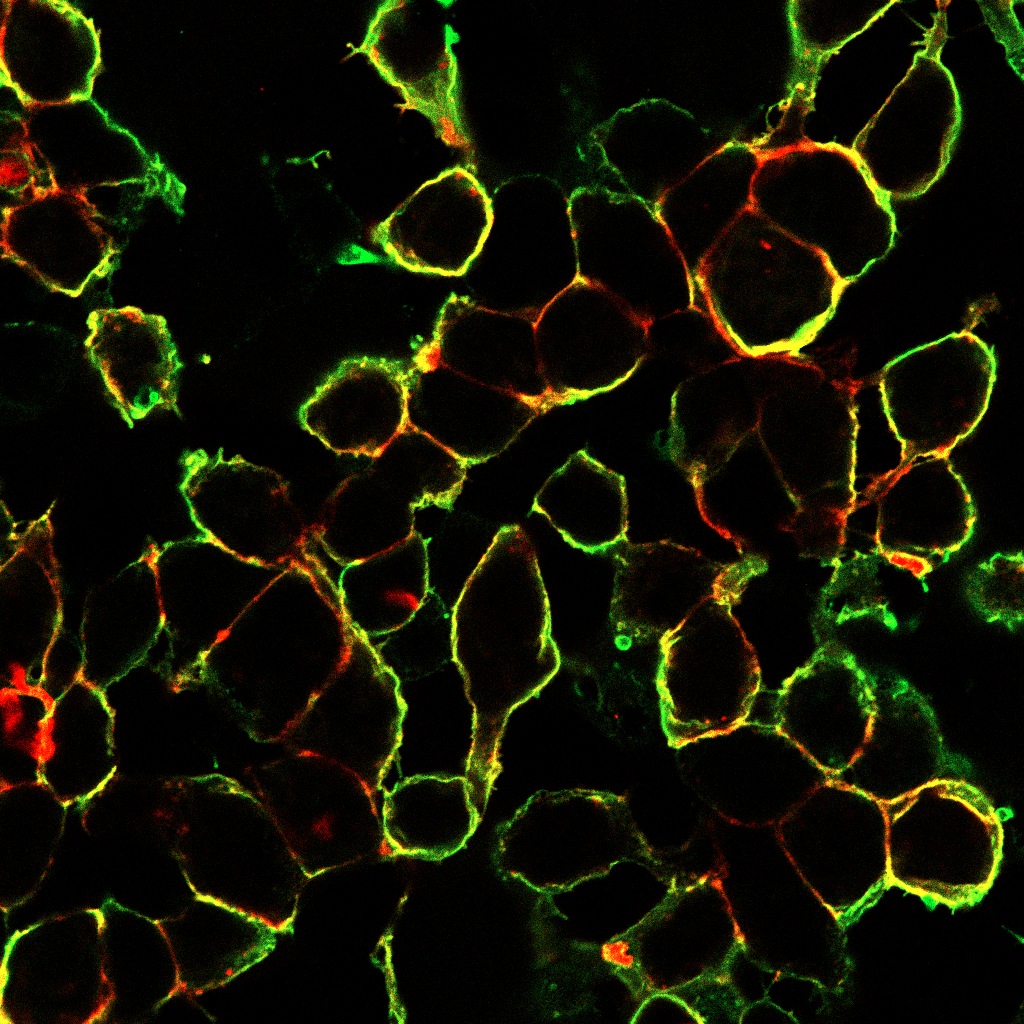

Supplement: Supplementary file 15 — Source data Fig. 1 [file 44321_2026_402_MOESM15_ESM.zip › Panel I and J/MDGA1 WT/MDGA1 WT (4).tif]

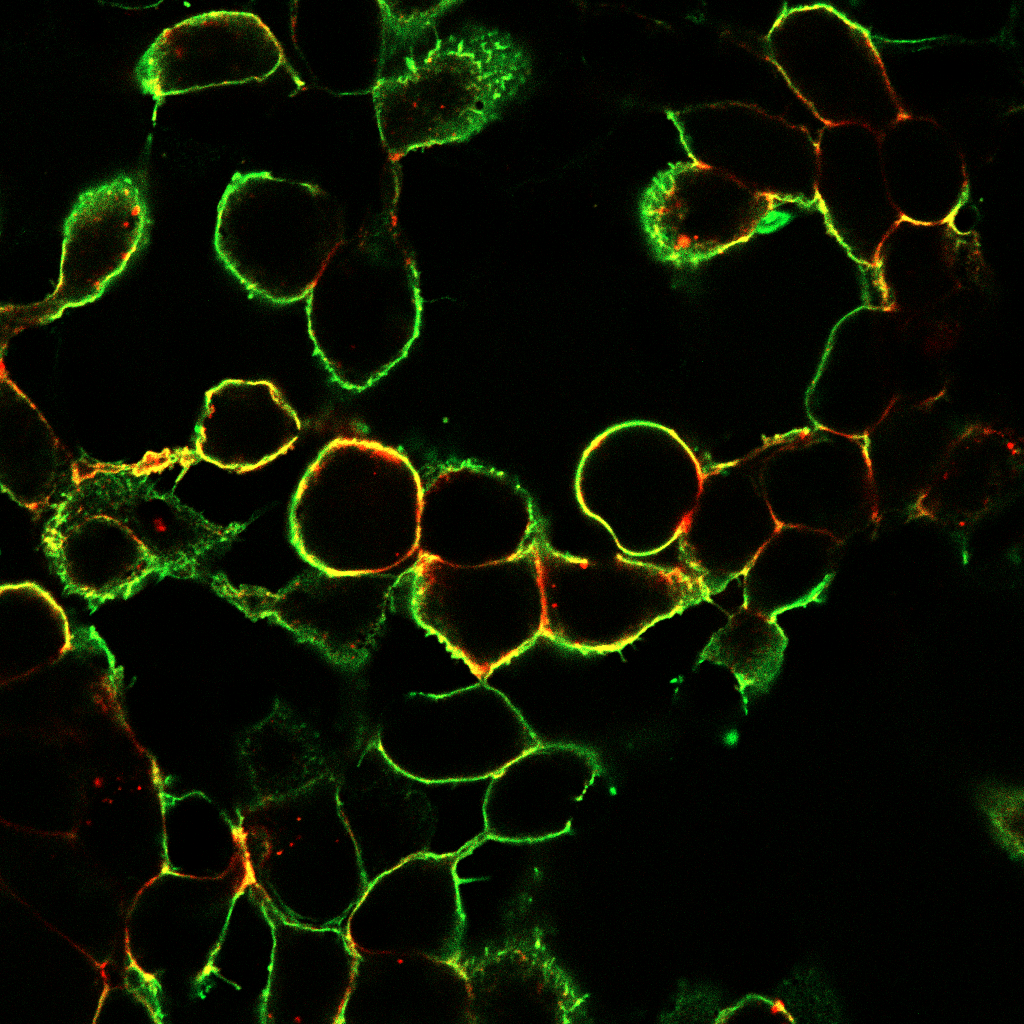

Supplement: Supplementary file 15 — Source data Fig. 1 [file 44321_2026_402_MOESM15_ESM.zip › Panel I and J/MDGA1 WT/MDGA1 WT (5).tif]

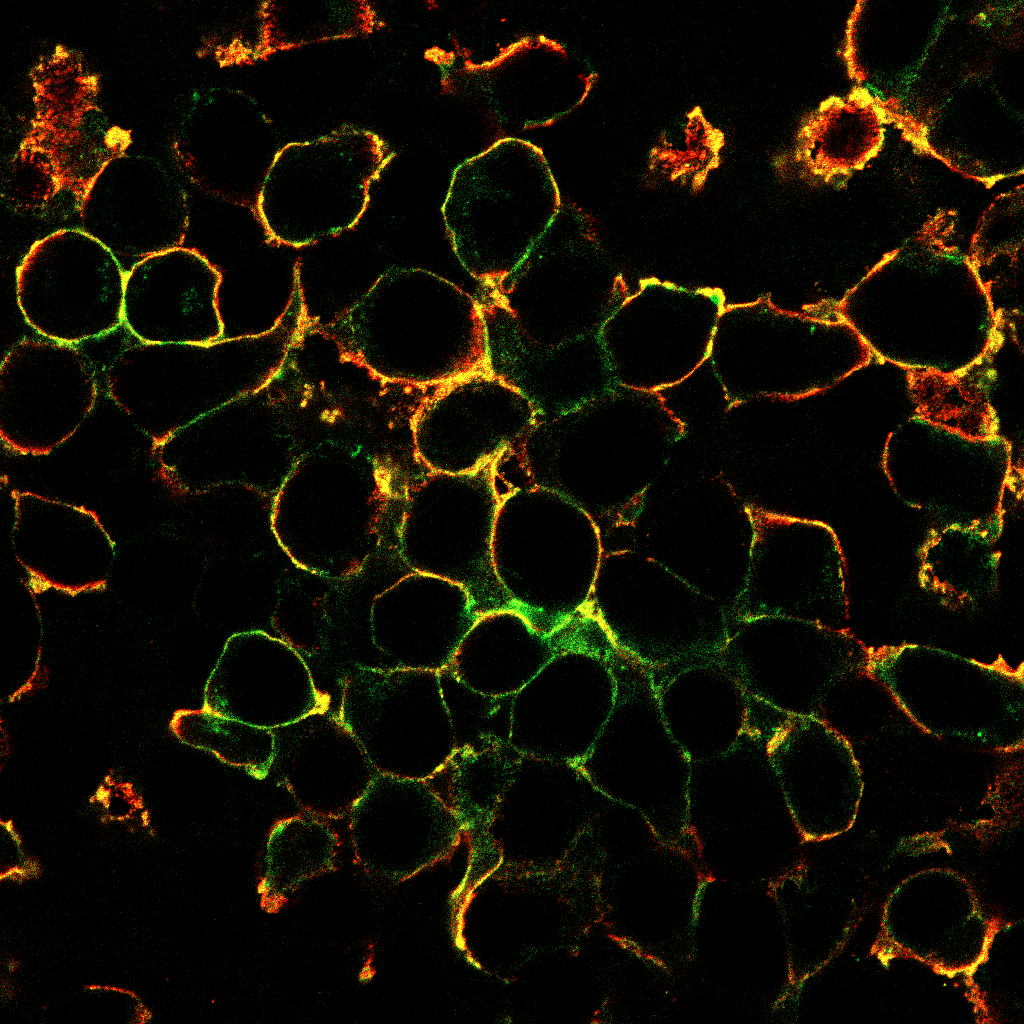

Supplement: Supplementary file 15 — Source data Fig. 1 [file 44321_2026_402_MOESM15_ESM.zip › Panel I and J/MDGA1 WT/MDGA1 WT (6).tif]

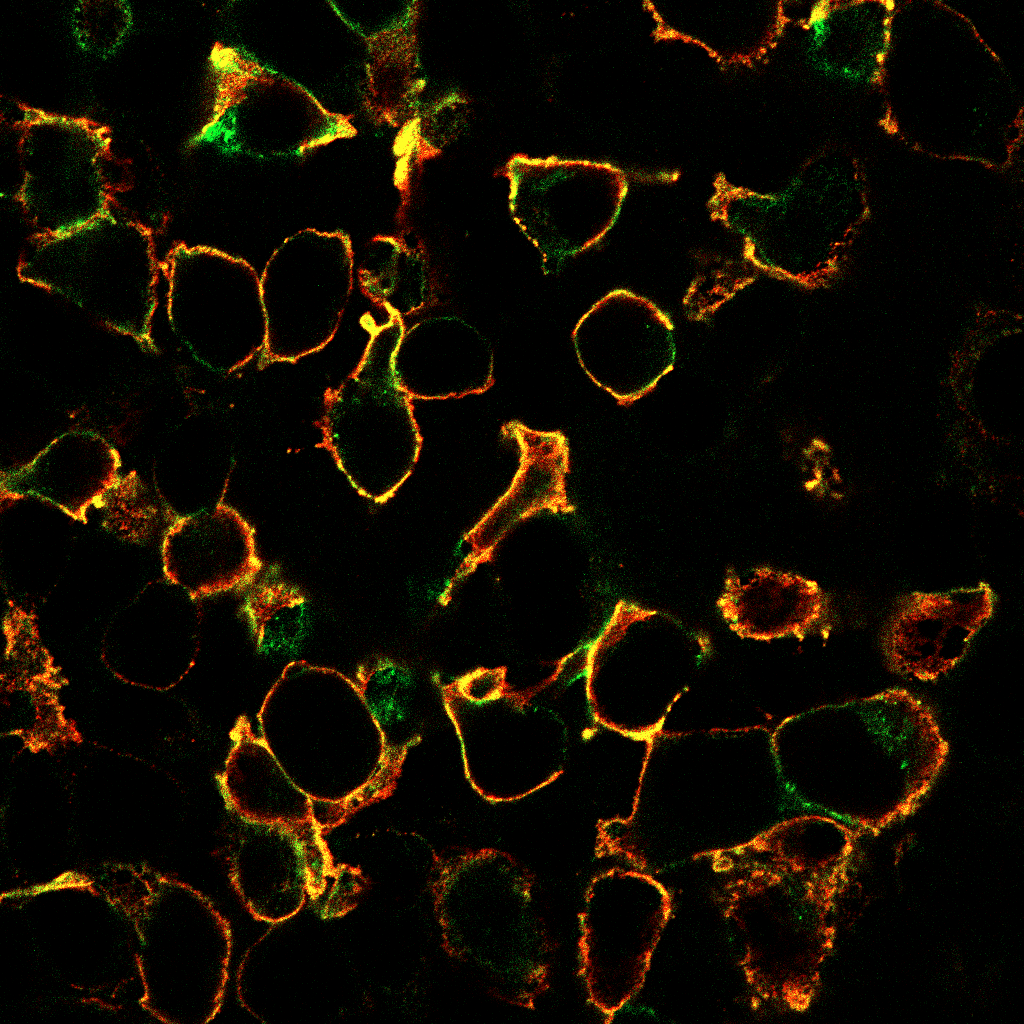

Supplement: Supplementary file 15 — Source data Fig. 1 [file 44321_2026_402_MOESM15_ESM.zip › Panel I and J/MDGA1 WT/MDGA1 WT (7).tif]

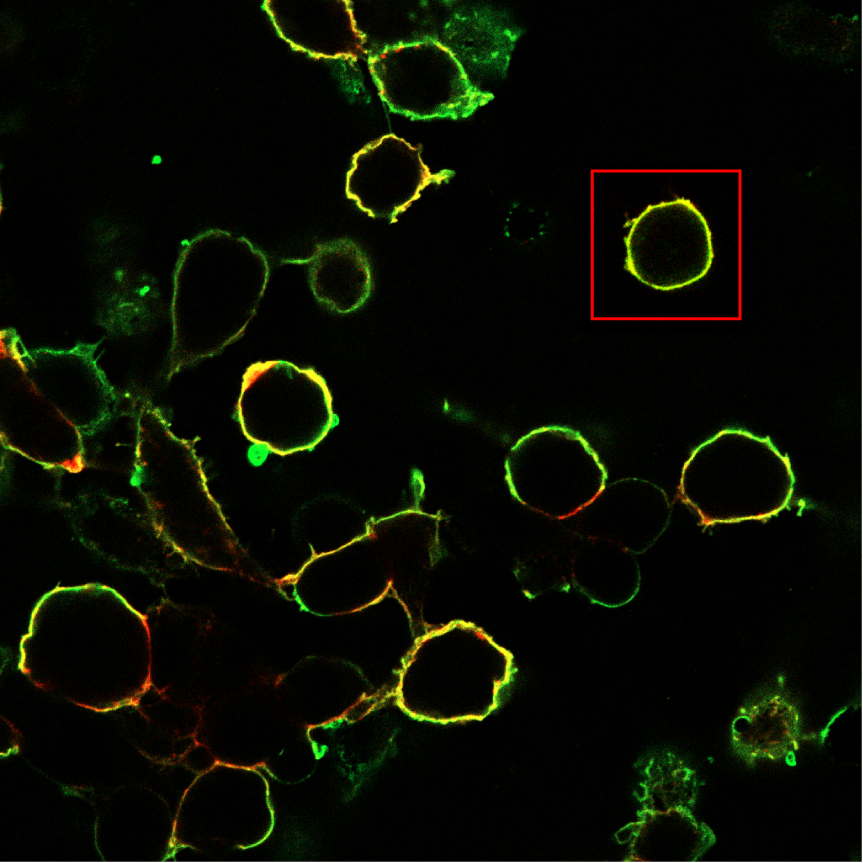

Supplement: Supplementary file 15 — Source data Fig. 1 [file 44321_2026_402_MOESM15_ESM.zip › Panel I and J/MDGA1 WT.tif]

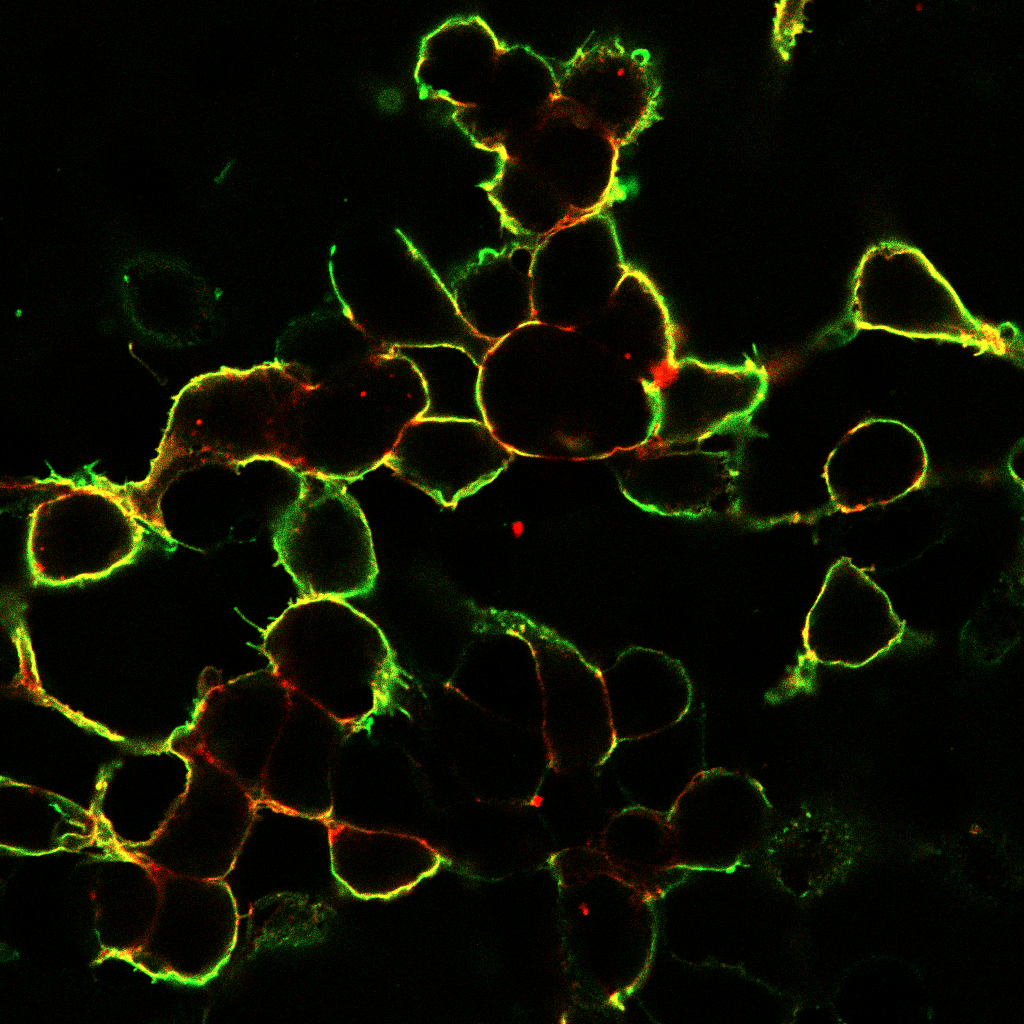

Supplement: Supplementary file 15 — Source data Fig. 1 [file 44321_2026_402_MOESM15_ESM.zip › Panel I and J/MDGA1 Y635C, E756Q/MDGA1 Y635C, E756Q (1).tif]

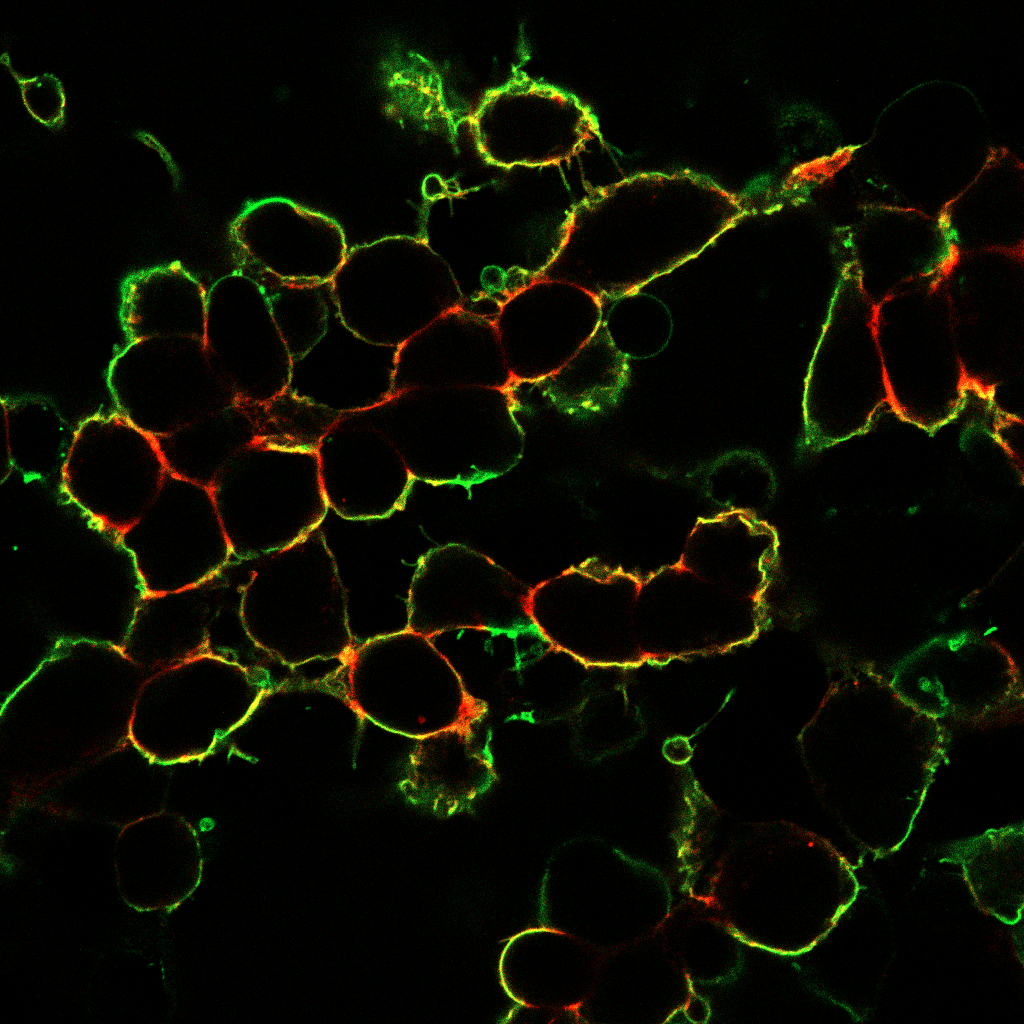

Supplement: Supplementary file 15 — Source data Fig. 1 [file 44321_2026_402_MOESM15_ESM.zip › Panel I and J/MDGA1 Y635C, E756Q/MDGA1 Y635C, E756Q (2).tif]

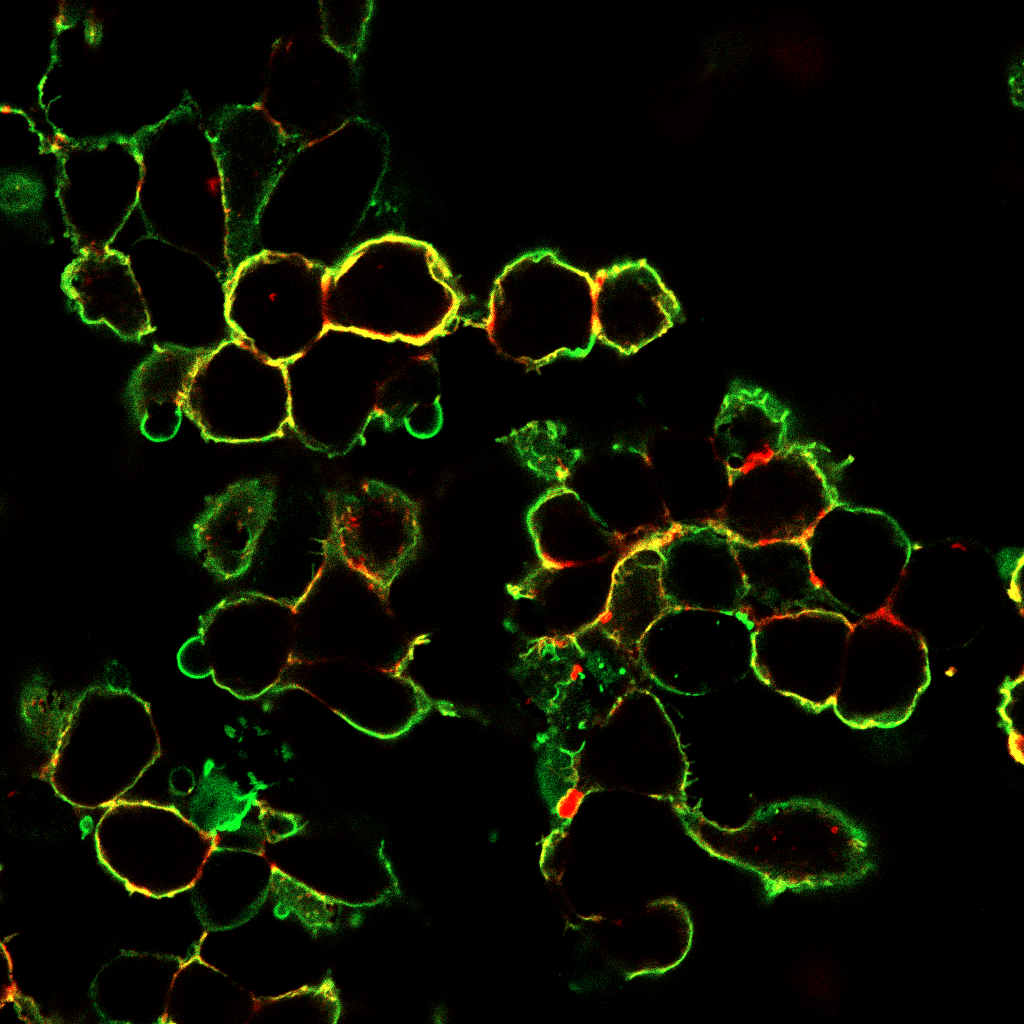

Supplement: Supplementary file 15 — Source data Fig. 1 [file 44321_2026_402_MOESM15_ESM.zip › Panel I and J/MDGA1 Y635C, E756Q/MDGA1 Y635C, E756Q (3).tif]

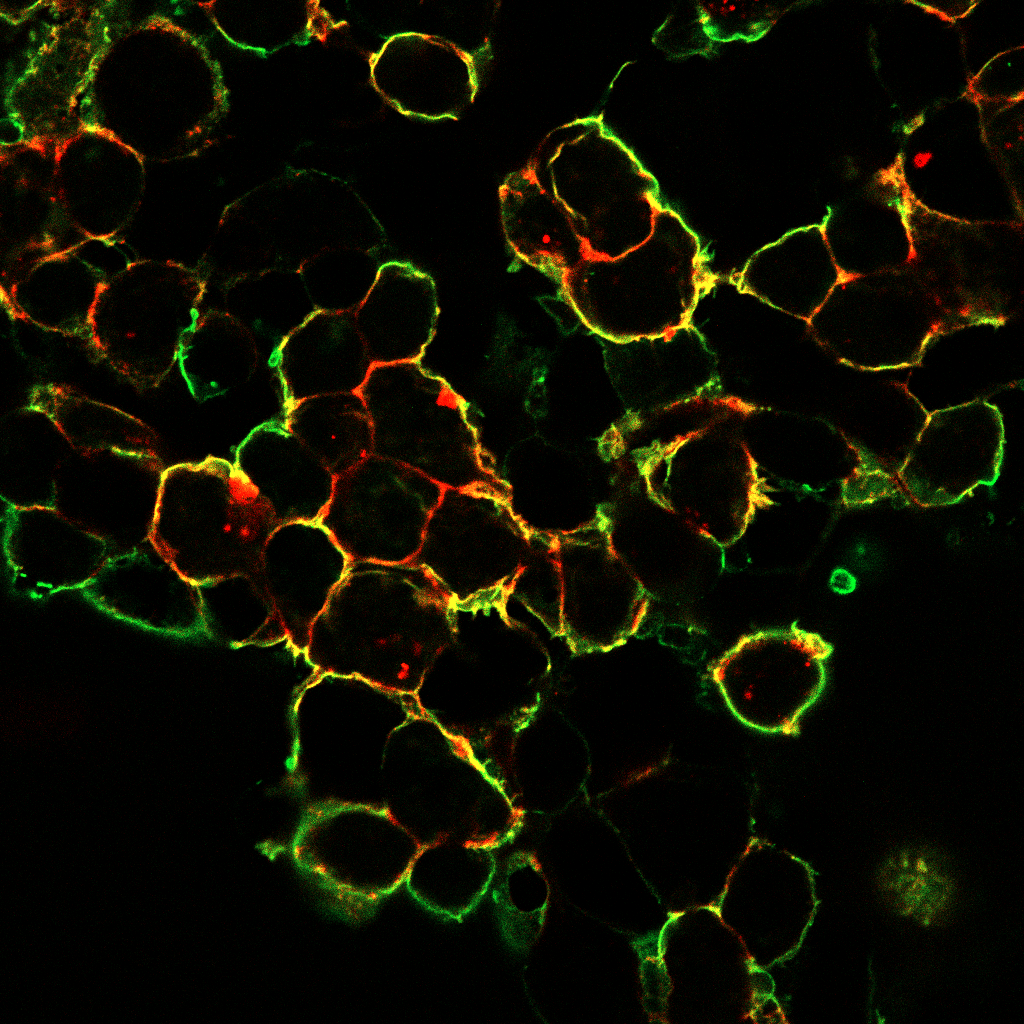

Supplement: Supplementary file 15 — Source data Fig. 1 [file 44321_2026_402_MOESM15_ESM.zip › Panel I and J/MDGA1 Y635C, E756Q/MDGA1 Y635C, E756Q (4).tif]

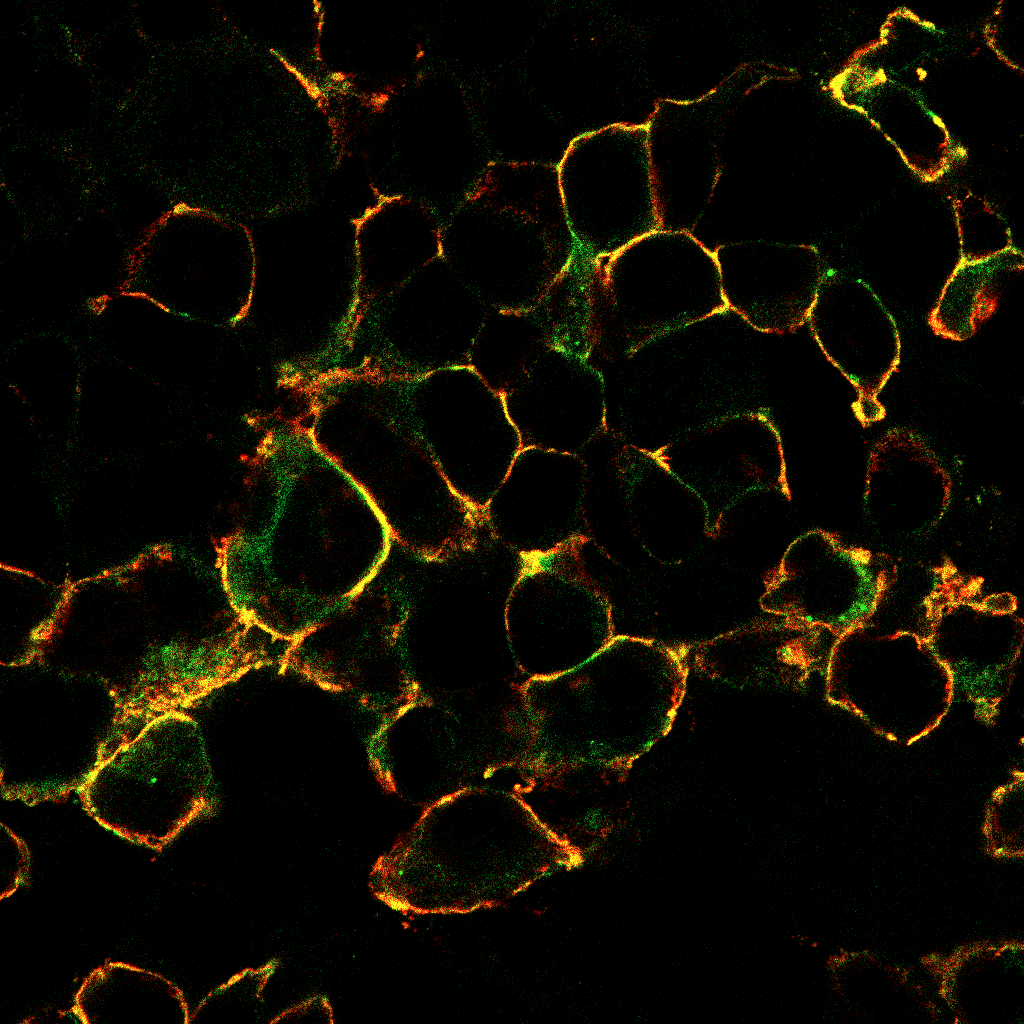

Supplement: Supplementary file 15 — Source data Fig. 1 [file 44321_2026_402_MOESM15_ESM.zip › Panel I and J/MDGA1 Y635C, E756Q/MDGA1 Y635C, E756Q (5).tif]

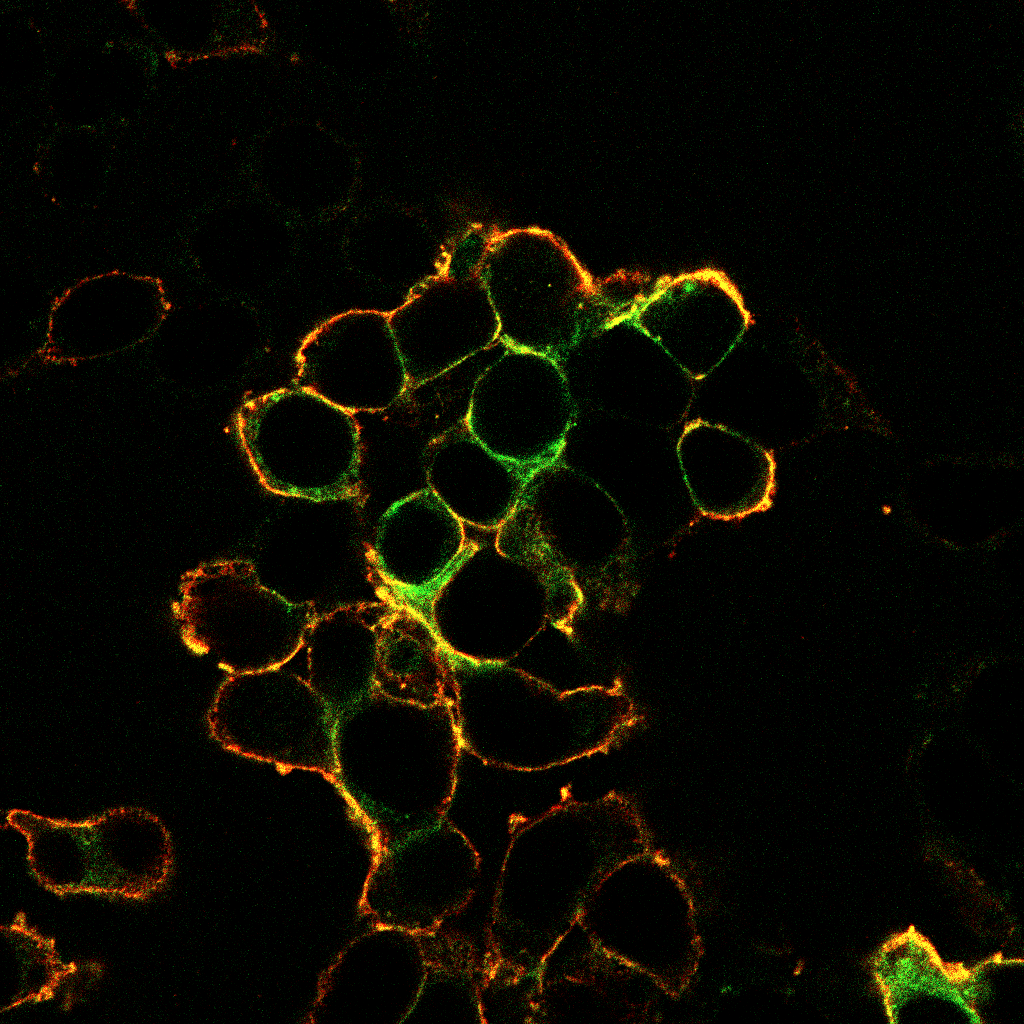

Supplement: Supplementary file 15 — Source data Fig. 1 [file 44321_2026_402_MOESM15_ESM.zip › Panel I and J/MDGA1 Y635C, E756Q/MDGA1 Y635C, E756Q (6).tif]

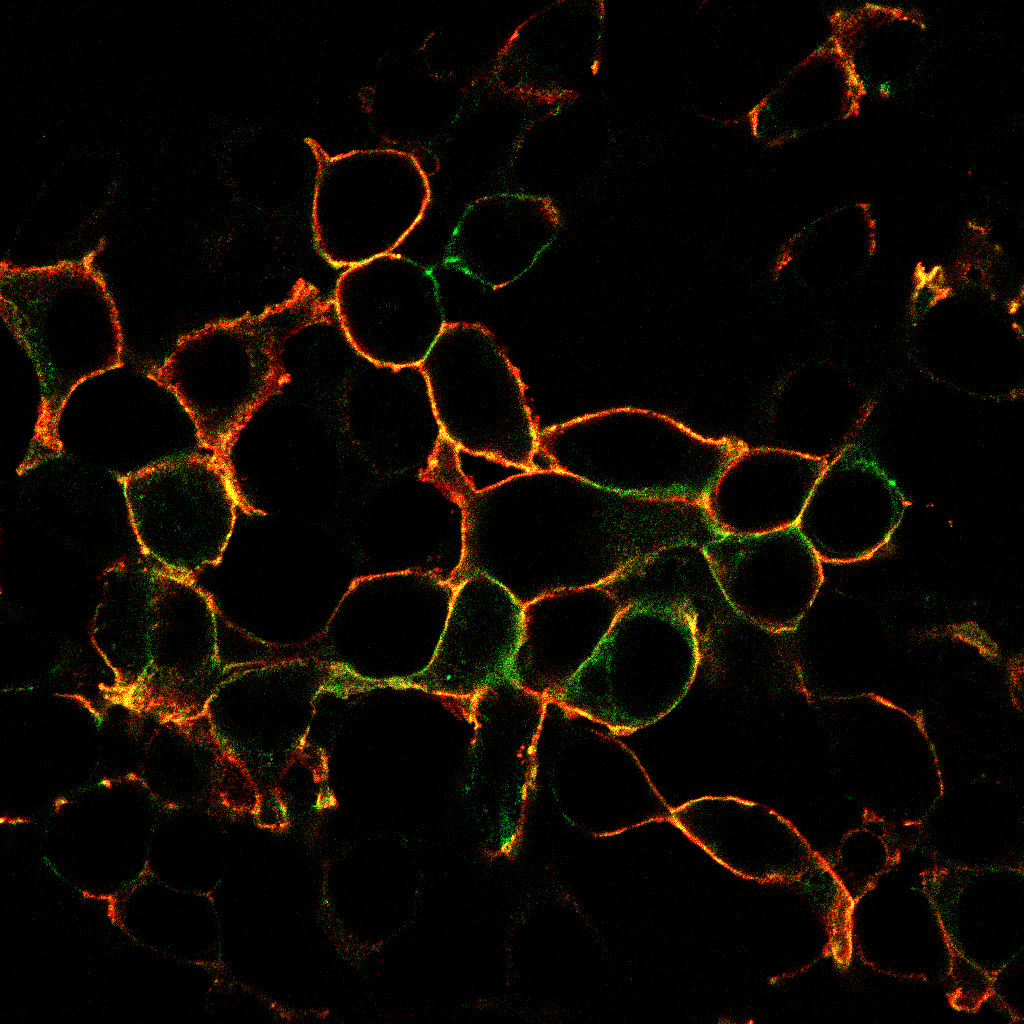

Supplement: Supplementary file 15 — Source data Fig. 1 [file 44321_2026_402_MOESM15_ESM.zip › Panel I and J/MDGA1 Y635C, E756Q/MDGA1 Y635C, E756Q (7).tif]

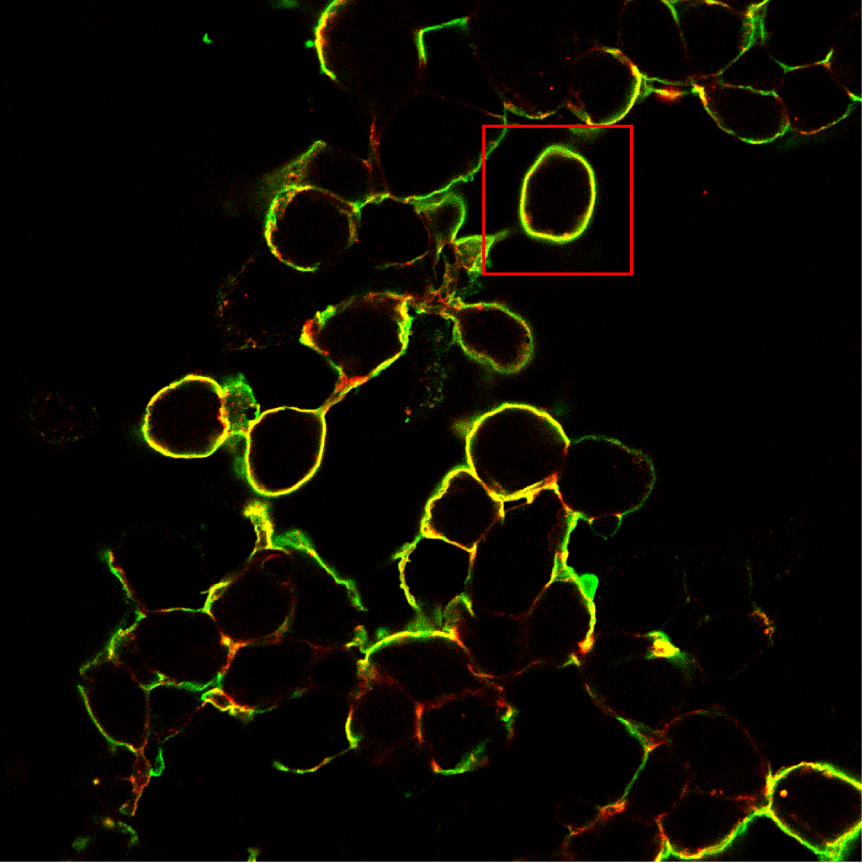

Supplement: Supplementary file 15 — Source data Fig. 1 [file 44321_2026_402_MOESM15_ESM.zip › Panel I and J/MDGA1 Y635C, E756Q.tif]

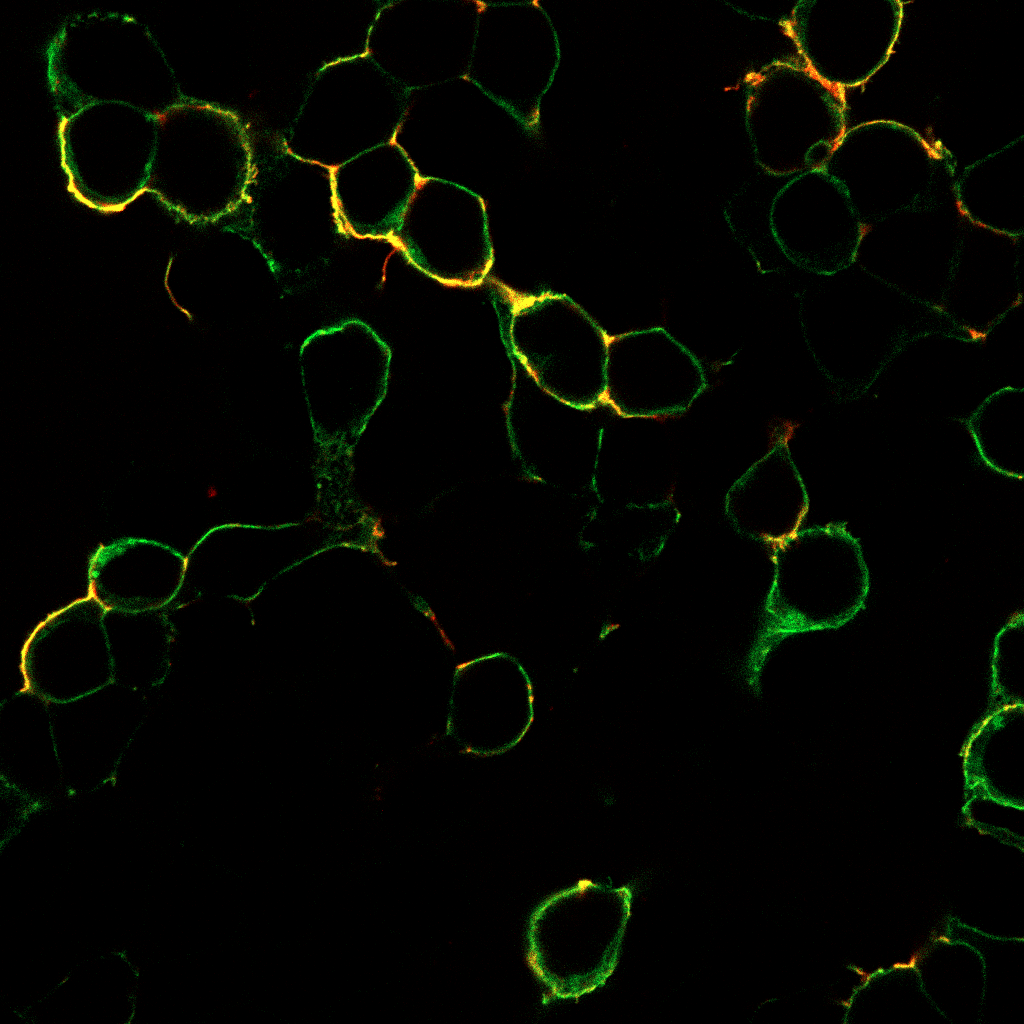

Supplement: Supplementary file 15 — Source data Fig. 1 [file 44321_2026_402_MOESM15_ESM.zip › Panel K and L/Figure 1K. Ig-APP, MDGA1 V116M, A688V/Ig-APP, MDGA1 V116M, A688V (1).tif]

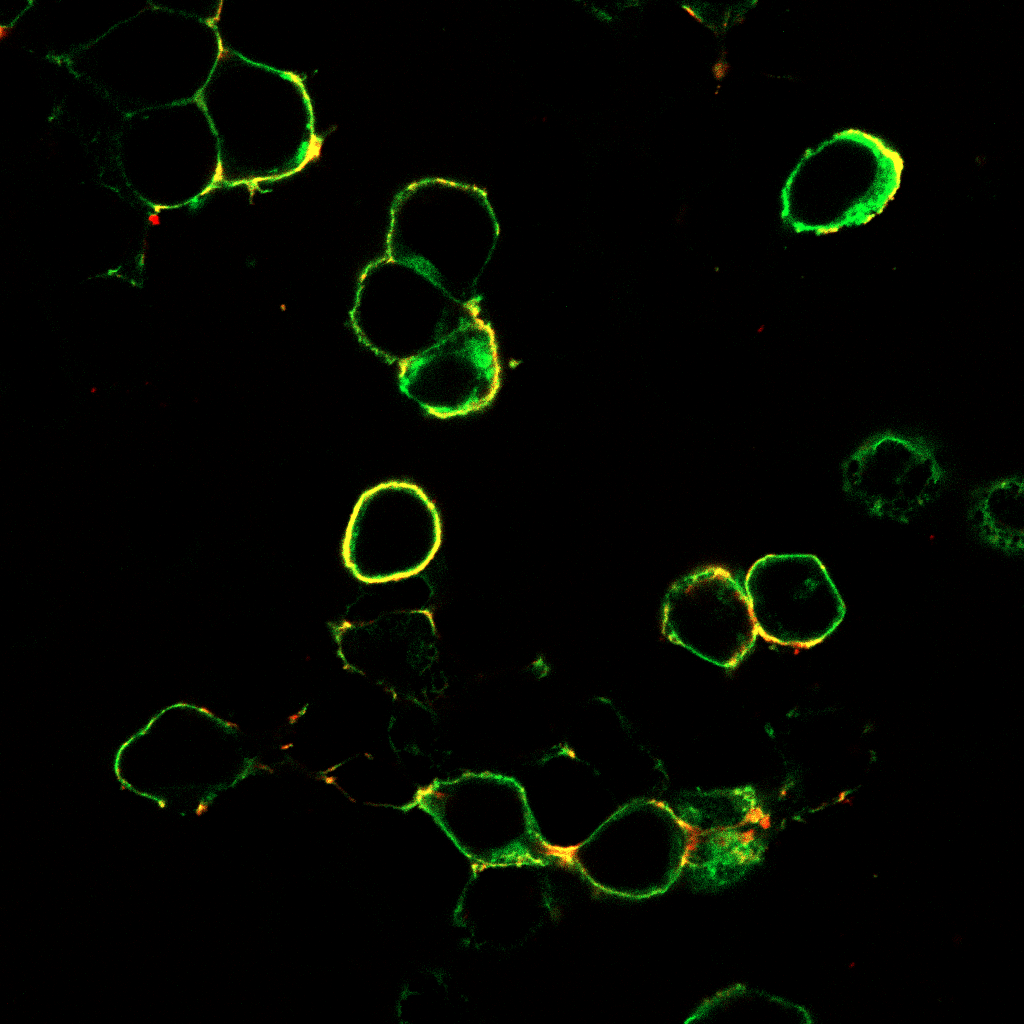

Supplement: Supplementary file 15 — Source data Fig. 1 [file 44321_2026_402_MOESM15_ESM.zip › Panel K and L/Figure 1K. Ig-APP, MDGA1 V116M, A688V/Ig-APP, MDGA1 V116M, A688V (2).tif]

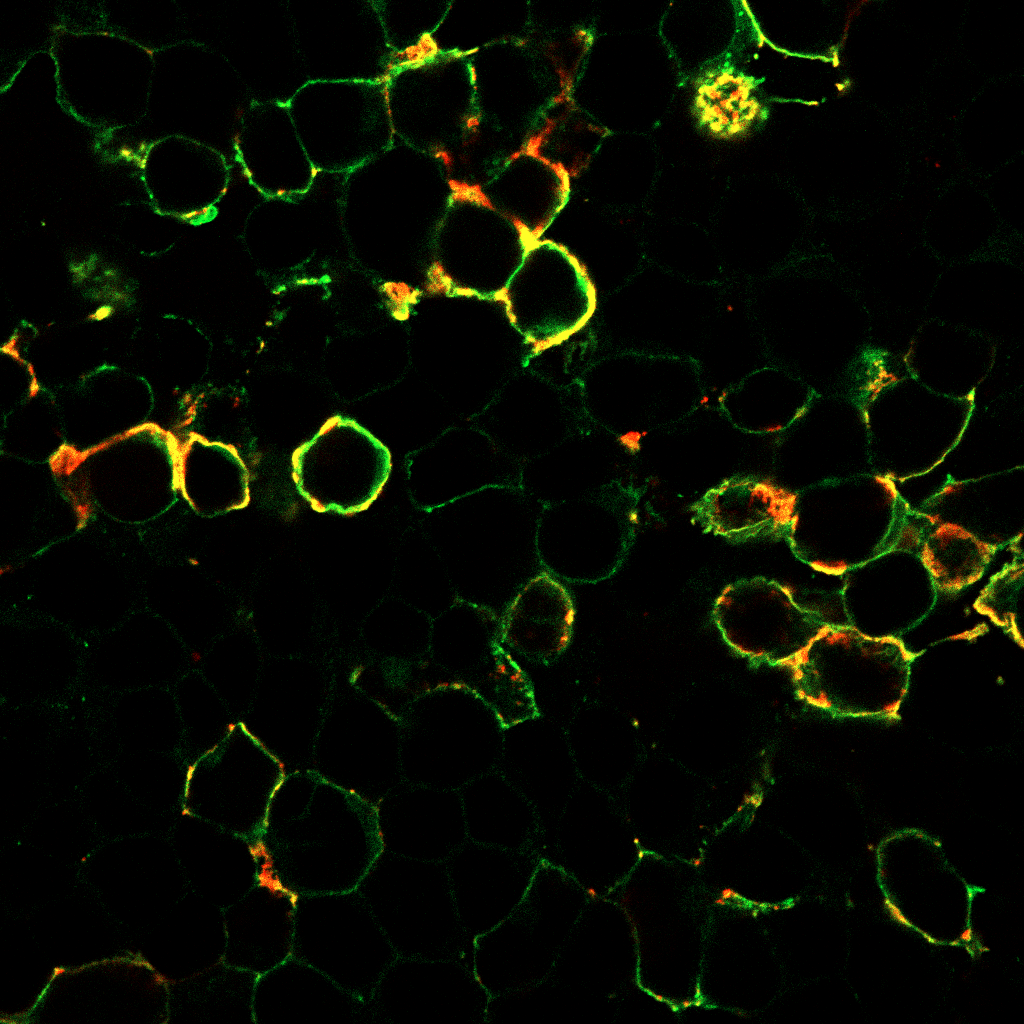

Supplement: Supplementary file 15 — Source data Fig. 1 [file 44321_2026_402_MOESM15_ESM.zip › Panel K and L/Figure 1K. Ig-APP, MDGA1 V116M, A688V/Ig-APP, MDGA1 V116M, A688V (3).tif]

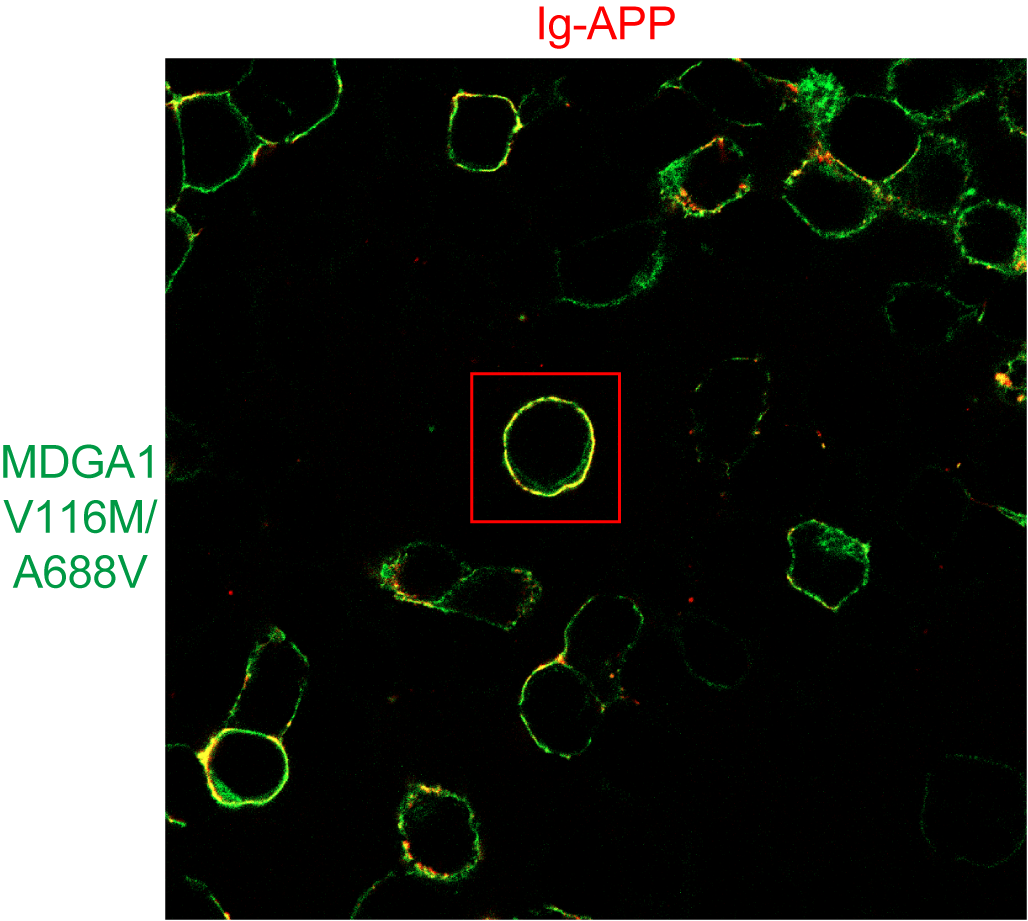

Supplement: Supplementary file 15 — Source data Fig. 1 [file 44321_2026_402_MOESM15_ESM.zip › Panel K and L/Figure 1K. Ig-APP, MDGA1 V116M, A688V.tif]

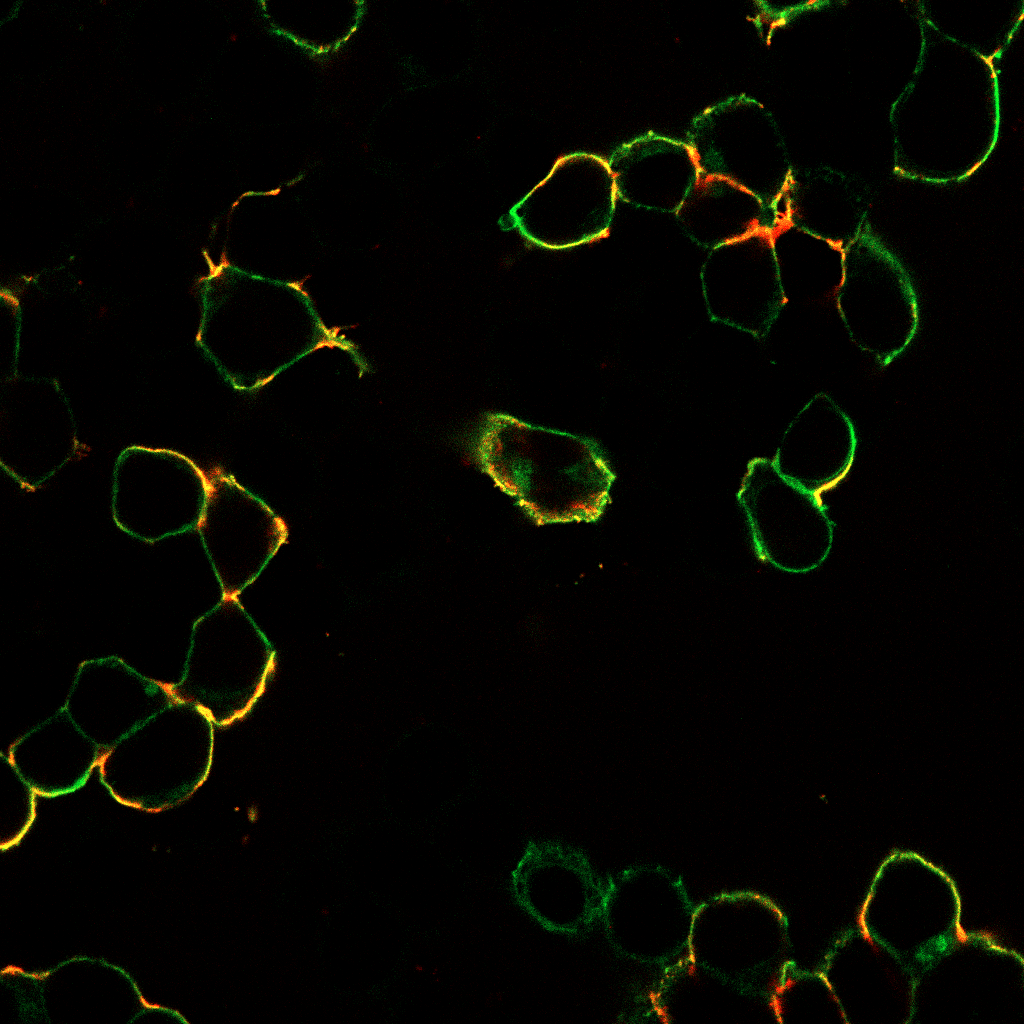

Supplement: Supplementary file 15 — Source data Fig. 1 [file 44321_2026_402_MOESM15_ESM.zip › Panel K and L/Figure 1K. Ig-APP, MDGA1 WT/Ig-APP, MDGA1 WT (1).tif]

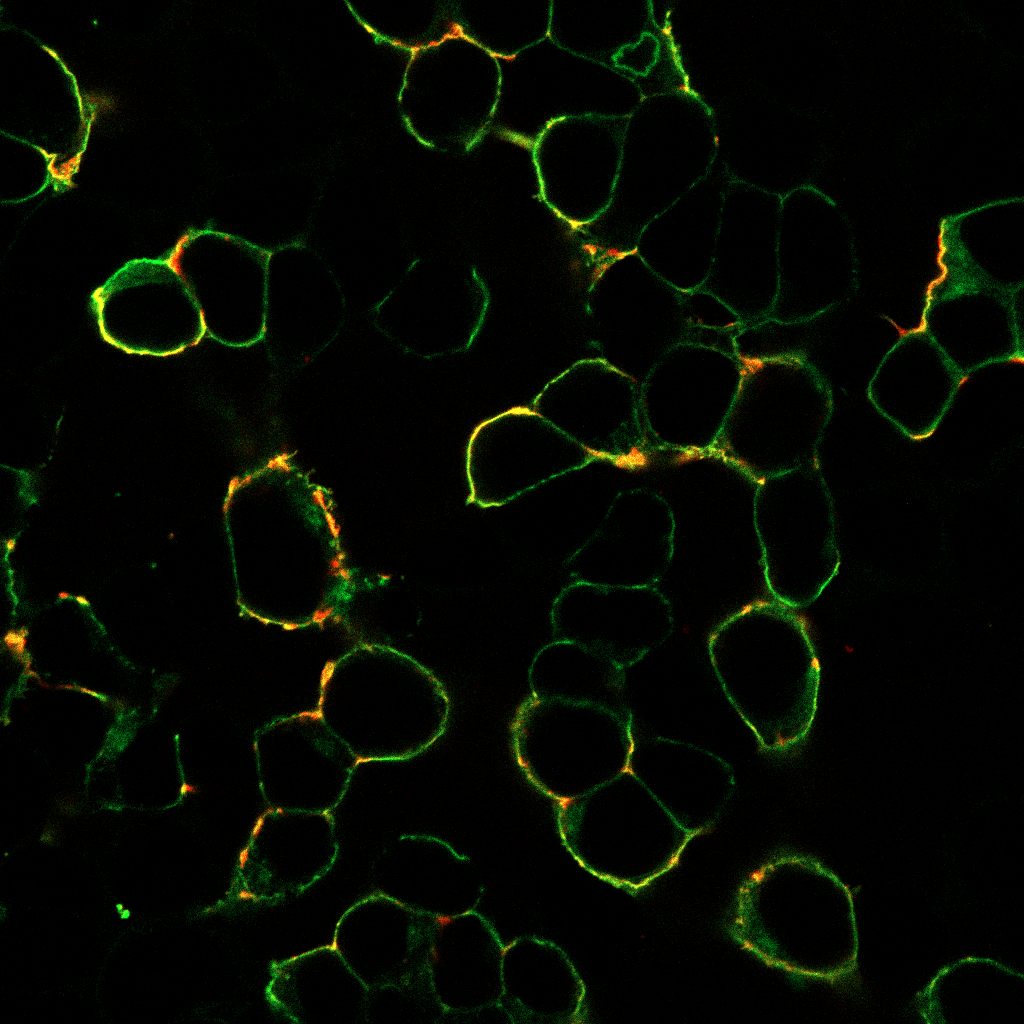

Supplement: Supplementary file 15 — Source data Fig. 1 [file 44321_2026_402_MOESM15_ESM.zip › Panel K and L/Figure 1K. Ig-APP, MDGA1 WT/Ig-APP, MDGA1 WT (2).tif]

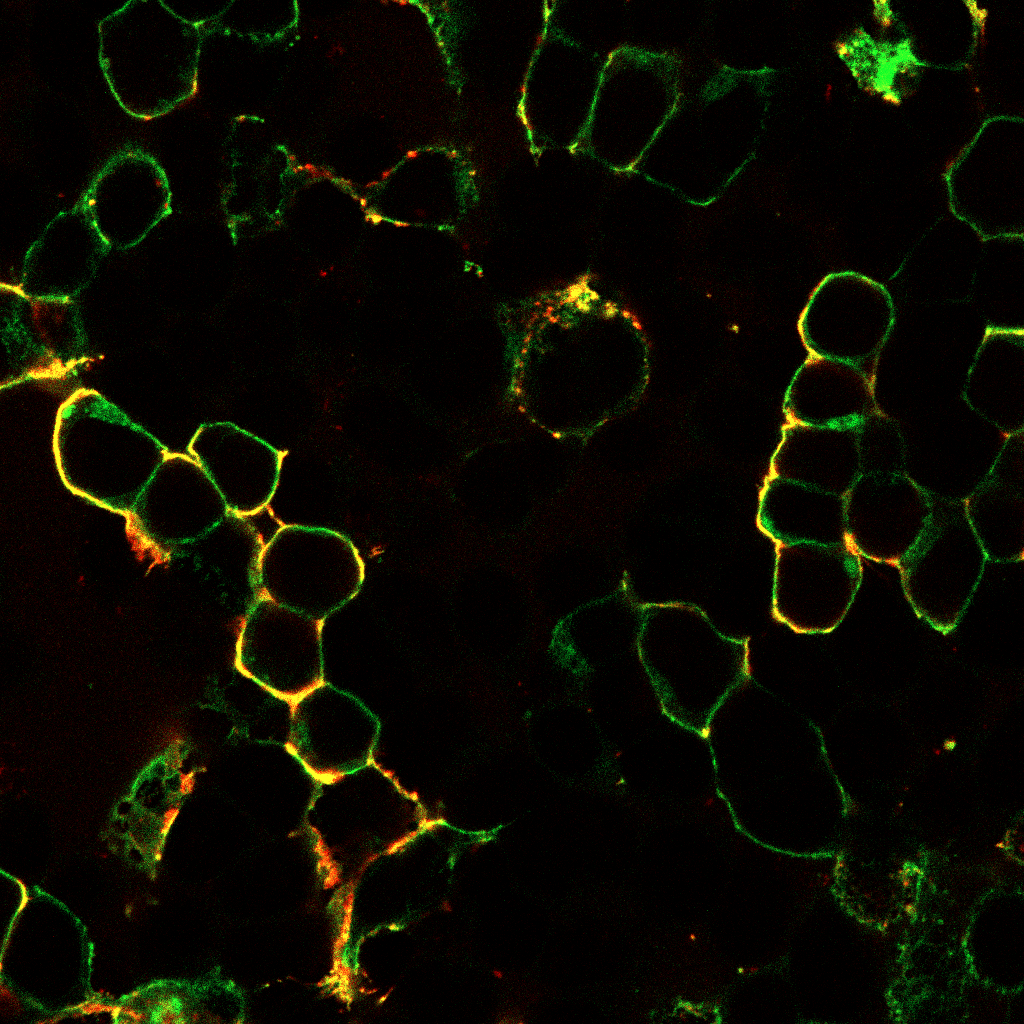

Supplement: Supplementary file 15 — Source data Fig. 1 [file 44321_2026_402_MOESM15_ESM.zip › Panel K and L/Figure 1K. Ig-APP, MDGA1 WT/Ig-APP, MDGA1 WT (3).tif]

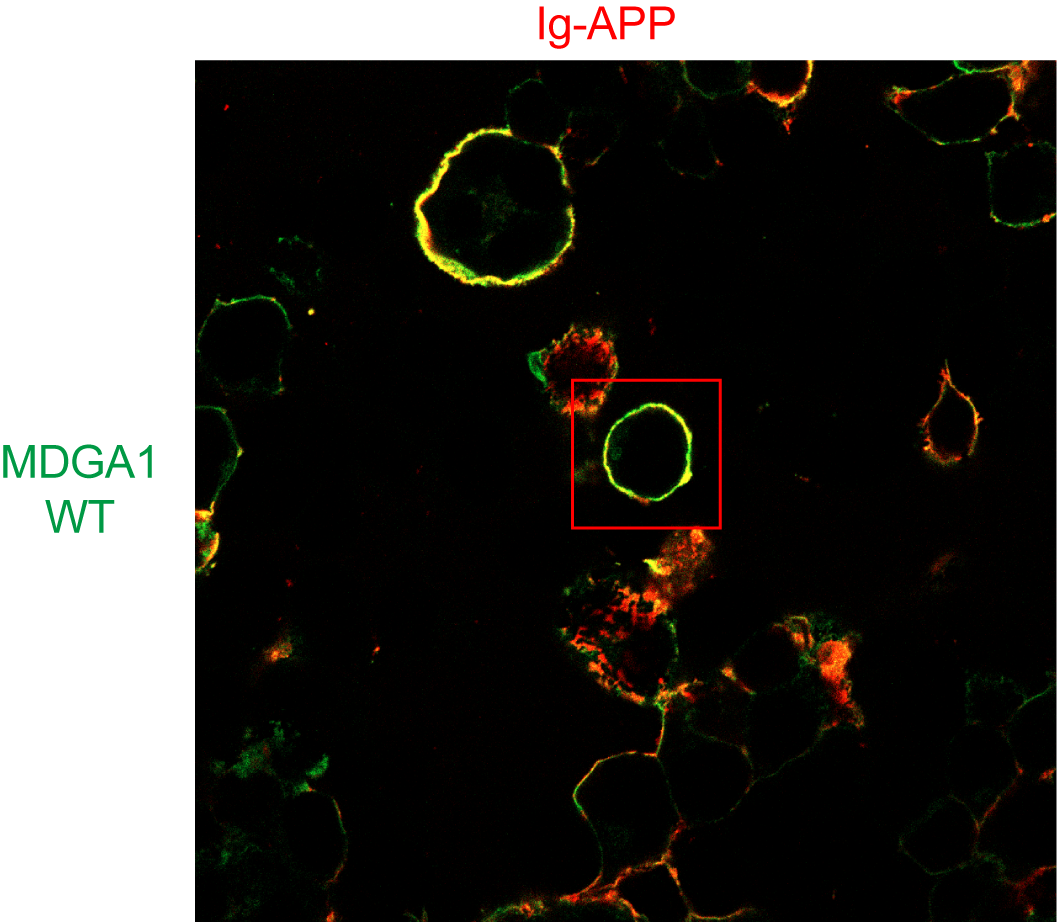

Supplement: Supplementary file 15 — Source data Fig. 1 [file 44321_2026_402_MOESM15_ESM.zip › Panel K and L/Figure 1K. Ig-APP, MDGA1 WT.tif]

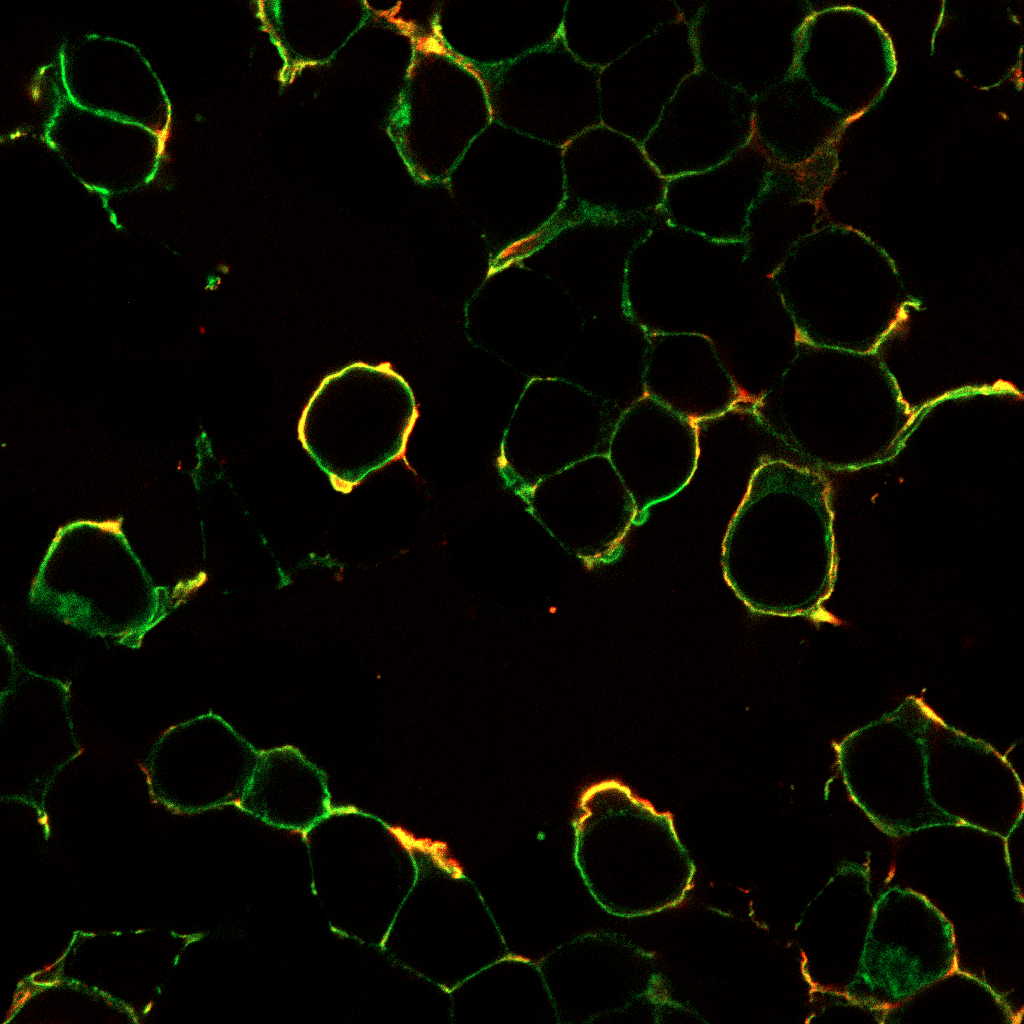

Supplement: Supplementary file 15 — Source data Fig. 1 [file 44321_2026_402_MOESM15_ESM.zip › Panel K and L/Figure 1K. Ig-APP, MDGA1 Y635C, E756Q/Ig-APP, MDGA1 Y635C, E756Q (1).tif]

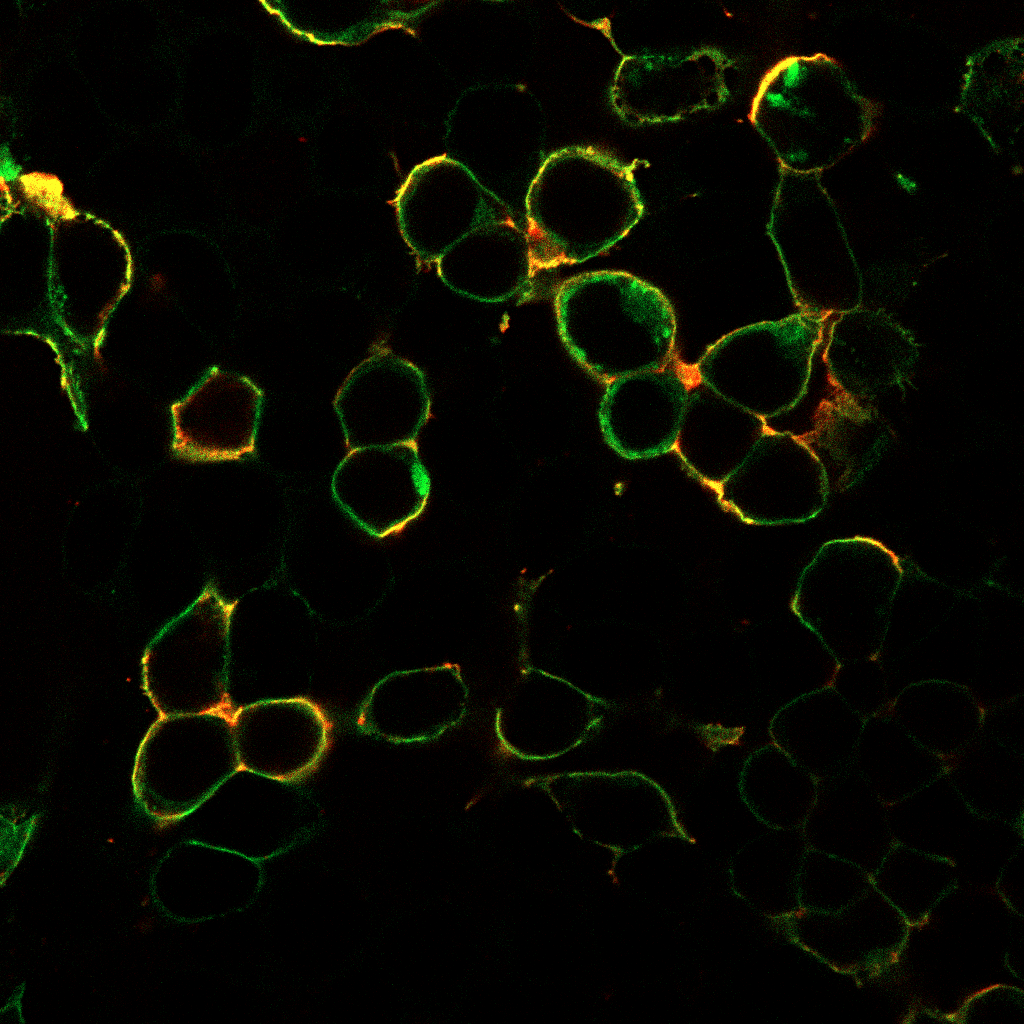

Supplement: Supplementary file 15 — Source data Fig. 1 [file 44321_2026_402_MOESM15_ESM.zip › Panel K and L/Figure 1K. Ig-APP, MDGA1 Y635C, E756Q/Ig-APP, MDGA1 Y635C, E756Q (2).tif]

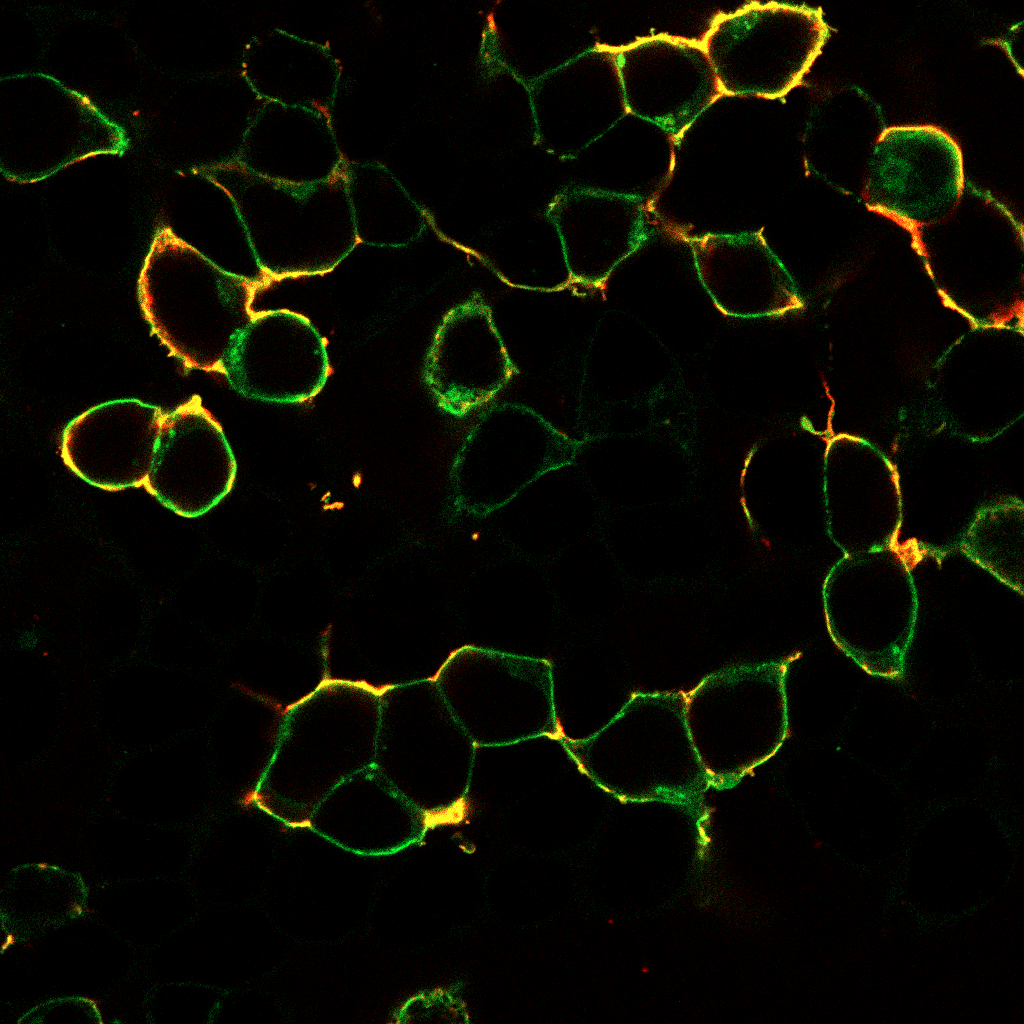

Supplement: Supplementary file 15 — Source data Fig. 1 [file 44321_2026_402_MOESM15_ESM.zip › Panel K and L/Figure 1K. Ig-APP, MDGA1 Y635C, E756Q/Ig-APP, MDGA1 Y635C, E756Q (3).tif]

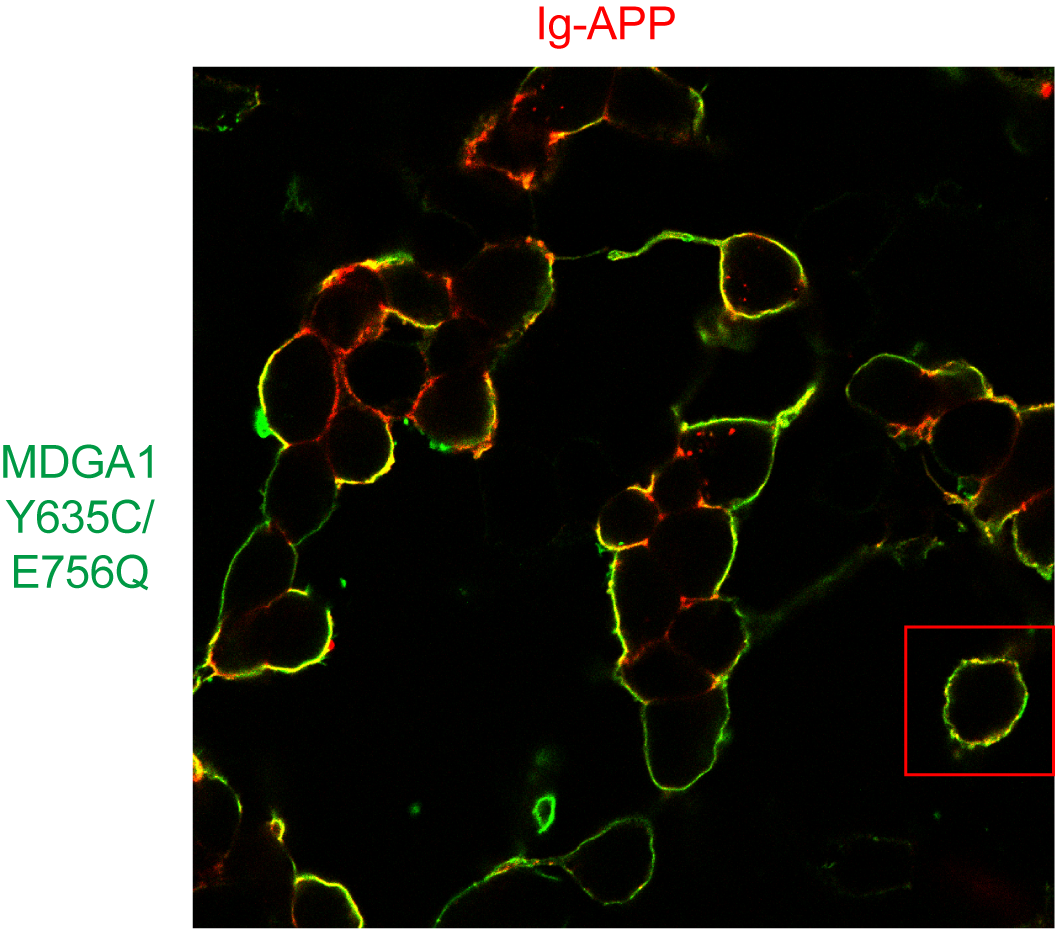

Supplement: Supplementary file 15 — Source data Fig. 1 [file 44321_2026_402_MOESM15_ESM.zip › Panel K and L/Figure 1K. Ig-APP, MDGA1 Y635C, E756Q.tif]

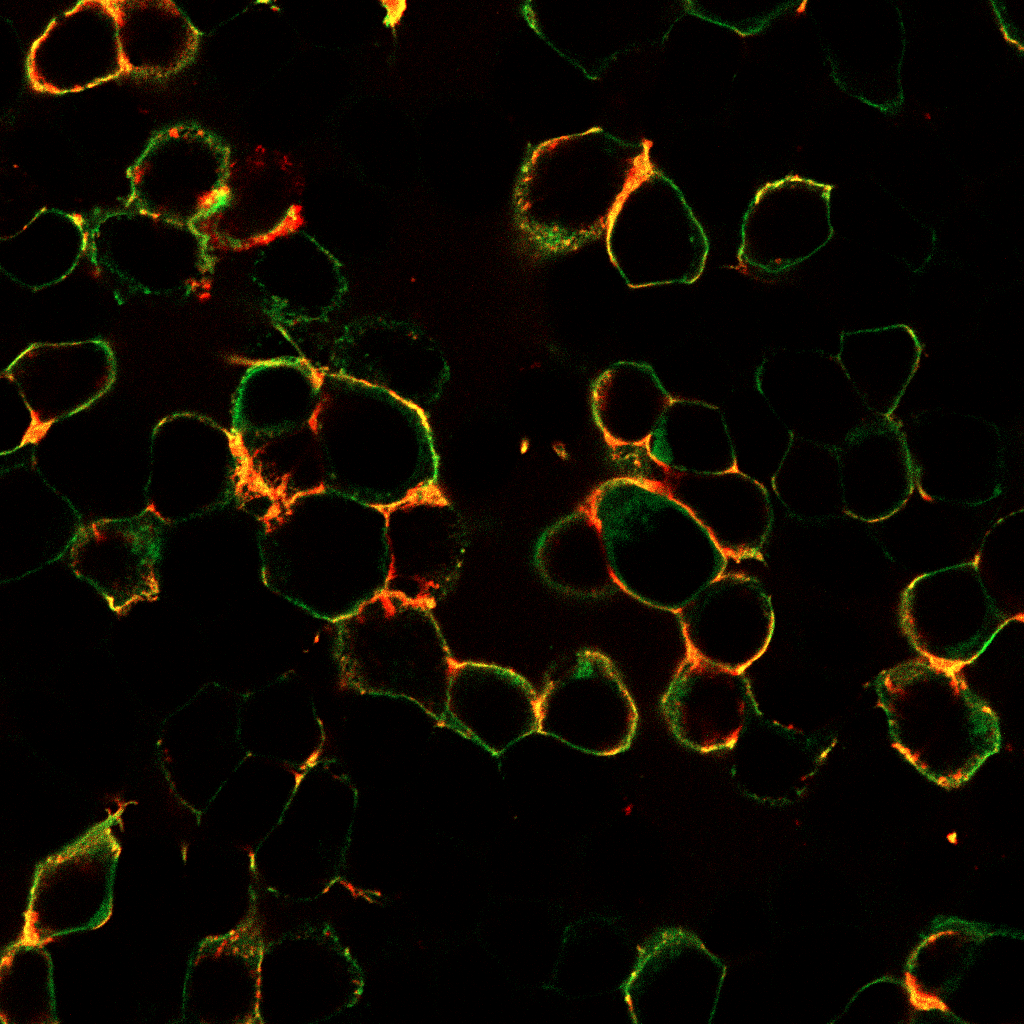

Supplement: Supplementary file 15 — Source data Fig. 1 [file 44321_2026_402_MOESM15_ESM.zip › Panel K and L/Figure 1K. Ig-Nlgn2, MDGA1 V116M, A688V/Ig-Nlgn2, MDGA1 V116M, A688V (1).tif]

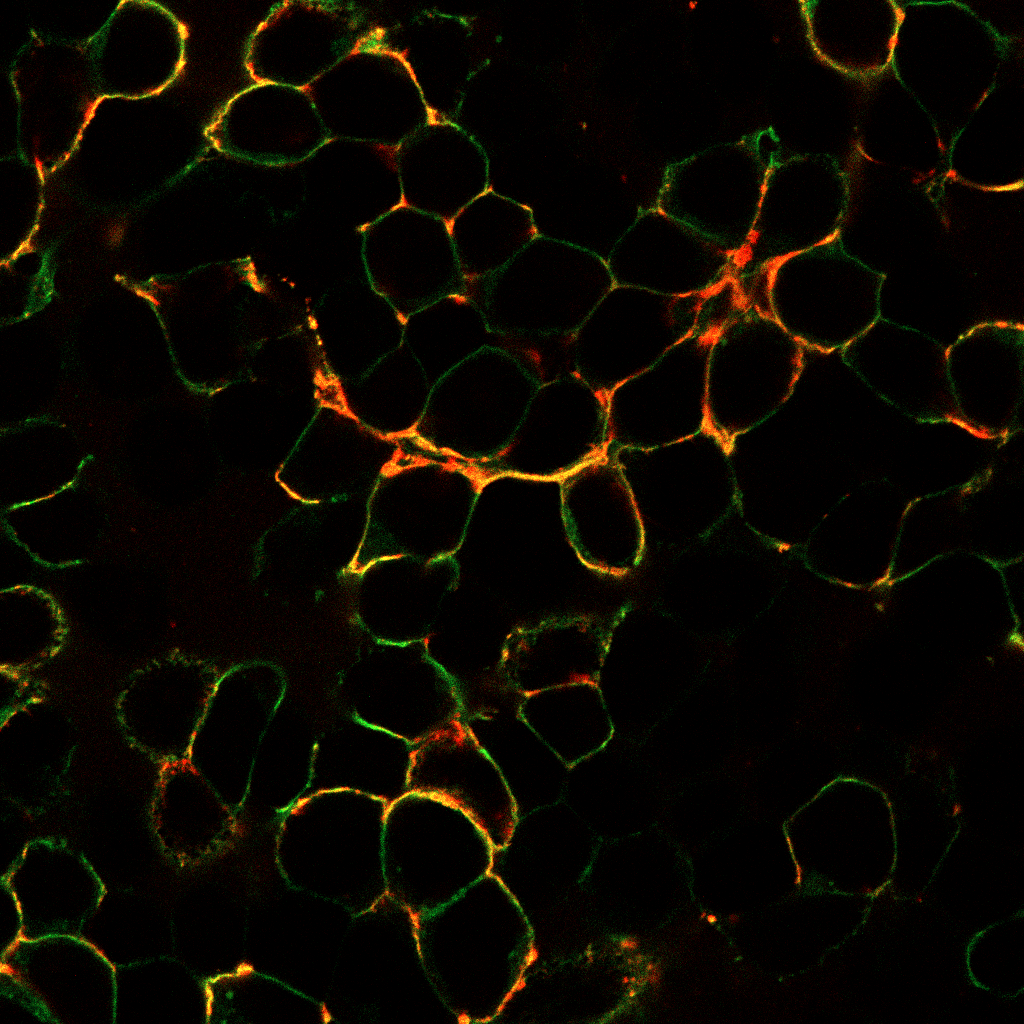

Supplement: Supplementary file 15 — Source data Fig. 1 [file 44321_2026_402_MOESM15_ESM.zip › Panel K and L/Figure 1K. Ig-Nlgn2, MDGA1 V116M, A688V/Ig-Nlgn2, MDGA1 V116M, A688V (2).tif]

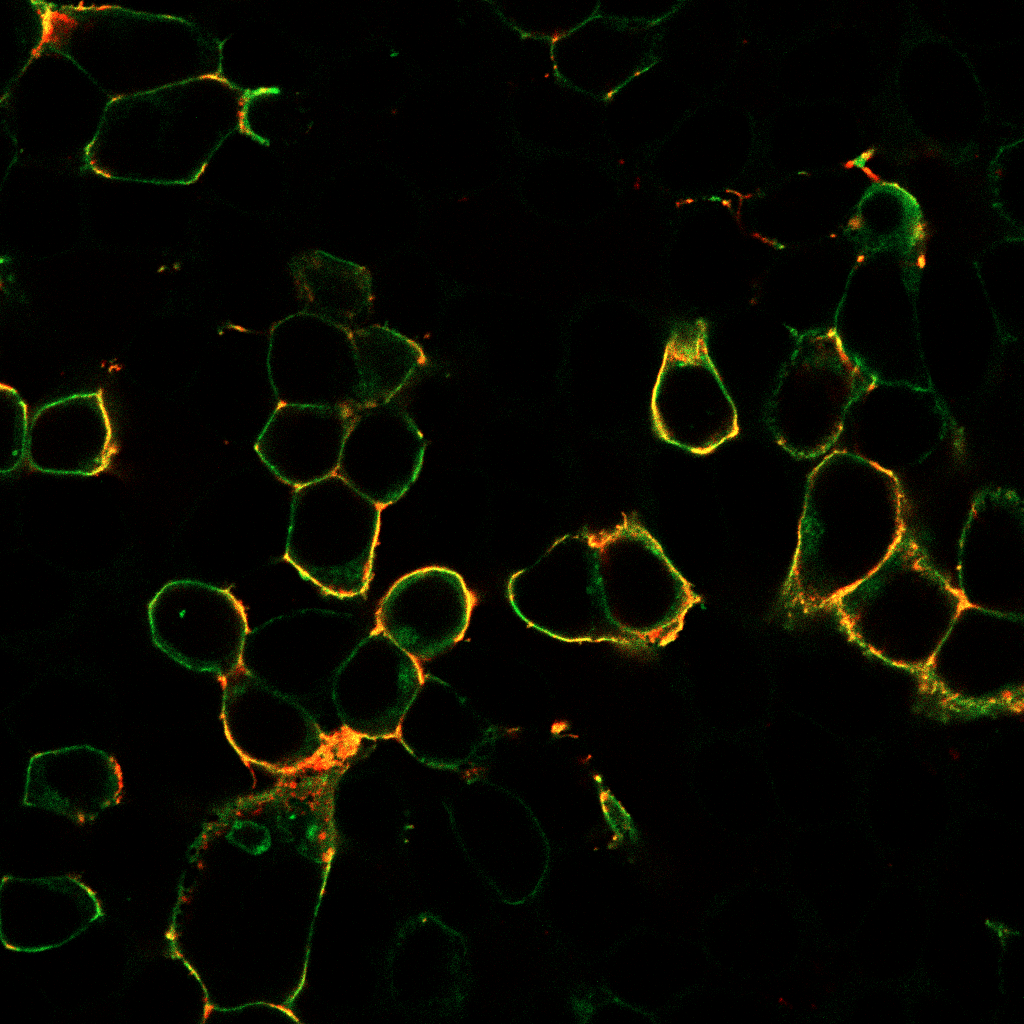

Supplement: Supplementary file 15 — Source data Fig. 1 [file 44321_2026_402_MOESM15_ESM.zip › Panel K and L/Figure 1K. Ig-Nlgn2, MDGA1 V116M, A688V/Ig-Nlgn2, MDGA1 V116M, A688V (3).tif]

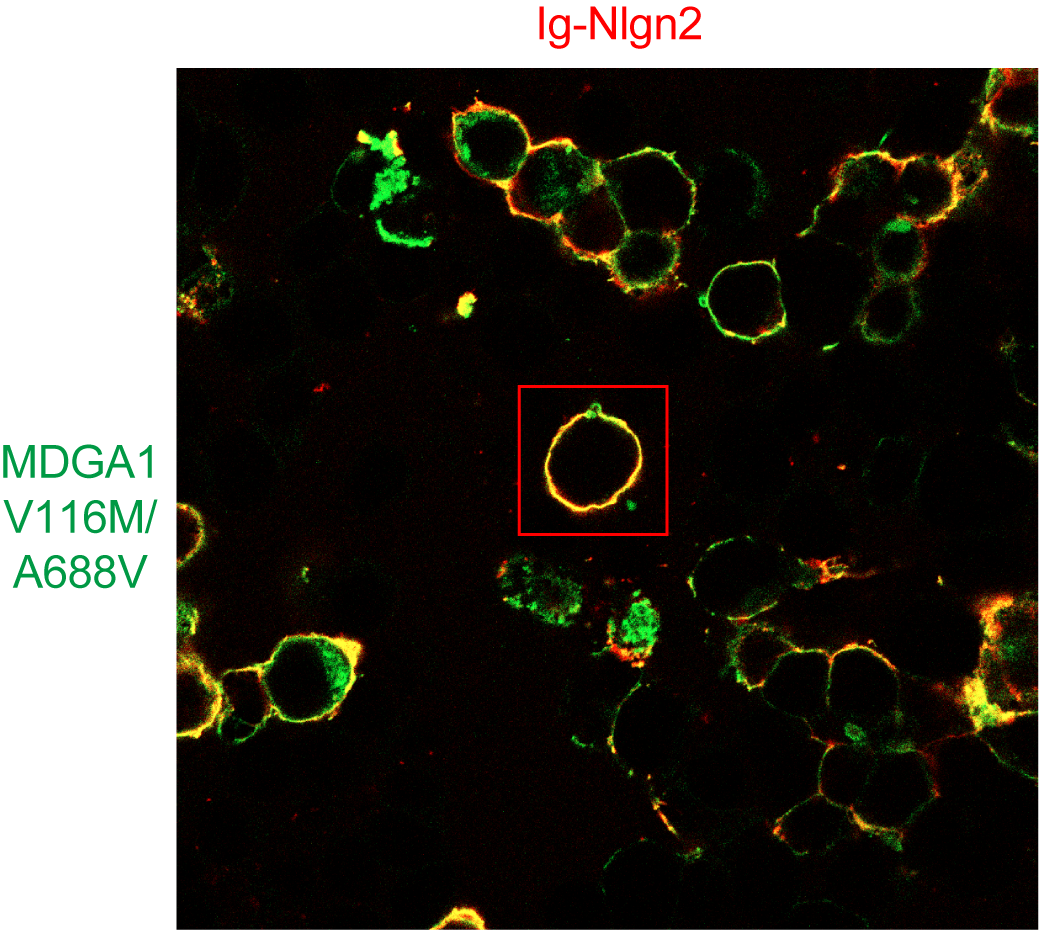

Supplement: Supplementary file 15 — Source data Fig. 1 [file 44321_2026_402_MOESM15_ESM.zip › Panel K and L/Figure 1K. Ig-Nlgn2, MDGA1 V116M, A688V.tif]

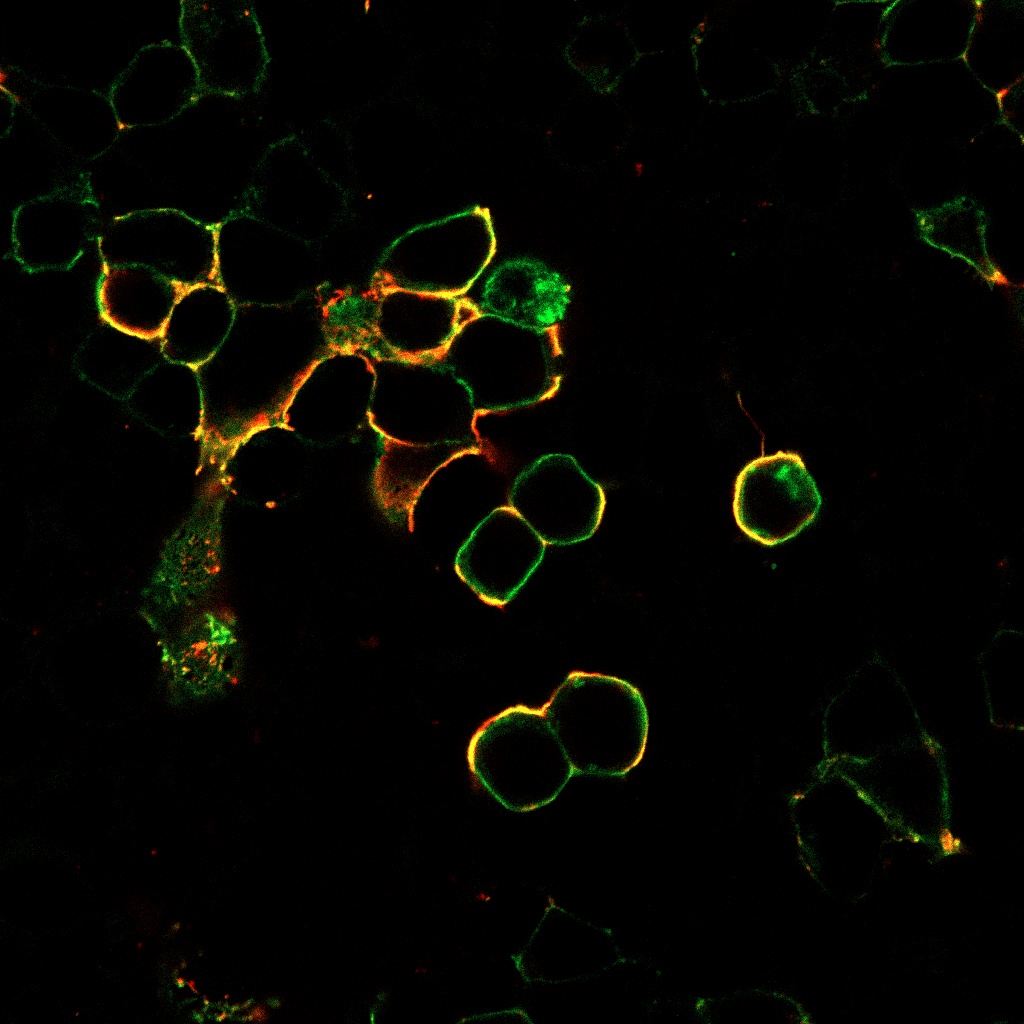

Supplement: Supplementary file 15 — Source data Fig. 1 [file 44321_2026_402_MOESM15_ESM.zip › Panel K and L/Figure 1K. Ig-Nlgn2, MDGA1 WT/Ig-Nlgn2, MDGA1 WT (1).tif]

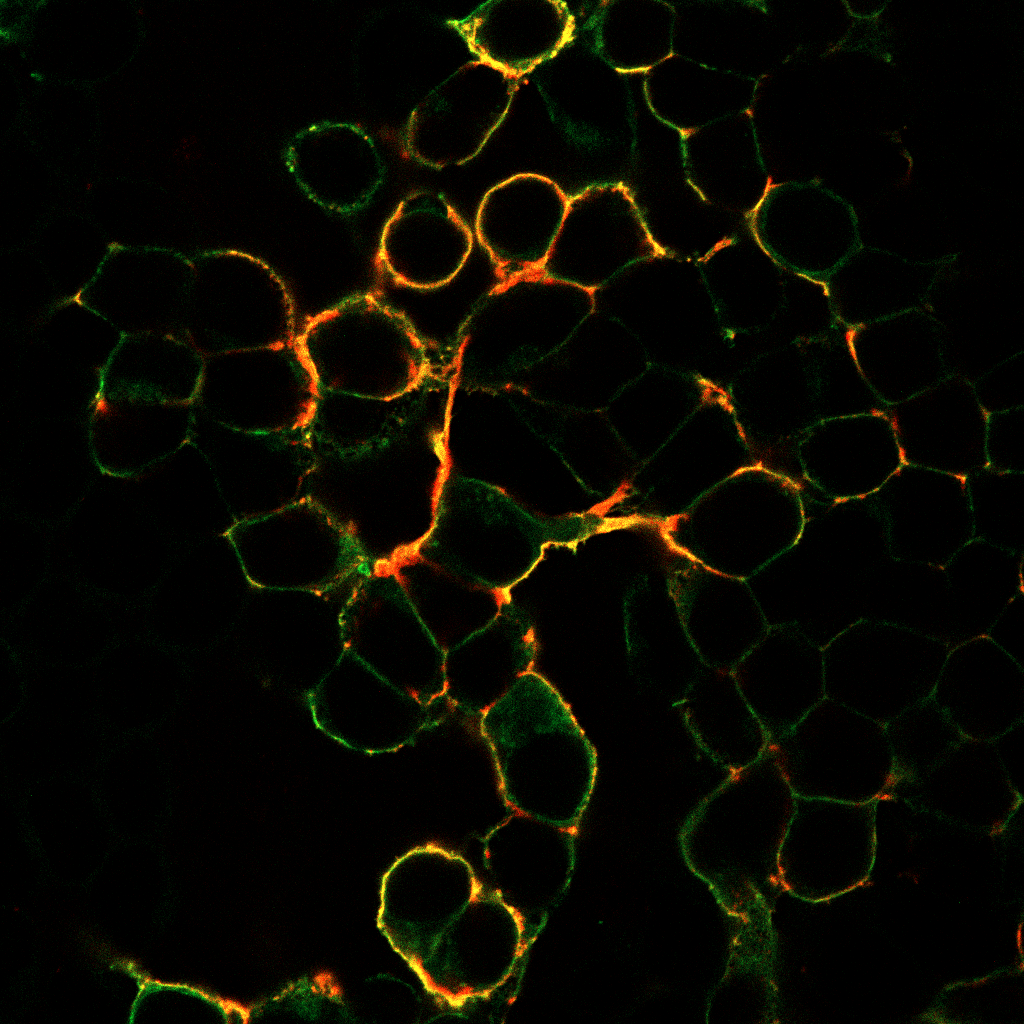

Supplement: Supplementary file 15 — Source data Fig. 1 [file 44321_2026_402_MOESM15_ESM.zip › Panel K and L/Figure 1K. Ig-Nlgn2, MDGA1 WT/Ig-Nlgn2, MDGA1 WT (2).tif]

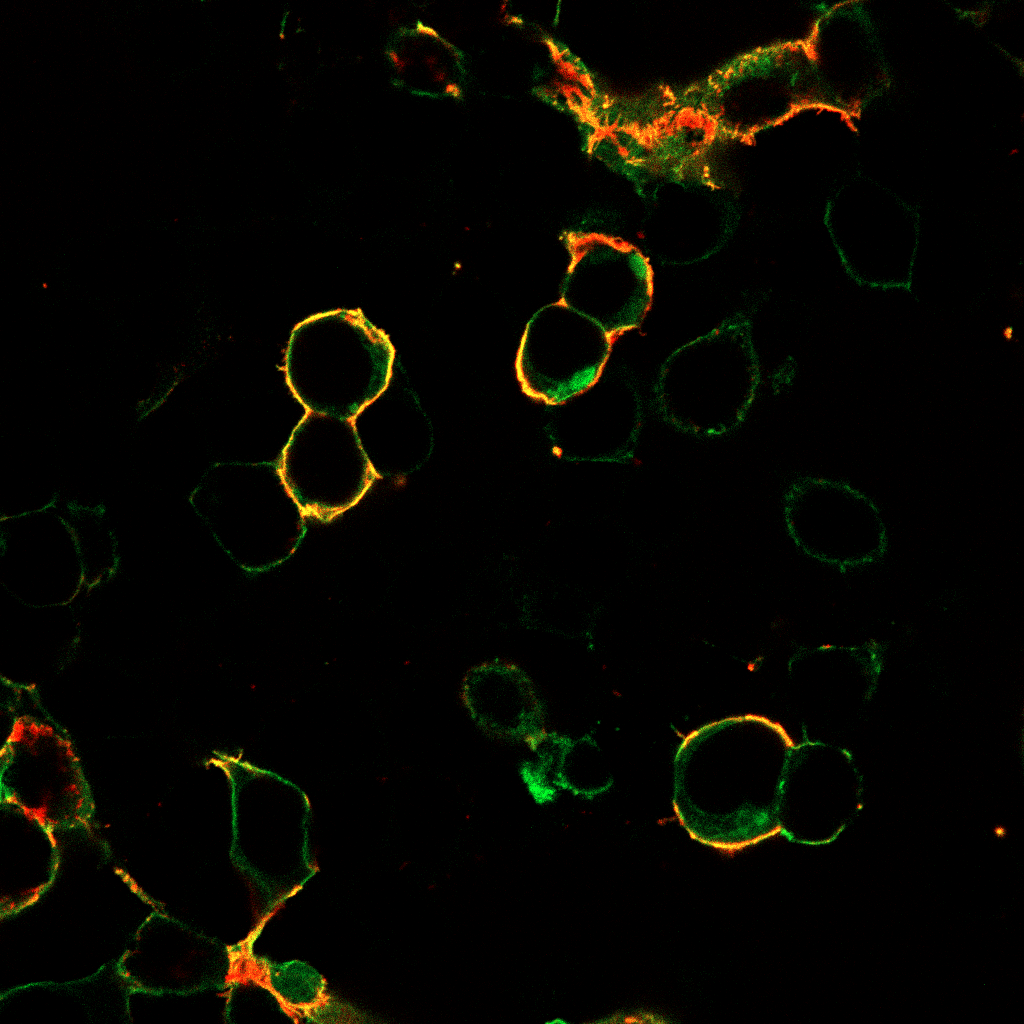

Supplement: Supplementary file 15 — Source data Fig. 1 [file 44321_2026_402_MOESM15_ESM.zip › Panel K and L/Figure 1K. Ig-Nlgn2, MDGA1 WT/Ig-Nlgn2, MDGA1 WT (3).tif]

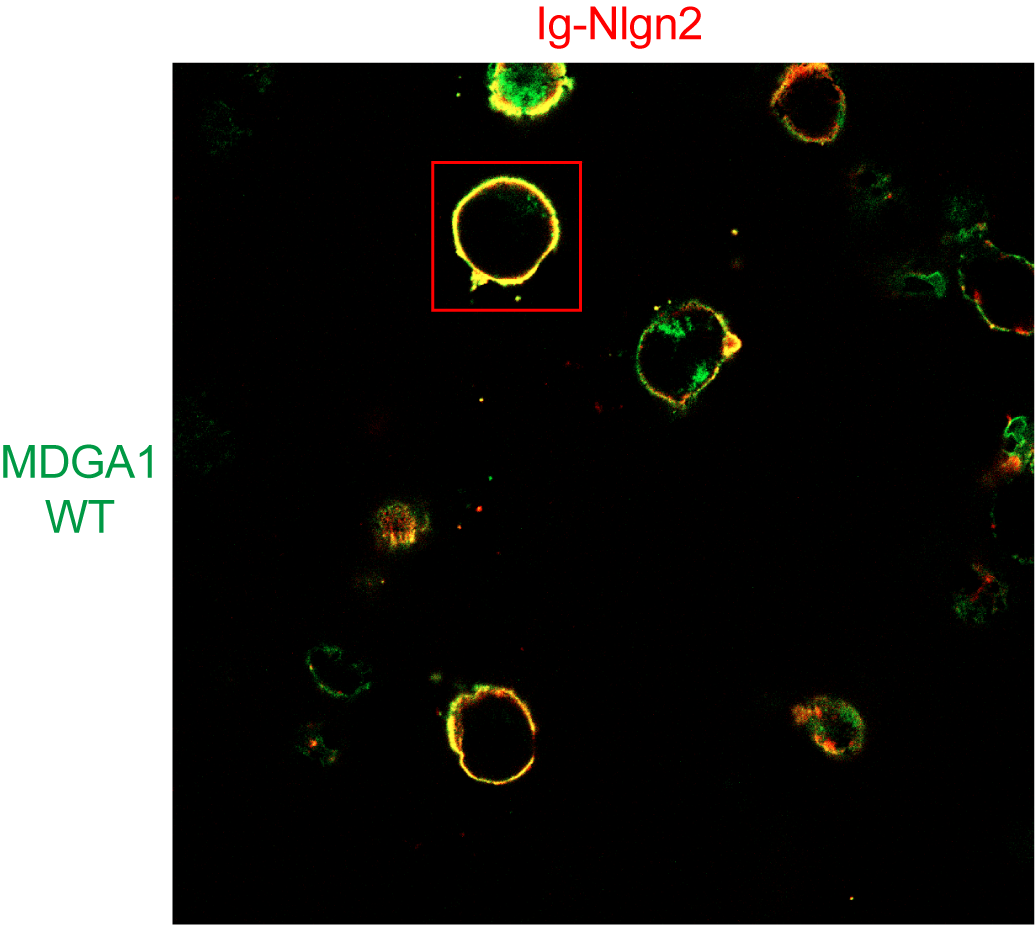

Supplement: Supplementary file 15 — Source data Fig. 1 [file 44321_2026_402_MOESM15_ESM.zip › Panel K and L/Figure 1K. Ig-Nlgn2, MDGA1 WT.tif]

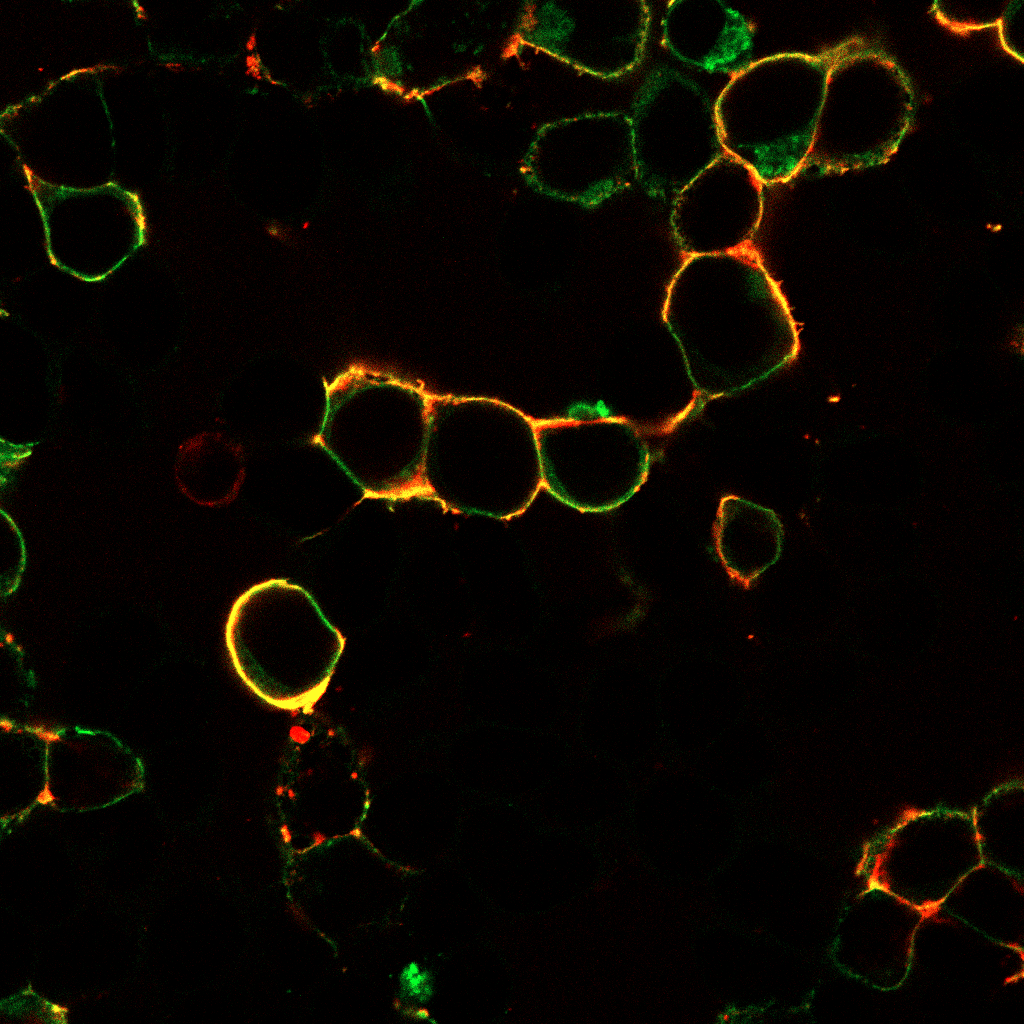

Supplement: Supplementary file 15 — Source data Fig. 1 [file 44321_2026_402_MOESM15_ESM.zip › Panel K and L/Figure 1K. Ig-Nlgn2, MDGA1 Y635C, E756Q/Ig-Nlgn2, MDGA1 Y635C, E756Q (1).tif]

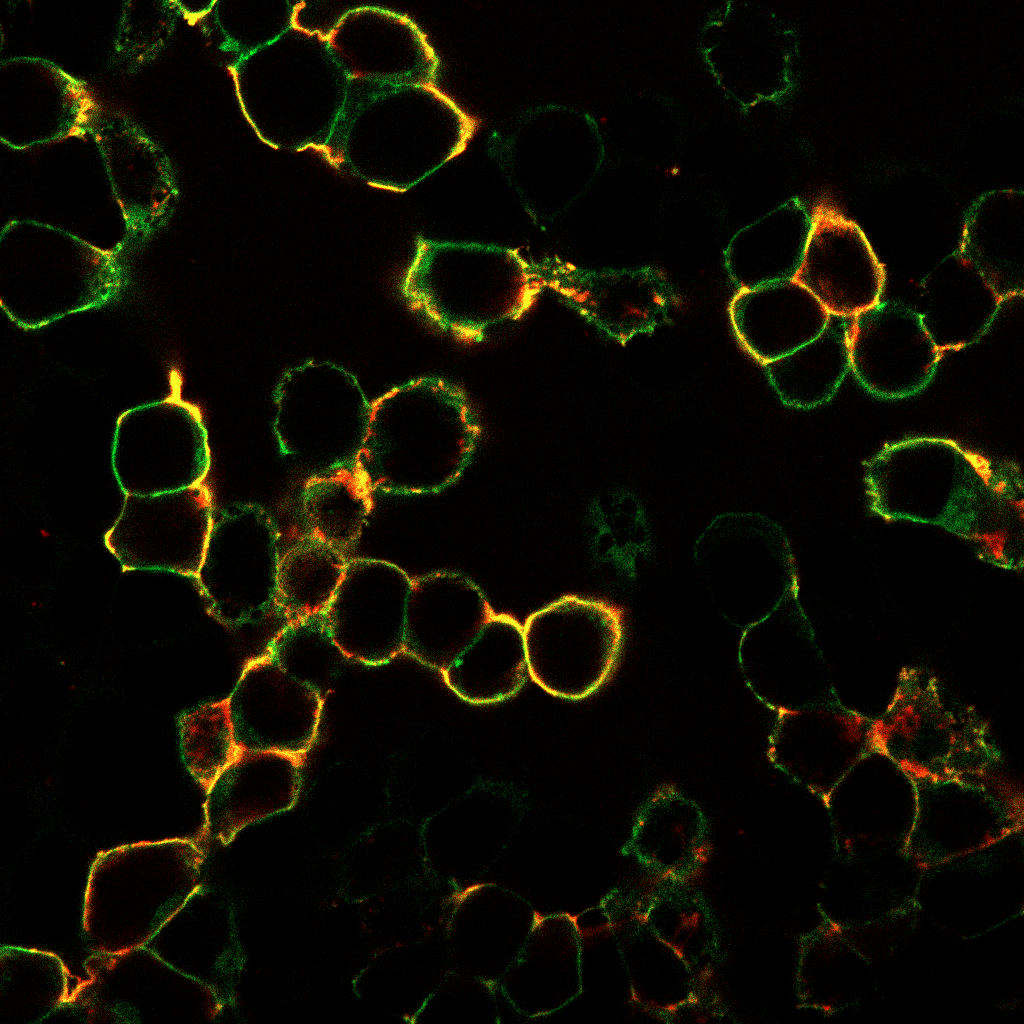

Supplement: Supplementary file 15 — Source data Fig. 1 [file 44321_2026_402_MOESM15_ESM.zip › Panel K and L/Figure 1K. Ig-Nlgn2, MDGA1 Y635C, E756Q/Ig-Nlgn2, MDGA1 Y635C, E756Q (2).tif]

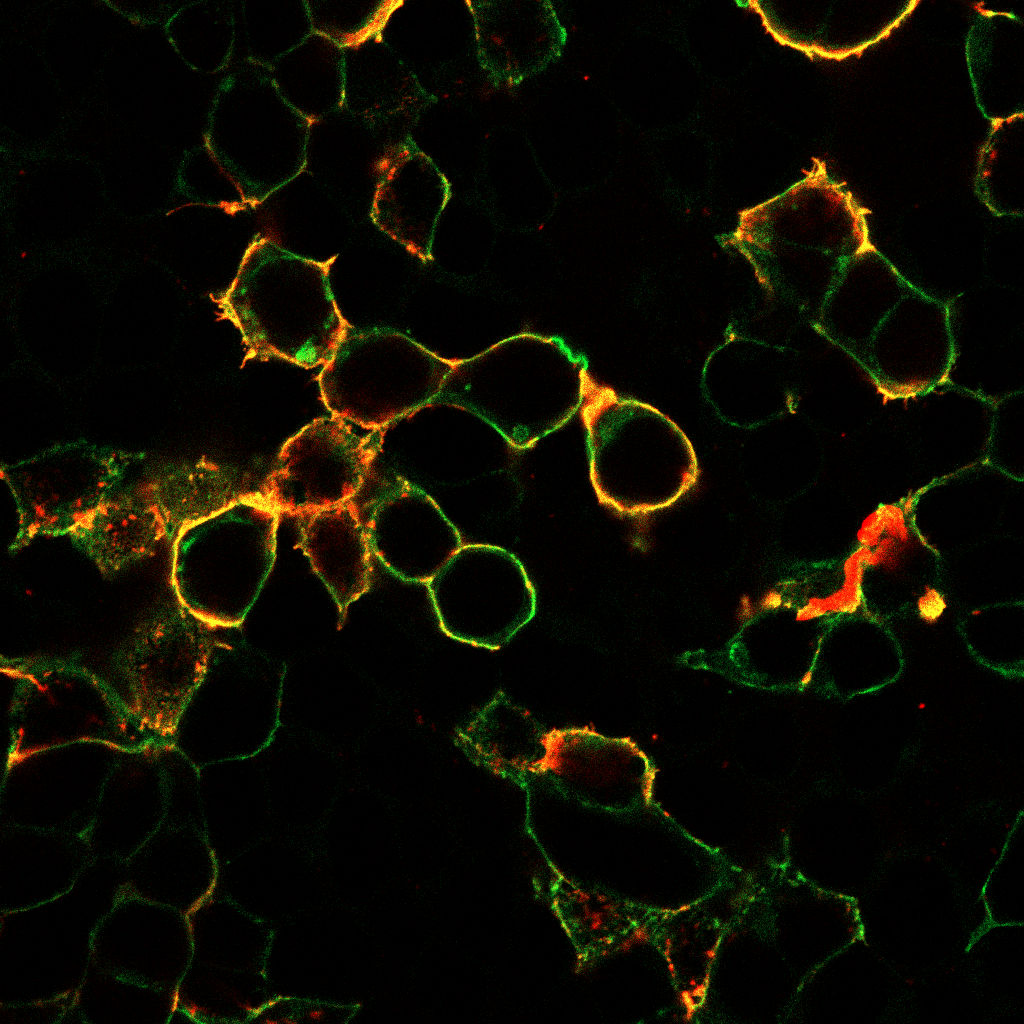

Supplement: Supplementary file 15 — Source data Fig. 1 [file 44321_2026_402_MOESM15_ESM.zip › Panel K and L/Figure 1K. Ig-Nlgn2, MDGA1 Y635C, E756Q/Ig-Nlgn2, MDGA1 Y635C, E756Q (3).tif]

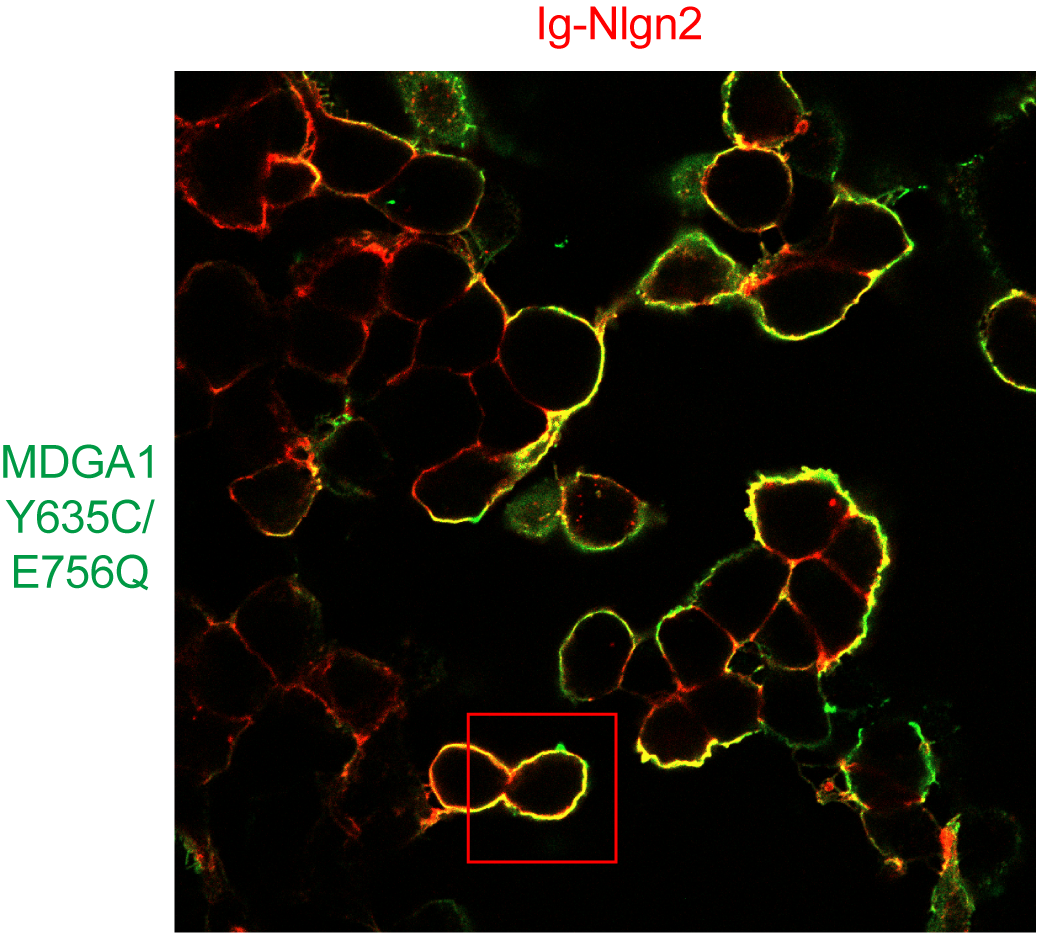

Supplement: Supplementary file 15 — Source data Fig. 1 [file 44321_2026_402_MOESM15_ESM.zip › Panel K and L/Figure 1K. Ig-Nlgn2, MDGA1 Y635C, E756Q.tif]

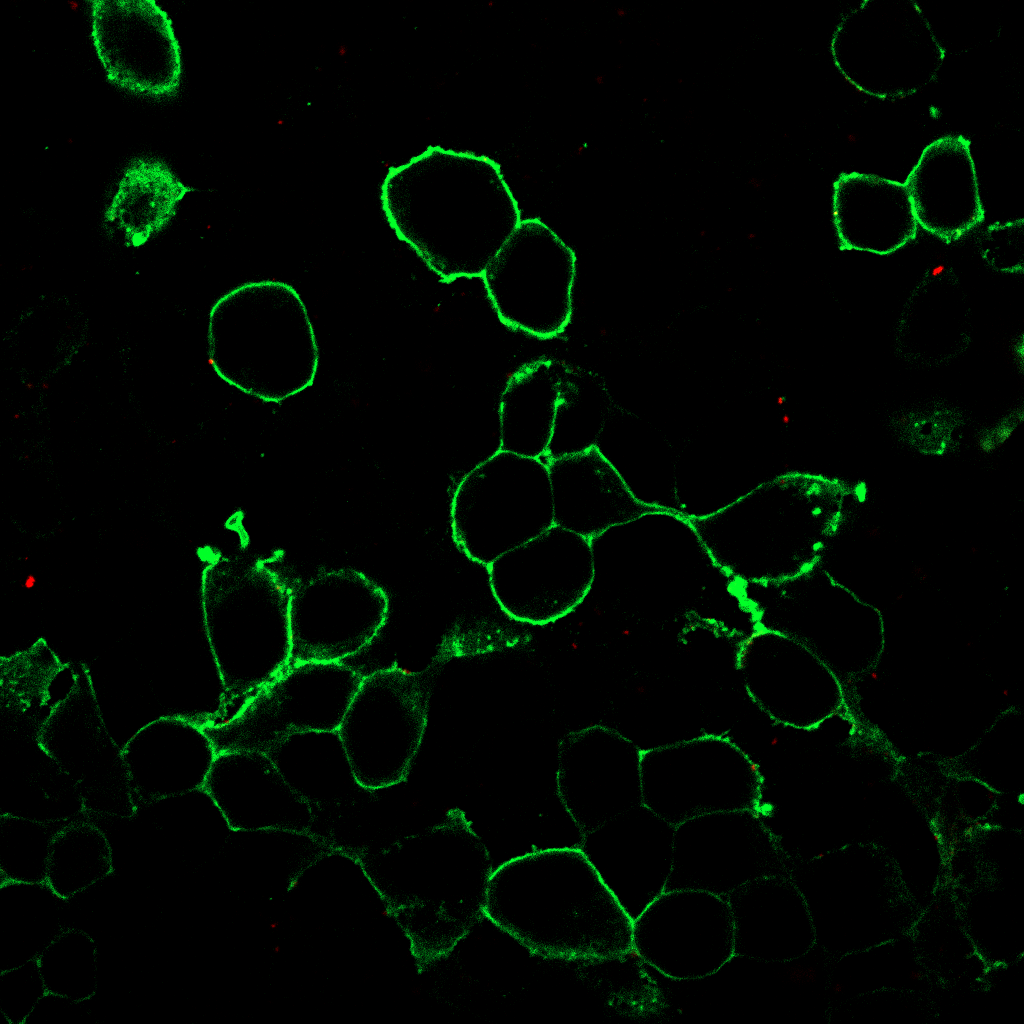

Supplement: Supplementary file 15 — Source data Fig. 1 [file 44321_2026_402_MOESM15_ESM.zip › Panel K and L/Figure 1K. IgC, MDGA1 V116M, A688V/IgC, MDGA1 V116M, A688V (1).tif]

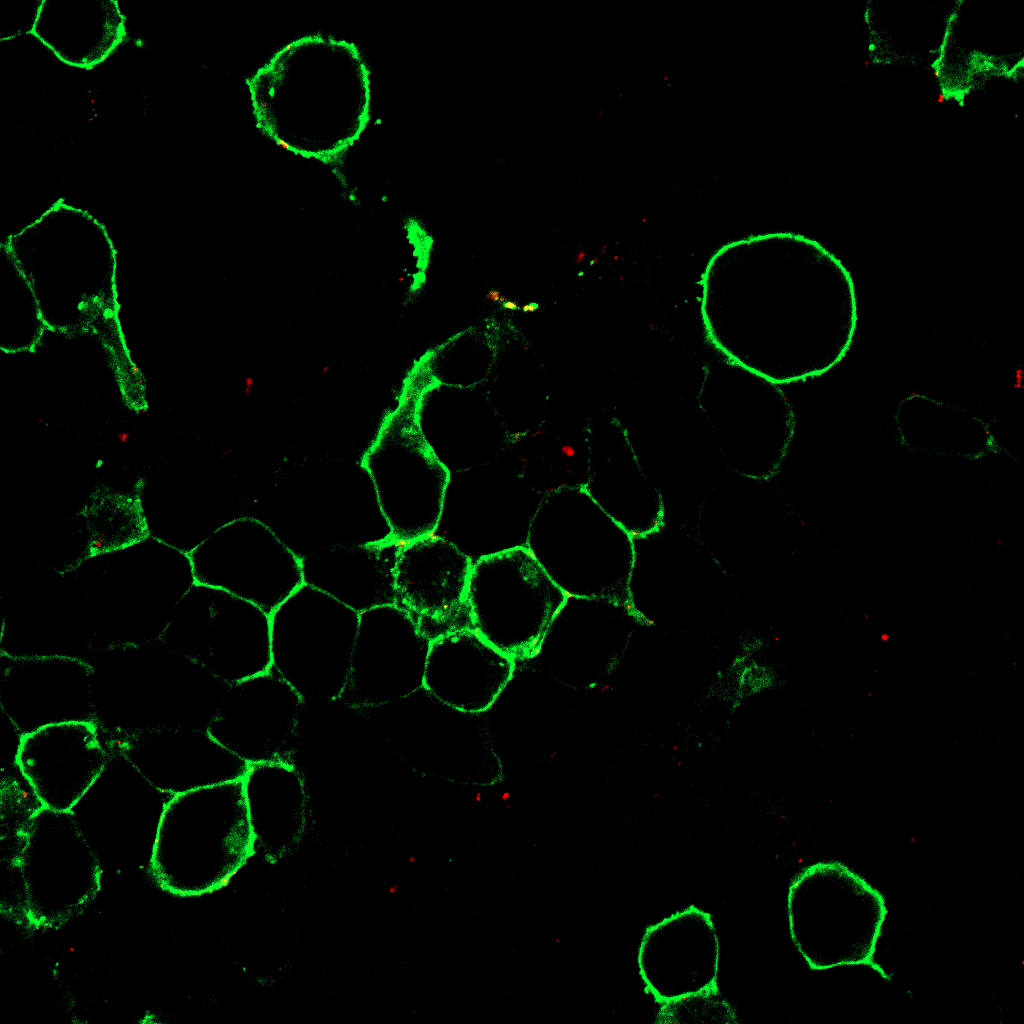

Supplement: Supplementary file 15 — Source data Fig. 1 [file 44321_2026_402_MOESM15_ESM.zip › Panel K and L/Figure 1K. IgC, MDGA1 V116M, A688V/IgC, MDGA1 V116M, A688V (2).tif]

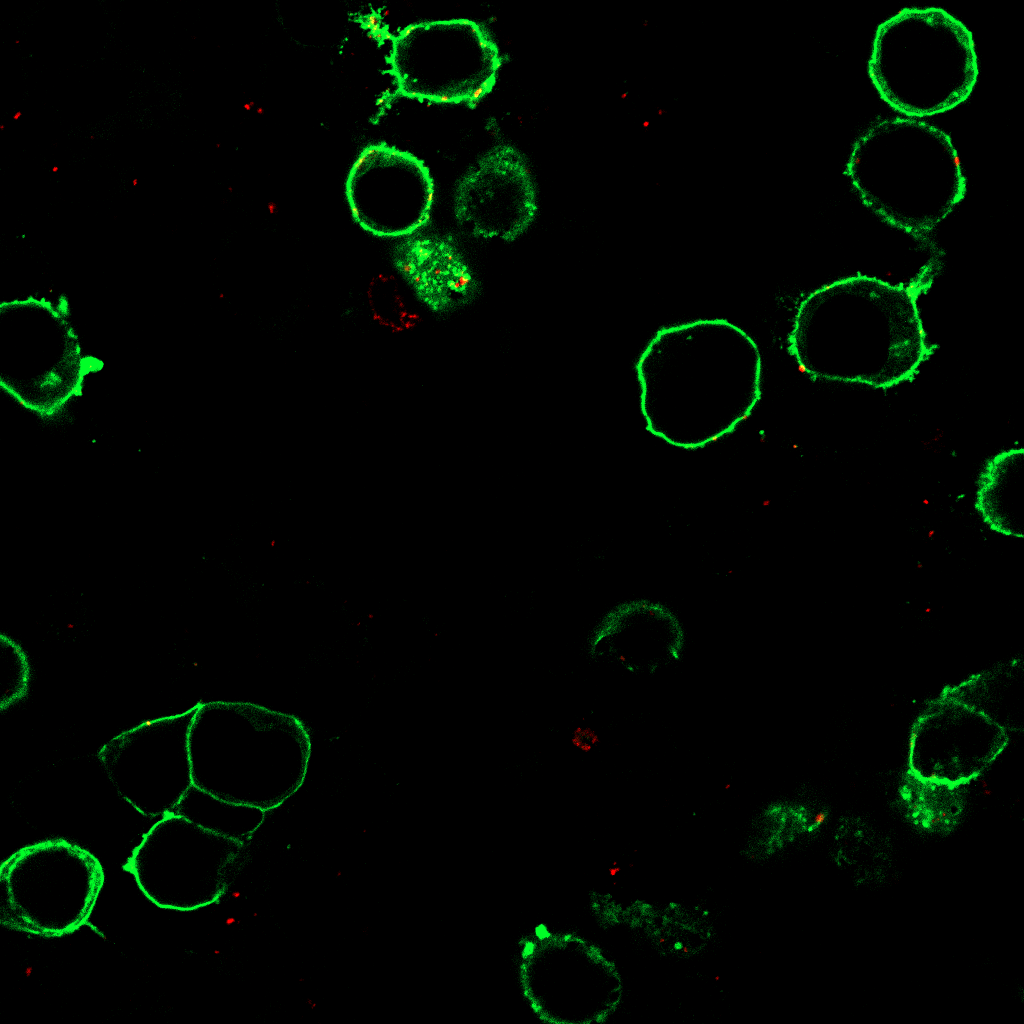

Supplement: Supplementary file 15 — Source data Fig. 1 [file 44321_2026_402_MOESM15_ESM.zip › Panel K and L/Figure 1K. IgC, MDGA1 V116M, A688V/IgC, MDGA1 V116M, A688V (3).tif]

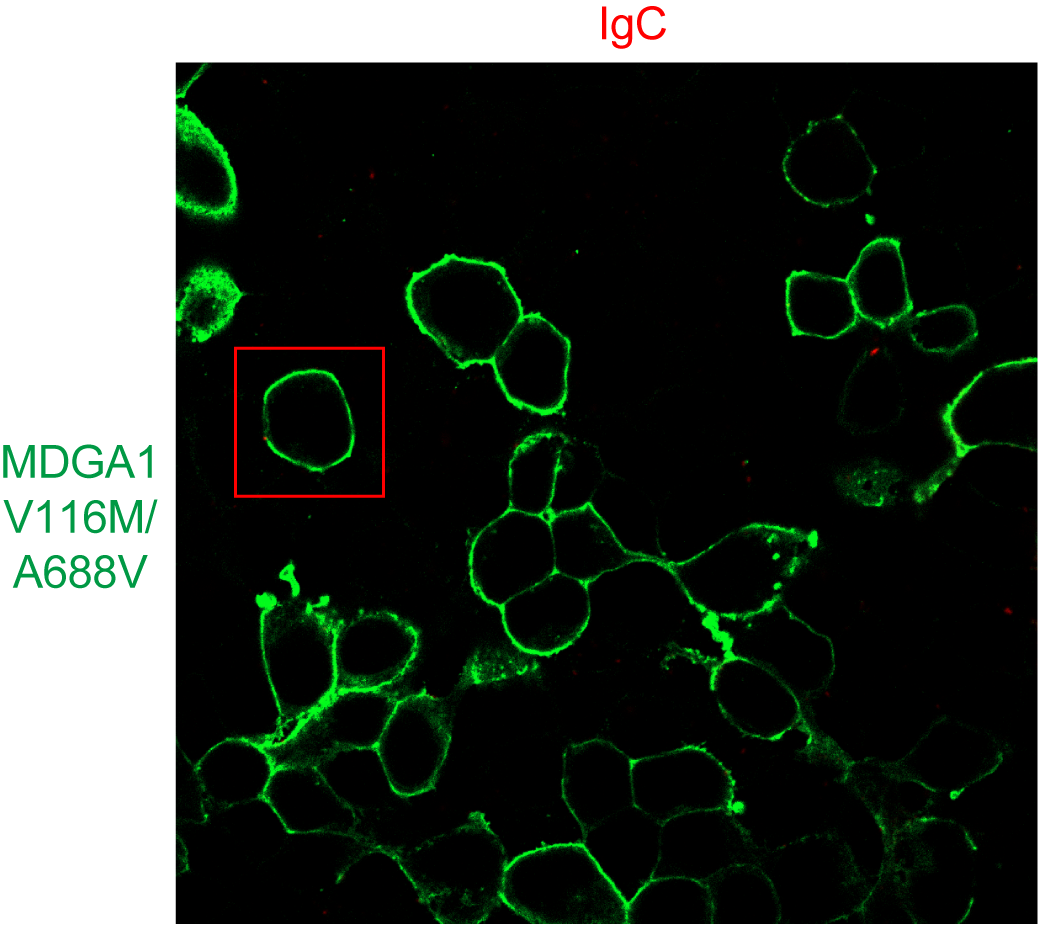

Supplement: Supplementary file 15 — Source data Fig. 1 [file 44321_2026_402_MOESM15_ESM.zip › Panel K and L/Figure 1K. IgC, MDGA1 V116M, A688V.tif]

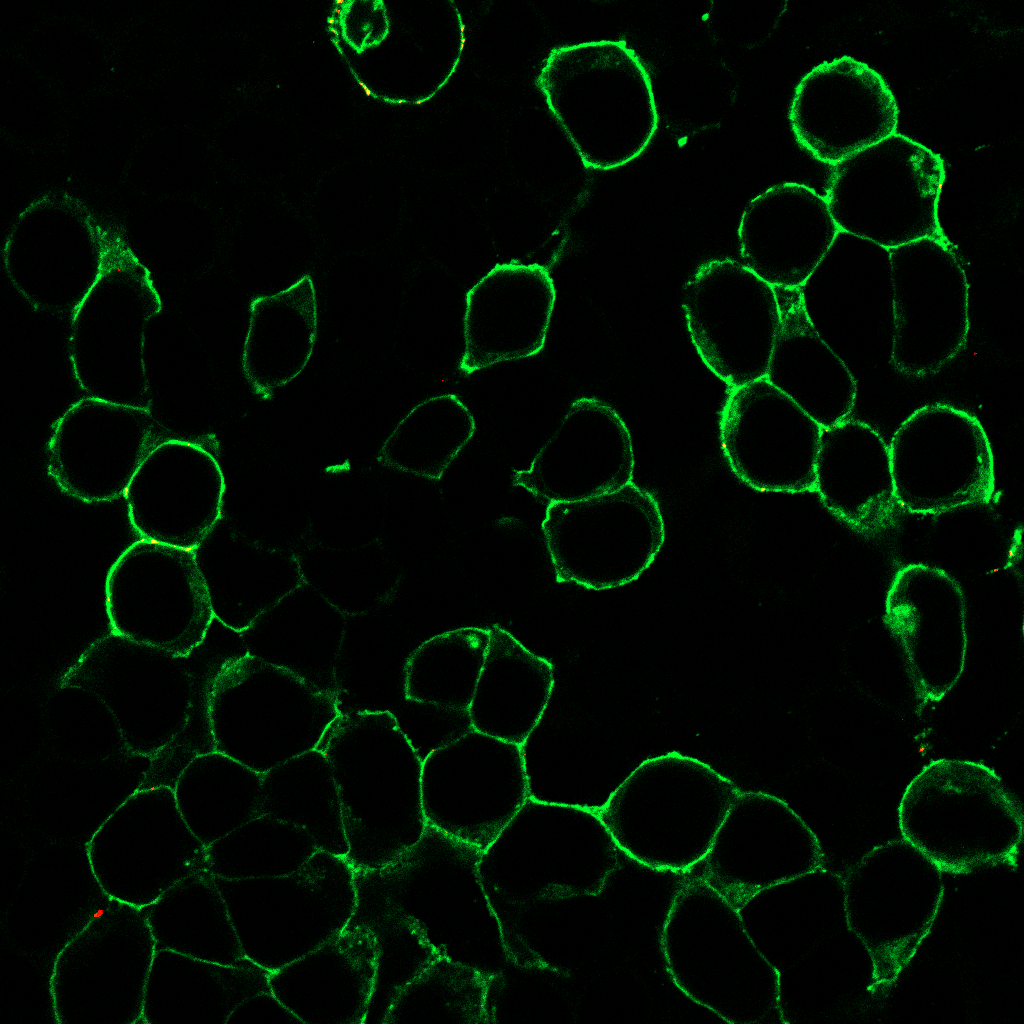

Supplement: Supplementary file 15 — Source data Fig. 1 [file 44321_2026_402_MOESM15_ESM.zip › Panel K and L/Figure 1K. IgC, MDGA1 WT/IgC, MDGA1 WT (1).tif]

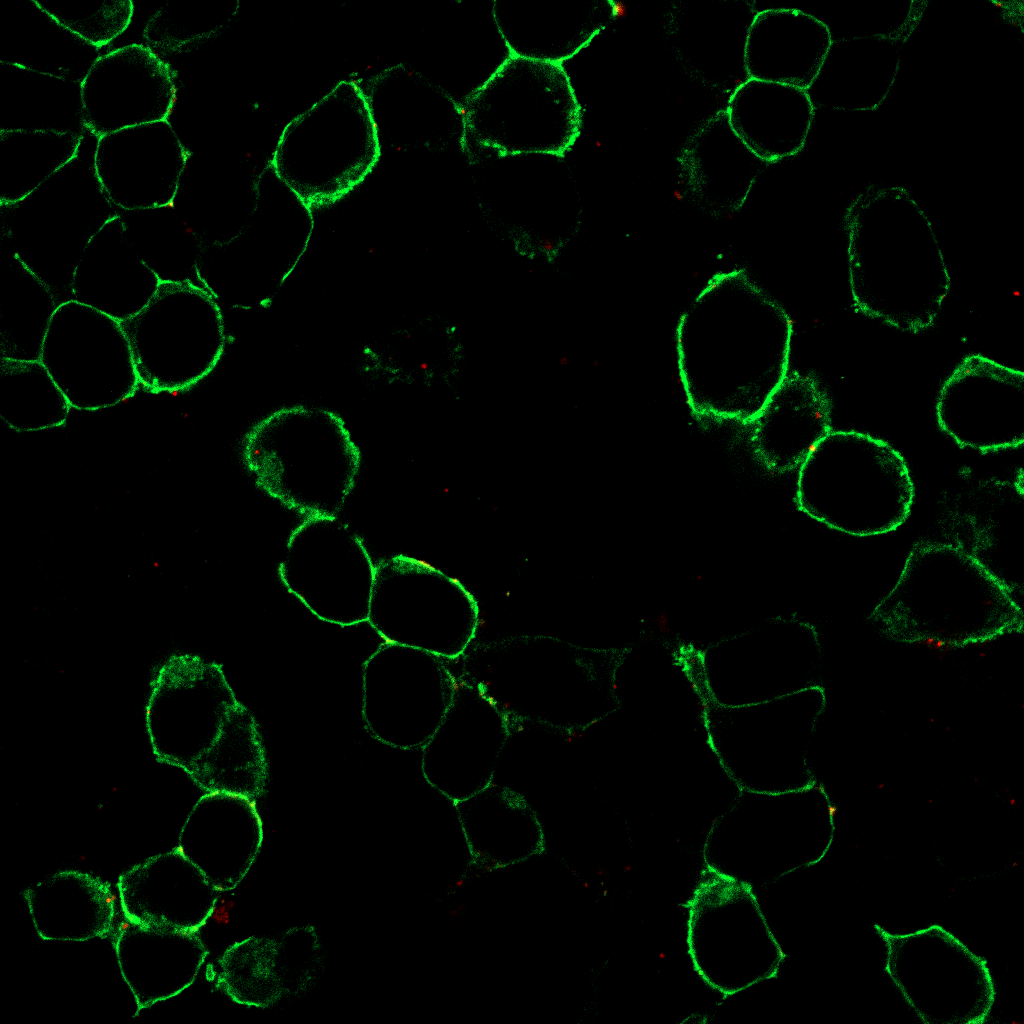

Supplement: Supplementary file 15 — Source data Fig. 1 [file 44321_2026_402_MOESM15_ESM.zip › Panel K and L/Figure 1K. IgC, MDGA1 WT/IgC, MDGA1 WT (2).tif]

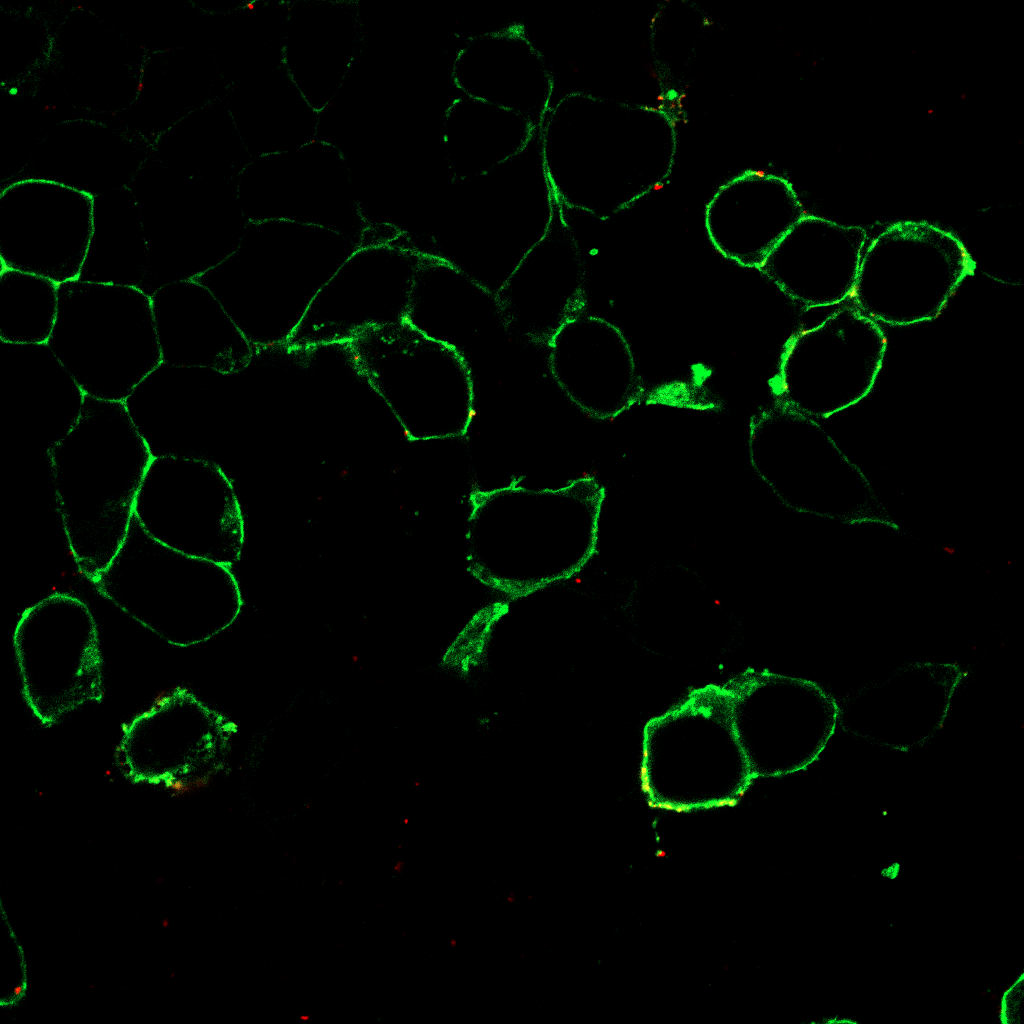

Supplement: Supplementary file 15 — Source data Fig. 1 [file 44321_2026_402_MOESM15_ESM.zip › Panel K and L/Figure 1K. IgC, MDGA1 WT/IgC, MDGA1 WT (3).tif]

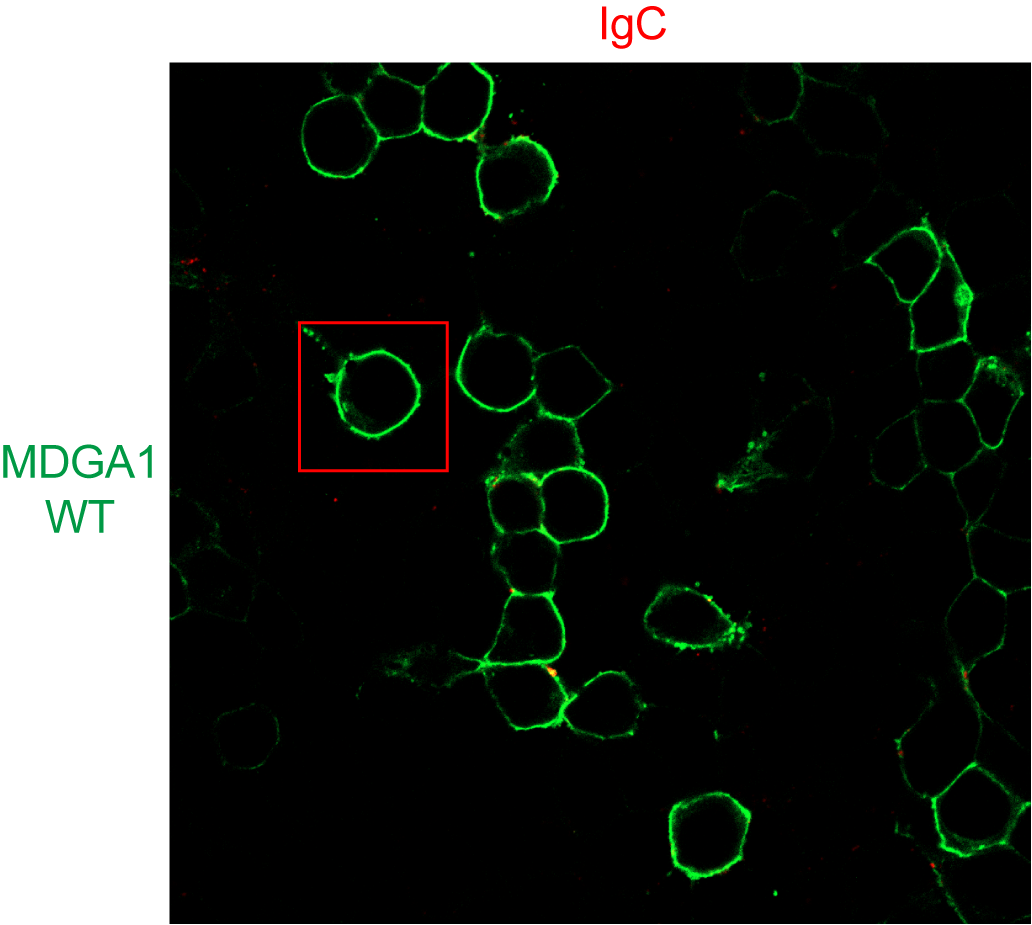

Supplement: Supplementary file 15 — Source data Fig. 1 [file 44321_2026_402_MOESM15_ESM.zip › Panel K and L/Figure 1K. IgC, MDGA1 WT.tif]

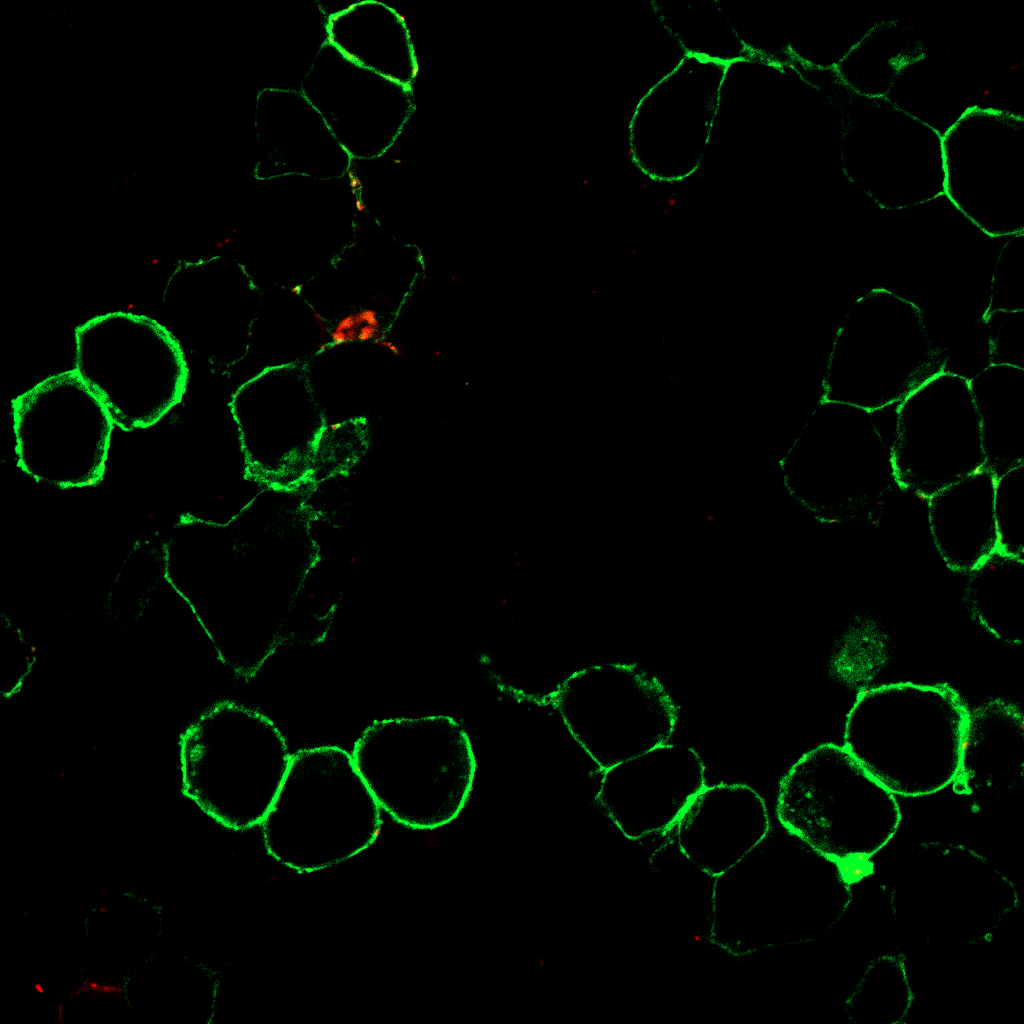

Supplement: Supplementary file 15 — Source data Fig. 1 [file 44321_2026_402_MOESM15_ESM.zip › Panel K and L/Figure 1K. IgC, MDGA1 Y635C, E756Q/IgC, MDGA1 Y635C, E756Q (1).tif]

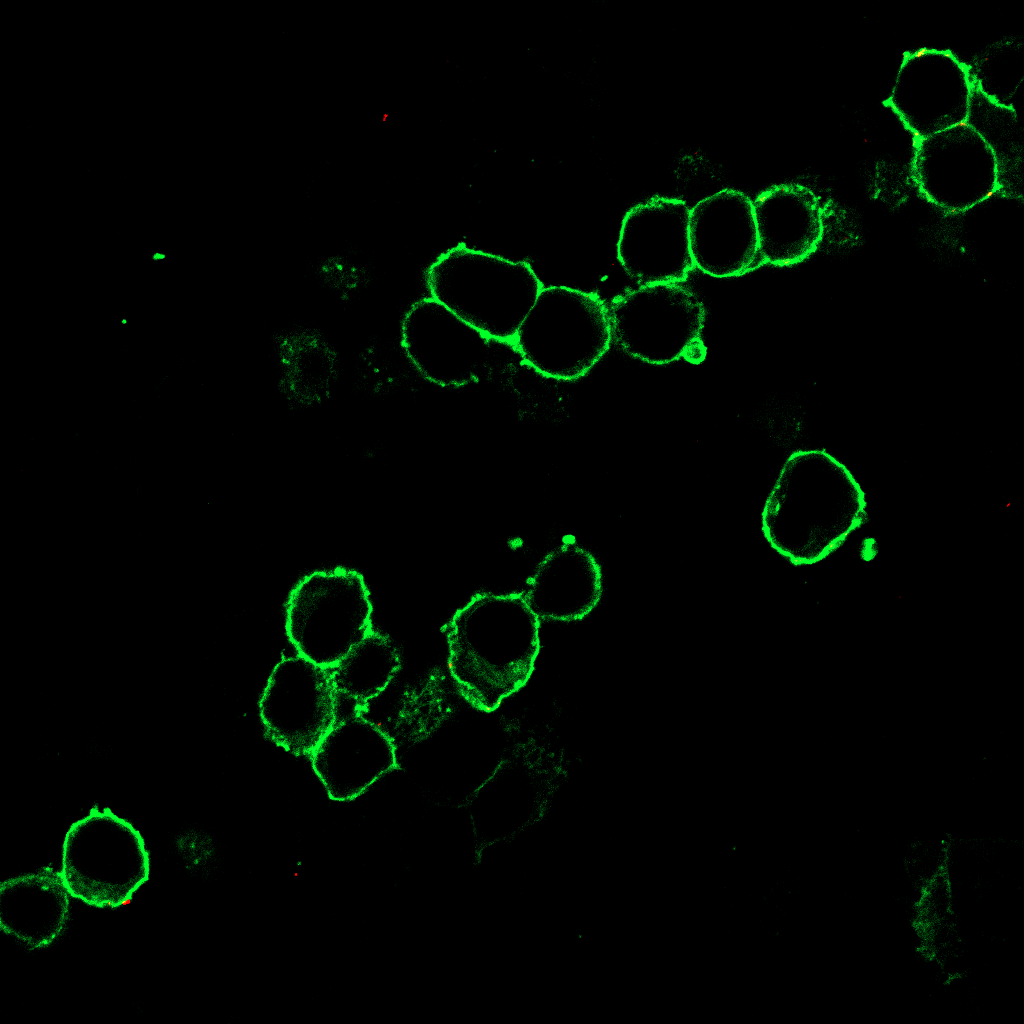

Supplement: Supplementary file 15 — Source data Fig. 1 [file 44321_2026_402_MOESM15_ESM.zip › Panel K and L/Figure 1K. IgC, MDGA1 Y635C, E756Q/IgC, MDGA1 Y635C, E756Q (2).tif]

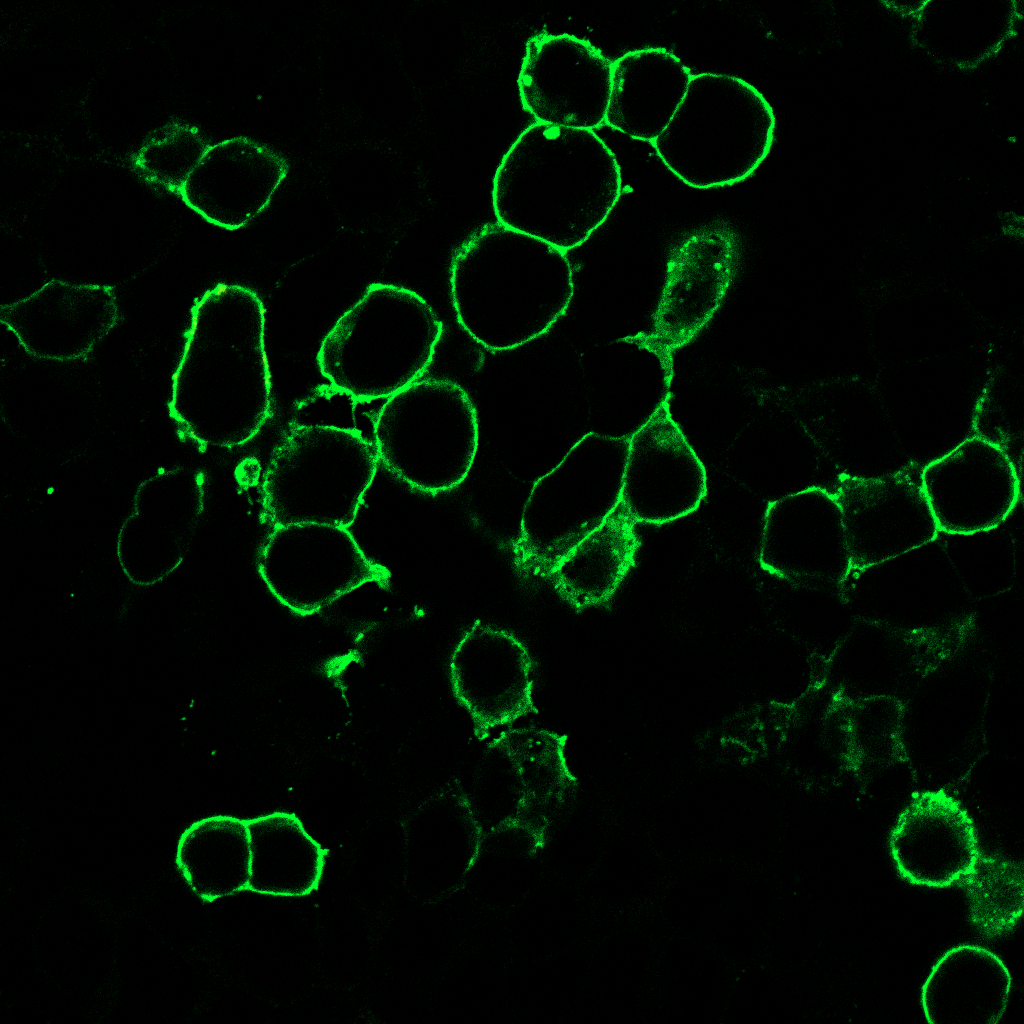

Supplement: Supplementary file 15 — Source data Fig. 1 [file 44321_2026_402_MOESM15_ESM.zip › Panel K and L/Figure 1K. IgC, MDGA1 Y635C, E756Q/IgC, MDGA1 Y635C, E756Q (3).tif]

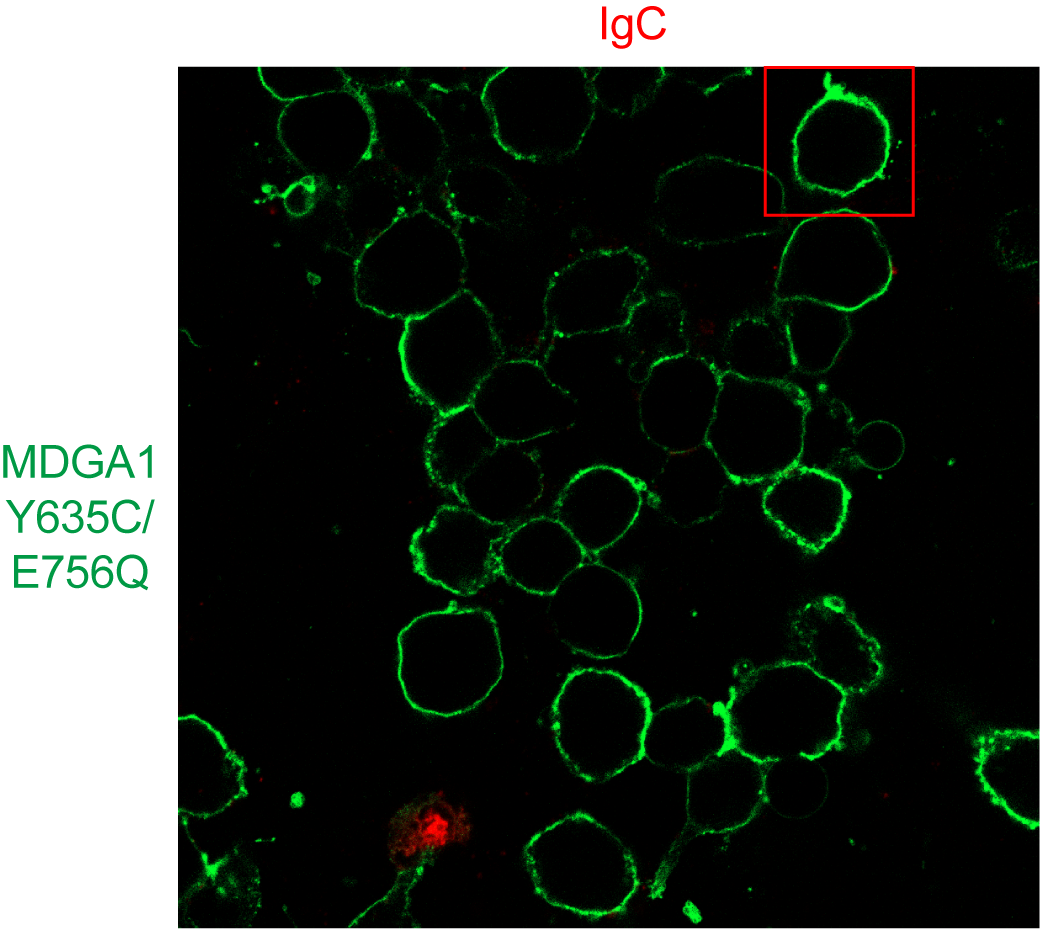

Supplement: Supplementary file 15 — Source data Fig. 1 [file 44321_2026_402_MOESM15_ESM.zip › Panel K and L/Figure 1K. IgC, MDGA1 Y635C, E756Q.tif]

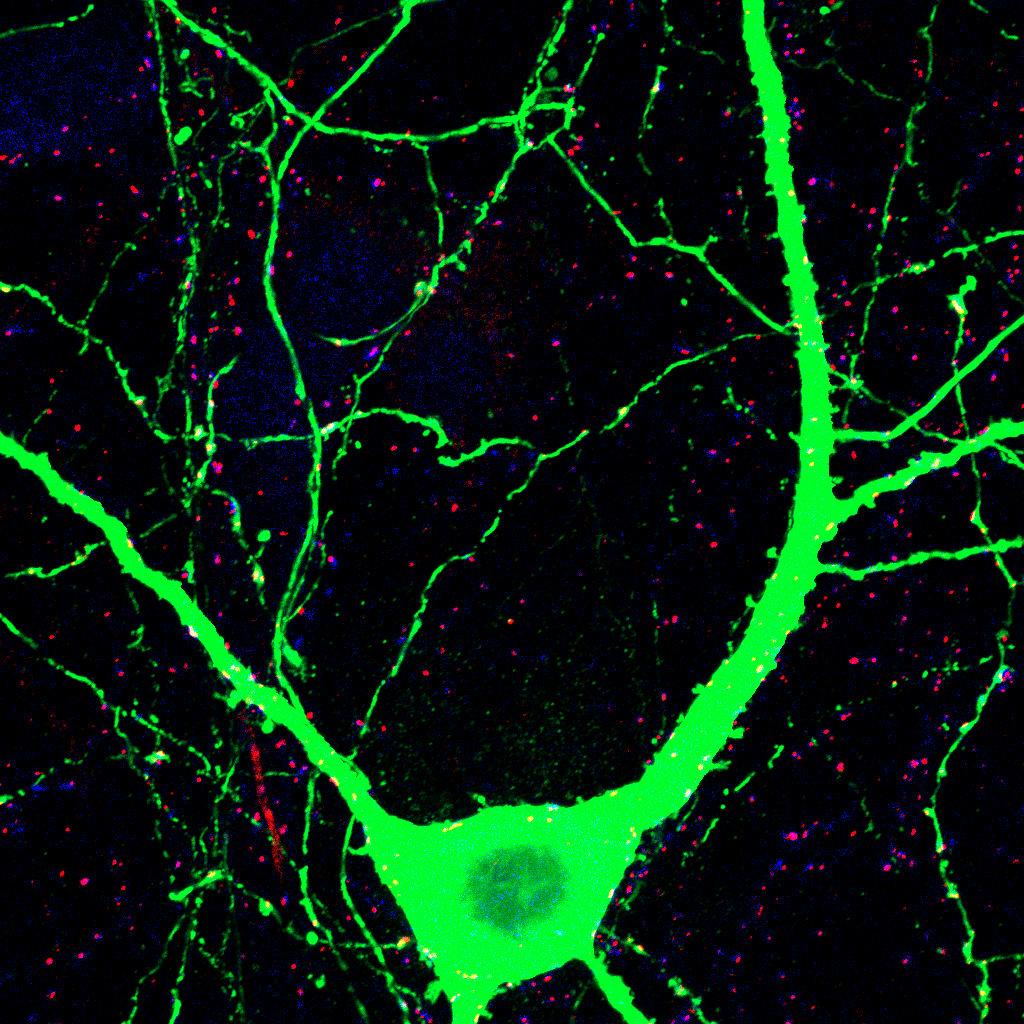

Supplement: Supplementary file 16 — Source data Fig. 2 [file 44321_2026_402_MOESM16_ESM.zip › Panel A and B/Control/Control (1).tif]

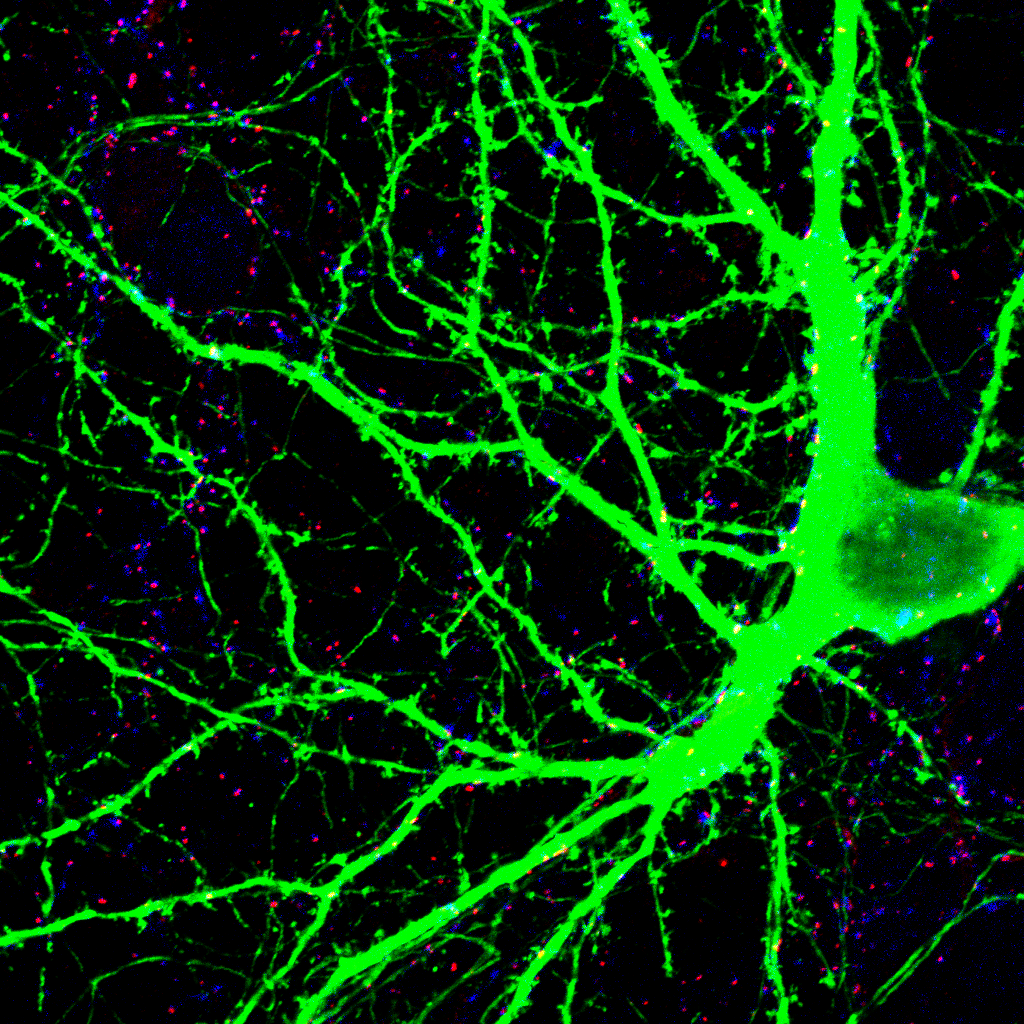

Supplement: Supplementary file 16 — Source data Fig. 2 [file 44321_2026_402_MOESM16_ESM.zip › Panel A and B/Control/Control (10).tif]

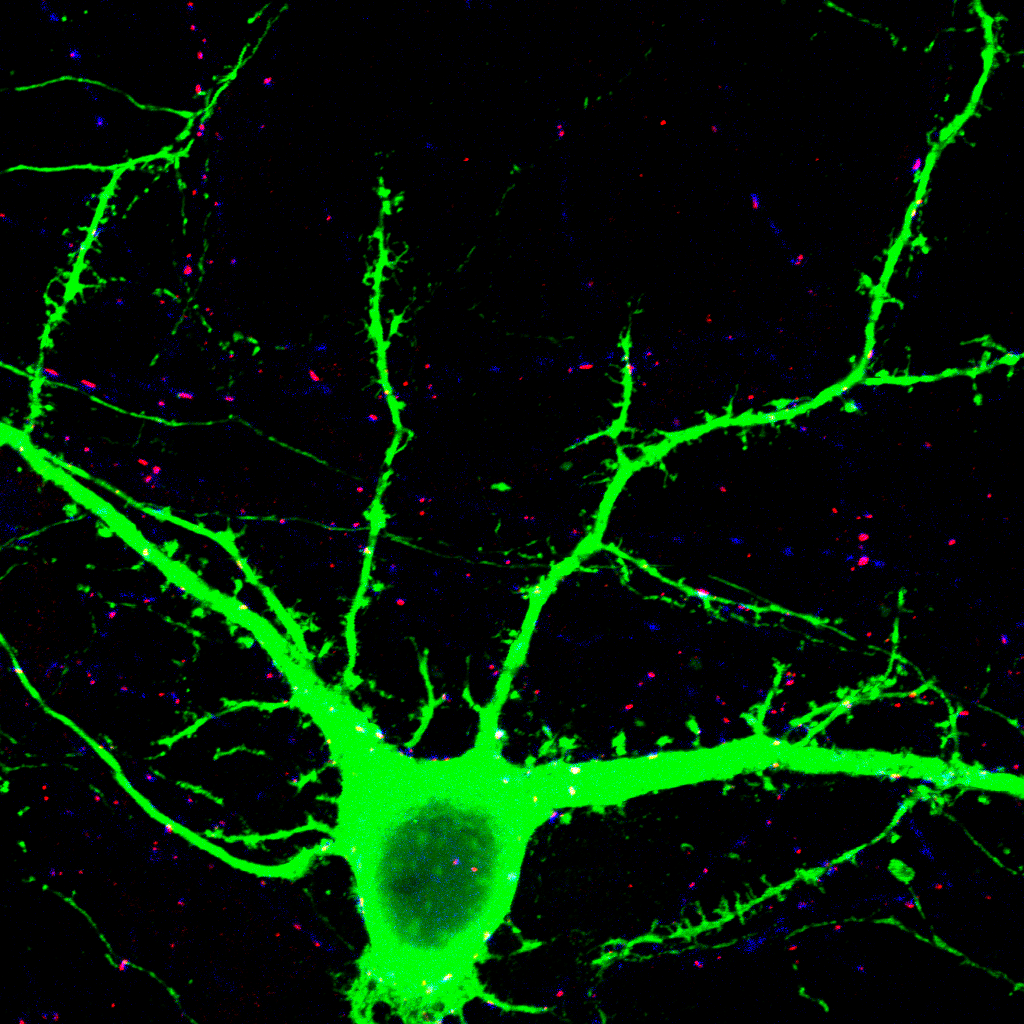

Supplement: Supplementary file 16 — Source data Fig. 2 [file 44321_2026_402_MOESM16_ESM.zip › Panel A and B/Control/Control (11).tif]

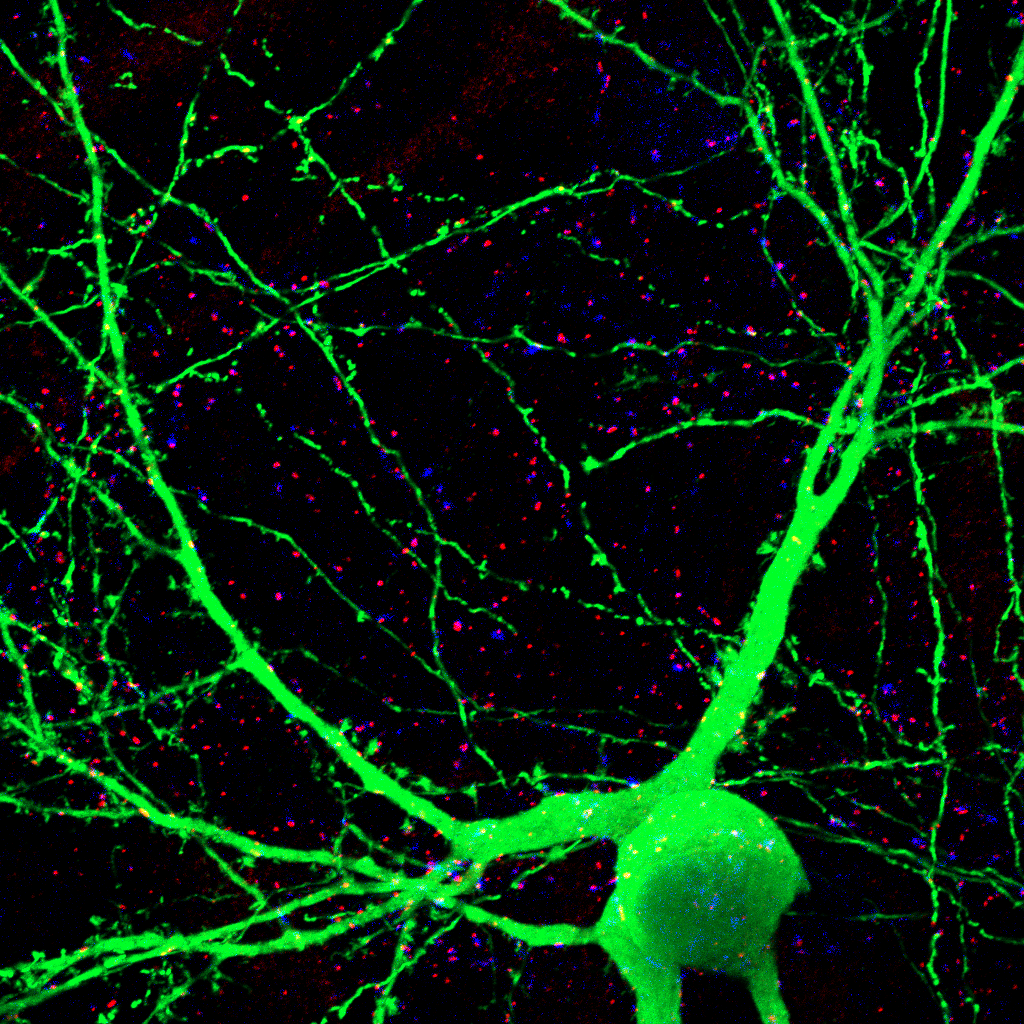

Supplement: Supplementary file 16 — Source data Fig. 2 [file 44321_2026_402_MOESM16_ESM.zip › Panel A and B/Control/Control (12).tif]

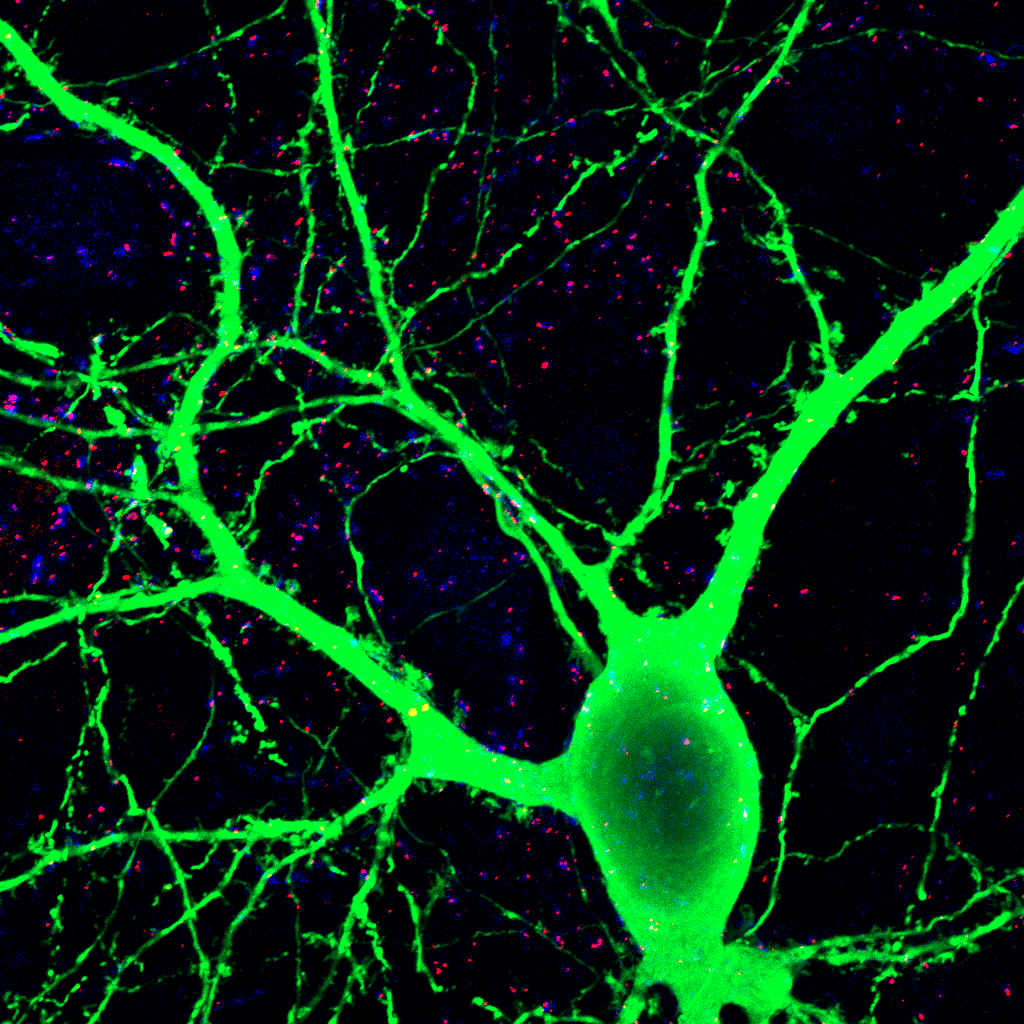

Supplement: Supplementary file 16 — Source data Fig. 2 [file 44321_2026_402_MOESM16_ESM.zip › Panel A and B/Control/Control (13).tif]

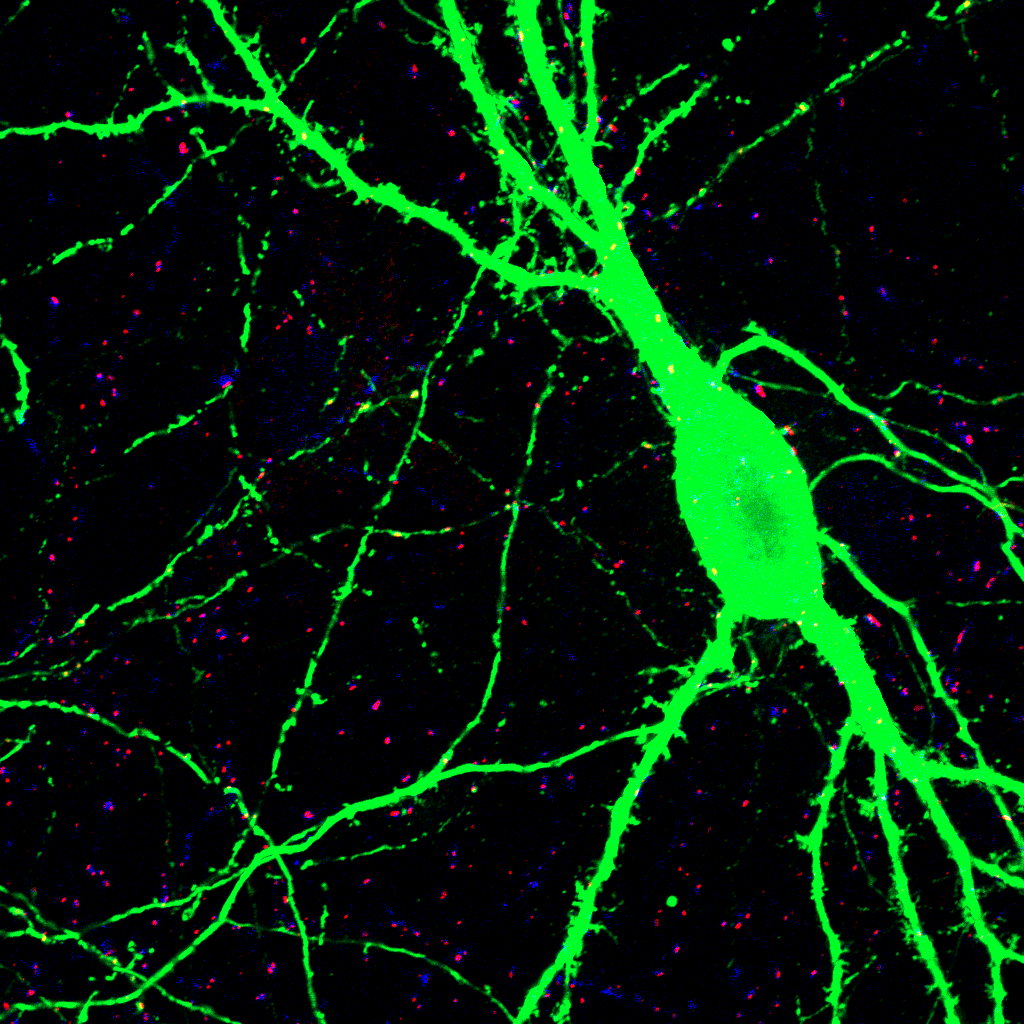

Supplement: Supplementary file 16 — Source data Fig. 2 [file 44321_2026_402_MOESM16_ESM.zip › Panel A and B/Control/Control (14).tif]

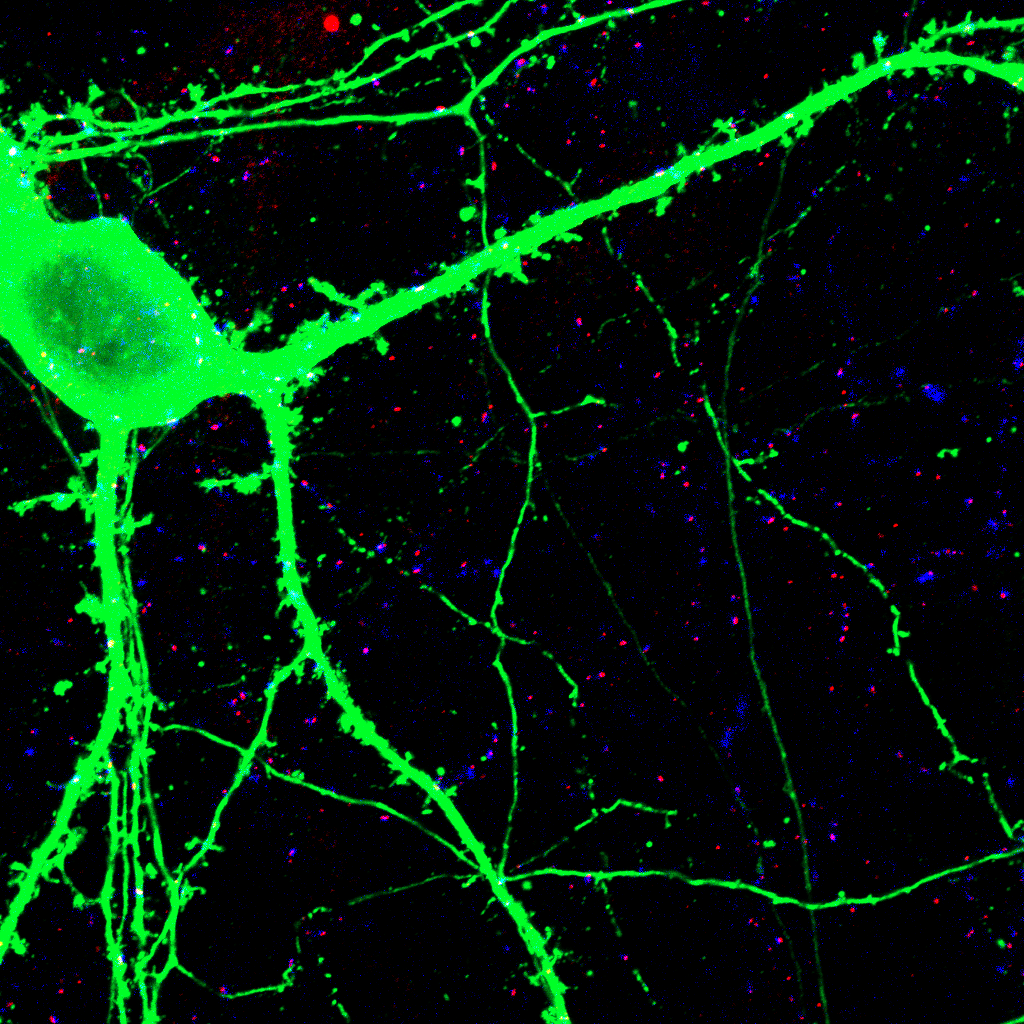

Supplement: Supplementary file 16 — Source data Fig. 2 [file 44321_2026_402_MOESM16_ESM.zip › Panel A and B/Control/Control (2).tif]

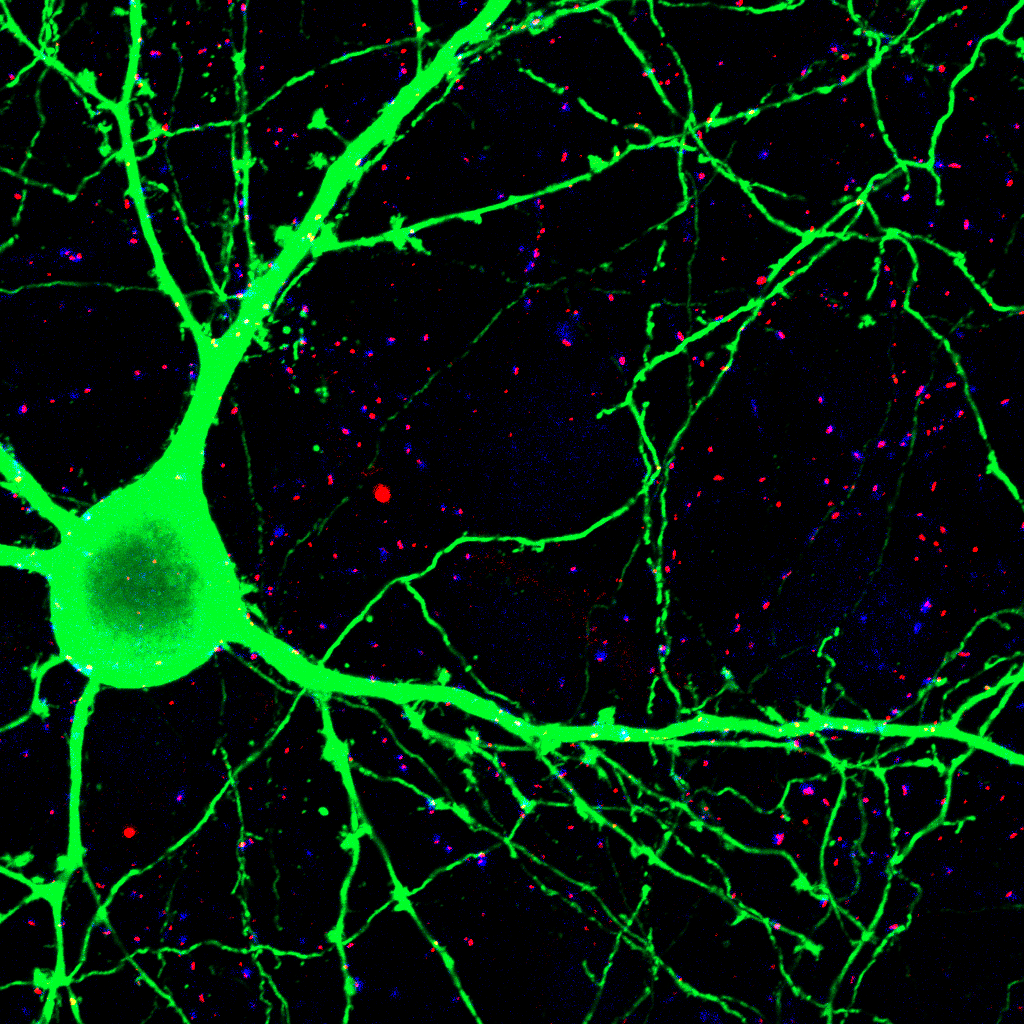

Supplement: Supplementary file 16 — Source data Fig. 2 [file 44321_2026_402_MOESM16_ESM.zip › Panel A and B/Control/Control (3).tif]

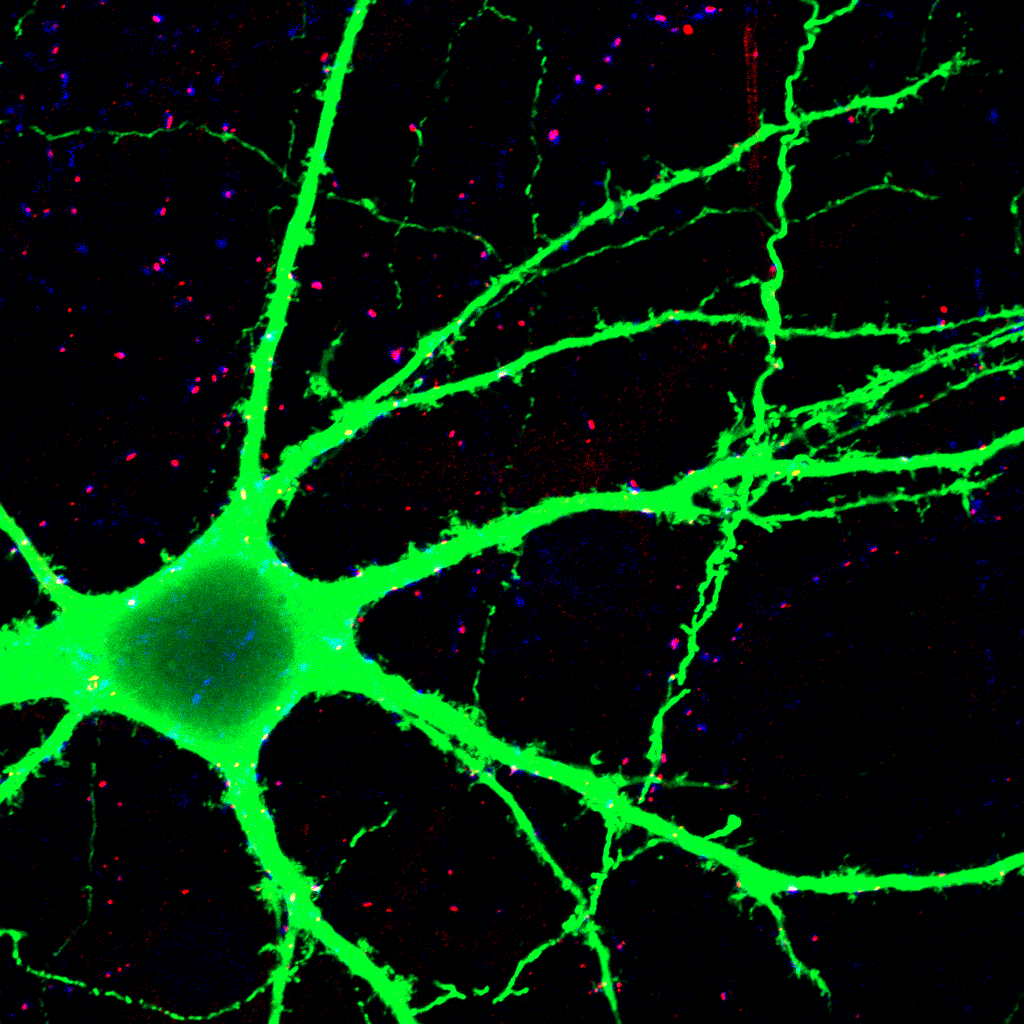

Supplement: Supplementary file 16 — Source data Fig. 2 [file 44321_2026_402_MOESM16_ESM.zip › Panel A and B/Control/Control (4).tif]

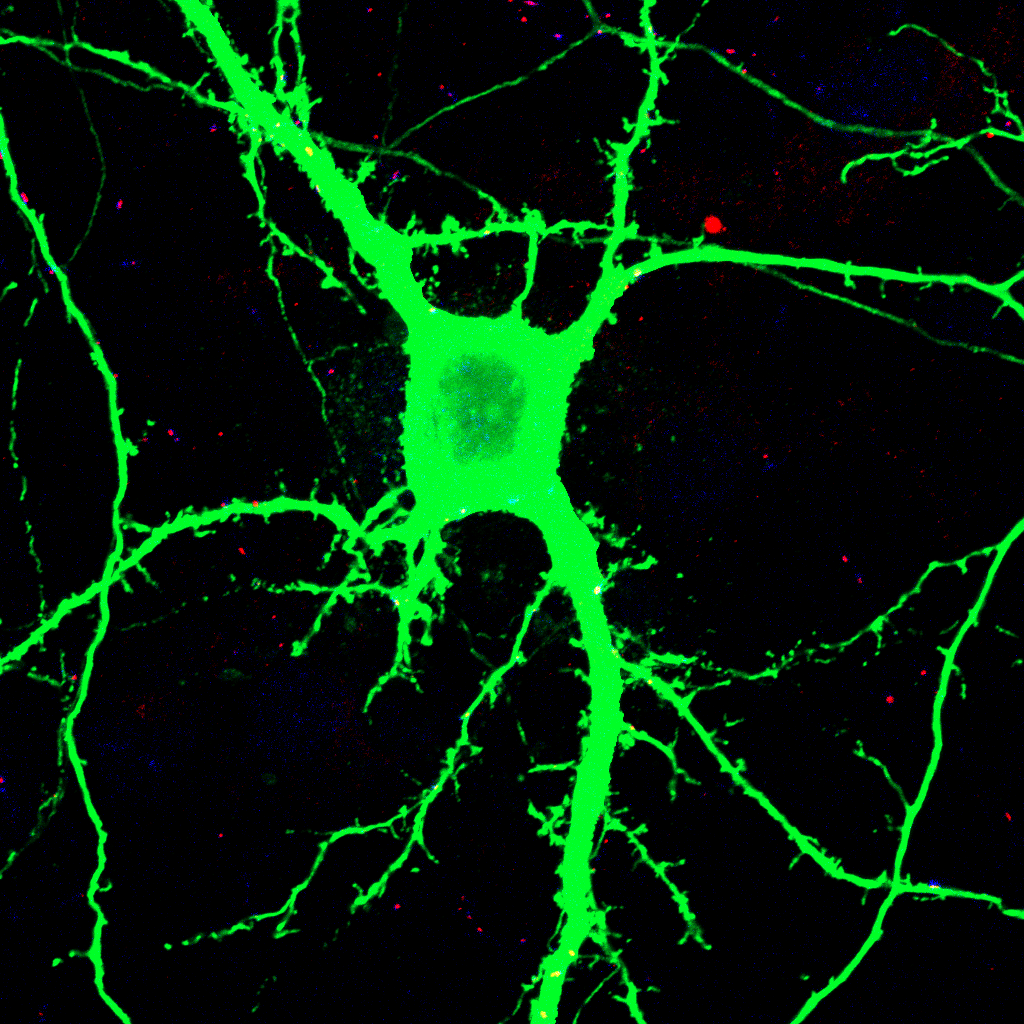

Supplement: Supplementary file 16 — Source data Fig. 2 [file 44321_2026_402_MOESM16_ESM.zip › Panel A and B/Control/Control (5).tif]

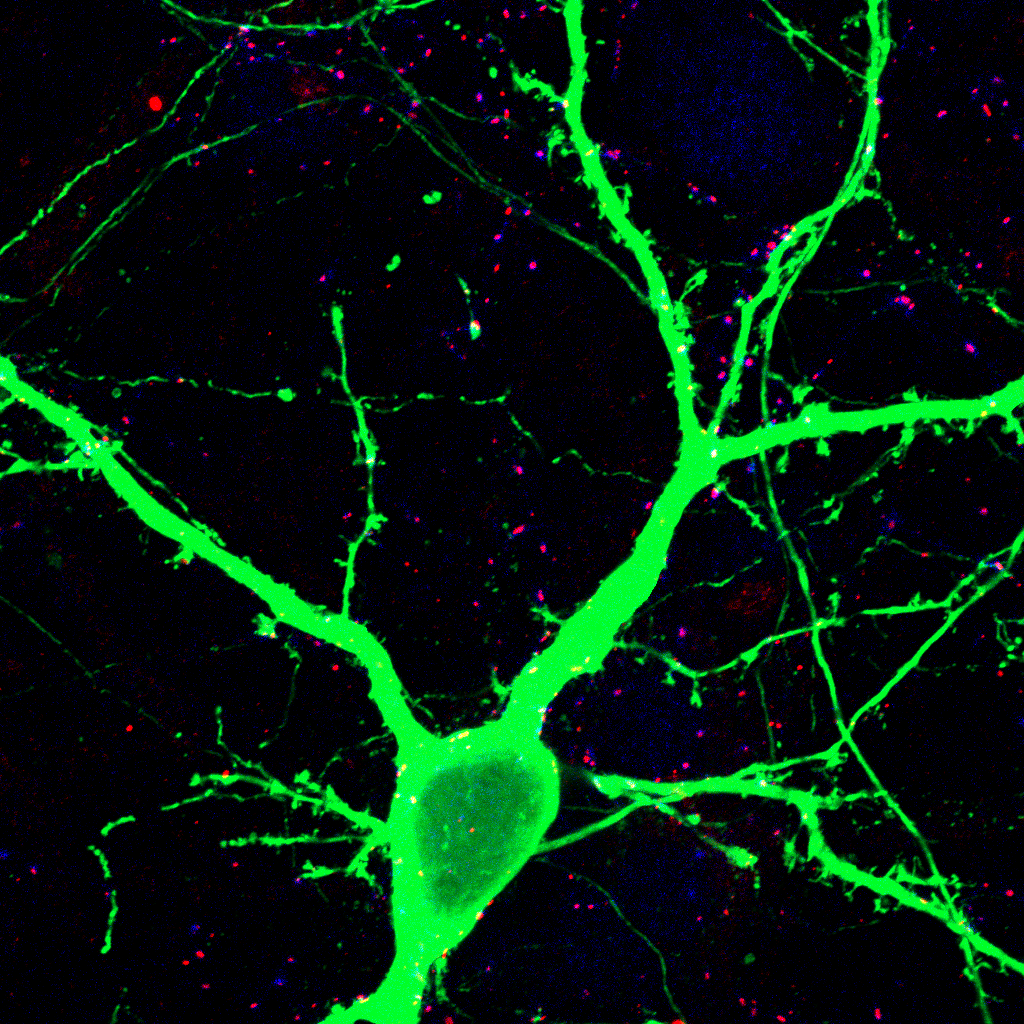

Supplement: Supplementary file 16 — Source data Fig. 2 [file 44321_2026_402_MOESM16_ESM.zip › Panel A and B/Control/Control (6).tif]

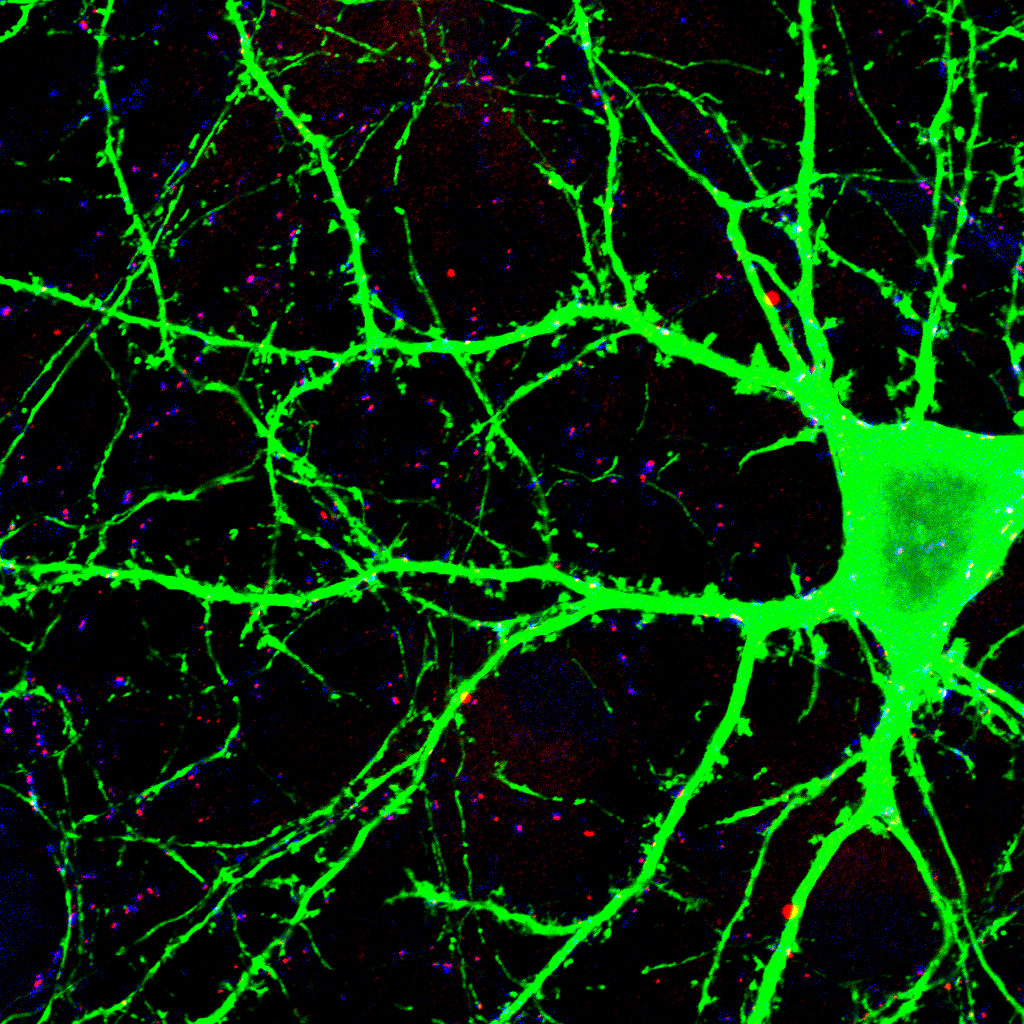

Supplement: Supplementary file 16 — Source data Fig. 2 [file 44321_2026_402_MOESM16_ESM.zip › Panel A and B/Control/Control (7).tif]

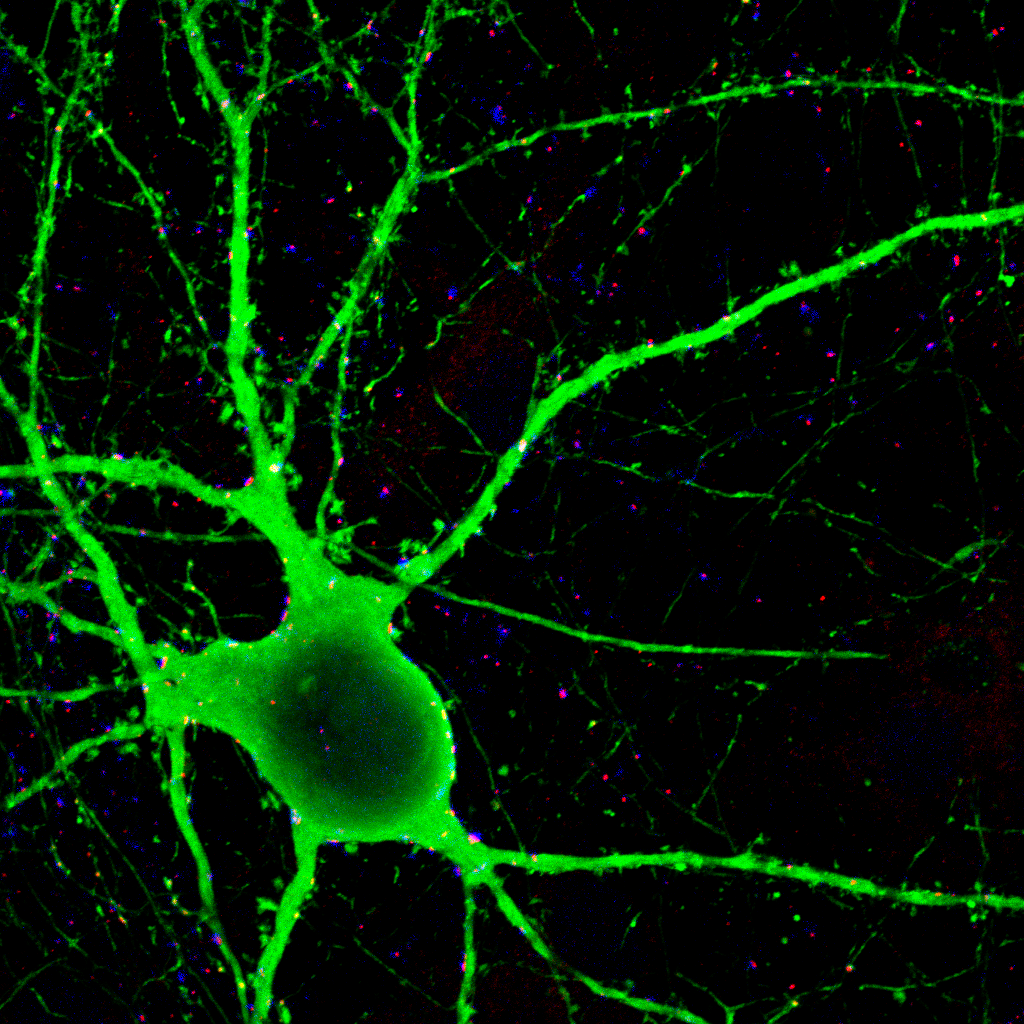

Supplement: Supplementary file 16 — Source data Fig. 2 [file 44321_2026_402_MOESM16_ESM.zip › Panel A and B/Control/Control (8).tif]

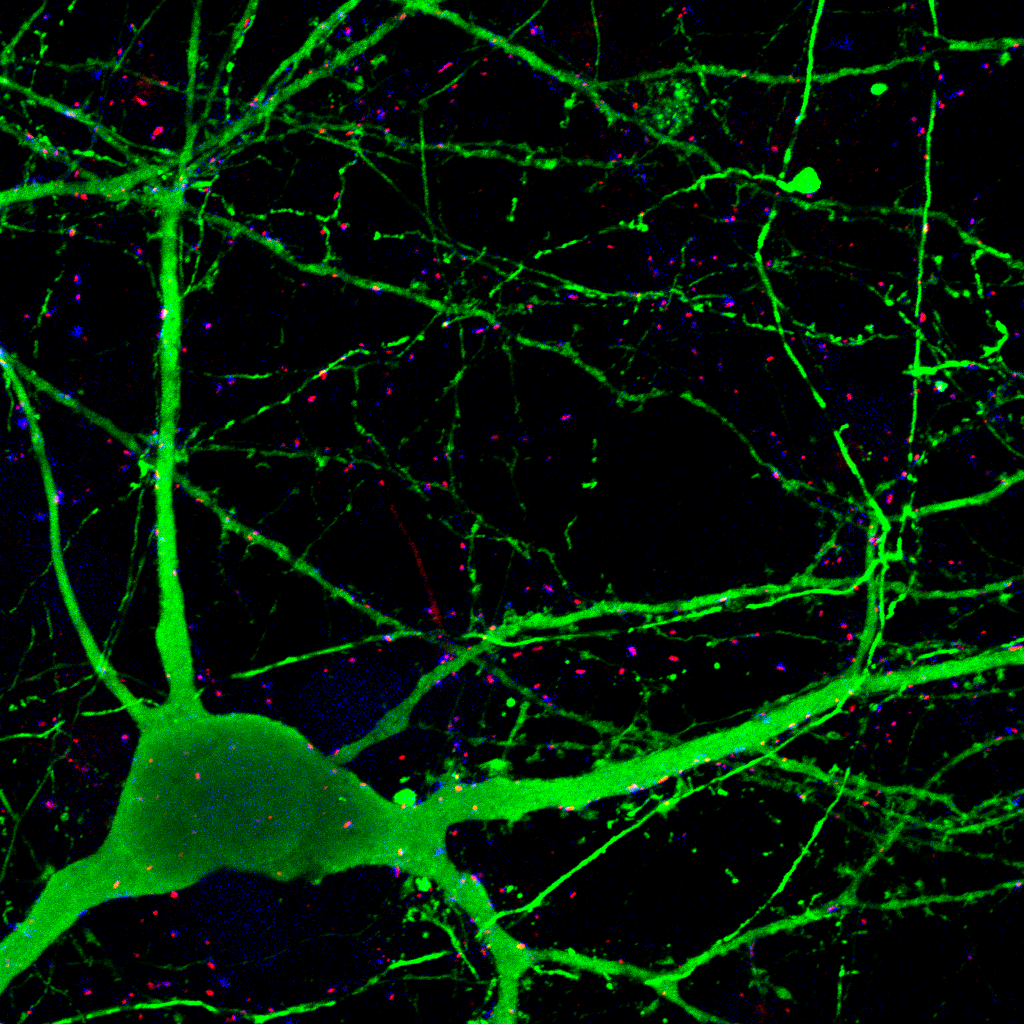

Supplement: Supplementary file 16 — Source data Fig. 2 [file 44321_2026_402_MOESM16_ESM.zip › Panel A and B/Control/Control (9).tif]

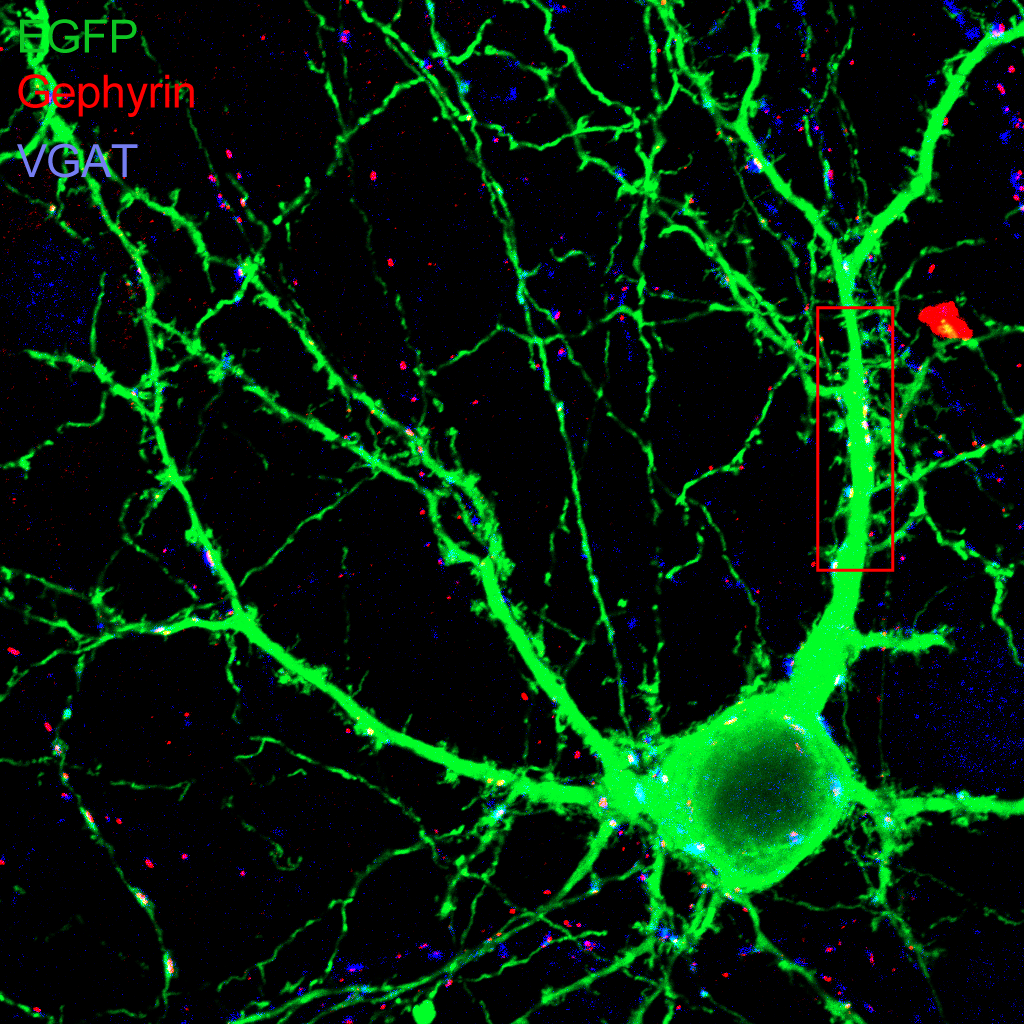

Supplement: Supplementary file 16 — Source data Fig. 2 [file 44321_2026_402_MOESM16_ESM.zip › Panel A and B/Control.tif]

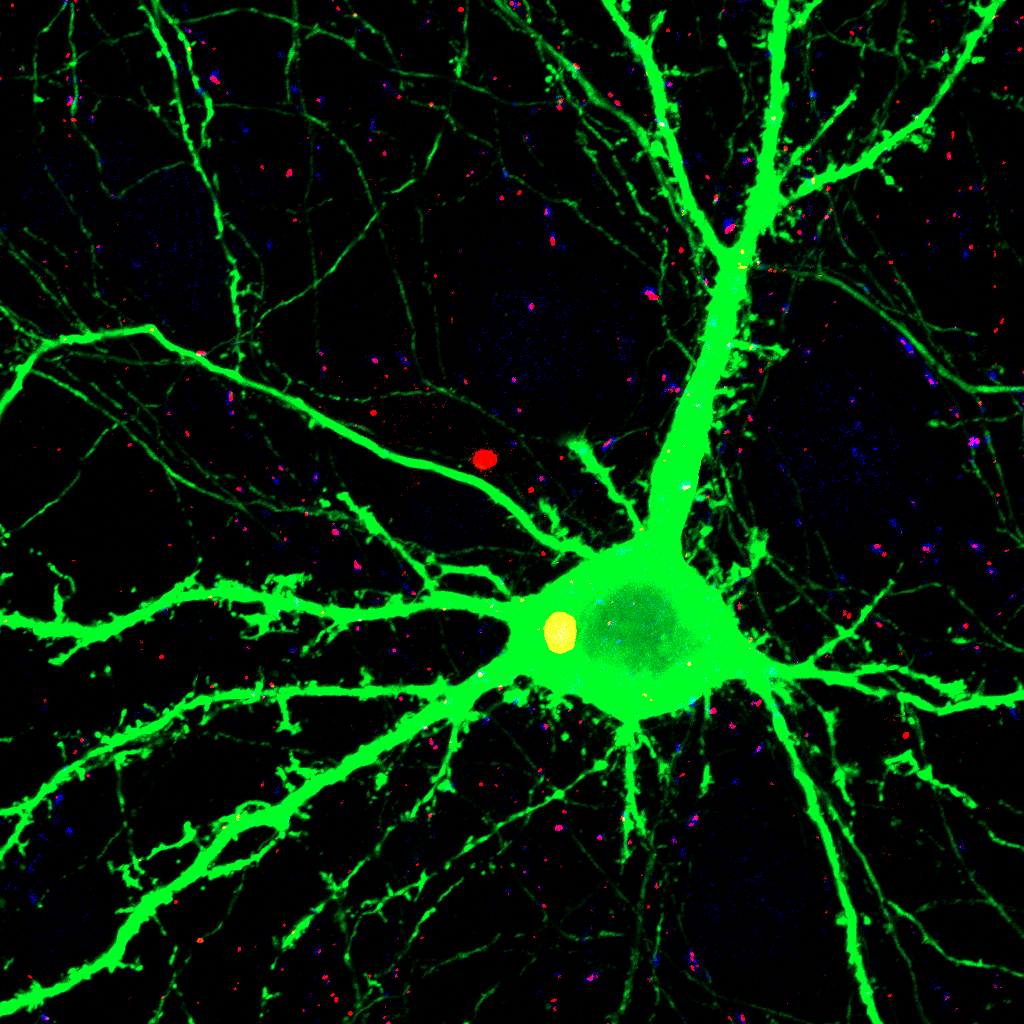

Supplement: Supplementary file 16 — Source data Fig. 2 [file 44321_2026_402_MOESM16_ESM.zip › Panel A and B/MDGA1 V116M, A688V/MDGA1 V116M, A688V (1).tif]

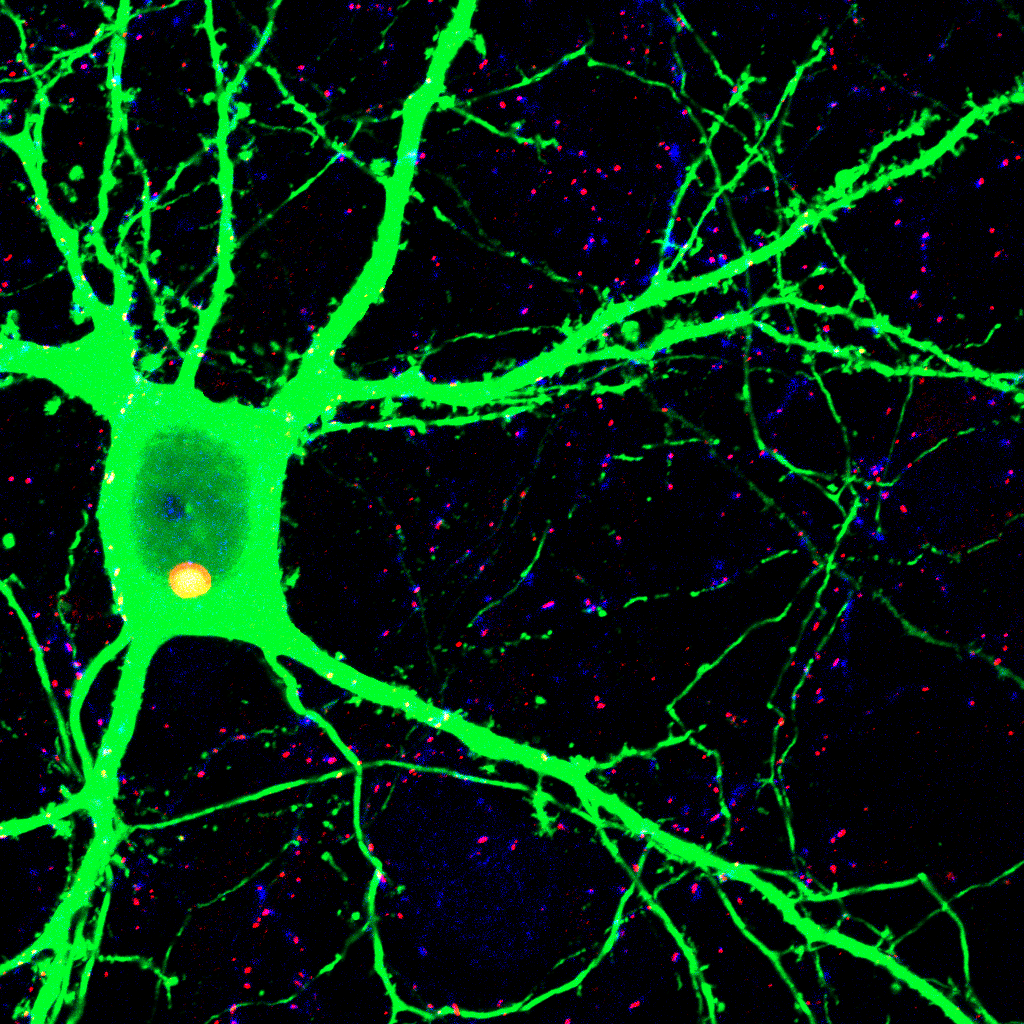

Supplement: Supplementary file 16 — Source data Fig. 2 [file 44321_2026_402_MOESM16_ESM.zip › Panel A and B/MDGA1 V116M, A688V/MDGA1 V116M, A688V (10).tif]

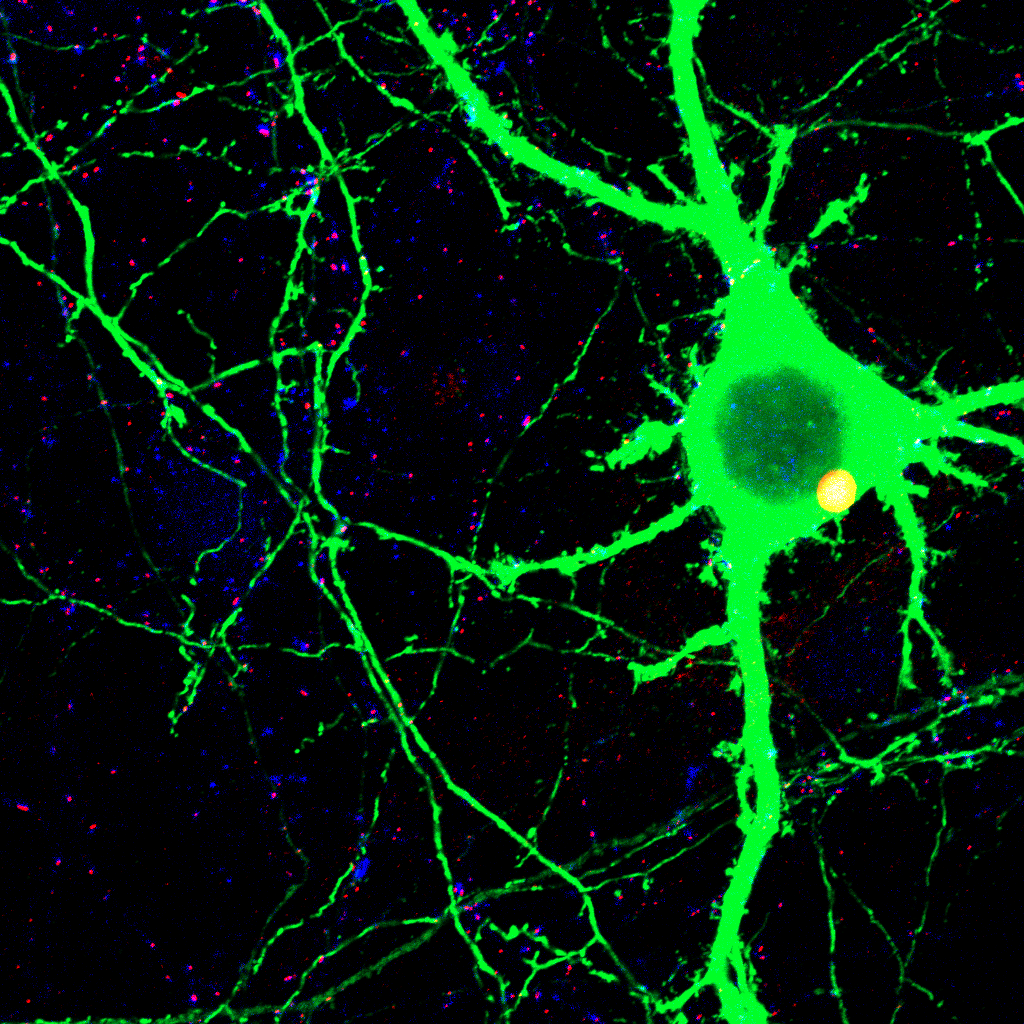

Supplement: Supplementary file 16 — Source data Fig. 2 [file 44321_2026_402_MOESM16_ESM.zip › Panel A and B/MDGA1 V116M, A688V/MDGA1 V116M, A688V (11).tif]

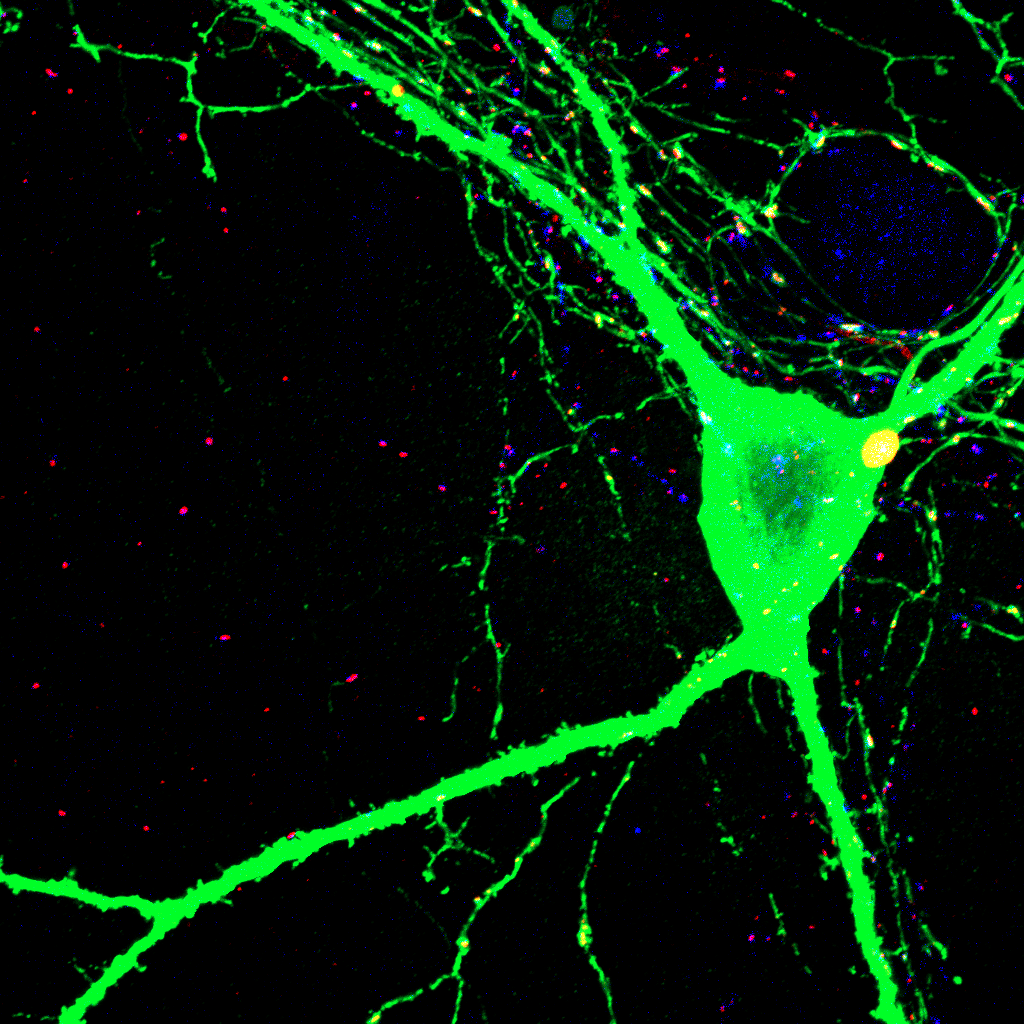

Supplement: Supplementary file 16 — Source data Fig. 2 [file 44321_2026_402_MOESM16_ESM.zip › Panel A and B/MDGA1 V116M, A688V/MDGA1 V116M, A688V (12).tif]

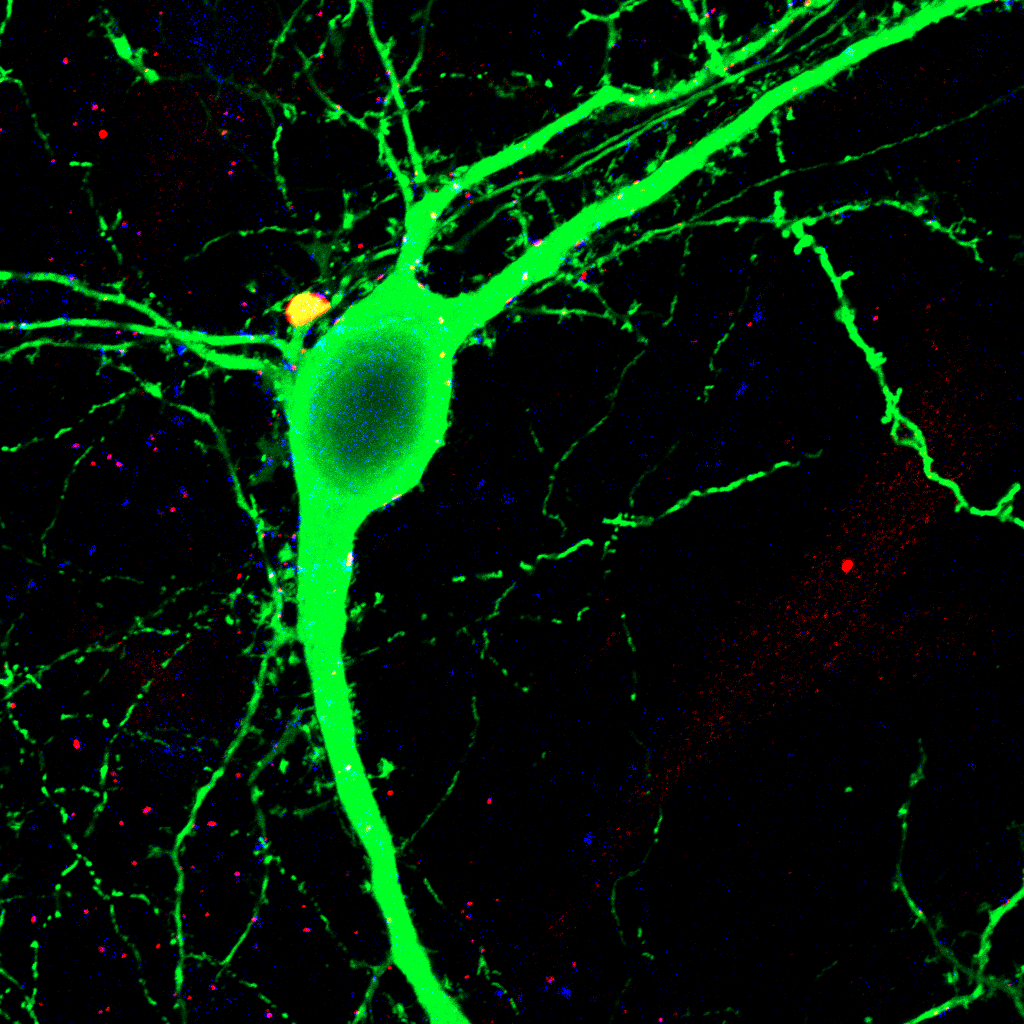

Supplement: Supplementary file 16 — Source data Fig. 2 [file 44321_2026_402_MOESM16_ESM.zip › Panel A and B/MDGA1 V116M, A688V/MDGA1 V116M, A688V (13).tif]

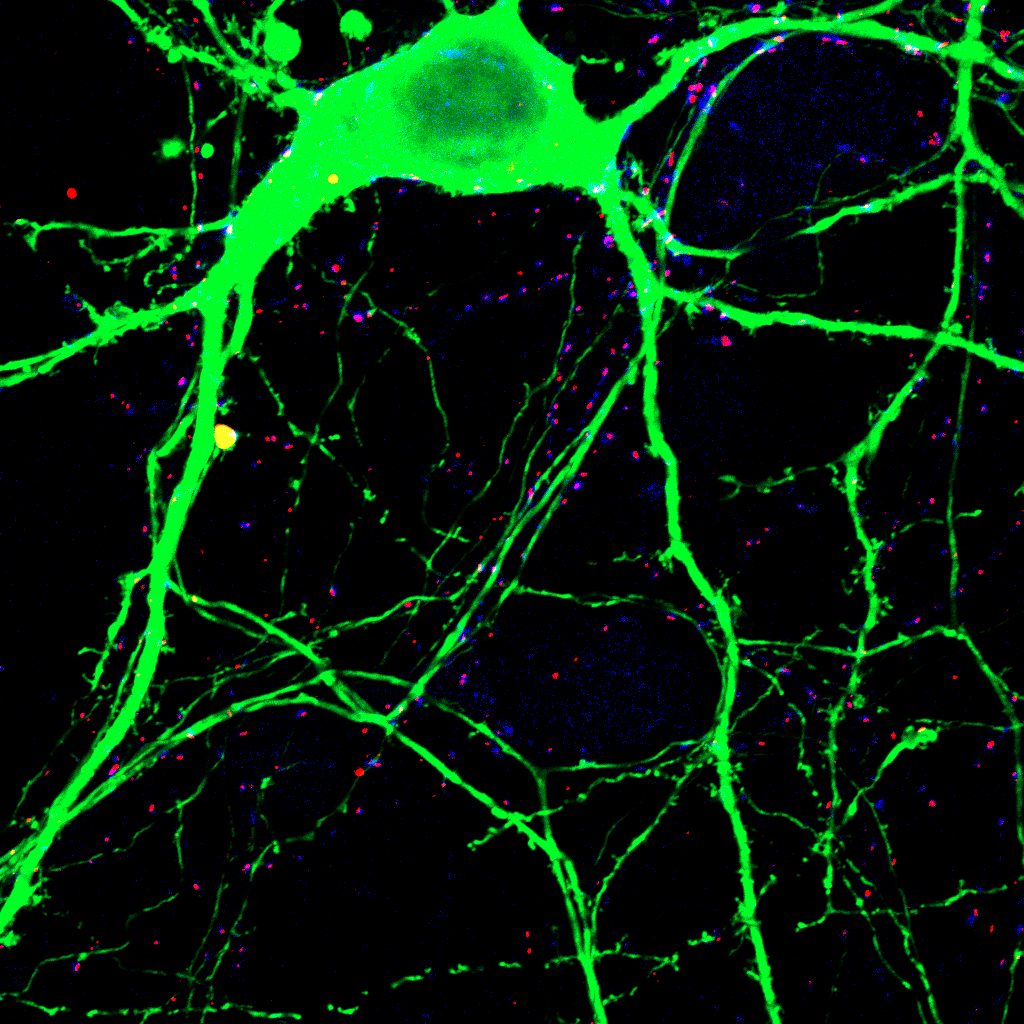

Supplement: Supplementary file 16 — Source data Fig. 2 [file 44321_2026_402_MOESM16_ESM.zip › Panel A and B/MDGA1 V116M, A688V/MDGA1 V116M, A688V (2).tif]

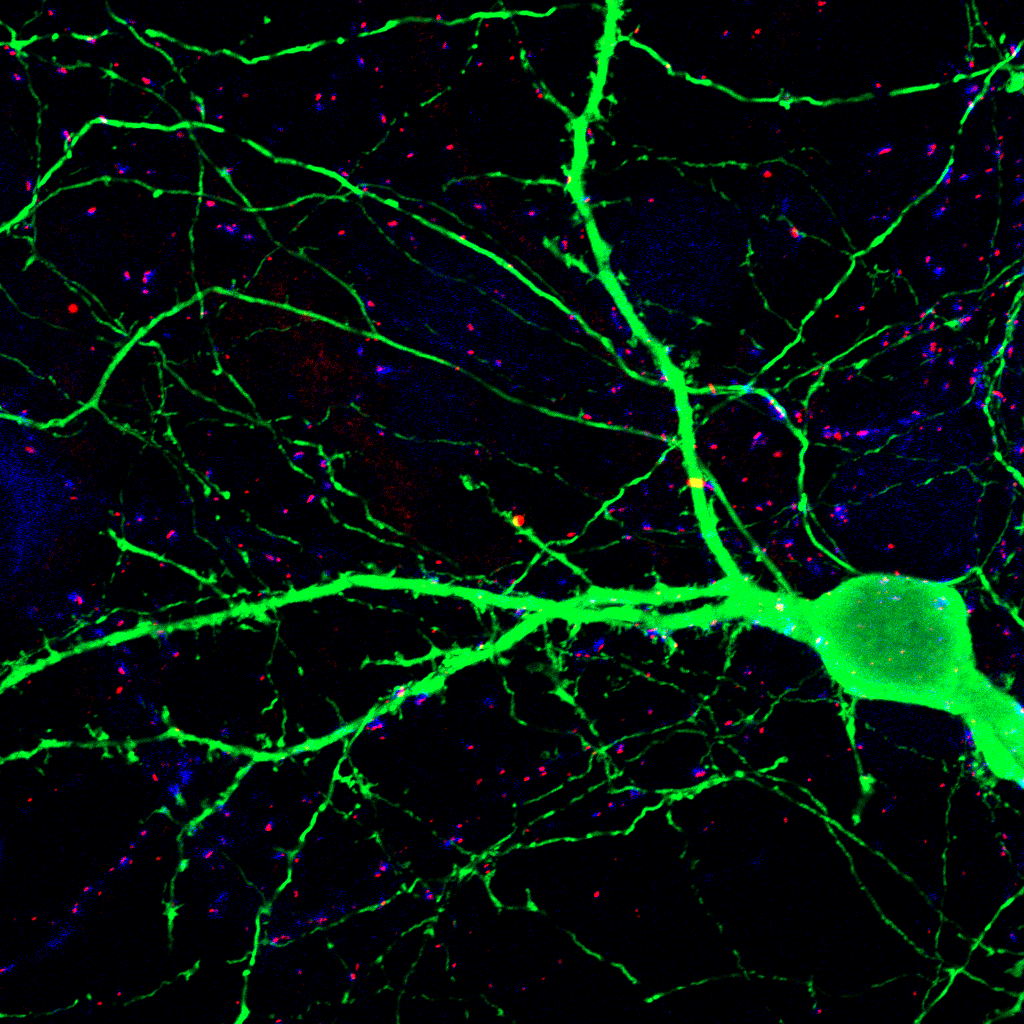

Supplement: Supplementary file 16 — Source data Fig. 2 [file 44321_2026_402_MOESM16_ESM.zip › Panel A and B/MDGA1 V116M, A688V/MDGA1 V116M, A688V (3).tif]

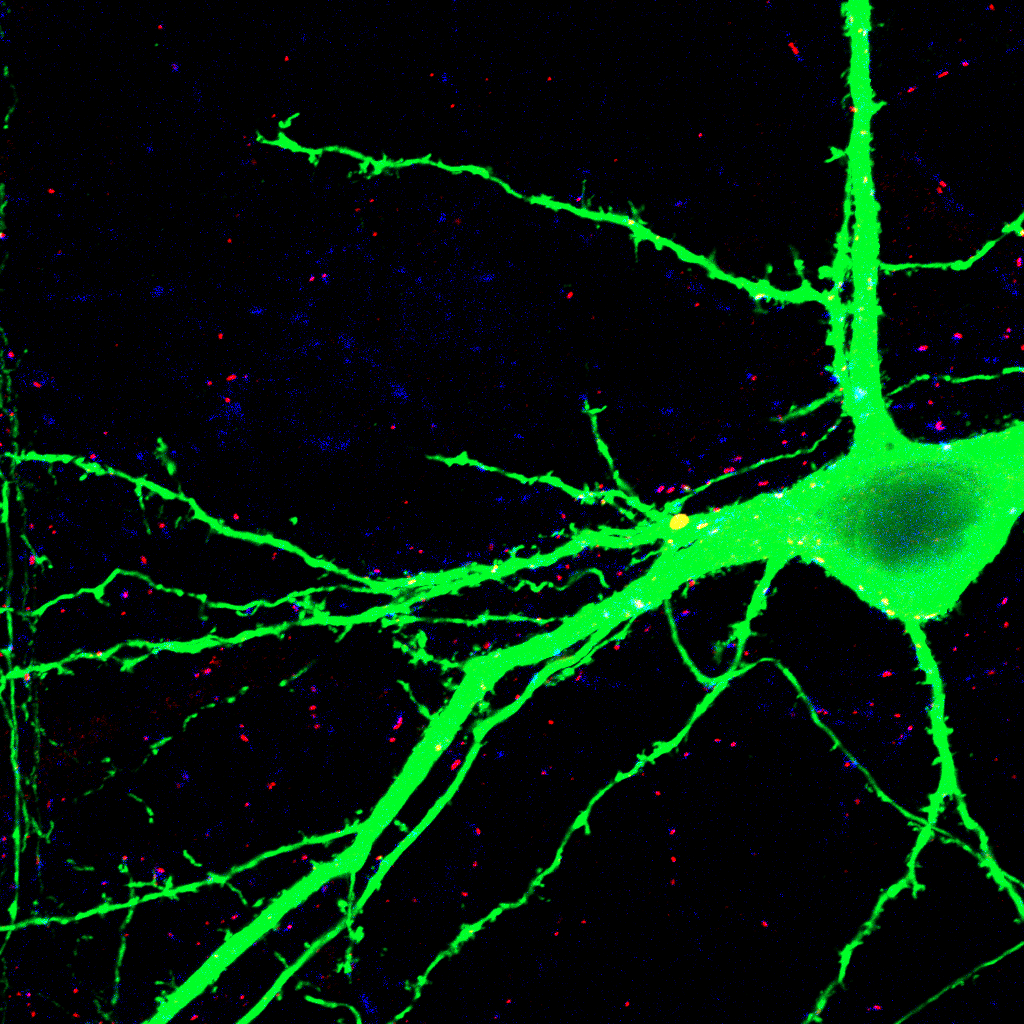

Supplement: Supplementary file 16 — Source data Fig. 2 [file 44321_2026_402_MOESM16_ESM.zip › Panel A and B/MDGA1 V116M, A688V/MDGA1 V116M, A688V (4).tif]

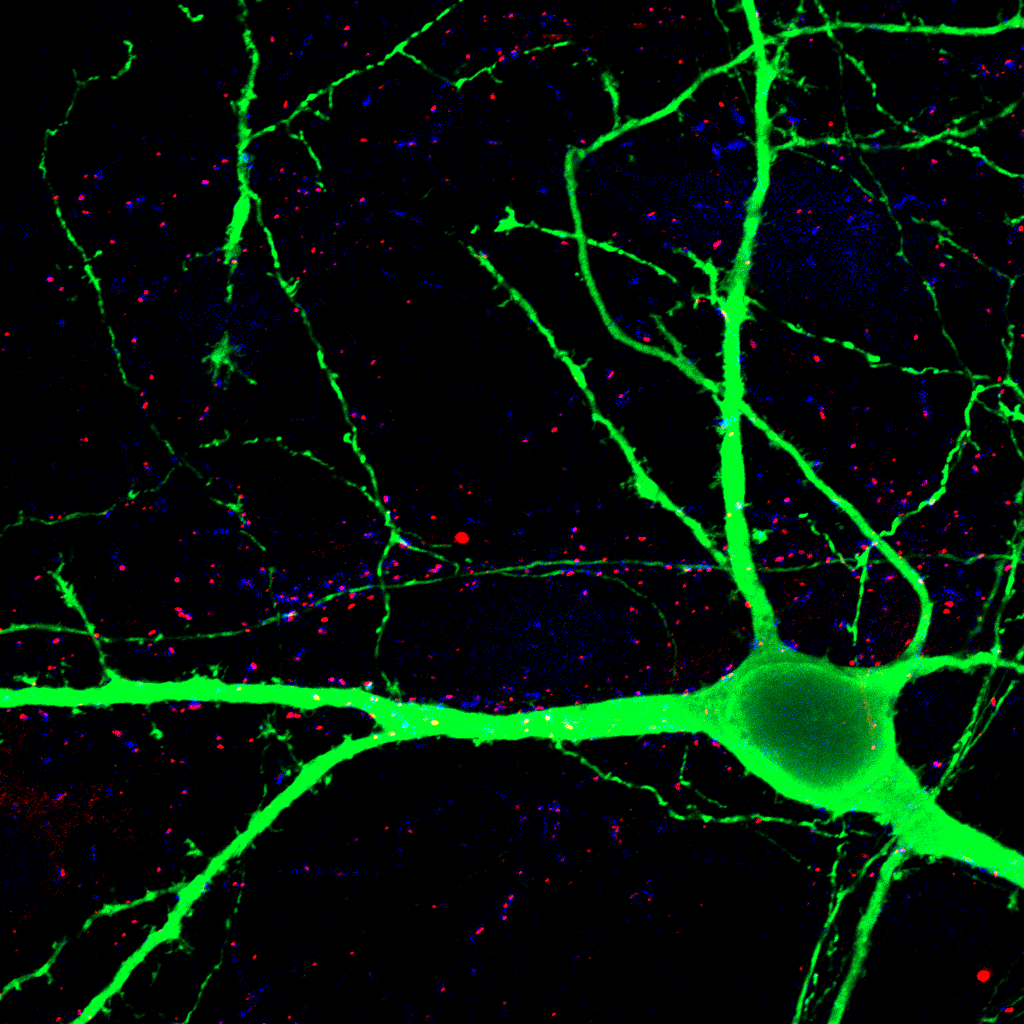

Supplement: Supplementary file 16 — Source data Fig. 2 [file 44321_2026_402_MOESM16_ESM.zip › Panel A and B/MDGA1 V116M, A688V/MDGA1 V116M, A688V (5).tif]

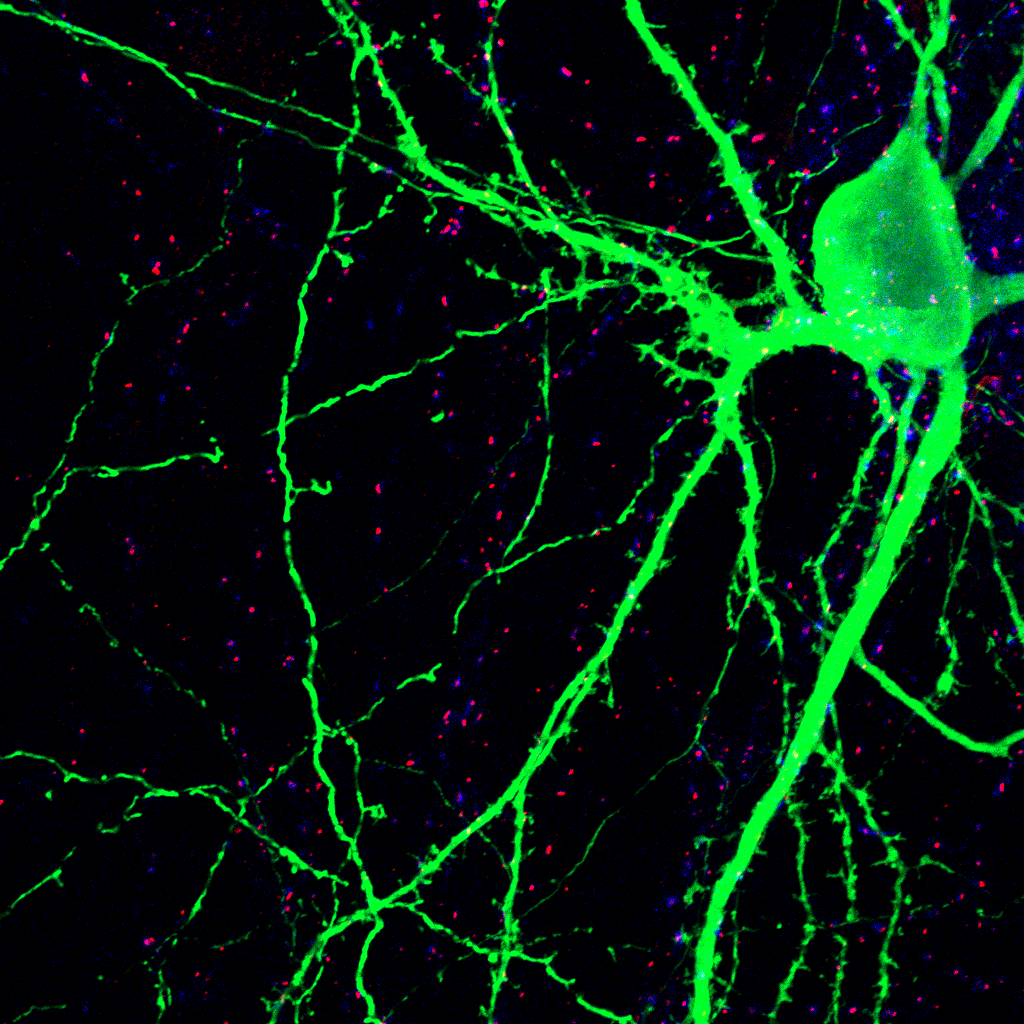

Supplement: Supplementary file 16 — Source data Fig. 2 [file 44321_2026_402_MOESM16_ESM.zip › Panel A and B/MDGA1 V116M, A688V/MDGA1 V116M, A688V (6).tif]

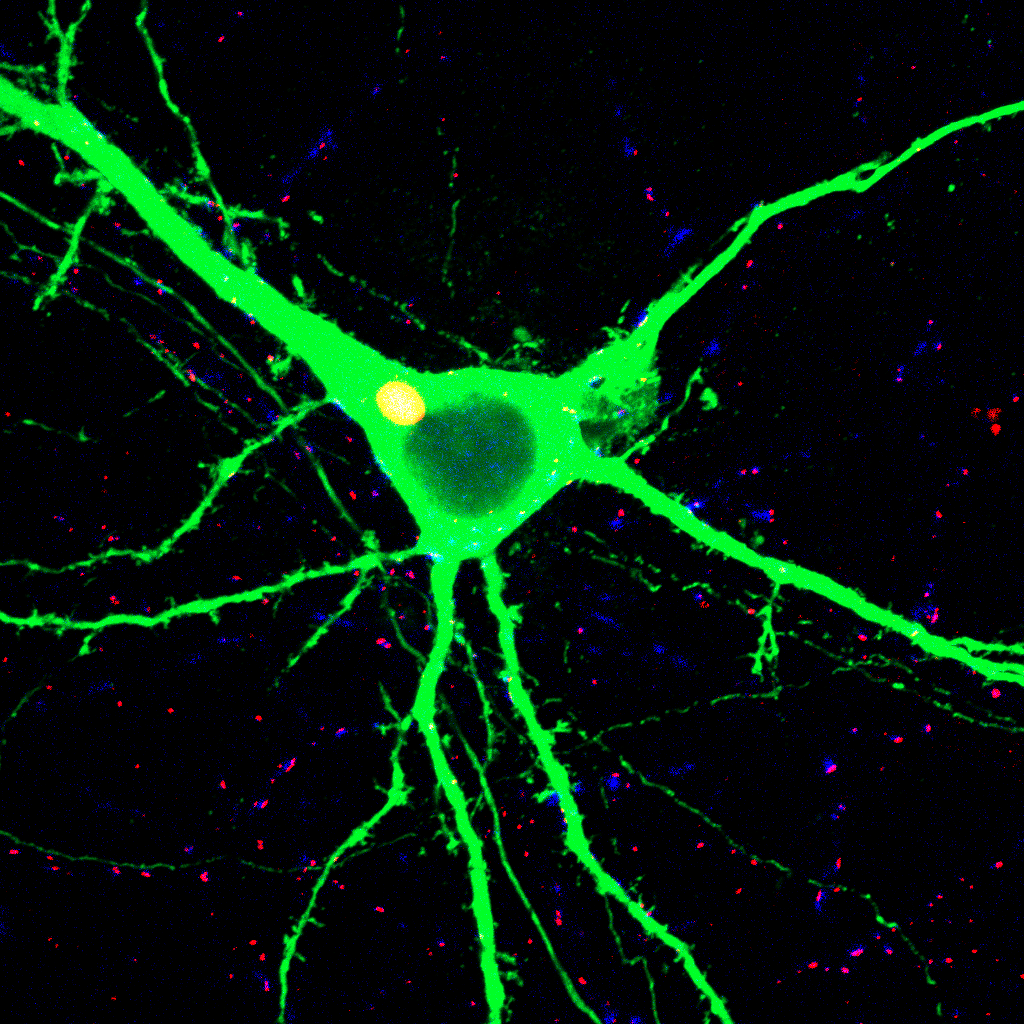

Supplement: Supplementary file 16 — Source data Fig. 2 [file 44321_2026_402_MOESM16_ESM.zip › Panel A and B/MDGA1 V116M, A688V/MDGA1 V116M, A688V (7).tif]

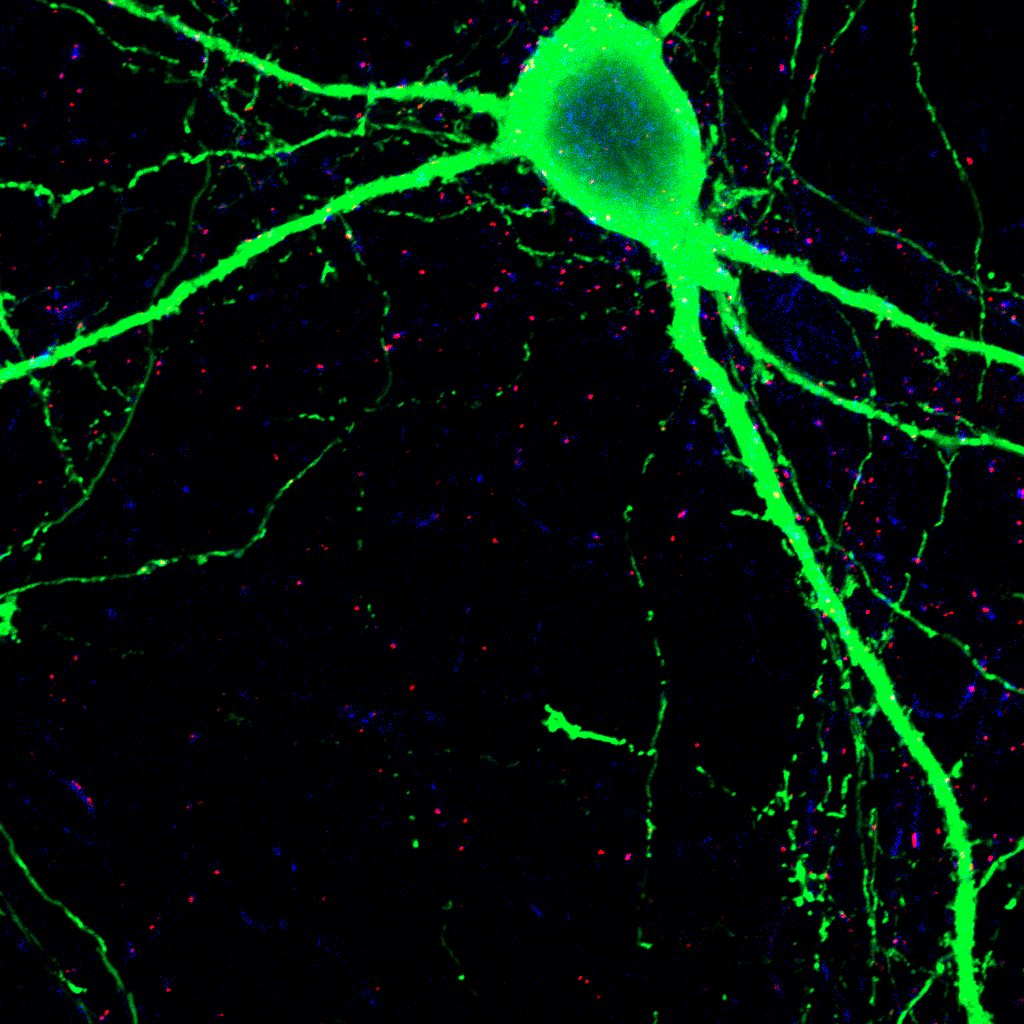

Supplement: Supplementary file 16 — Source data Fig. 2 [file 44321_2026_402_MOESM16_ESM.zip › Panel A and B/MDGA1 V116M, A688V/MDGA1 V116M, A688V (8).tif]

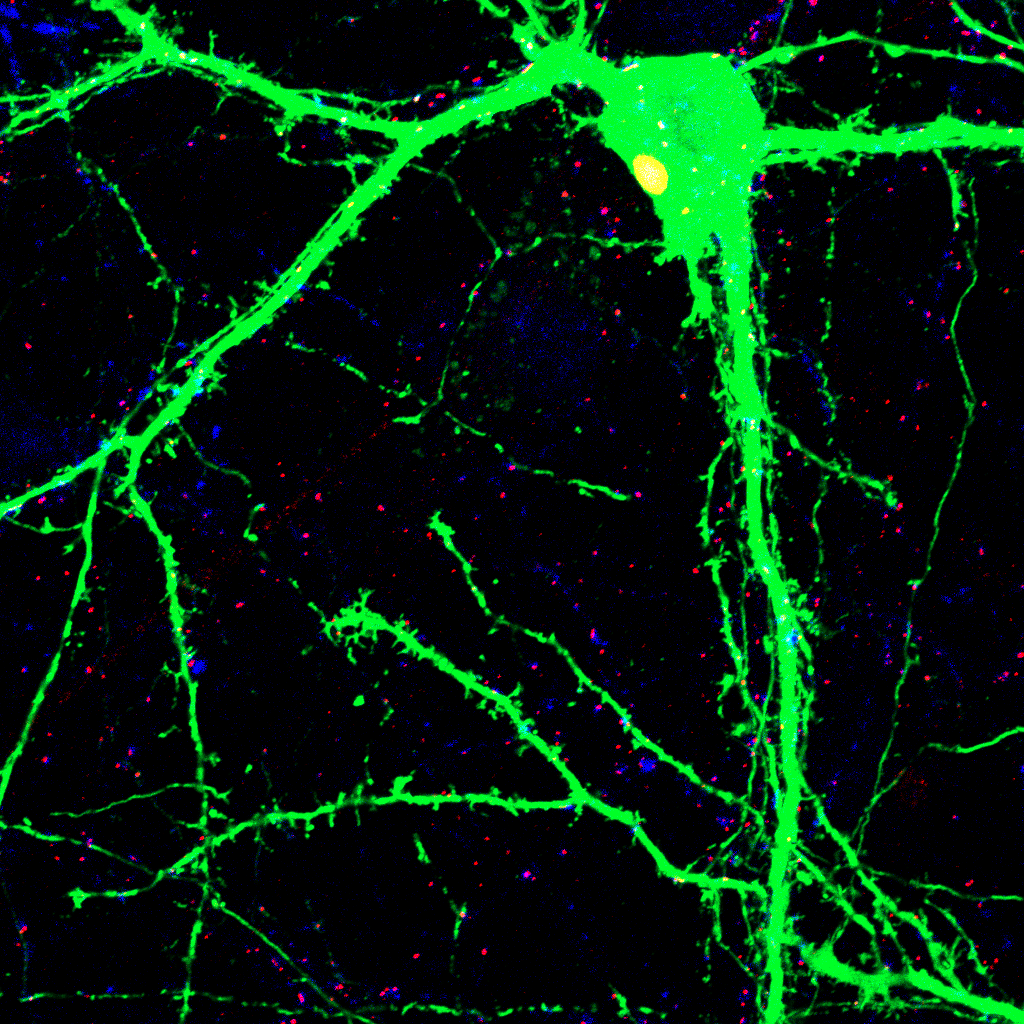

Supplement: Supplementary file 16 — Source data Fig. 2 [file 44321_2026_402_MOESM16_ESM.zip › Panel A and B/MDGA1 V116M, A688V/MDGA1 V116M, A688V (9).tif]

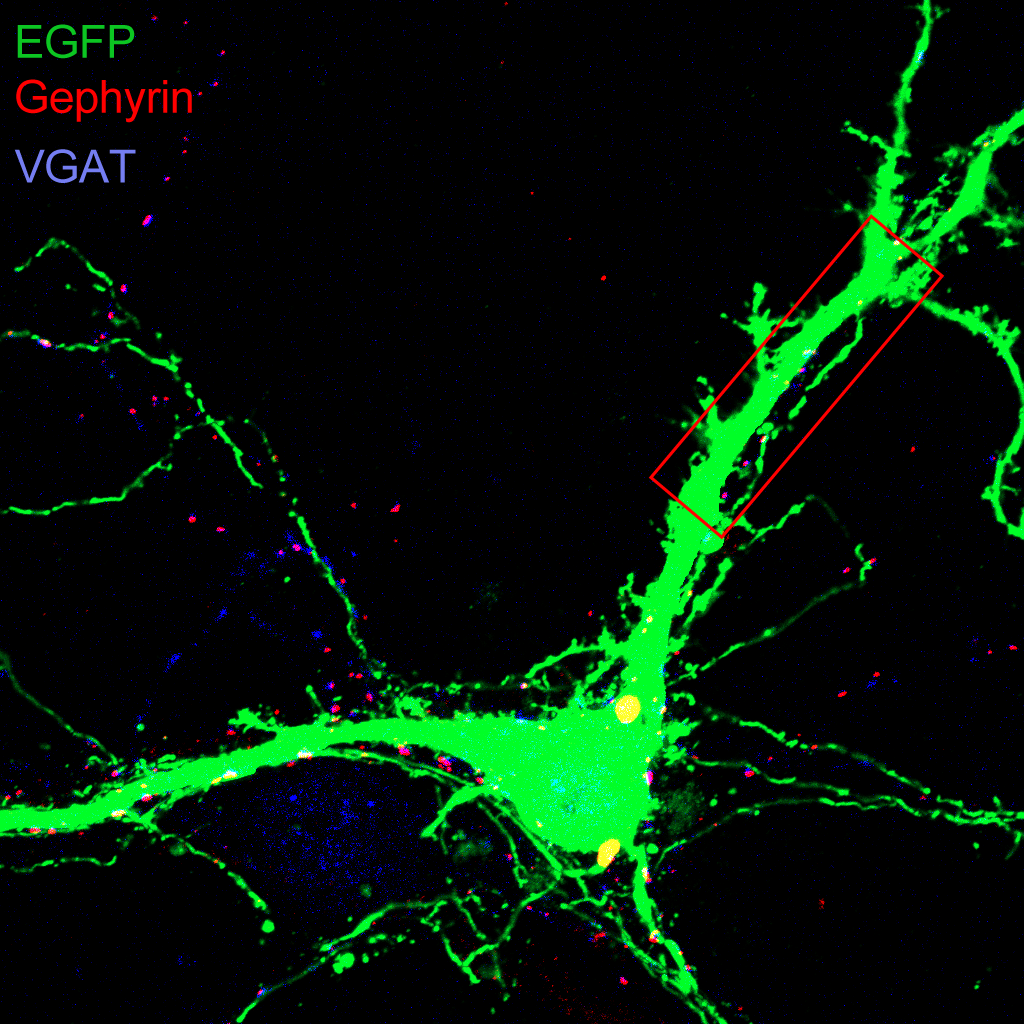

Supplement: Supplementary file 16 — Source data Fig. 2 [file 44321_2026_402_MOESM16_ESM.zip › Panel A and B/MDGA1 V116M, A688V.tif]

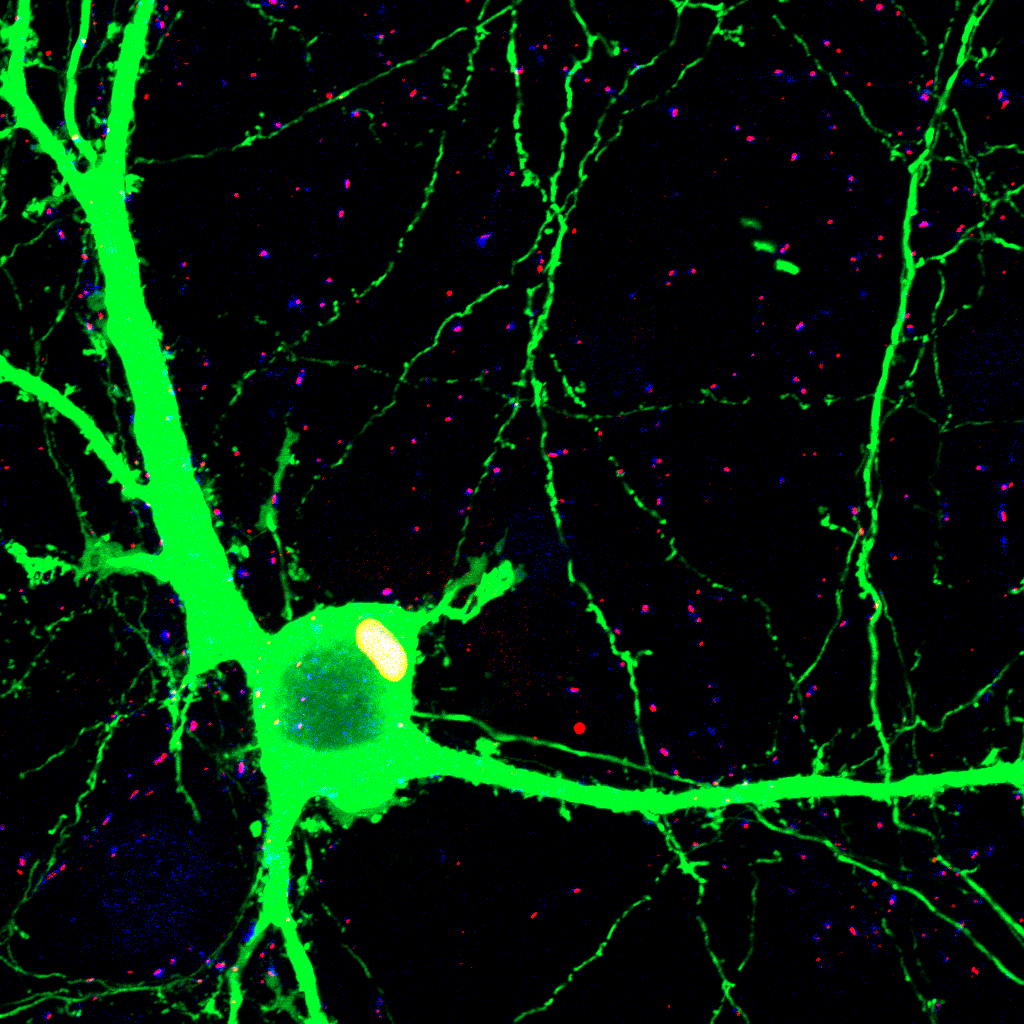

Supplement: Supplementary file 16 — Source data Fig. 2 [file 44321_2026_402_MOESM16_ESM.zip › Panel A and B/MDGA1 WT/MDGA1 WT (1).tif]

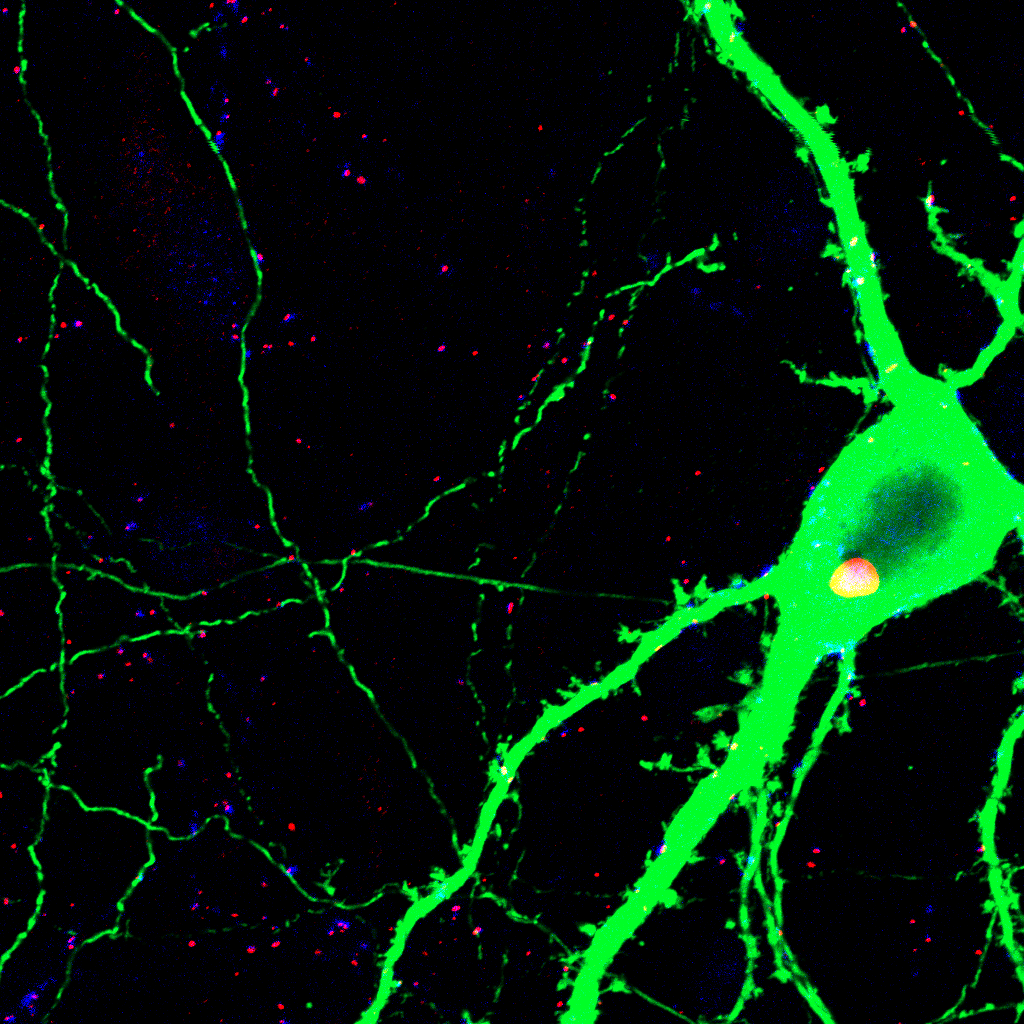

Supplement: Supplementary file 16 — Source data Fig. 2 [file 44321_2026_402_MOESM16_ESM.zip › Panel A and B/MDGA1 WT/MDGA1 WT (10).tif]

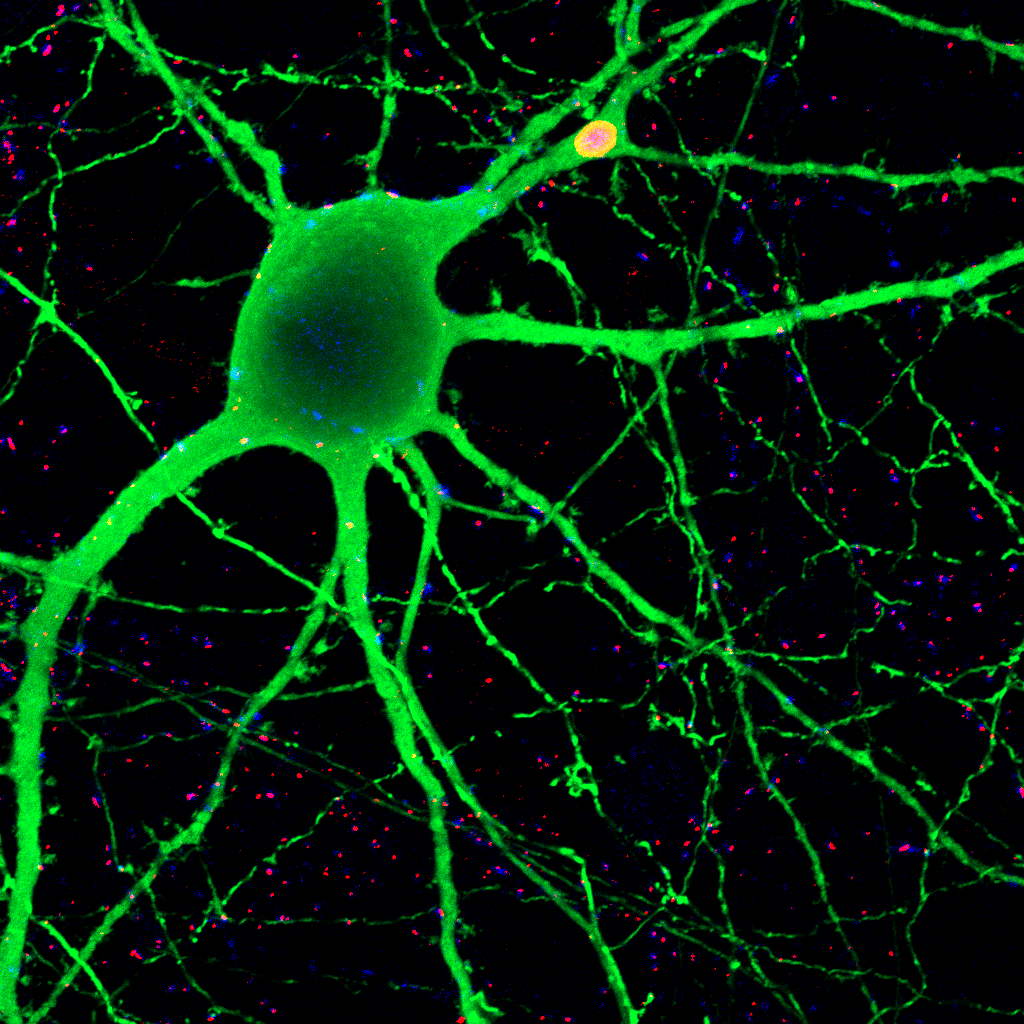

Supplement: Supplementary file 16 — Source data Fig. 2 [file 44321_2026_402_MOESM16_ESM.zip › Panel A and B/MDGA1 WT/MDGA1 WT (11).tif]

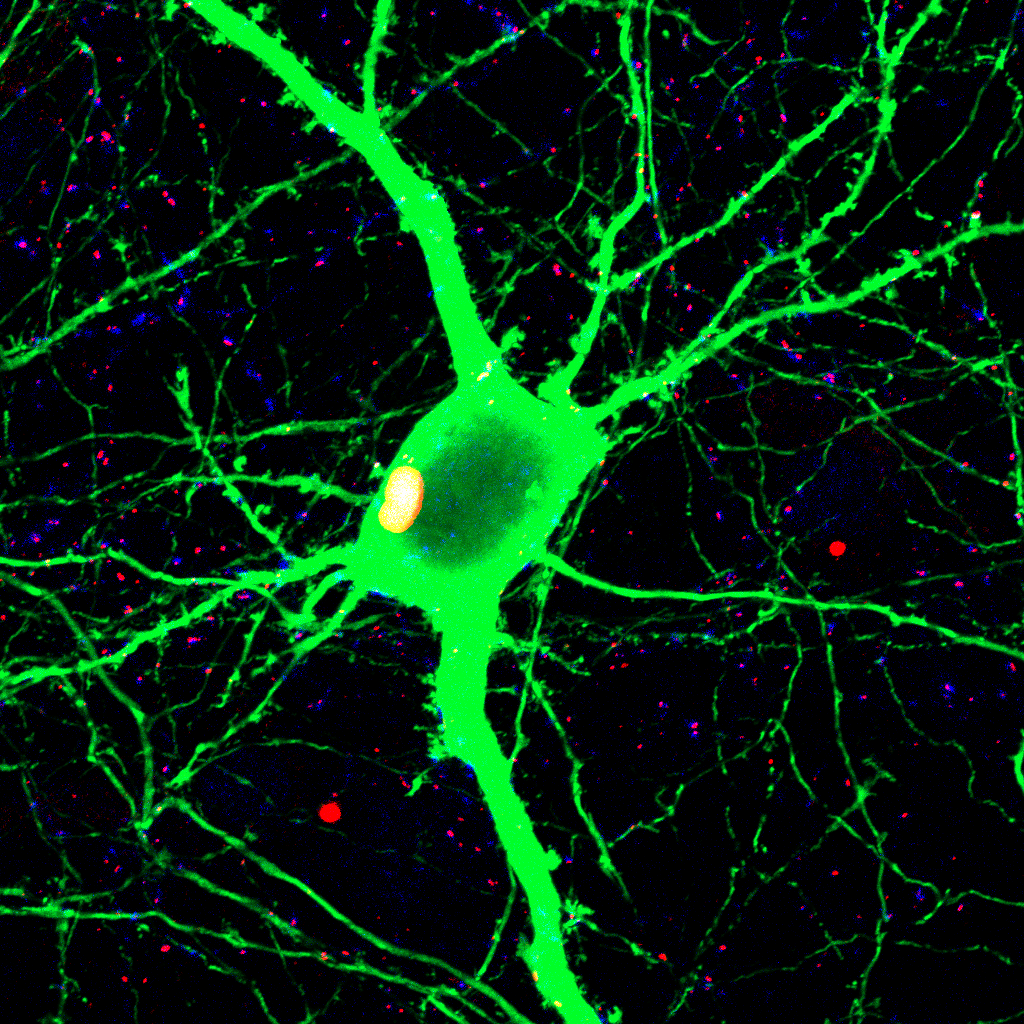

Supplement: Supplementary file 16 — Source data Fig. 2 [file 44321_2026_402_MOESM16_ESM.zip › Panel A and B/MDGA1 WT/MDGA1 WT (12).tif]

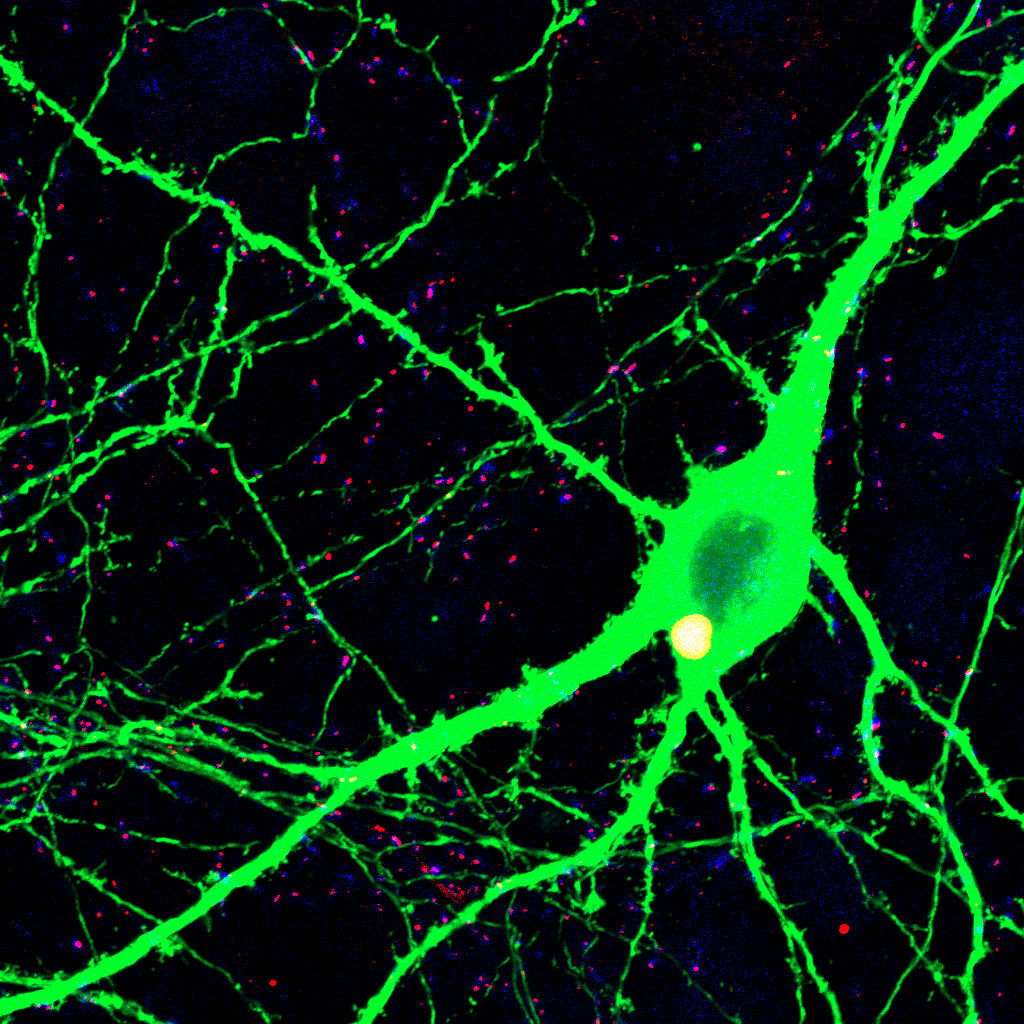

Supplement: Supplementary file 16 — Source data Fig. 2 [file 44321_2026_402_MOESM16_ESM.zip › Panel A and B/MDGA1 WT/MDGA1 WT (13).tif]

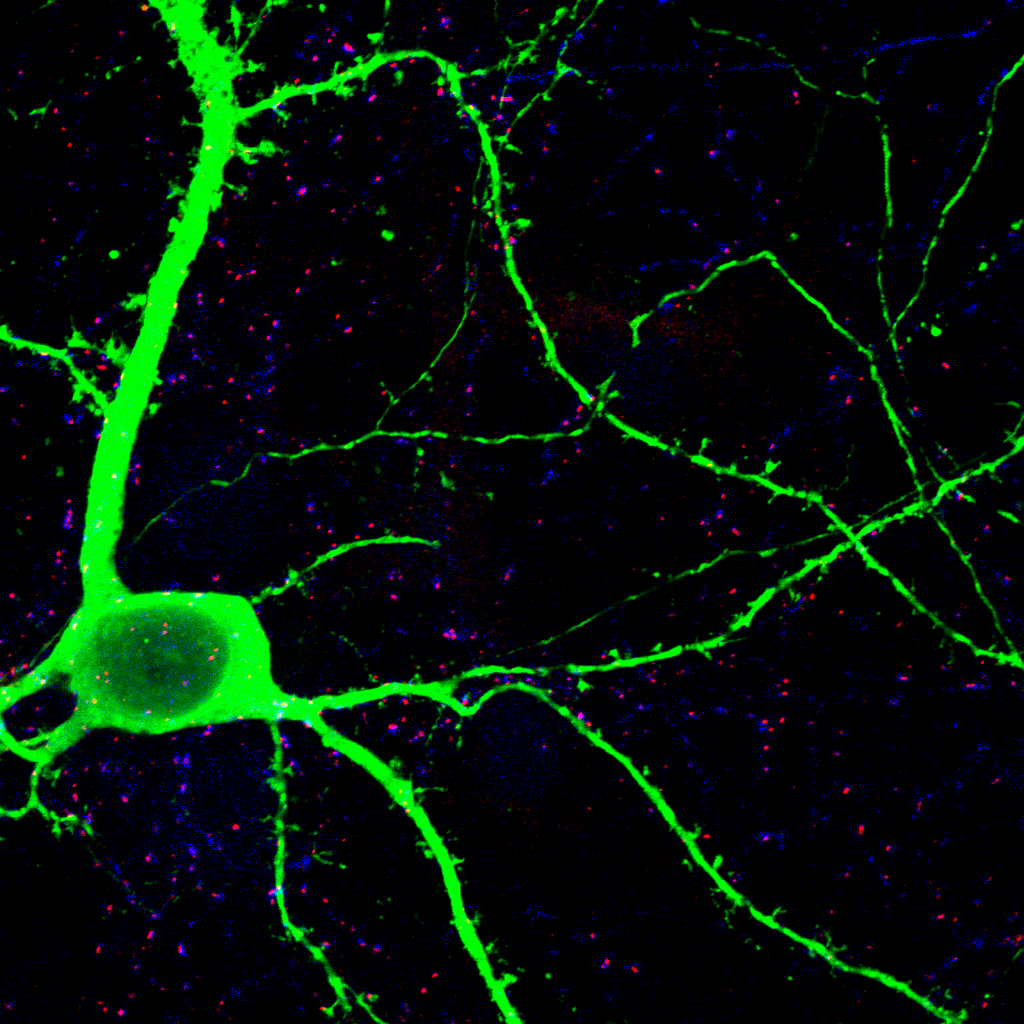

Supplement: Supplementary file 16 — Source data Fig. 2 [file 44321_2026_402_MOESM16_ESM.zip › Panel A and B/MDGA1 WT/MDGA1 WT (2).tif]

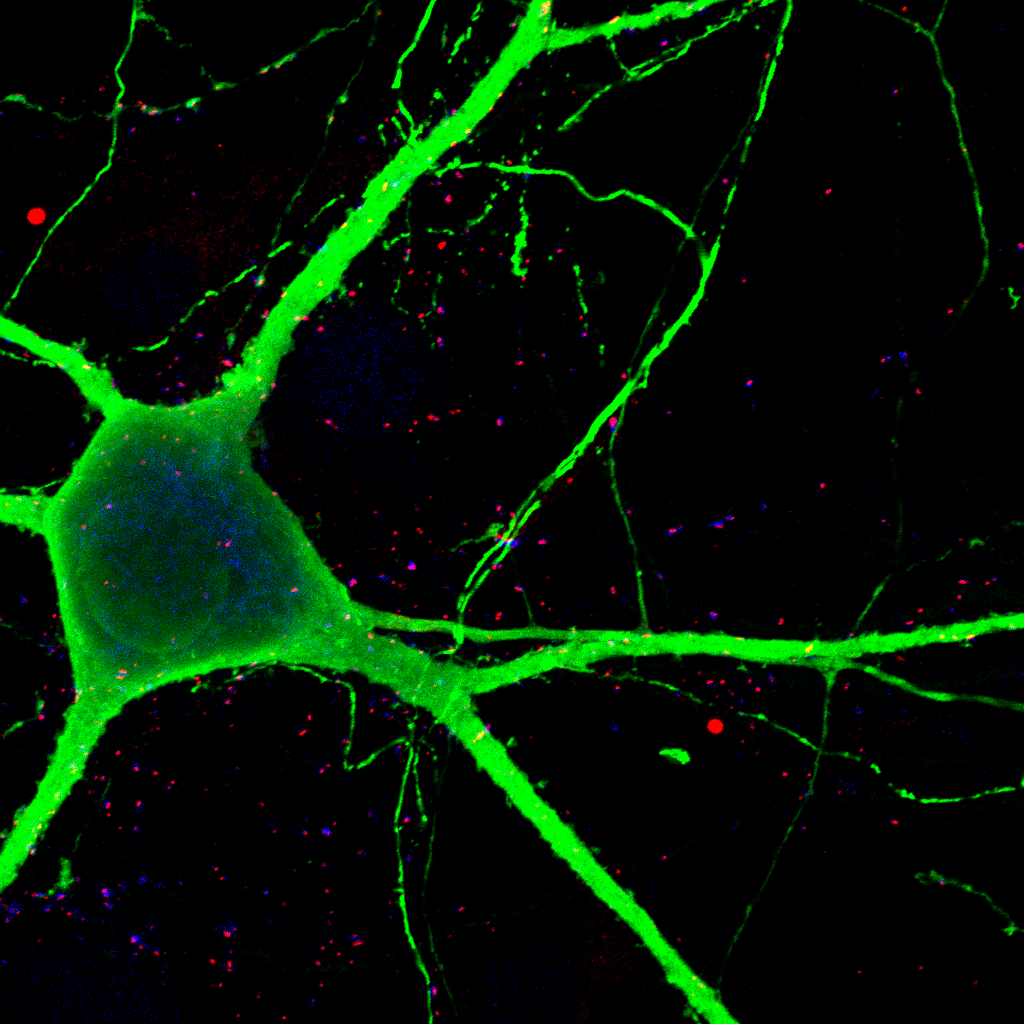

Supplement: Supplementary file 16 — Source data Fig. 2 [file 44321_2026_402_MOESM16_ESM.zip › Panel A and B/MDGA1 WT/MDGA1 WT (3).tif]
