# Supplementary material for: Bazedoxifene reverses sexually dimorphic autistic-like abnormalities in biallelic MDGA1-mutant mice
Source: EMBO Mol Med. 2026 Mar 20;18(4):1358–98. doi: 10.1038/s44321-026-00402-y (PMC13084050; doi:10.1038/s44321-026-00402-y)
Supplement: Supplementary file 16 — Source data Fig. 2 [file 44321_2026_402_MOESM16_ESM.zip › Panel C-G/Figure2C MDGA1 patient mutant overexpression mIPSC .docx]

# Figure2C MDGA1 patient mutant overexpression mIPSC

# Control – Representative mIPSC trace

• File: 2024_01_31_0028.abf

• Condition: Control

• Cell Type: Rat hippocampal cultured neuron

• Recording: mIPSC at −70 mV holding potential

• Start Point: 58.93419 s (Duration: 5 seconds)

• Analysis Software: Clampfit 11

## WT – Representative mIPSC trace

• File: 2024_01_30_0025.abf

• Condition: WT

• Cell Type: Rat hippocampal cultured neuron

• Recording: mIPSC at −70 mV holding potential

• Start Point: 243.88547 s (Duration: 5 seconds)

• Analysis Software: Clampfit 11

## VM (V116M, A688V) – Representative mIPSC trace

• File: 2024_01_31_0023.abf

• Condition: VM (V116M, A688V)

• Cell Type: Rat hippocampal cultured neuron

• Recording: mIPSC at −70 mV holding potential

• Start Point: 118.71257 s (Duration: 5 seconds)

• Analysis Software: Clampfit 11

## YC (Y635C, E756Q) – Representative mIPSC trace

• File: 2024_01_30_0026.abf

• Condition: YC (Y635C, E756Q)

• Cell Type: Rat hippocampal cultured neuron

• Recording: mIPSC at −70 mV holding potential

• Start Point: 244.68031 s (Duration: 5 seconds)

• Analysis Software: Clampfit 11
