# Supplementary material for: Bazedoxifene reverses sexually dimorphic autistic-like abnormalities in biallelic MDGA1-mutant mice
Source: EMBO Mol Med. 2026 Mar 20;18(4):1358–98. doi: 10.1038/s44321-026-00402-y (PMC13084050; doi:10.1038/s44321-026-00402-y)
Supplement: Supplementary file 16 — Source data Fig. 2 [file 44321_2026_402_MOESM16_ESM.zip › Panel K-N/Figure 2.docx]

**Figure 2 K – N**

**Control**

File name: 2021_11_16_0046.abf

Cropped trace timepoint (ms): 414.5 – 514.5

**WT**

File name: 2021_11_15_0037.abf

Cropped trace timepoint (ms): 414.5 – 514.5

**VM/AV**

File name: 2021_11_15_0043.abf

Cropped trace timepoint (ms): 414.5 – 514.5

**VM/AV**

File name: 2021_06_19_0018.abf

Cropped trace timepoint (ms): 414.5 – 514.5
