# Supplementary material for: Bazedoxifene reverses sexually dimorphic autistic-like abnormalities in biallelic MDGA1-mutant mice
Source: EMBO Mol Med. 2026 Mar 20;18(4):1358–98. doi: 10.1038/s44321-026-00402-y (PMC13084050; doi:10.1038/s44321-026-00402-y)
Supplement: Supplementary file 16 — Source data Fig. 2 [file 44321_2026_402_MOESM16_ESM.zip › Panel R-T/WT/Fig 1R WT.pptx]

## Slide 1
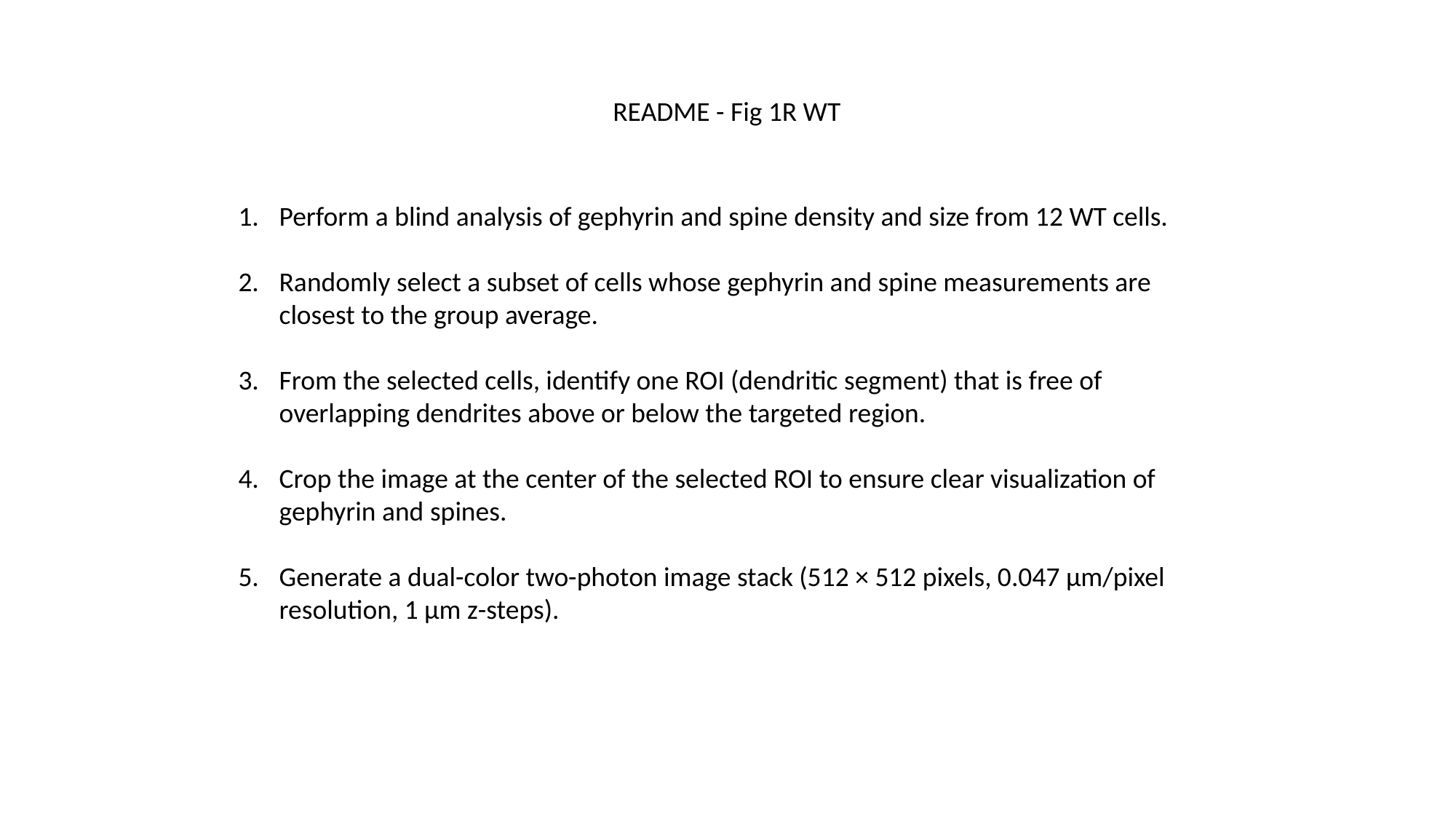

README - Fig 1R WT
Perform a blind analysis of gephyrin and spine density and size from 12 WT cells.
Randomly select a subset of cells whose gephyrin and spine measurements are closest to the group average.
From the selected cells, identify one ROI (dendritic segment) that is free of overlapping dendrites above or below the targeted region.
Crop the image at the center of the selected ROI to ensure clear visualization of gephyrin and spines.
Generate a dual-color two-photon image stack (512 × 512 pixels, 0.047 µm/pixel resolution, 1 µm z-steps).
