# Supplementary figures and images for: Bazedoxifene reverses sexually dimorphic autistic-like abnormalities in biallelic MDGA1-mutant mice (part 2 of 2)
Source: EMBO Mol Med. 2026 Mar 20;18(4):1358–98. doi: 10.1038/s44321-026-00402-y (PMC13084050; doi:10.1038/s44321-026-00402-y)

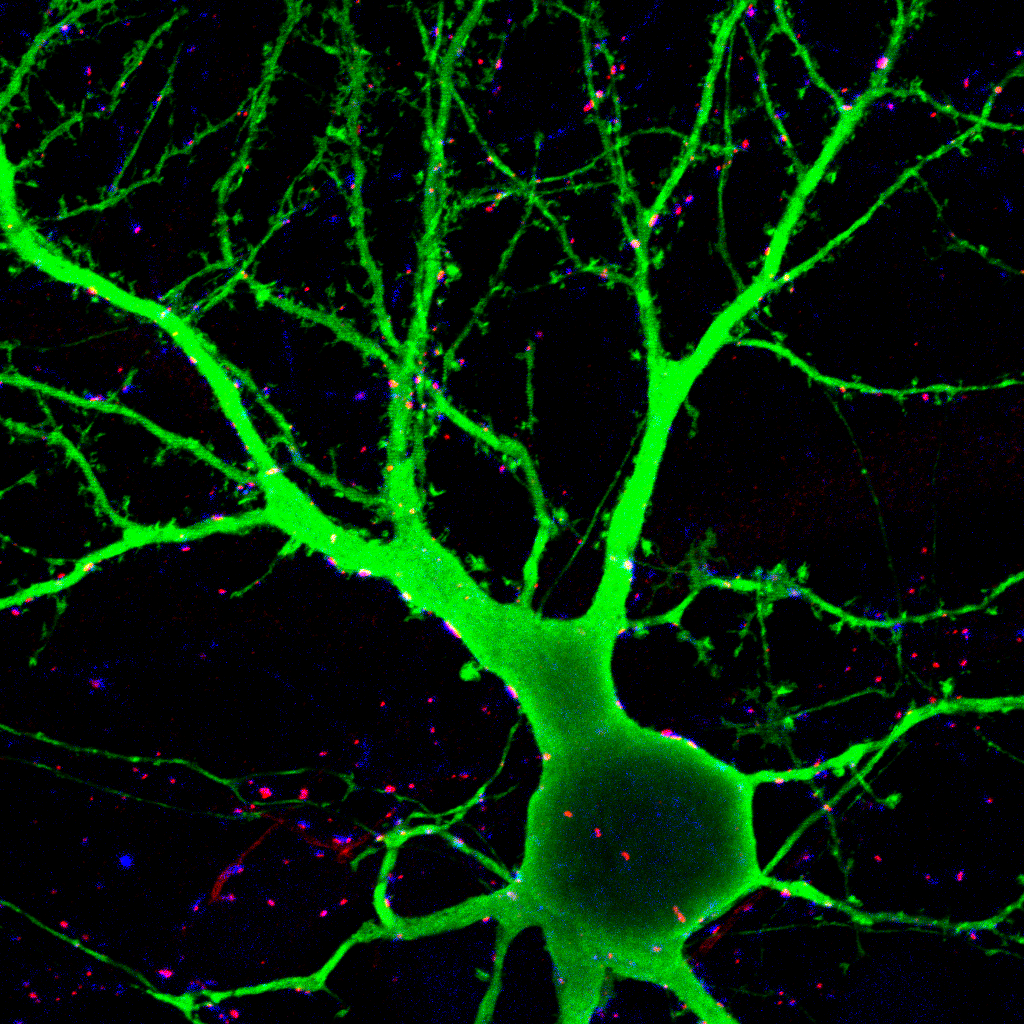

Supplement: Supplementary file 16 — Source data Fig. 2 [file 44321_2026_402_MOESM16_ESM.zip › Panel A and B/MDGA1 WT/MDGA1 WT (4).tif]

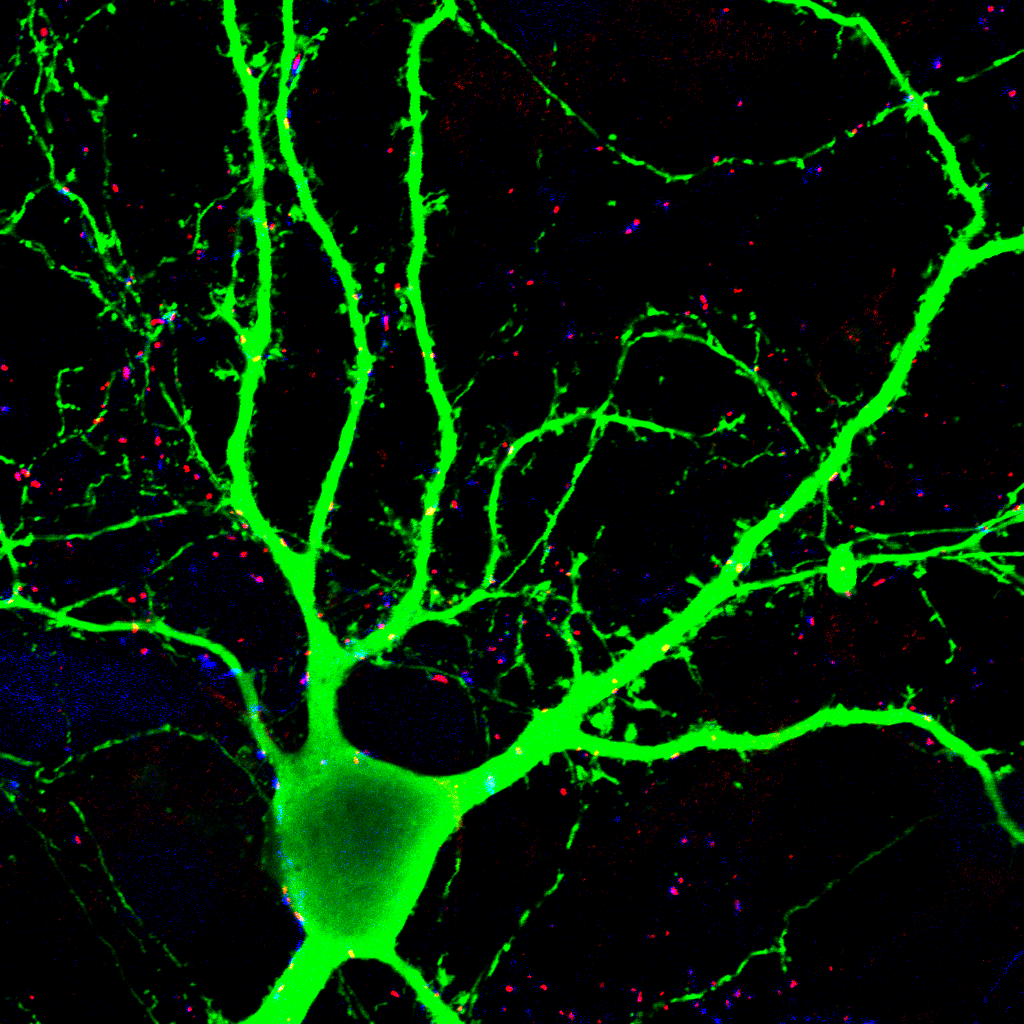

Supplement: Supplementary file 16 — Source data Fig. 2 [file 44321_2026_402_MOESM16_ESM.zip › Panel A and B/MDGA1 WT/MDGA1 WT (5).tif]

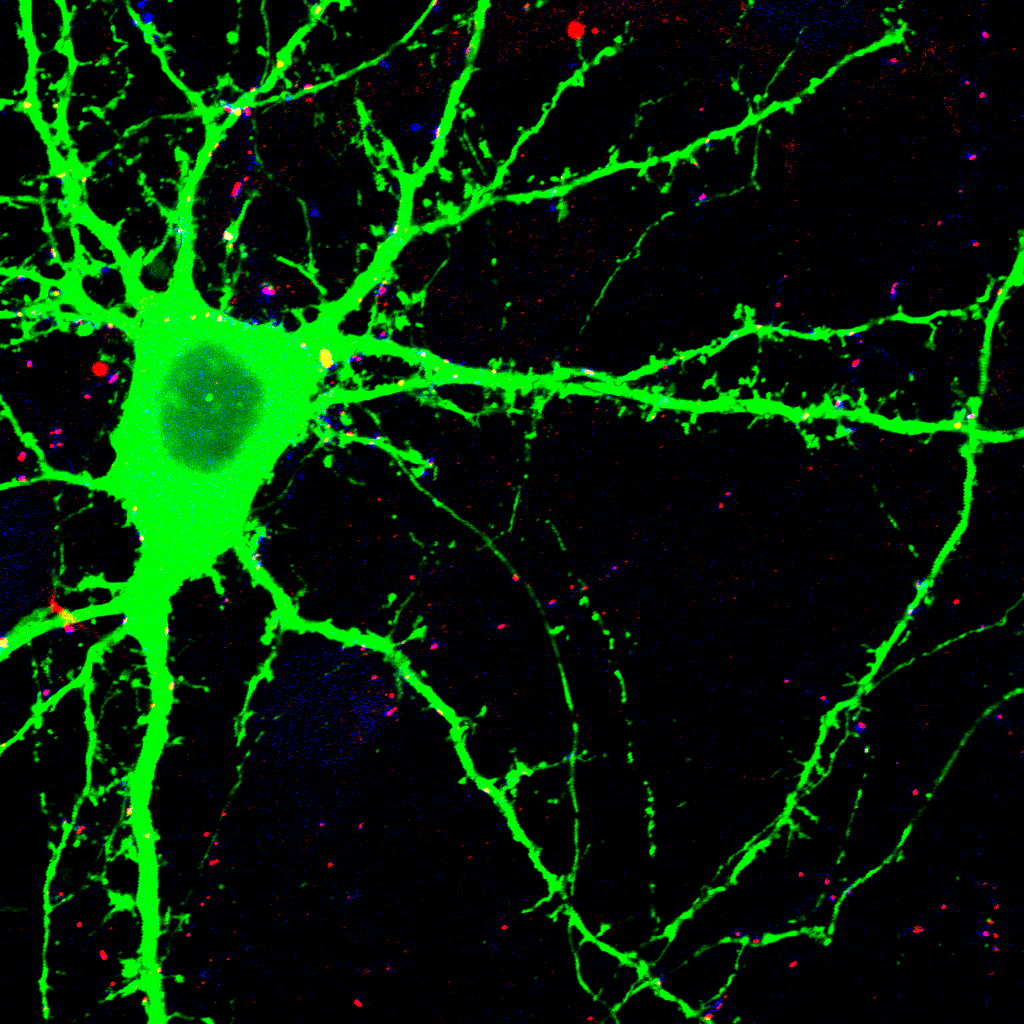

Supplement: Supplementary file 16 — Source data Fig. 2 [file 44321_2026_402_MOESM16_ESM.zip › Panel A and B/MDGA1 WT/MDGA1 WT (6).tif]

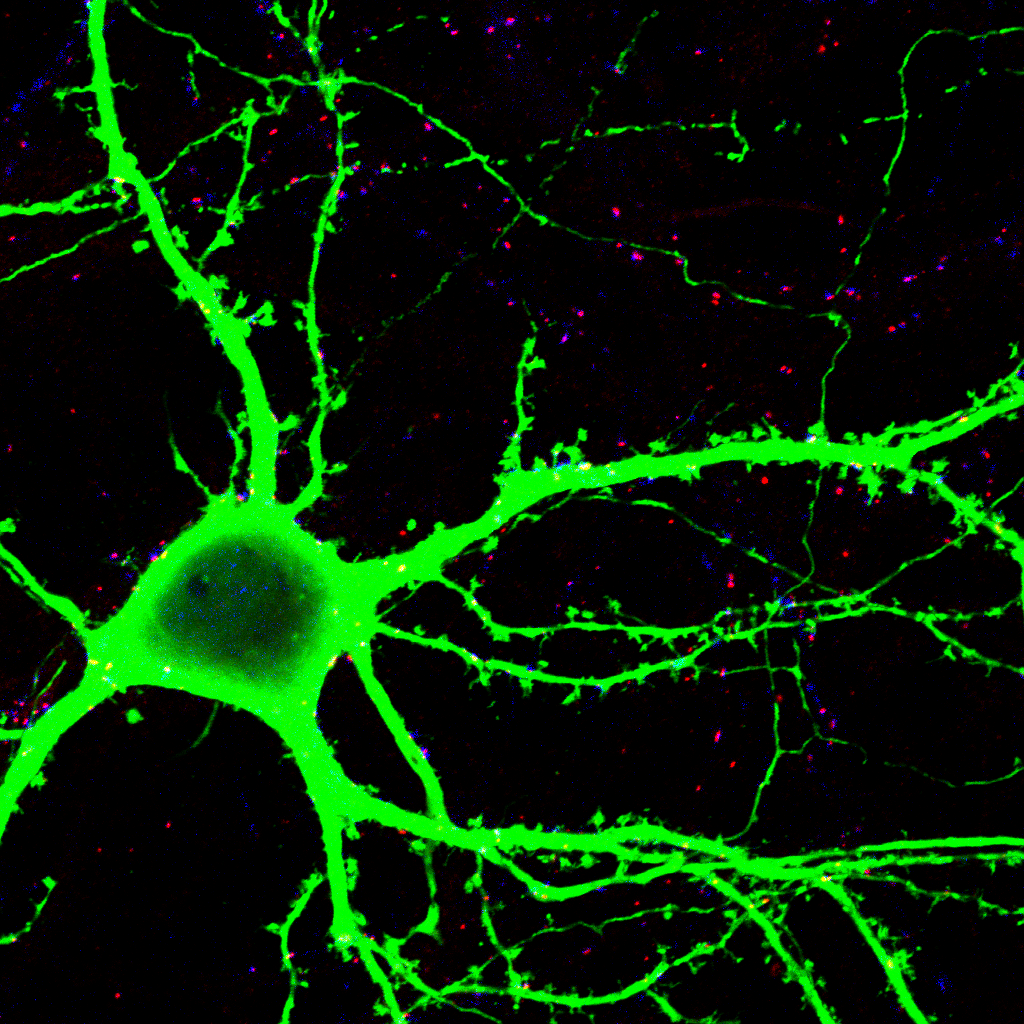

Supplement: Supplementary file 16 — Source data Fig. 2 [file 44321_2026_402_MOESM16_ESM.zip › Panel A and B/MDGA1 WT/MDGA1 WT (7).tif]

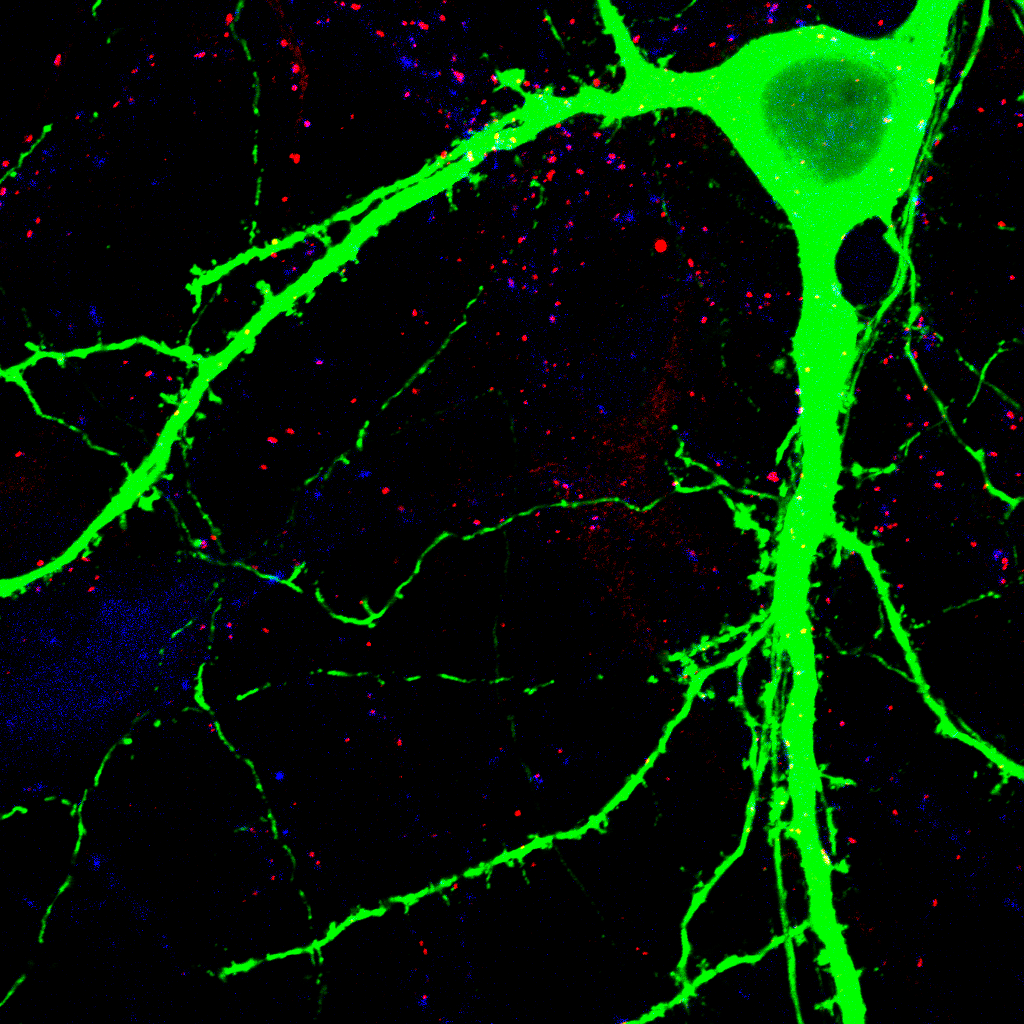

Supplement: Supplementary file 16 — Source data Fig. 2 [file 44321_2026_402_MOESM16_ESM.zip › Panel A and B/MDGA1 WT/MDGA1 WT (8).tif]

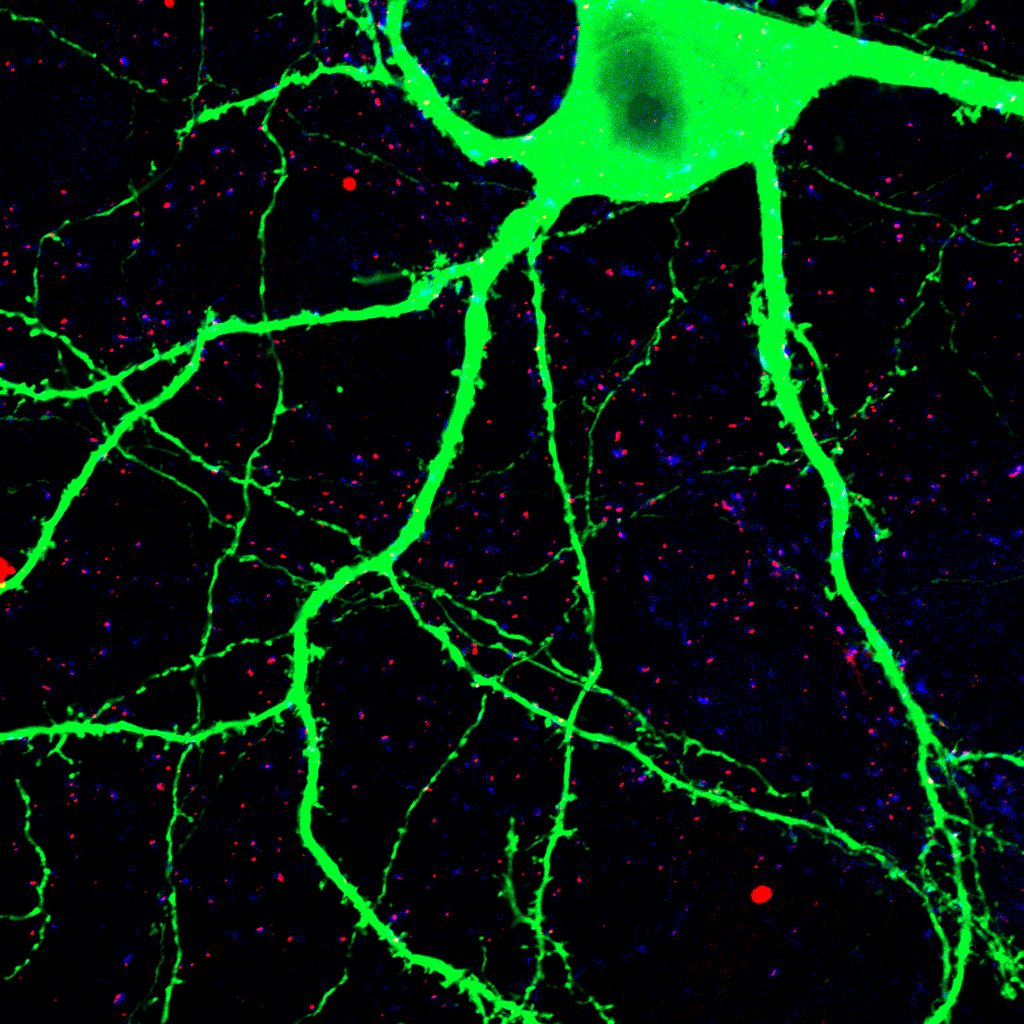

Supplement: Supplementary file 16 — Source data Fig. 2 [file 44321_2026_402_MOESM16_ESM.zip › Panel A and B/MDGA1 WT/MDGA1 WT (9).tif]

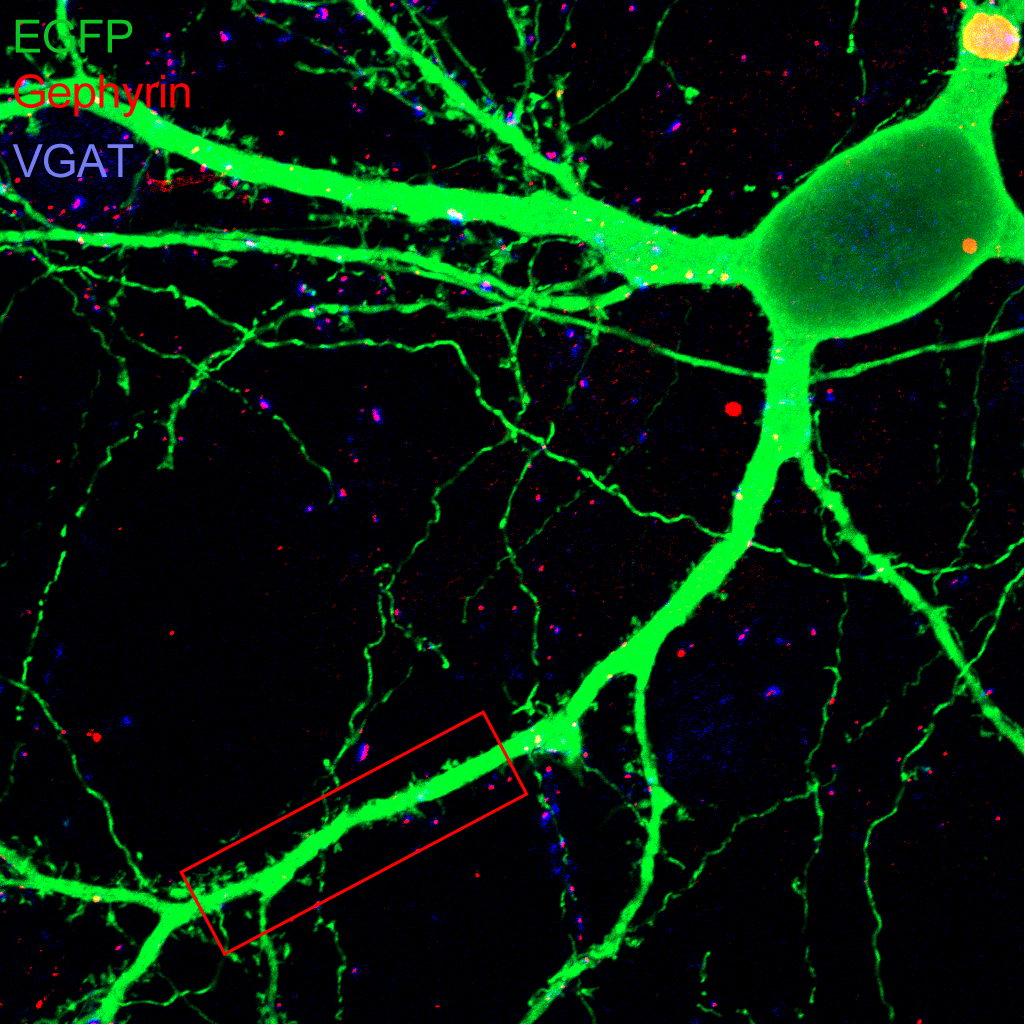

Supplement: Supplementary file 16 — Source data Fig. 2 [file 44321_2026_402_MOESM16_ESM.zip › Panel A and B/MDGA1 WT.tif]

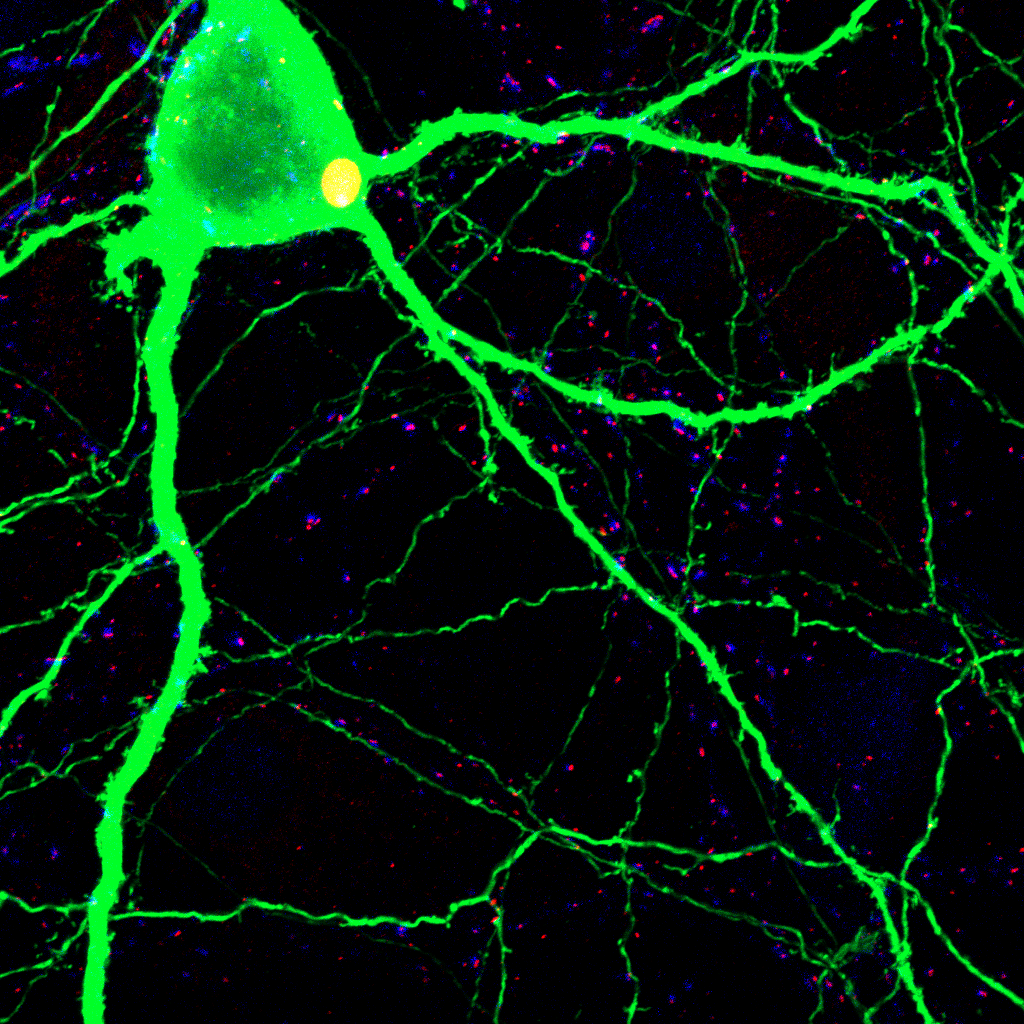

Supplement: Supplementary file 16 — Source data Fig. 2 [file 44321_2026_402_MOESM16_ESM.zip › Panel A and B/MDGA1 Y635C, E756Q/MDGA1 Y635C, E756Q (1).tif]

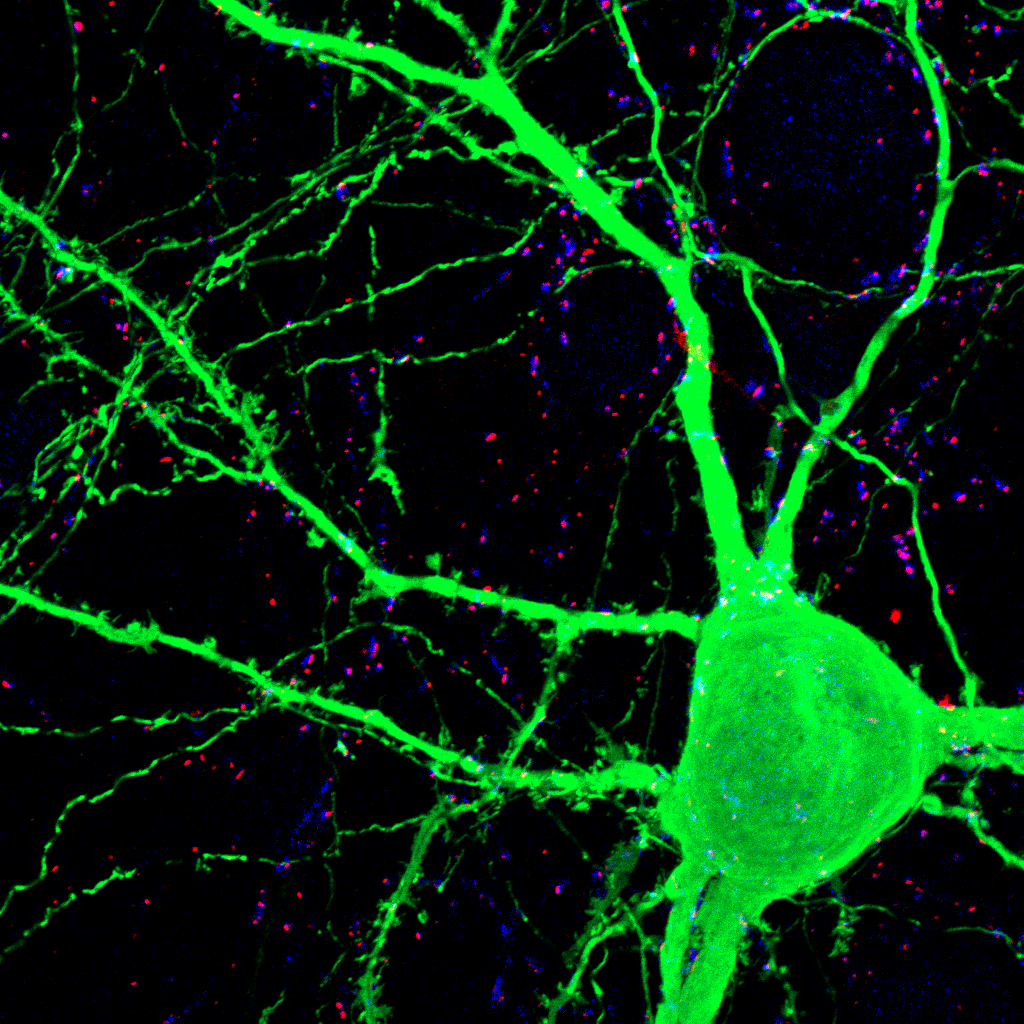

Supplement: Supplementary file 16 — Source data Fig. 2 [file 44321_2026_402_MOESM16_ESM.zip › Panel A and B/MDGA1 Y635C, E756Q/MDGA1 Y635C, E756Q (10).tif]

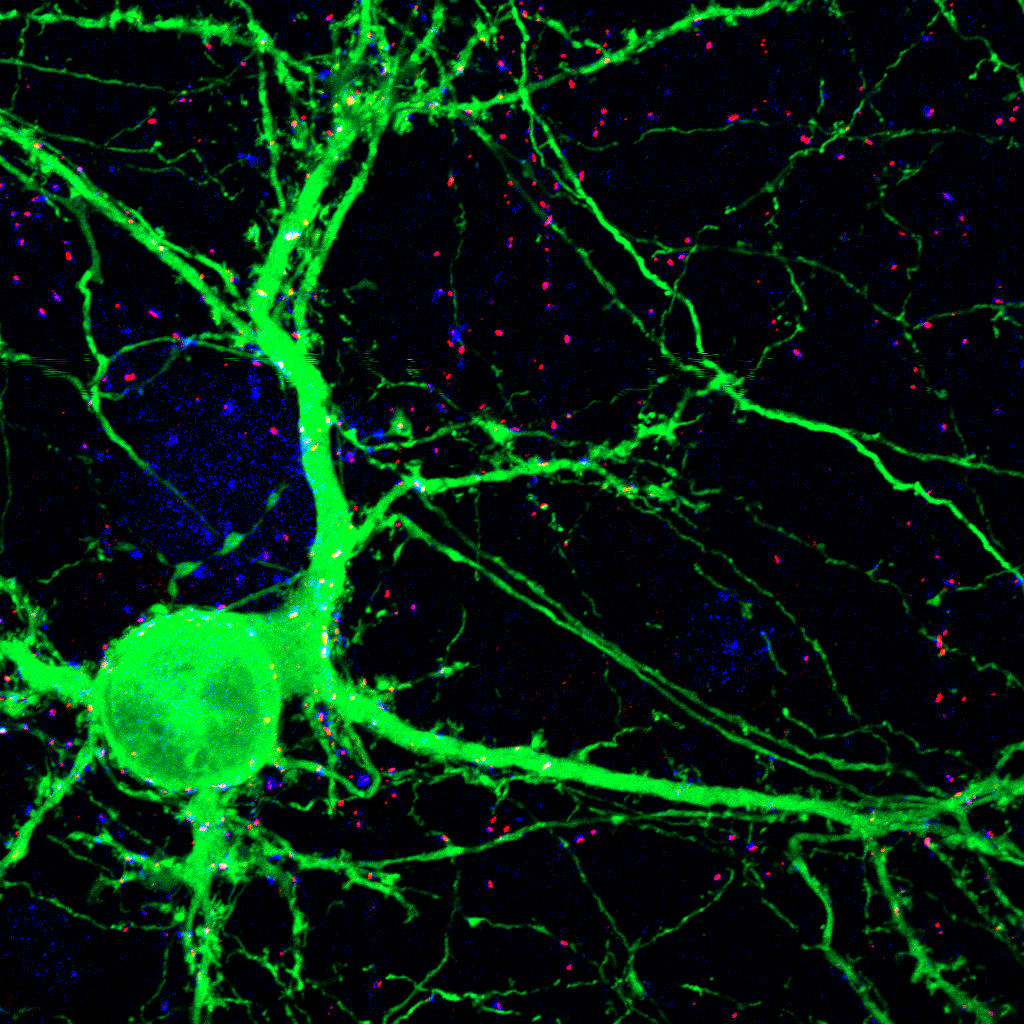

Supplement: Supplementary file 16 — Source data Fig. 2 [file 44321_2026_402_MOESM16_ESM.zip › Panel A and B/MDGA1 Y635C, E756Q/MDGA1 Y635C, E756Q (11).tif]

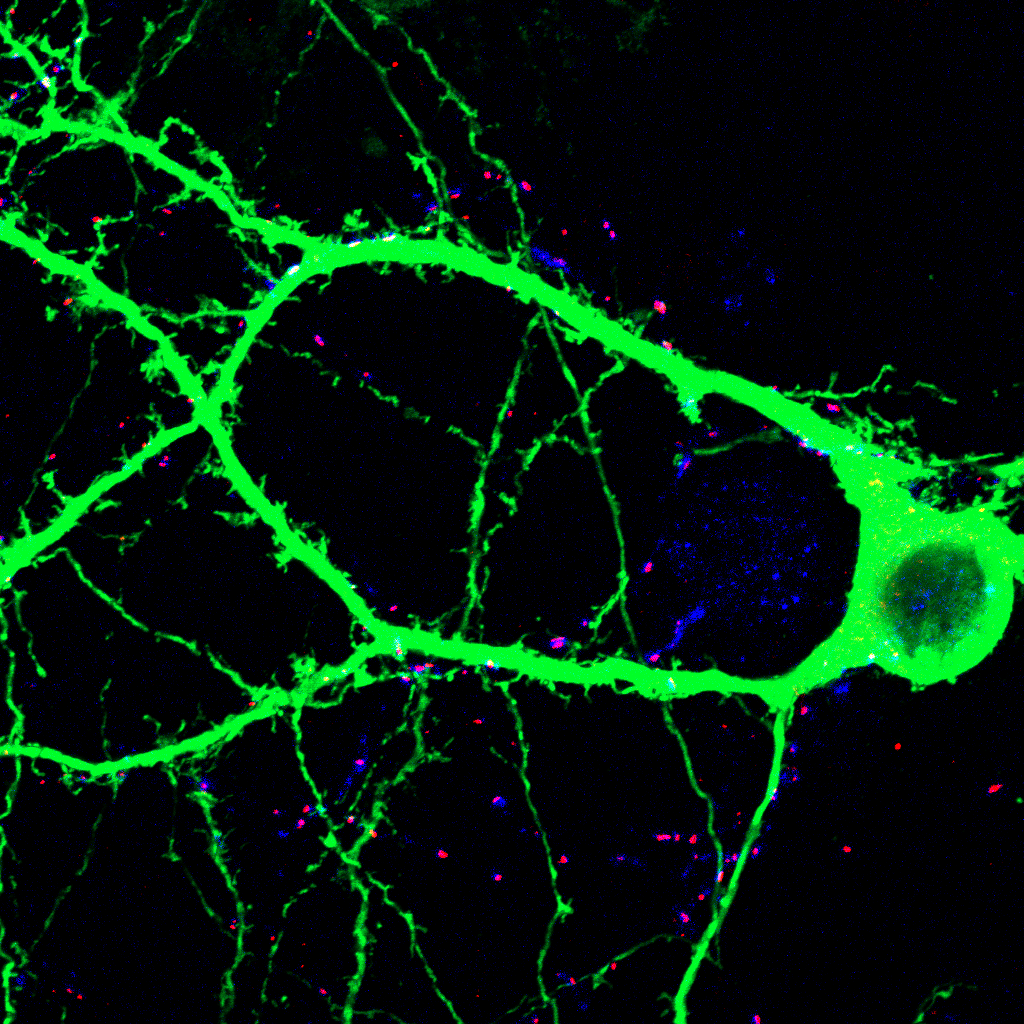

Supplement: Supplementary file 16 — Source data Fig. 2 [file 44321_2026_402_MOESM16_ESM.zip › Panel A and B/MDGA1 Y635C, E756Q/MDGA1 Y635C, E756Q (12).tif]

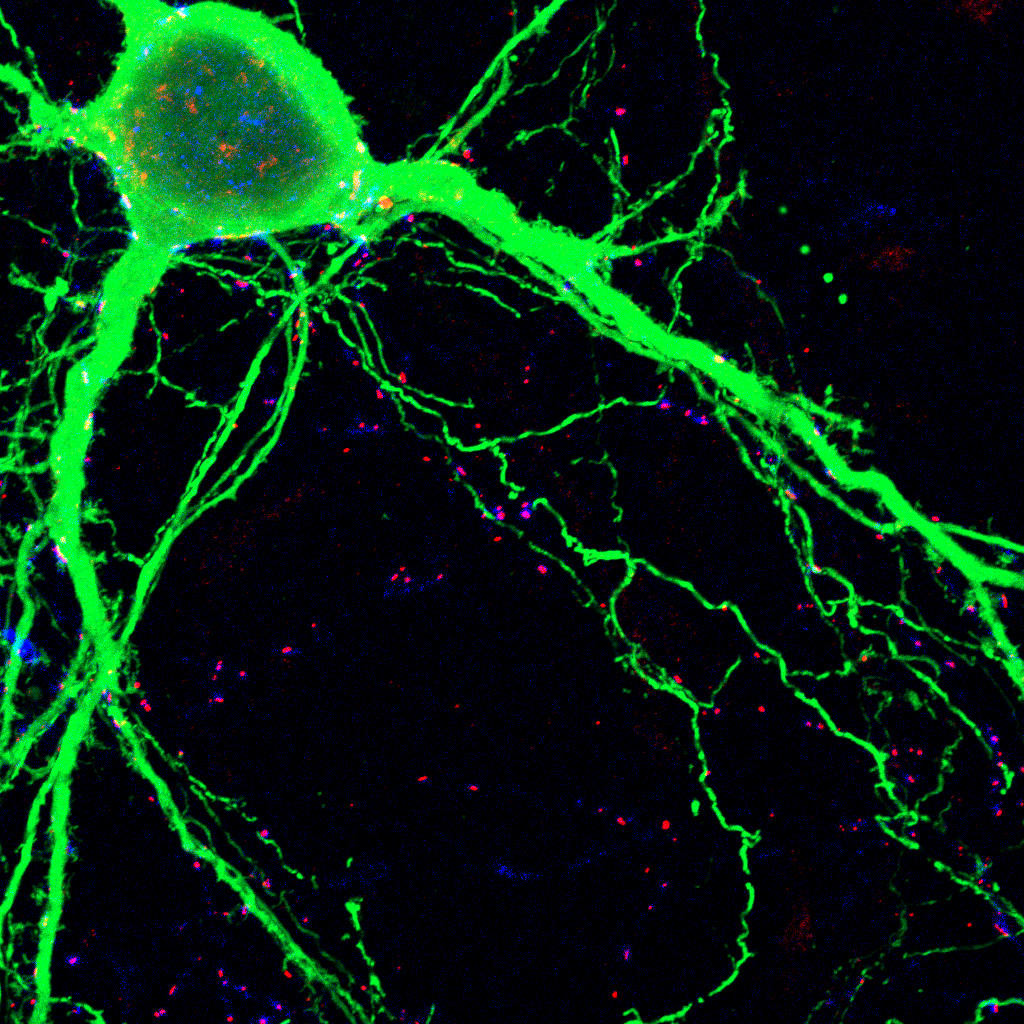

Supplement: Supplementary file 16 — Source data Fig. 2 [file 44321_2026_402_MOESM16_ESM.zip › Panel A and B/MDGA1 Y635C, E756Q/MDGA1 Y635C, E756Q (13).tif]

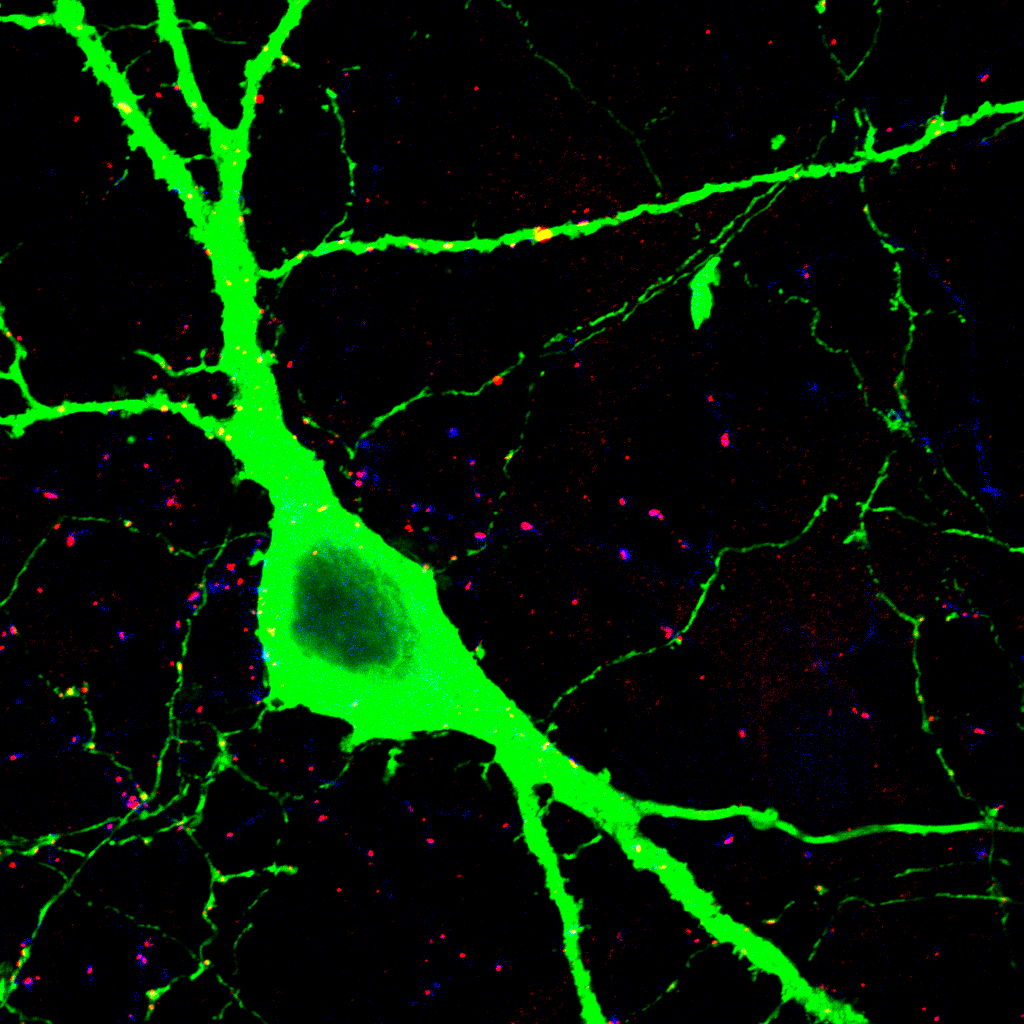

Supplement: Supplementary file 16 — Source data Fig. 2 [file 44321_2026_402_MOESM16_ESM.zip › Panel A and B/MDGA1 Y635C, E756Q/MDGA1 Y635C, E756Q (2).tif]

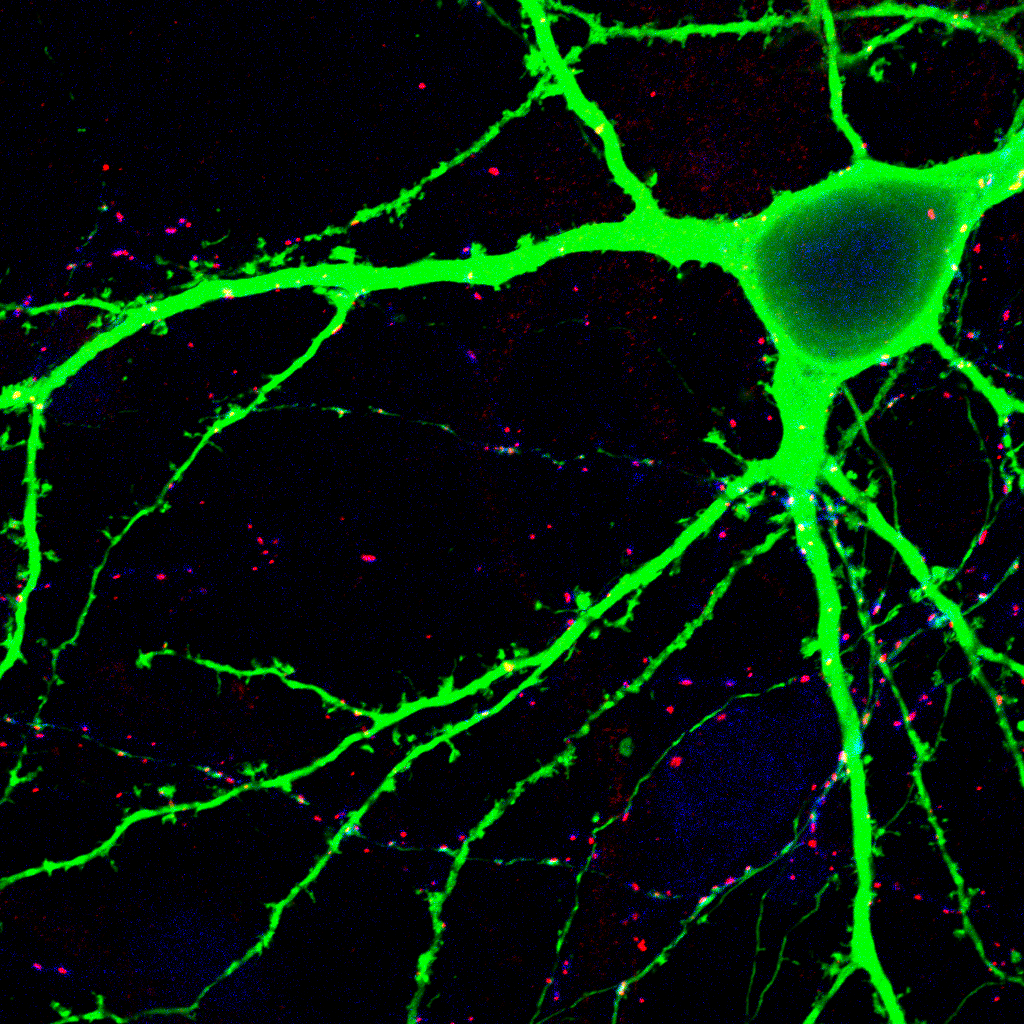

Supplement: Supplementary file 16 — Source data Fig. 2 [file 44321_2026_402_MOESM16_ESM.zip › Panel A and B/MDGA1 Y635C, E756Q/MDGA1 Y635C, E756Q (3).tif]

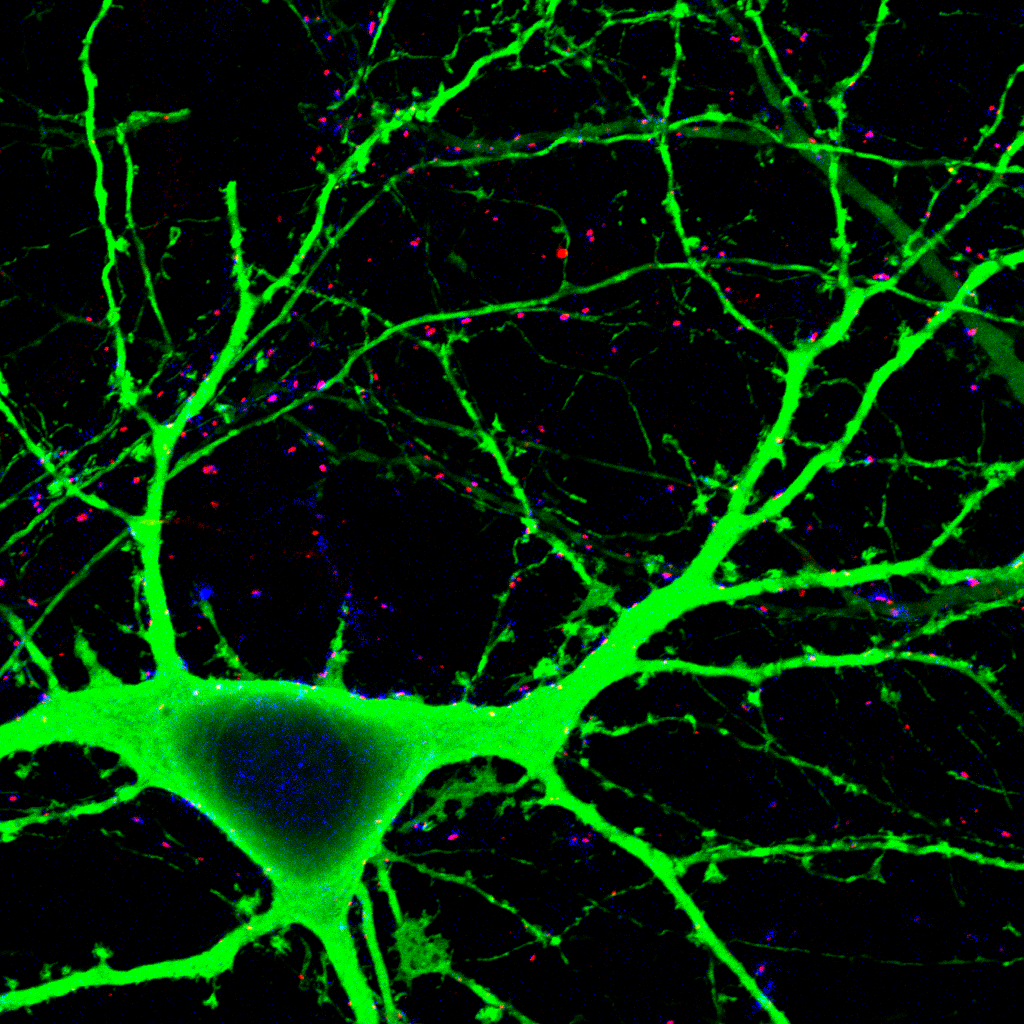

Supplement: Supplementary file 16 — Source data Fig. 2 [file 44321_2026_402_MOESM16_ESM.zip › Panel A and B/MDGA1 Y635C, E756Q/MDGA1 Y635C, E756Q (4).tif]

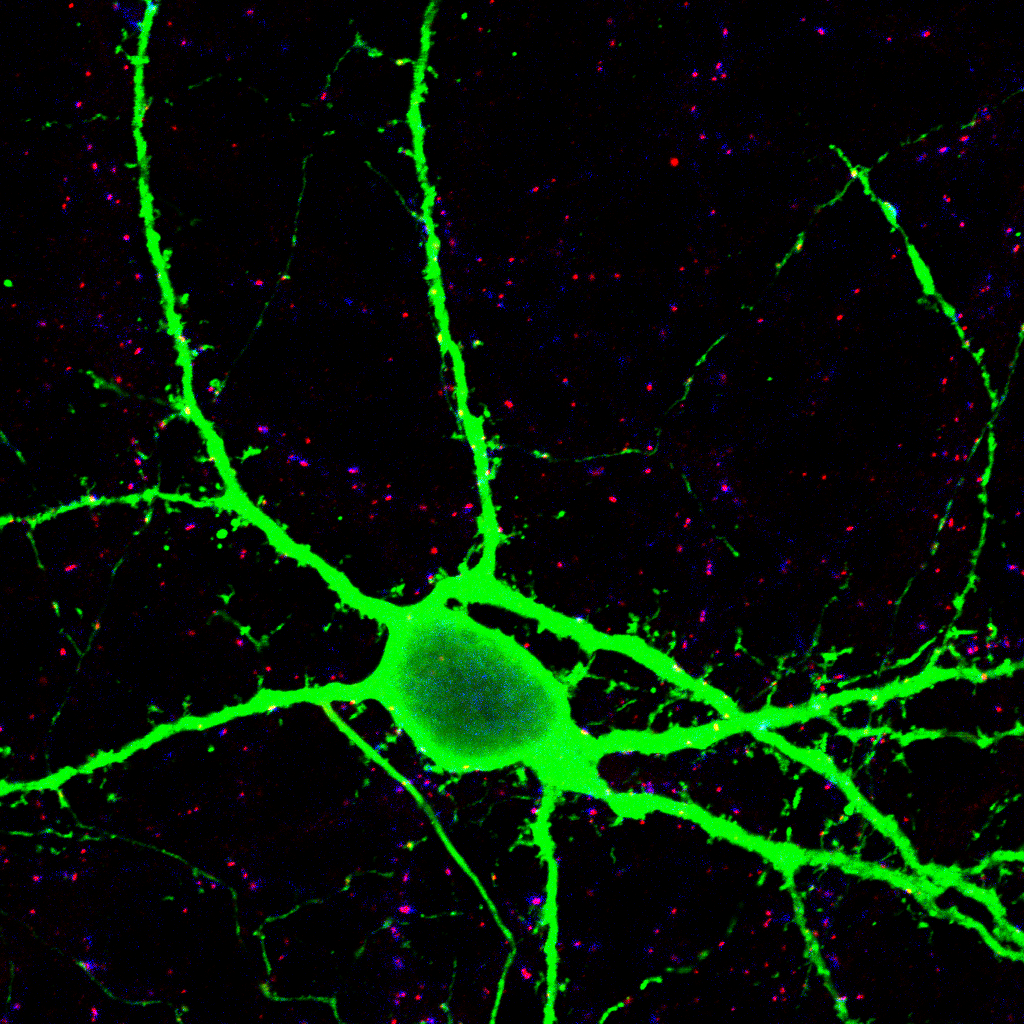

Supplement: Supplementary file 16 — Source data Fig. 2 [file 44321_2026_402_MOESM16_ESM.zip › Panel A and B/MDGA1 Y635C, E756Q/MDGA1 Y635C, E756Q (5).tif]

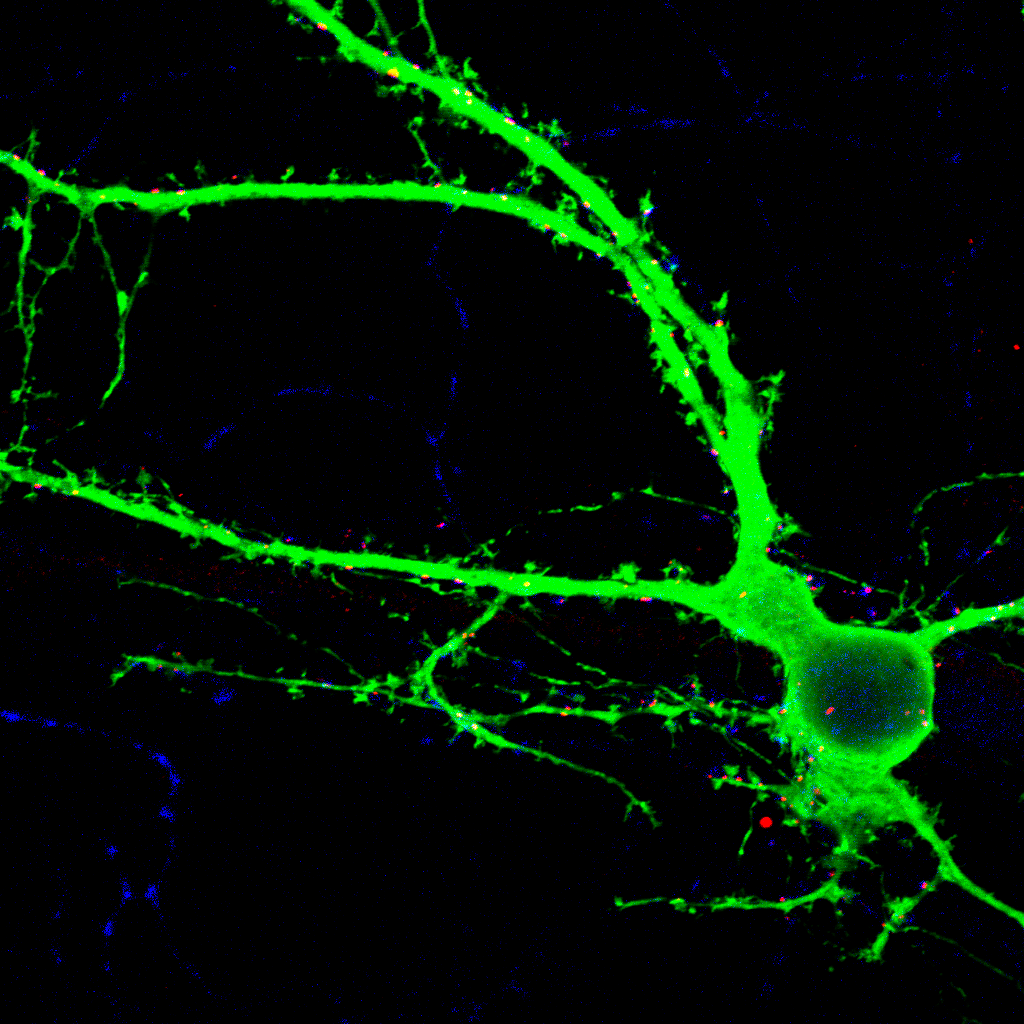

Supplement: Supplementary file 16 — Source data Fig. 2 [file 44321_2026_402_MOESM16_ESM.zip › Panel A and B/MDGA1 Y635C, E756Q/MDGA1 Y635C, E756Q (6).tif]

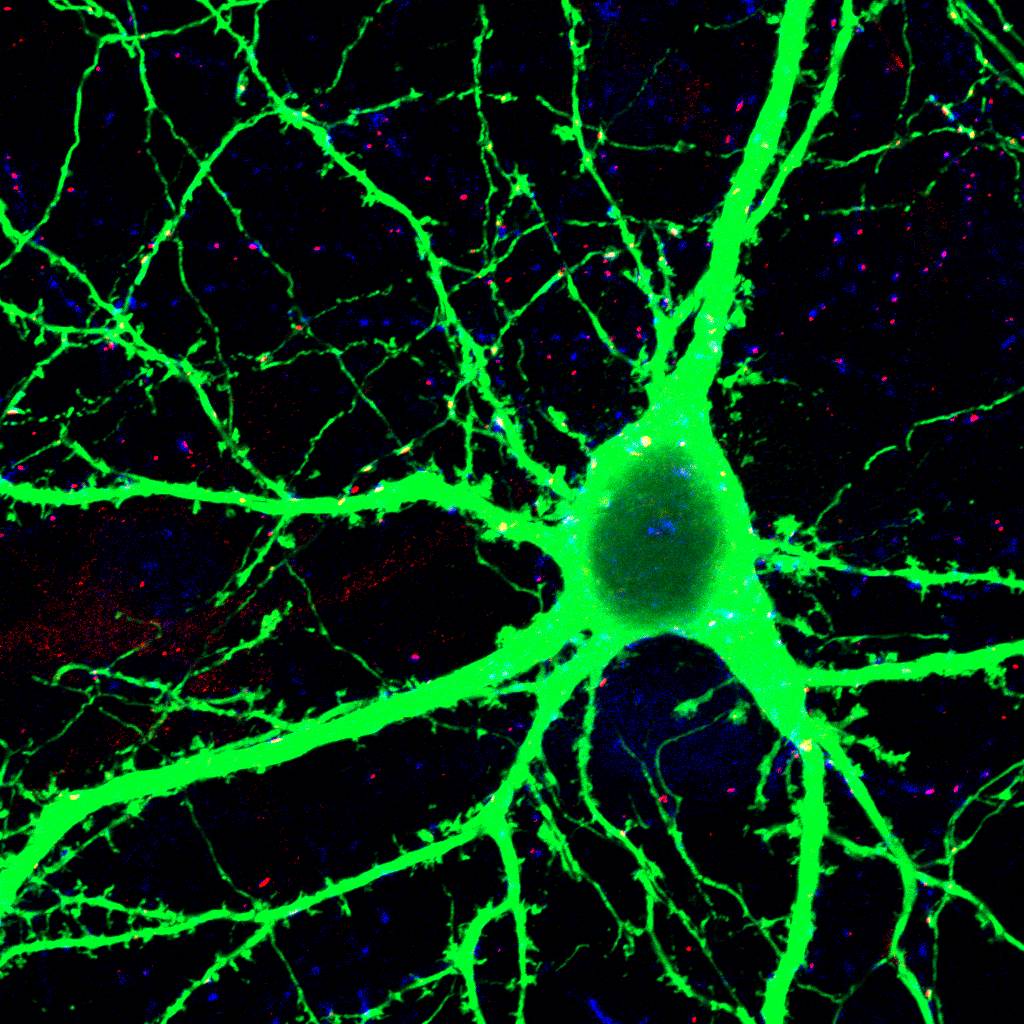

Supplement: Supplementary file 16 — Source data Fig. 2 [file 44321_2026_402_MOESM16_ESM.zip › Panel A and B/MDGA1 Y635C, E756Q/MDGA1 Y635C, E756Q (7).tif]

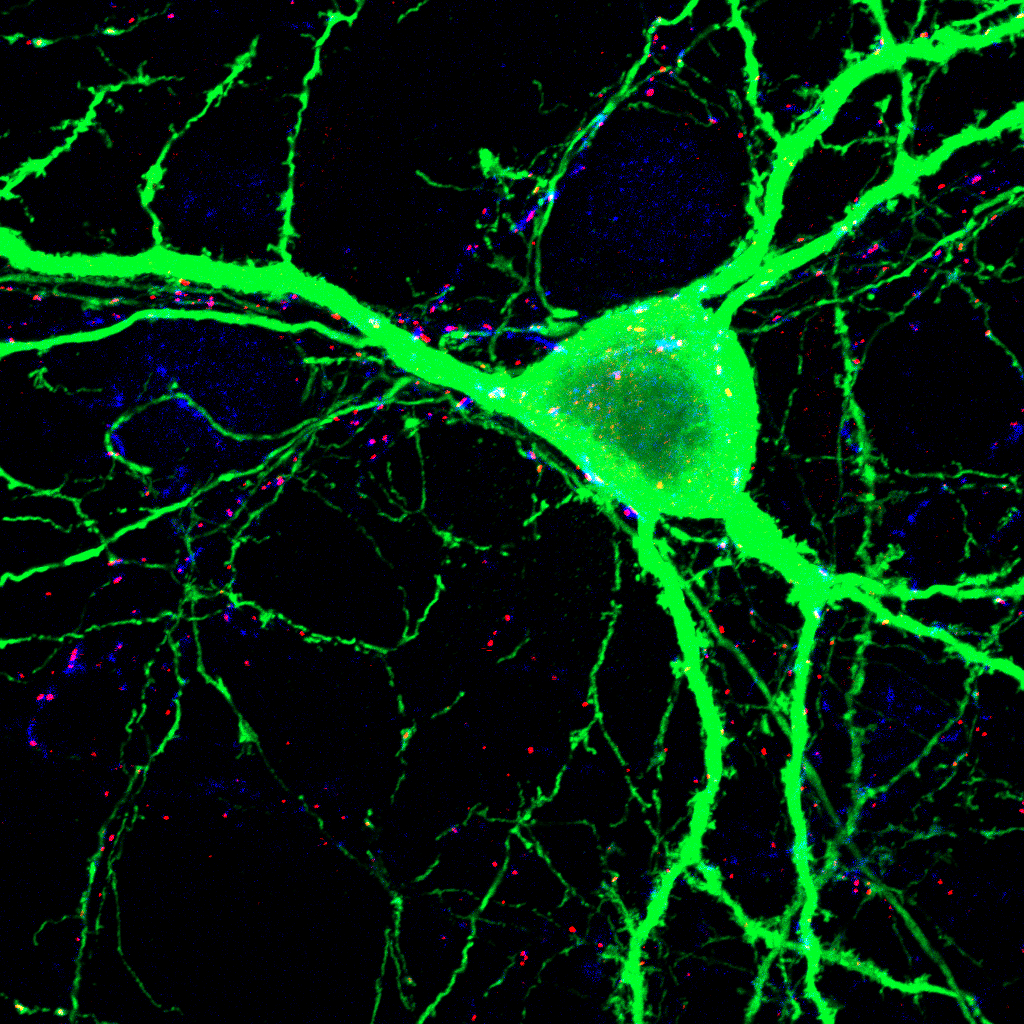

Supplement: Supplementary file 16 — Source data Fig. 2 [file 44321_2026_402_MOESM16_ESM.zip › Panel A and B/MDGA1 Y635C, E756Q/MDGA1 Y635C, E756Q (8).tif]

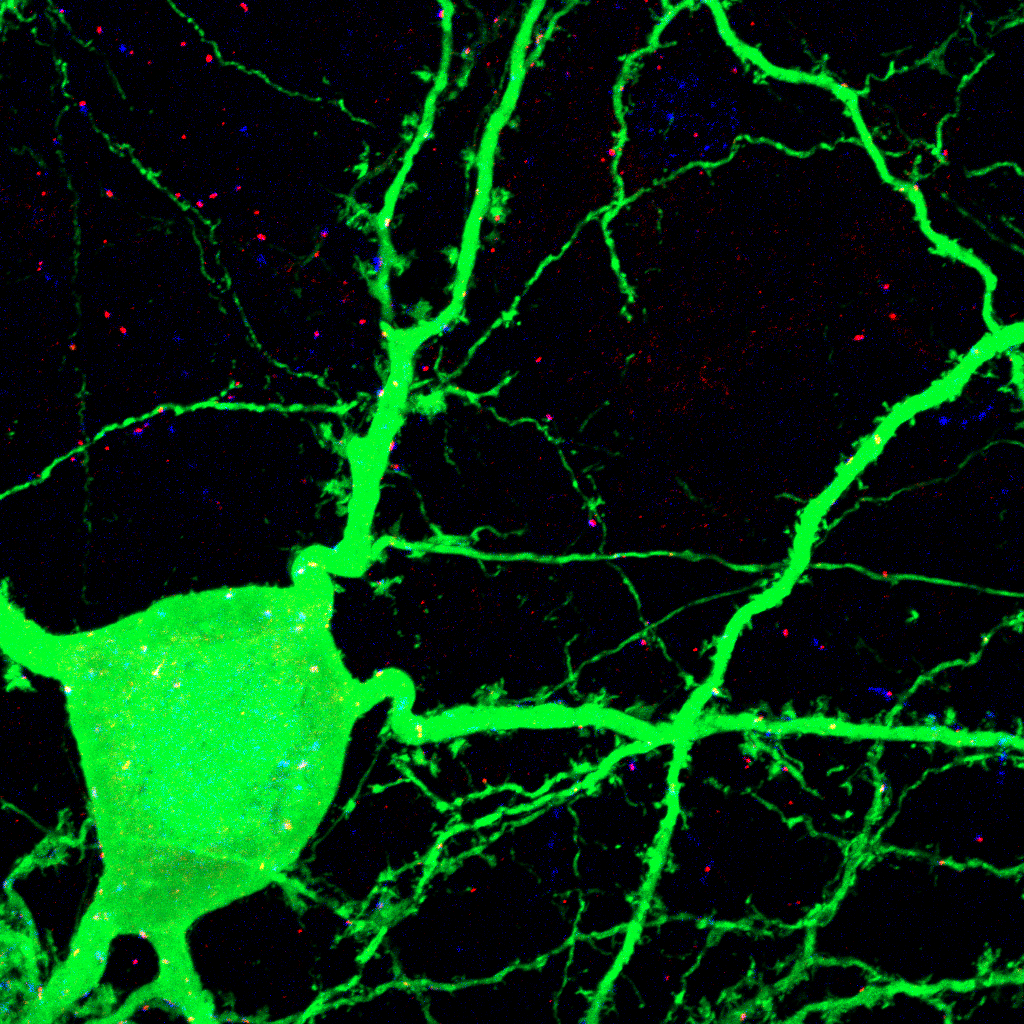

Supplement: Supplementary file 16 — Source data Fig. 2 [file 44321_2026_402_MOESM16_ESM.zip › Panel A and B/MDGA1 Y635C, E756Q/MDGA1 Y635C, E756Q (9).tif]

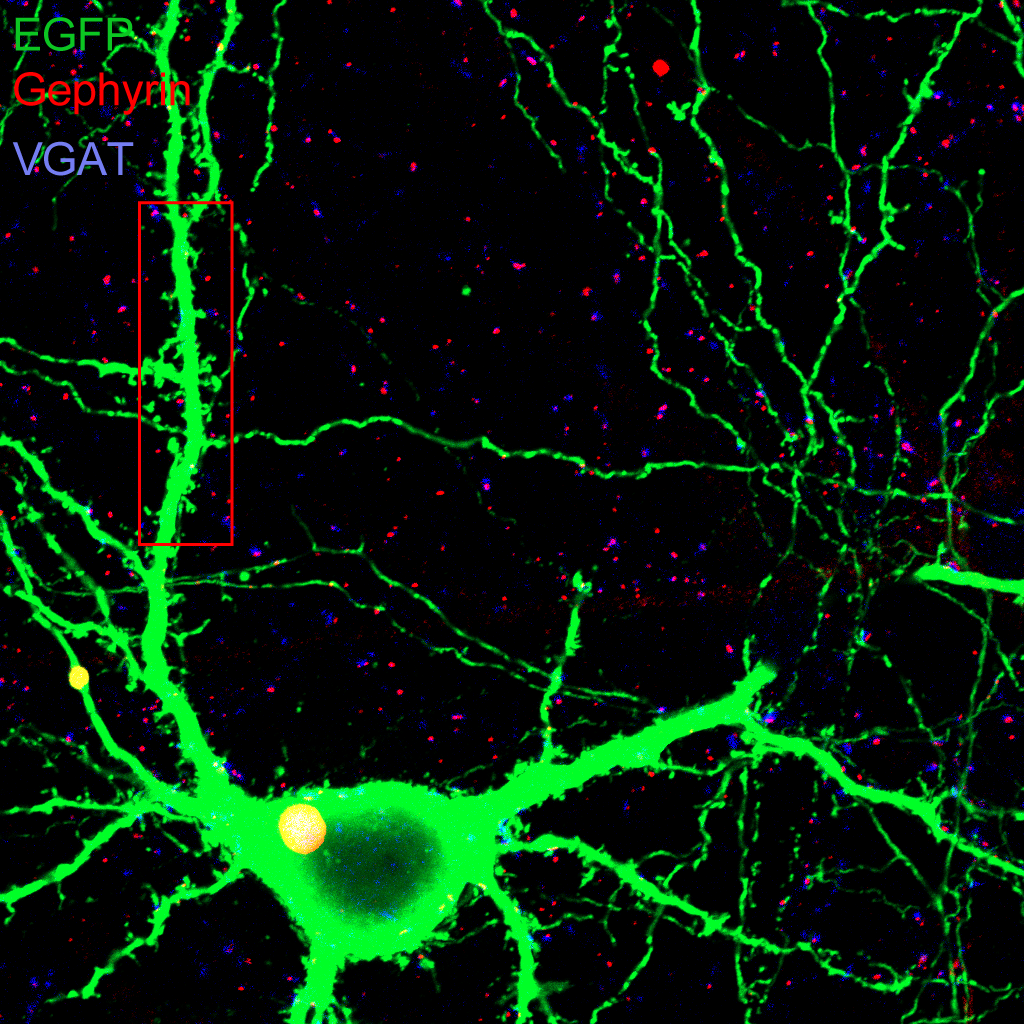

Supplement: Supplementary file 16 — Source data Fig. 2 [file 44321_2026_402_MOESM16_ESM.zip › Panel A and B/MDGA1 Y635C, E756Q.tif]

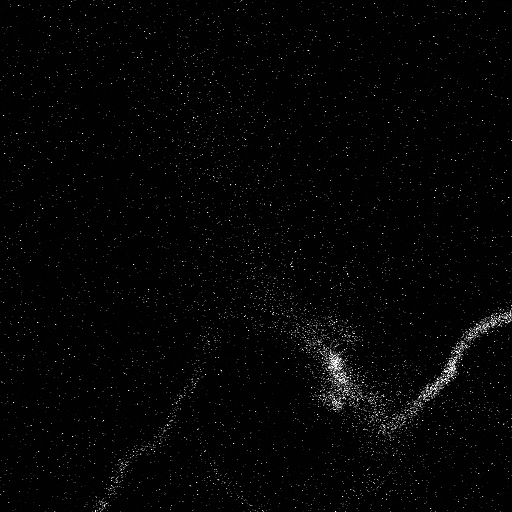

Supplement: Supplementary file 16 — Source data Fig. 2 [file 44321_2026_402_MOESM16_ESM.zip › Panel R-T/Control/Fig 1R Control.tif]

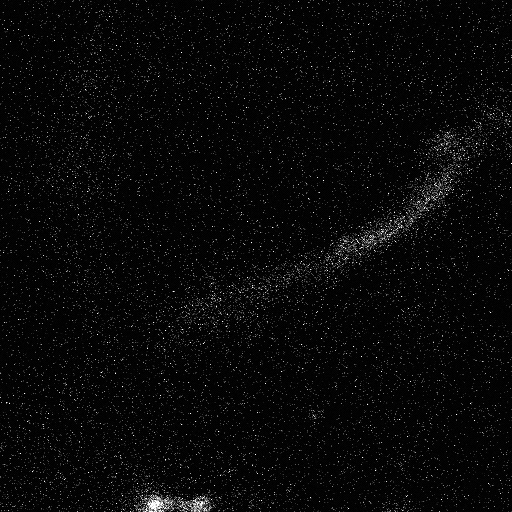

Supplement: Supplementary file 16 — Source data Fig. 2 [file 44321_2026_402_MOESM16_ESM.zip › Panel R-T/V116M A688V/Fig 1R V116M A688V.tif]

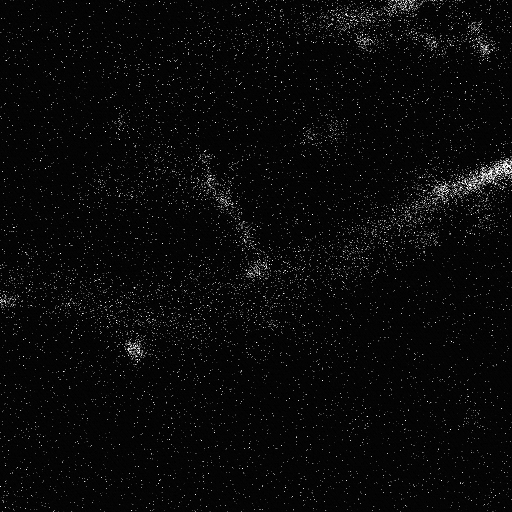

Supplement: Supplementary file 16 — Source data Fig. 2 [file 44321_2026_402_MOESM16_ESM.zip › Panel R-T/WT/Fig 1R WT.tif]

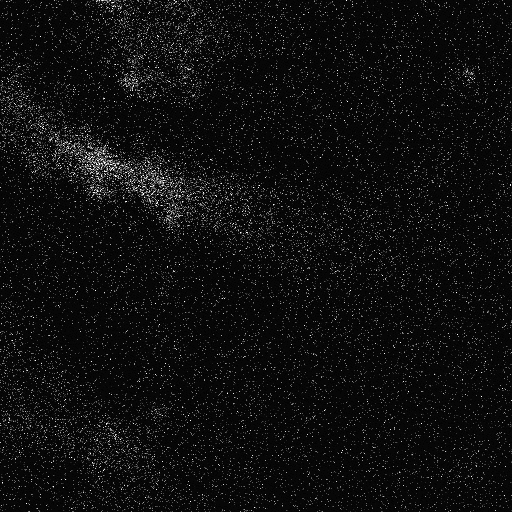

Supplement: Supplementary file 16 — Source data Fig. 2 [file 44321_2026_402_MOESM16_ESM.zip › Panel R-T/Y635C E756Q/Fig 1R Y635C E756Q.tif]

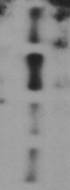

Supplement: Supplementary file 20 — Source data Fig. 7 [file 44321_2026_402_MOESM20_ESM.zip › Panel B/Figure7_PanelB_Blot_replicate1_GABARr2.jpg]

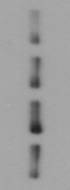

Supplement: Supplementary file 20 — Source data Fig. 7 [file 44321_2026_402_MOESM20_ESM.zip › Panel B/Figure7_PanelB_Blot_replicate1_Gephyrin.jpg]

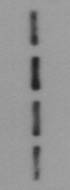

Supplement: Supplementary file 20 — Source data Fig. 7 [file 44321_2026_402_MOESM20_ESM.zip › Panel B/Figure7_PanelB_Blot_replicate1_NL2.jpg]

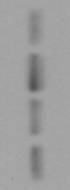

Supplement: Supplementary file 20 — Source data Fig. 7 [file 44321_2026_402_MOESM20_ESM.zip › Panel B/Figure7_PanelB_Blot_replicate1_VGAT.jpg]

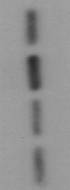

Supplement: Supplementary file 20 — Source data Fig. 7 [file 44321_2026_402_MOESM20_ESM.zip › Panel B/Figure7_PanelB_Blot_replicate2_GABARr2.jpg]

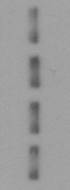

Supplement: Supplementary file 20 — Source data Fig. 7 [file 44321_2026_402_MOESM20_ESM.zip › Panel B/Figure7_PanelB_Blot_replicate2_Gephyrin.jpg]

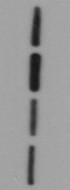

Supplement: Supplementary file 20 — Source data Fig. 7 [file 44321_2026_402_MOESM20_ESM.zip › Panel B/Figure7_PanelB_Blot_replicate2_NL2.jpg]

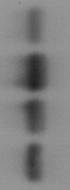

Supplement: Supplementary file 20 — Source data Fig. 7 [file 44321_2026_402_MOESM20_ESM.zip › Panel B/Figure7_PanelB_Blot_replicate2_VGAT.jpg]

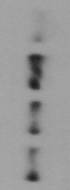

Supplement: Supplementary file 20 — Source data Fig. 7 [file 44321_2026_402_MOESM20_ESM.zip › Panel B/Figure7_PanelB_Blot_replicate3_GABARr2.jpg]

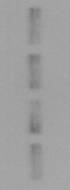

Supplement: Supplementary file 20 — Source data Fig. 7 [file 44321_2026_402_MOESM20_ESM.zip › Panel B/Figure7_PanelB_Blot_replicate3_Gephyrin.jpg]

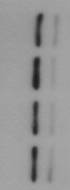

Supplement: Supplementary file 20 — Source data Fig. 7 [file 44321_2026_402_MOESM20_ESM.zip › Panel B/Figure7_PanelB_Blot_replicate3_NL2.jpg]

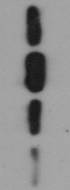

Supplement: Supplementary file 20 — Source data Fig. 7 [file 44321_2026_402_MOESM20_ESM.zip › Panel B/Figure7_PanelB_Blot_replicate3_VGAT.jpg]

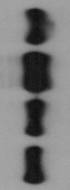

Supplement: Supplementary file 20 — Source data Fig. 7 [file 44321_2026_402_MOESM20_ESM.zip › Panel B/Figure7_PanelB_Blot_replicate4_GABARr2.jpg]

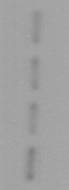

Supplement: Supplementary file 20 — Source data Fig. 7 [file 44321_2026_402_MOESM20_ESM.zip › Panel B/Figure7_PanelB_Blot_replicate4_Gephyrin.jpg]

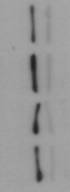

Supplement: Supplementary file 20 — Source data Fig. 7 [file 44321_2026_402_MOESM20_ESM.zip › Panel B/Figure7_PanelB_Blot_replicate4_NL2.jpg]

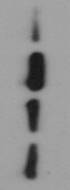

Supplement: Supplementary file 20 — Source data Fig. 7 [file 44321_2026_402_MOESM20_ESM.zip › Panel B/Figure7_PanelB_Blot_replicate4_VGAT.jpg]

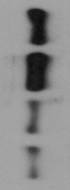

Supplement: Supplementary file 20 — Source data Fig. 7 [file 44321_2026_402_MOESM20_ESM.zip › Panel B/Figure7_PanelB_Blot_replicate5_GABARr2.jpg]

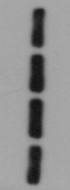

Supplement: Supplementary file 20 — Source data Fig. 7 [file 44321_2026_402_MOESM20_ESM.zip › Panel B/Figure7_PanelB_Blot_replicate5_Gephyrin.jpg]

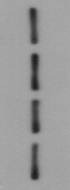

Supplement: Supplementary file 20 — Source data Fig. 7 [file 44321_2026_402_MOESM20_ESM.zip › Panel B/Figure7_PanelB_Blot_replicate5_NL2.jpg]

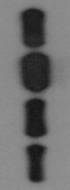

Supplement: Supplementary file 20 — Source data Fig. 7 [file 44321_2026_402_MOESM20_ESM.zip › Panel B/Figure7_PanelB_Blot_replicate5_VGAT.jpg]

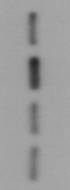

Supplement: Supplementary file 20 — Source data Fig. 7 [file 44321_2026_402_MOESM20_ESM.zip › Panel B/Figure7_PanelB_Blot_representative_ NL2.jpg]

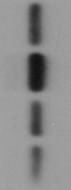

Supplement: Supplementary file 20 — Source data Fig. 7 [file 44321_2026_402_MOESM20_ESM.zip › Panel B/Figure7_PanelB_Blot_representative_GABARr2.jpg]

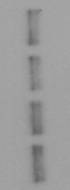

Supplement: Supplementary file 20 — Source data Fig. 7 [file 44321_2026_402_MOESM20_ESM.zip › Panel B/Figure7_PanelB_Blot_representative_Gephyrin.jpg]

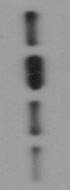

Supplement: Supplementary file 20 — Source data Fig. 7 [file 44321_2026_402_MOESM20_ESM.zip › Panel B/Figure7_PanelB_Blot_representative_VGAT.jpg]

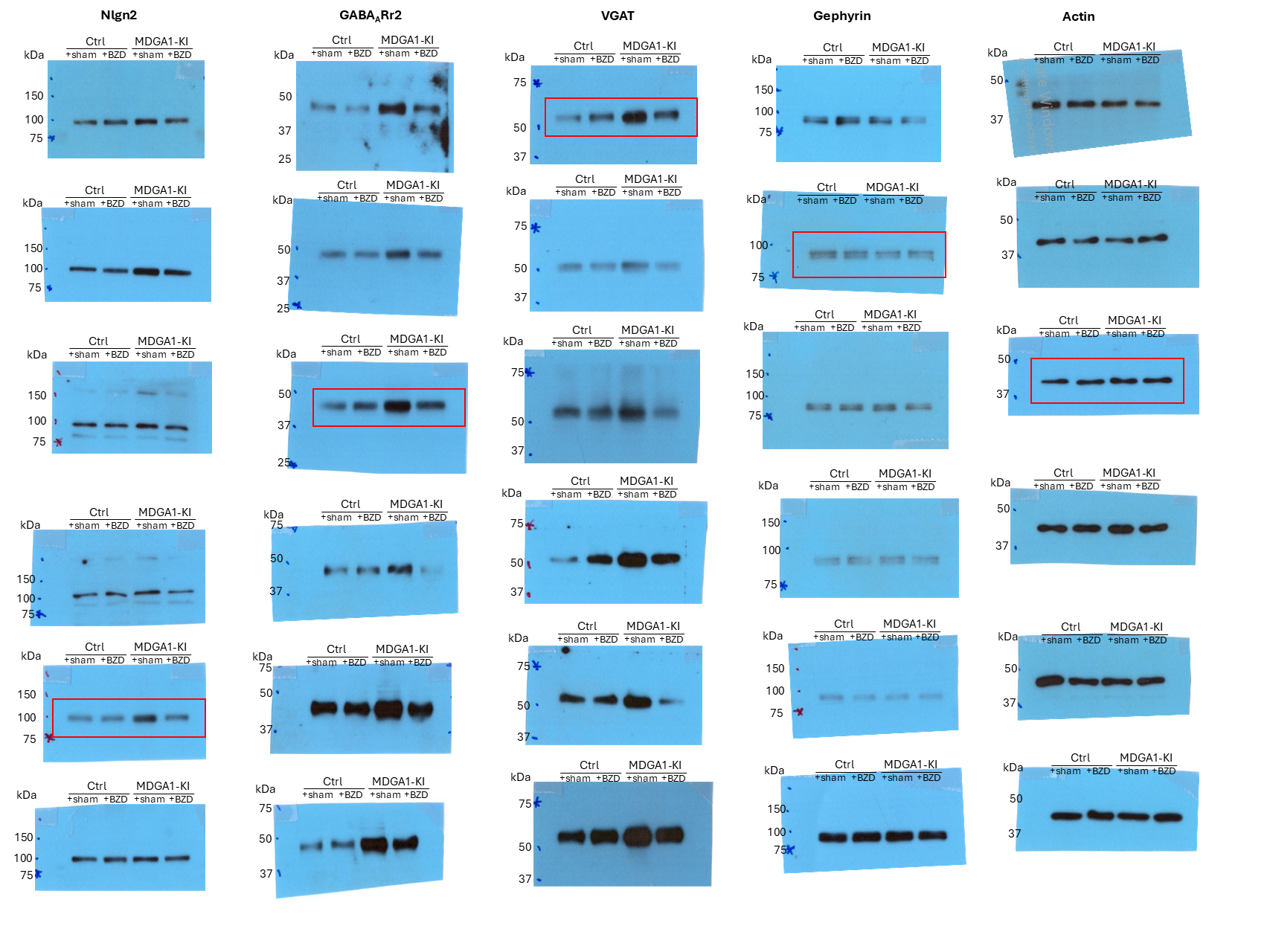

Supplement: Supplementary file 20 — Source data Fig. 7 [file 44321_2026_402_MOESM20_ESM.zip › Panel B/Figure7_PanelB_Crop_Annotation.TIF]

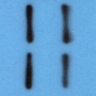

Supplement: Supplementary file 20 — Source data Fig. 7 [file 44321_2026_402_MOESM20_ESM.zip › Panel D/(X)Figure7_PanelD_Blot_representative_Syn2_IP(female).jpg]

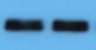

Supplement: Supplementary file 20 — Source data Fig. 7 [file 44321_2026_402_MOESM20_ESM.zip › Panel D/Figure7_PanelD_Blot_representative_ Syn1_input(female).jpg]

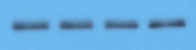

Supplement: Supplementary file 20 — Source data Fig. 7 [file 44321_2026_402_MOESM20_ESM.zip › Panel D/Figure7_PanelD_Blot_representative_Syn1_input(male).jpg]

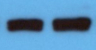

Supplement: Supplementary file 20 — Source data Fig. 7 [file 44321_2026_402_MOESM20_ESM.zip › Panel D/Figure7_PanelD_Blot_representative_Syn1_IP(female).jpg]

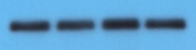

Supplement: Supplementary file 20 — Source data Fig. 7 [file 44321_2026_402_MOESM20_ESM.zip › Panel D/Figure7_PanelD_Blot_representative_Syn1_IP(male).jpg]

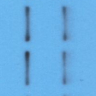

Supplement: Supplementary file 20 — Source data Fig. 7 [file 44321_2026_402_MOESM20_ESM.zip › Panel D/Figure7_PanelD_Blot_representative_Syn2_input(female).jpg]

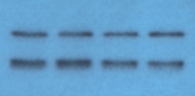

Supplement: Supplementary file 20 — Source data Fig. 7 [file 44321_2026_402_MOESM20_ESM.zip › Panel D/Figure7_PanelD_Blot_representative_Syn2_input(male).jpg]

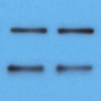

Supplement: Supplementary file 20 — Source data Fig. 7 [file 44321_2026_402_MOESM20_ESM.zip › Panel D/Figure7_PanelD_Blot_representative_Syn2_IP(female).jpg]

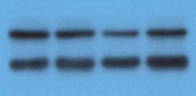

Supplement: Supplementary file 20 — Source data Fig. 7 [file 44321_2026_402_MOESM20_ESM.zip › Panel D/Figure7_PanelD_Blot_representative_Syn2_IP(male).jpg]

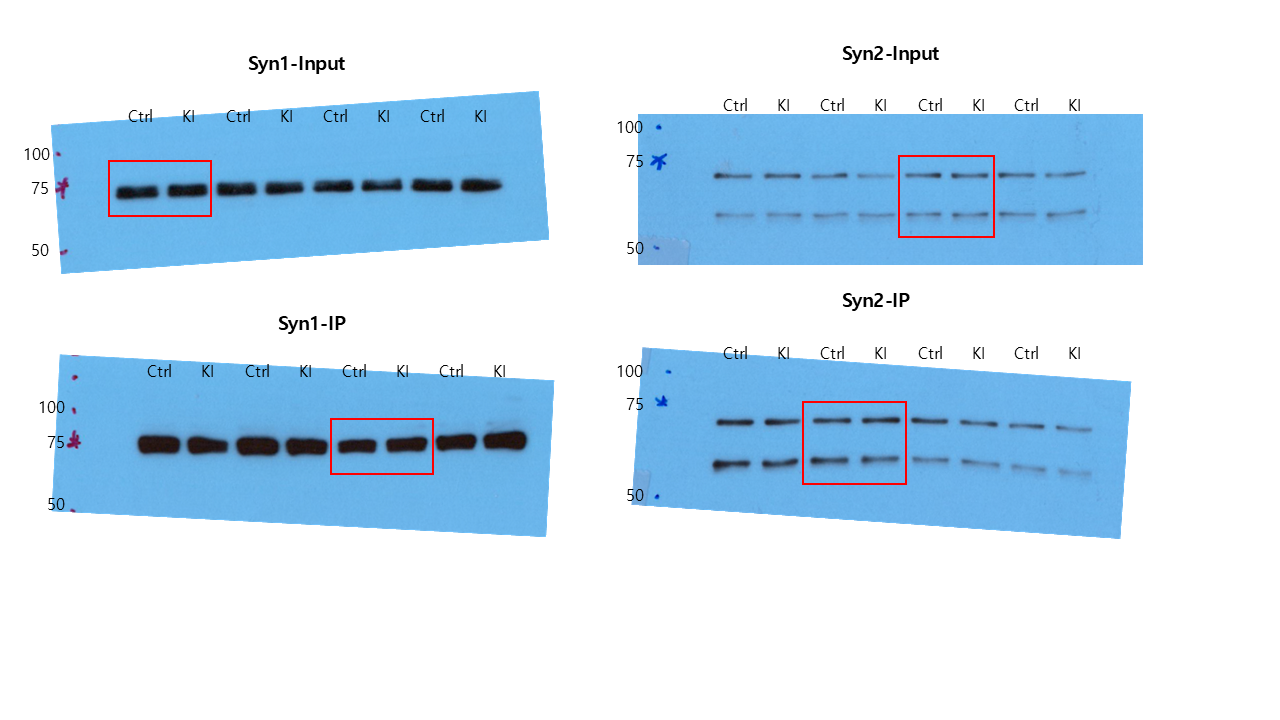

Supplement: Supplementary file 20 — Source data Fig. 7 [file 44321_2026_402_MOESM20_ESM.zip › Panel D/Figure7_PanelD_Crop_Annotation(female).TIF]

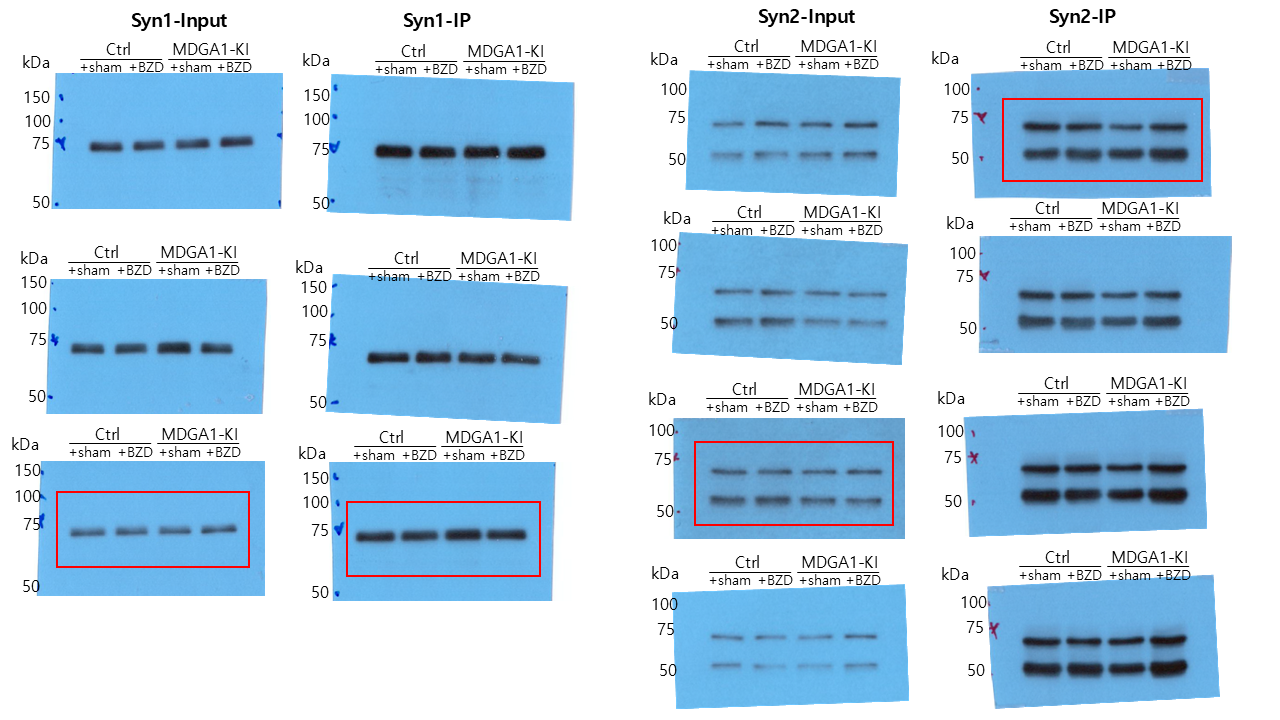

Supplement: Supplementary file 20 — Source data Fig. 7 [file 44321_2026_402_MOESM20_ESM.zip › Panel D/Figure7_PanelD_Crop_Annotation(male).TIF]
