# Supplementary material for: Bazedoxifene reverses sexually dimorphic autistic-like abnormalities in biallelic MDGA1-mutant mice
Source: EMBO Mol Med. 2026 Mar 20;18(4):1358–98. doi: 10.1038/s44321-026-00402-y (PMC13084050; doi:10.1038/s44321-026-00402-y)
Supplement: Supplementary file 19 — Source data Fig. 5 [file 44321_2026_402_MOESM19_ESM.zip › Figure 5.docx]

**Figure 5D**

**Control**

File name: 2023_09_28_0015.abf

Cropped trace timepoint (ms): 96.4012 – 98.4012

**KI**

File name: 2023_09_29_0017.abf

Cropped trace timepoint (ms): 156.78337 – 158.78337

**Figure 5H**

**Control**

File name: 2023_10_09_0012.abf

Cropped trace timepoint (ms): 77.15913 – 79.15913

**KI**

File name: 2023_10_09_0022.abf

Cropped trace timepoint (ms): 171.56805 – 173.56805

**Figure 5L**

**Control**

File name: 2023_07_27_0016.abf

Cropped trace timepoint (ms): 95.26622 – 97.26622

**KI**

File name: 2023_07_27_0009.abf

Cropped trace timepoint (ms): 105.09683 – 107.09683

**Figure 5F**

**Control**

File name: 2023_09_29_0012.abf

Cropped trace timepoint (ms): 50.64083 – 52.64083

**KI**

File name: 2023_09_29_0020.abf

Cropped trace timepoint (ms): 120.94753 – 122.94753

**Figure 5J**

**Control**

File name: 2023_10_10_0006.abf

Cropped trace timepoint (ms): 74.97977 – 76.97977

**KI**

File name: 2023_10_10_0019.abf

Cropped trace timepoint (ms): 12.15806 – 14.15806

**Figure 5N**

**Control**

File name: 2023_07_24_0003.abf

Cropped trace timepoint (ms): 180.88049 – 182.88049

**KI**

File name: 2023_07_24_0010.abf

Cropped trace timepoint (ms): 155.77342 – 157.77342

**Figure 5P**

**Control**

File name: 2023_12_10_0038.abf

Cropped trace timepoint (ms): 417.5 – 517.5

**KI**

File name: 2023_12_10_0002.abf

Cropped trace timepoint (ms): 417.5 – 517.5

**Figure 5S**

**Control**

File name: 2023_12_09_0044.abf

Cropped trace timepoint (ms): 520 – 720

**KI**

File name: 2023_12_09_0040.abf

Cropped trace timepoint (ms): 520 – 720

**Figure 5U**

**Control**

File name: 2023_11_07_0021.abf

Cropped trace timepoint (ms): 417.5 – 517.5

**KI**

File name: 2023_11_07_0009.abf

Cropped trace timepoint (ms): 417.5 – 517.5

**Figure 5X**

**Control**

File name: 2023_11_07_0041.abf

Cropped trace timepoint (ms): 520 – 720

**KI**

File name: 2023_11_07_0005.abf

Cropped trace timepoint (ms): 520 – 720
