# Supplementary material for: Bazedoxifene reverses sexually dimorphic autistic-like abnormalities in biallelic MDGA1-mutant mice
Source: EMBO Mol Med. 2026 Mar 20;18(4):1358–98. doi: 10.1038/s44321-026-00402-y (PMC13084050; doi:10.1038/s44321-026-00402-y)
Supplement: Supplementary file 21 — Expanded View Figures [file 44321_2026_402_MOESM21_ESM.pdf]

Expanded View Figures

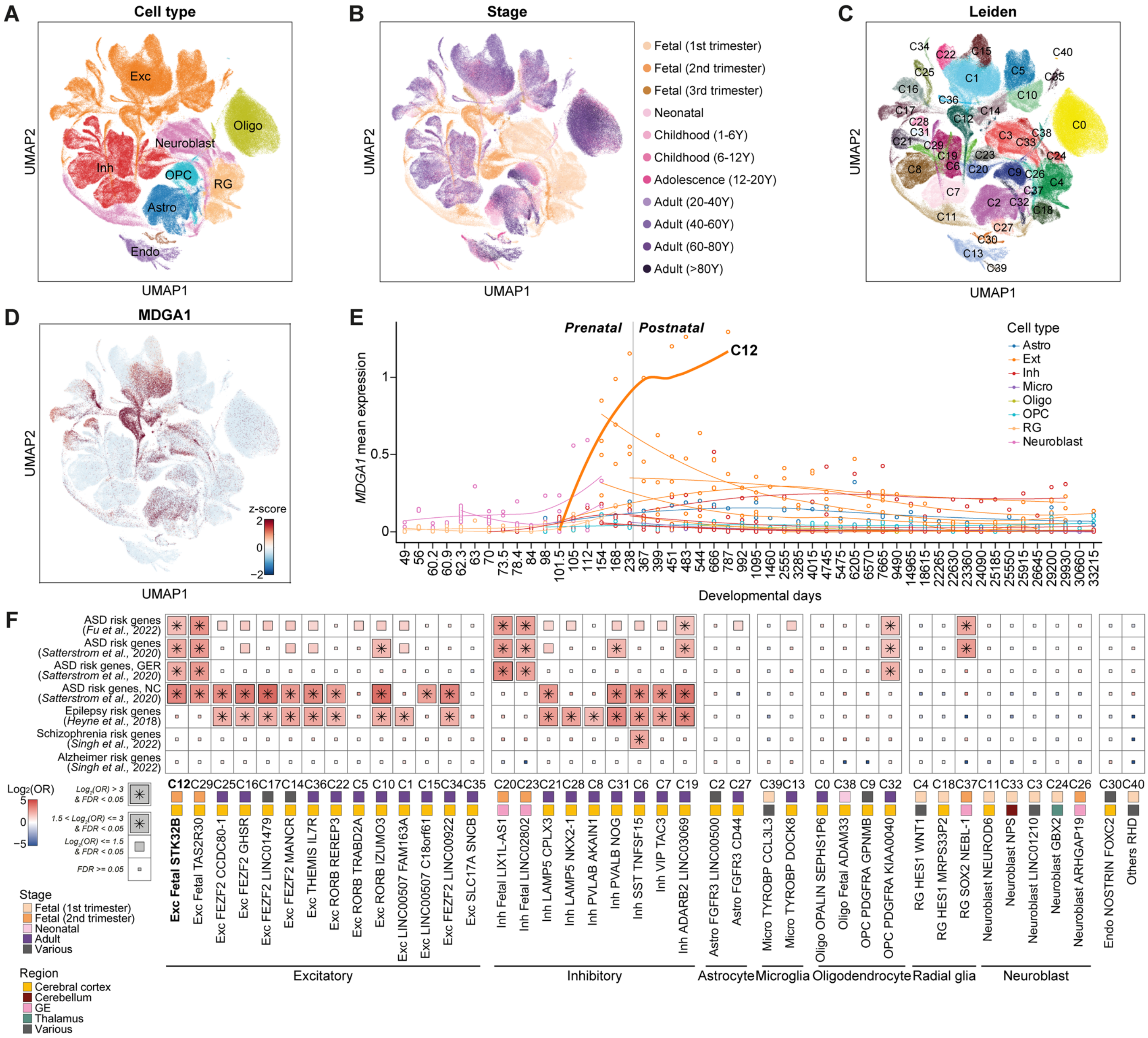

Figure EV1. MDGA1 expression in the human brain single-cell atlas.

(**A–D**) Uniform Manifold Approximation and Projection (UMAP) of the human brain single-cell atlas, colored according to primary cell types (**A**), developmental stages (**B**), Leiden clusters (**C**), and z-score normalized expression of *MDGA1* (**D**). (**E**) Temporal expression of MDGA1 across developmental stages. Data represent sample-wise mean log-normalized expression computed using a pseudobulk method. Analysis was restricted to clusters with ≥ 4600 cells (C0–C22). (**F**) Gene set enrichment analysis between neurological disorder genes and differentially expressed genes in each cluster. Statistical significance was determined using a Fisher's exact test. Significant enrichment was defined as a False Discovery Rate (FDR) < 0.05 and a $\log_2$ (odds ratio) > 1.5.

© The Author(s)

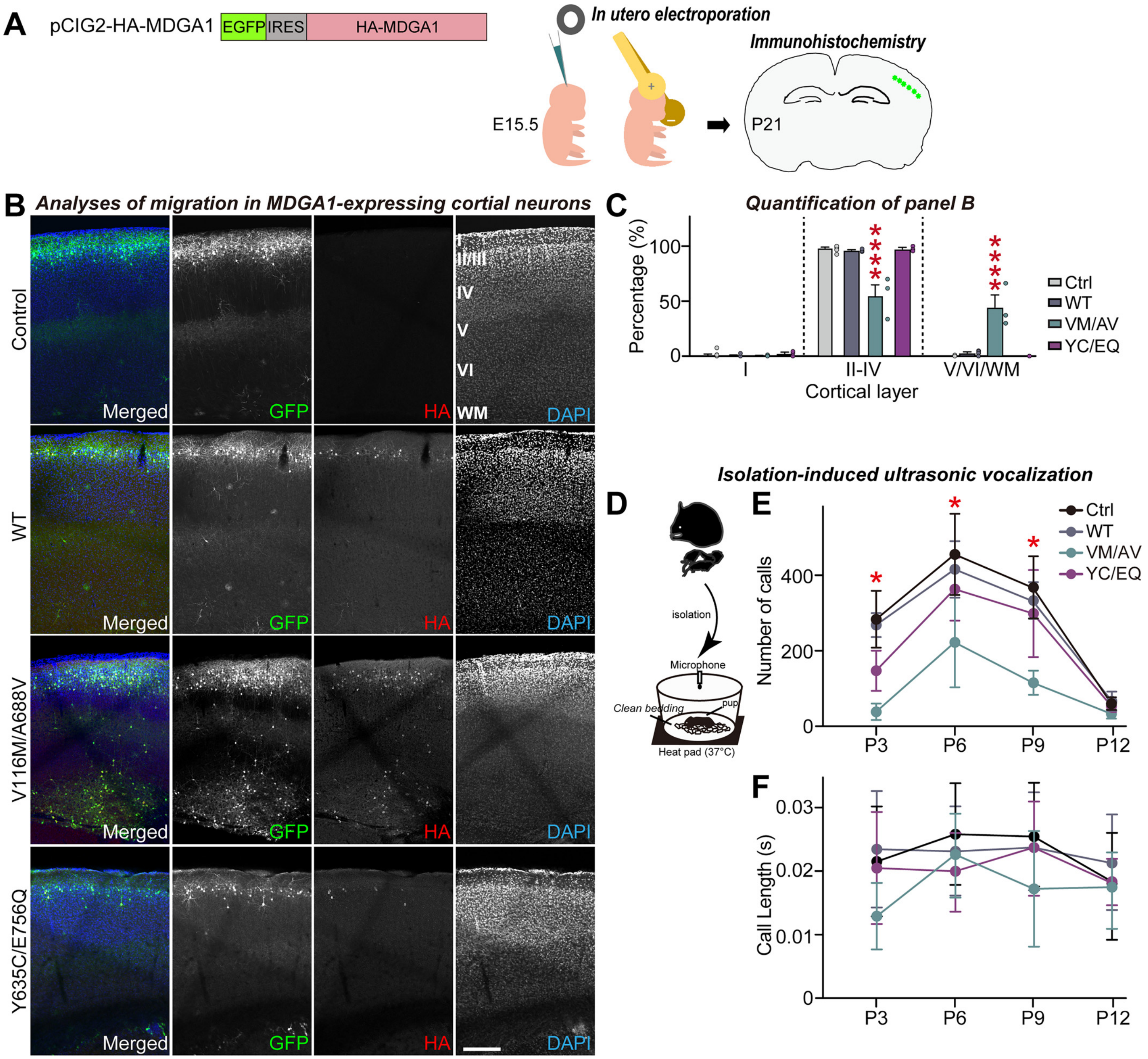

Figure EV2. Analysis of cortical neuronal migration and ultrasonic vocalization in mice expressing ASD-associated MDGA1 variants.

(**A**) Schematic of the in utero electroporation. The indicated plasmids (control or HA-tagged MDGA1 variants [HA-MDGA1-IRES-EGFP]) were electroporated in utero at E15.5 and neuronal migration was analyzed by immunohistochemistry using confocal microscopy at P21. (**B**, **C**) Analysis of the migration trajectory of cortical neurons expressing the indicated MDGA1 variants. Expression of the V116M/A688V variant impaired neuronal migration compared to that seen in control neurons or those expressing MDGA1 WT. Data are presented as means ± SEMs (‘*n*’ denotes number of images/mice; control, $n = 18/9$; WT, $n = 10/5$; V116M/A688V, $n = 6/3$; and Y635C/E756Q, $n = 5/3$; $^{****}P < 0.0001$ (II-IV), $^{****}P < 0.0001$ (V/VI/WM); Mann–Whitney *U* test). Scale bar, 500 µm. (**D**) Schematic of the experimental setup for pup isolation and USV recording. USVs were measured at P3, P6, P9, and P12 from WT pups electroporated in utero with control or MDGA1 variant plasmids. (**E**) Number of USV calls recorded from pups expressing the control or the indicated MDGA1 variant at P3, P6, P9 and P12. Data are presented as means ± SEMs ($n = 7$–9 pups/group; asterisks (*) denote significant differences between control and the VM/AV group; $^{*}P = 0.0134$ (P3), $^{*}P = 0.0338$ (P6), $^{*}P = 0.0385$ (P9); nonparametric Kruskal–Wallis test followed by Dunn’s *post hoc* test). (**F**) Duration of USV calls recorded at the same time points. No significant differences were observed among groups. Data are presented as means ± SEMs ($n = 7$–9 pups/group).

 © The Author(s)

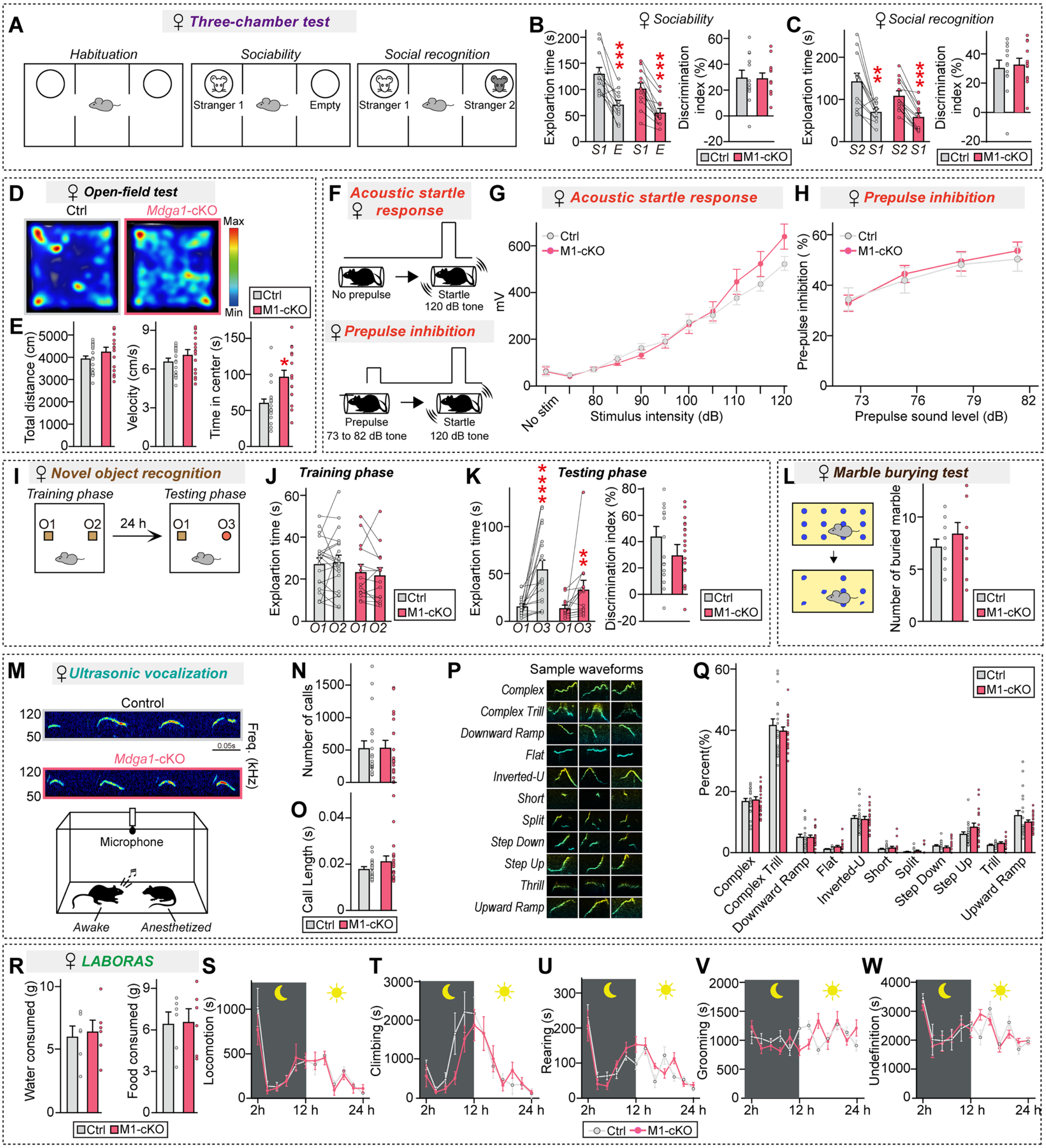
A
Three-chamber test
Habituation
Sociability
Social recognition
Stranger 1
Empty
Stranger 2
B
Sociability
C
Social recognition
D
Open-field test
Ctrl
Mdga1-cKO
E
F
Acoustic startle response
No prepulse
Startle
120 dB tone
Prepulse inhibition
Prepulse
73 to 82 dB tone
G
Acoustic startle response
Stimulus intensity (dB)
H
Prepulse inhibition
Prepulse sound level (dB)
I
Novel object recognition
Training phase
Testing phase
24 h
J
Training phase
K
Testing phase
L
Marble burying test
M
Ultrasonic vocalization
Control
Microphone
Awake
Anesthetized
N
O
P
Sample waveforms
Q
R
LABORAS
S
T
U
V
W
Ctrl
M1-cKO

Figure EV3. Analysis of behaviors of adult female *Mdga1*-cKO mice.

(**A–C**) Three-chamber test results showing a schematic of the test (**A**) and the exploration time and discrimination index during the sociability (**B**; stranger 1 vs. empty) and social recognition (**C**; stranger 1 vs. stranger 2) phases for female control and *Mdga1*-cKO mice. *Mdga1*-cKO females showed normal sociability and intact social recognition, as indicated by comparable exploration times and discrimination indices relative to controls. Data are presented as means ± SEMs ($n = 12$ mice/group; (**B**) ***$P = 0.0010$ (Ctrl), ***$P = 0.0005$ (M1-cKO); (**C**) ***$P = 0.0015$ (Ctrl), ***$P = 0.0005$ (M1-cKO); Mann–Whitney *U* test). (**D**, **E**) Open-field test results showing total distance traveled, velocity, and time spent in the center for female control and *Mdga1*-cKO mice. *Mdga1*-cKO mice exhibited increased time spent in the center zone, while total distance traveled and velocity were comparable to those of controls. Data are presented as means ± SEMs ($n = 15$–20 mice/group; (**E**) *$P = 0.0105$; Mann–Whitney *U* test). (**F**) Schematic of the prepulse inhibition (PPI) test. Mice were exposed to a prepulse sound (73–82 dB) followed by a startle pulse (120 dB), and the inhibition of the startle response was measured. (**G**) Acoustic startle response in female control and *Mdga1*-cKO mice. Data are presented as means ± SEMs ($n = 17$–18 mice/group). (**H**) PPI of the acoustic startle response in female control and *Mdga1*-cKO mice. Data are presented as means ± SEMs ($n = 14$–17 mice/group). (**I–K**) Novel object recognition test results showing a schematic of the test (**I**) and the exploration time and discrimination index during the training (**J**) and testing phases (**K**) for female control and *Mdga1*-cKO mice. Quantification of exploration times during training (**J**) and testing (**K**) revealed no significant differences in the discrimination index between groups, indicating that recognition memory was intact in *Mdga1*-cKO mice. Data are presented as means ± SEMs ($n = 13$–18 mice/group; (**K**) ****$P < 0.0001$ (Ctrl), **$P = 0.0034$ (M1-cKO); Wilcoxon matched-pairs signed rank test). (**L**) Marble burying test results showing the number of marbles buried by female control and *Mdga1*-cKO mice. The number of buried marbles was not significantly different between *Mdga1*-cKO and control mice, suggesting that there was no change in repetitive behavior. Data are presented as means ± SEMs ($n = 11$–14 mice/group). (**M–Q**) USV results showing a representative sonogram (**M**) and the number of calls (**N**) and call length (**O**) for female control and *Mdga1*-cKO mice. Representative waveforms (**P**) serve as standardized reference samples for each USV category and are utilized across figures (e.g., Fig. EV4P) as a consistent visual index for classification criteria. Distribution of different call types (**Q**) is shown. No significant differences in call-type proportions were observed, suggesting that vocal communication was intact. Data are presented as means ± SEMs ($n = 18$–20 mice/group; Mann–Whitney *U* test). (**R–W**) Quantification of spontaneous behaviors using the LABORAS system over a 24-h period. No significant differences were observed in food and water intake (**R**), locomotion (**S**), climbing (**T**), rearing (**U**), grooming (**V**), or undefined behaviors (**W**) between *Mdga1*-cKO and control mice. Data are presented as means ± SEMs ($n = 6$ mice/group).

 © The Author(s)

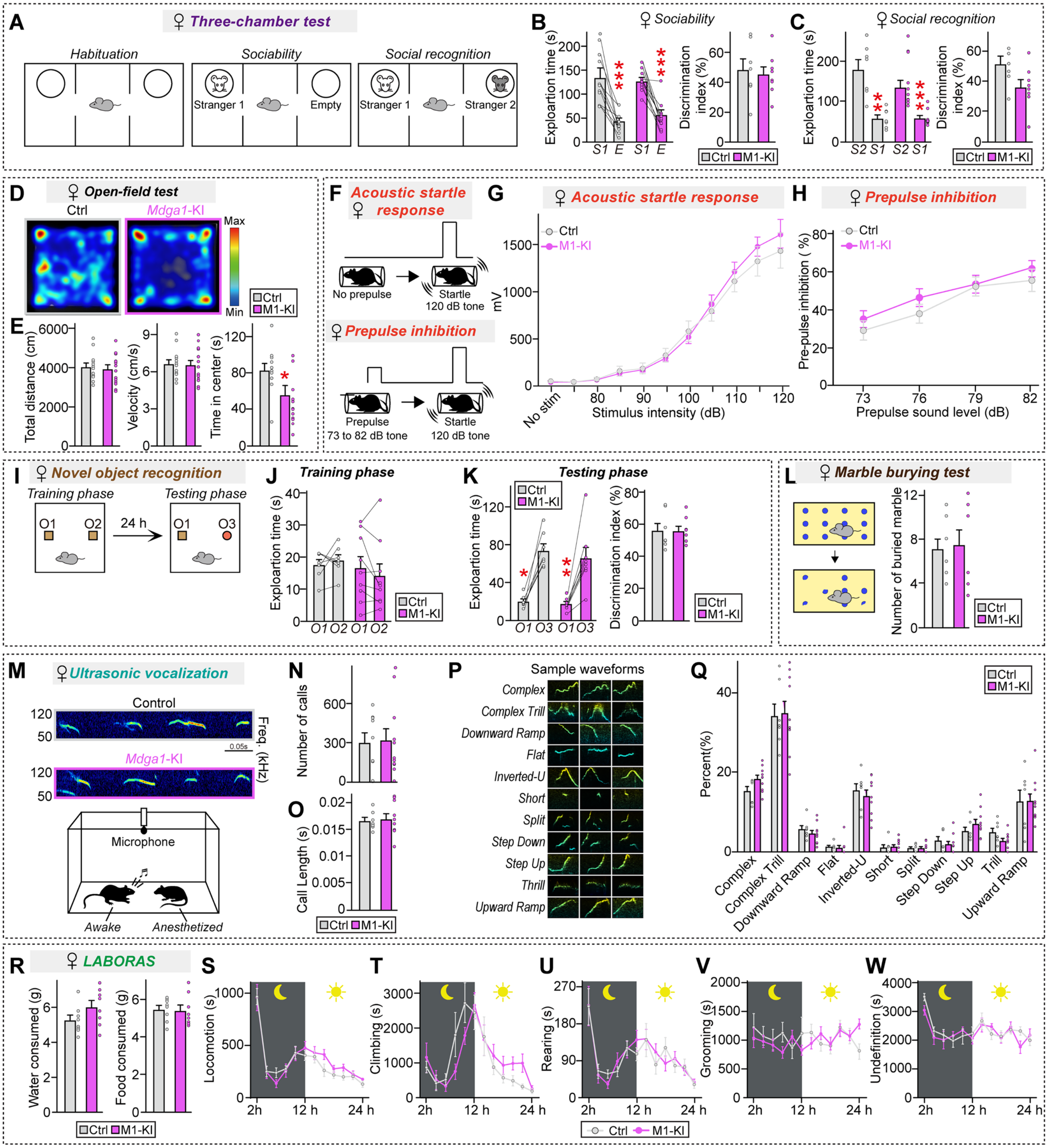
A
♀ Three-chamber test
Habituation
Sociability
Social recognition
Stranger 1
Empty
Stranger 1
Stranger 2
B
♀ Sociability
Exploartion time (s)
Discrimination index (%)
S1 E S1 E
Ctrl M1-KI
C
♀ Social recognition
S2 S1 S2 S1
D
♀ Open-field test
Ctrl
Mdga1-KI
Max
Min
E
Total distance (cm)
Velocity (cm/s)
Time in center (s)
F
♀ Acoustic startle response
No prepulse
Startle
120 dB tone
♀ Prepulse inhibition
Prepulse
73 to 82 dB tone
G
♀ Acoustic startle response
mV
No stim
Stimulus intensity (dB)
H
♀ Prepulse inhibition
Pre-pulse inhibition (%)
Prepulse sound level (dB)
I
♀ Novel object recognition
Training phase
Testing phase
24 h
O1 O2
O1 O3
J
Training phase
Exploartion time (s)
K
Testing phase
Discrimination index (%)
L
♀ Marble burying test
Number of buried marble
M
♀ Ultrasonic vocalization
Control
Mdga1-KI
Freq. (kHz)
0.05s
Microphone
Awake
Anesthetized
N
Number of calls
O
Call Length (s)
P
Sample waveforms
Complex
Complex Trill
Downward Ramp
Flat
Inverted-U
Short
Split
Step Down
Step Up
Thrill
Upward Ramp
Q
Percent(%)
Trill
R
♀ LABORAS
Water consumed (g)
Food consumed (g)
S
Locomotion (s)
T
Climbing (s)
U
Rearing (s)
V
Grooming (s)
W
Undefinition (s)
2h
12 h
24 h

© The Author(s)

Figure EV4. Analysis of behaviors of adult female *Mdga1*$^{Y636C/E751Q}$ KI mice.

(**A–C**) Three-chamber test results showing a schematic of the test (**A**) and the exploration time and discrimination index during the sociability (**B**; stranger 1 vs. empty) and social recognition (**C**; stranger 1 vs. stranger 2) phases for female control and *Mdga1*$^{Y636C/E751Q}$ KI mice. *Mdga1*$^{Y636C/E751Q}$ KI females showed normal sociability and intact social recognition, as indicated by comparable exploration times and discrimination indices relative to those of controls. Data are means ± SEMs ($n = 11$–13 mice/group; (**B**) ***$P = 0.0010$ (Ctrl), ***$P = 0.0002$ (M1-KI); (**C**) **$P = 0.0068$ (Ctrl), ***$P = 0.0005$ (M1-KI); Wilcoxon matched-pairs signed rank test). (**D**, **E**) Open-field test results showing total distance traveled, velocity, and time spent in the center for female control and *Mdga1*$^{Y636C/E751Q}$ KI mice. *Mdga1*$^{Y636C/E751Q}$ KI mice exhibited decreased time spent in the center zone, while the total distance traveled and velocity were comparable to those of controls. Data are presented as means ± SEMs ($n = 11$–13 mice/group; (**E**) *$P = 0.0105$, Mann–Whitney *U* test). (**F–H**) Schematic (**F**) of the prepulse inhibition (PPI) test. Mice were exposed to a prepulse sound (73–82 dB) followed by a startle pulse (120 dB), and the inhibition of the startle response was measured. Acoustic startle response (**G**) in female control and female *Mdga1*$^{Y636C/E751Q}$ KI mice. Data are presented as means ± SEMs ($n = 11$–14 mice/group). PPI of the acoustic startle response (**H**) in female control and *Mdga1*$^{Y636C/E751Q}$ KI mice. Data are presented as means ± SEMs ($n = 11$–14 mice/group). (**I–K**) Novel object recognition test results showing a schematic of the test (**I**) and the exploration time and discrimination index during the training (**J**) and testing phases (**K**) for female control and *Mdga1*$^{Y636C/E751Q}$ KI mice. Quantification of exploration times during training (**J**) and testing (**K**) revealed no significant difference in the discrimination index between groups, indicating that recognition memory was intact in *Mdga1*$^{Y636C/E751Q}$ KI mice. Data are presented as means ± SEMs ($n = 7$–9 mice/group; (**K**) *$P = 0.0156$ (Ctrl), **$P = 0.0078$ (M1-KI); Wilcoxon matched-pairs signed rank test). (**L**) Marble burying test results showing the number of marbles buried by female control and female *Mdga1*$^{Y636C/E751Q}$ KI mice. The number of buried marbles was not significantly different between *Mdga1*$^{Y636C/E751Q}$ KI and control mice, suggesting that there was no change in repetitive behavior. Data are presented as means ± SEMs ($n = 8$ mice/group). (**M–Q**) USVs recorded from adult female mice under social isolation. Representative sonograms (**M**), quantification of USV call number (**N**) and duration (**O**), and representative waveforms (**P**) are shown. Note that the waveforms in (**P**) serve as standardized reference samples for each USV category and are utilized across figures (e.g., Fig. EV3P) as a consistent visual index for classification criteria. Distribution of call types (**Q**) was assessed; no significant differences in call-type proportions were observed, suggesting that vocal communication was intact. Data are presented as means ± SEMs ($n = 16$–18 mice/group). (**R–W**) Quantification of spontaneous behaviors using the LABORAS system over a 24-h period. No significant differences were observed in food and water intake (**R**), locomotion (**S**), climbing (**T**), rearing (**U**), grooming (**V**), or undefined behaviors (**W**) between *Mdga1*$^{Y636C/E751Q}$ KI and control mice. Data are presented as means ± SEMs ($n = 8$ mice/group).

 © The Author(s)

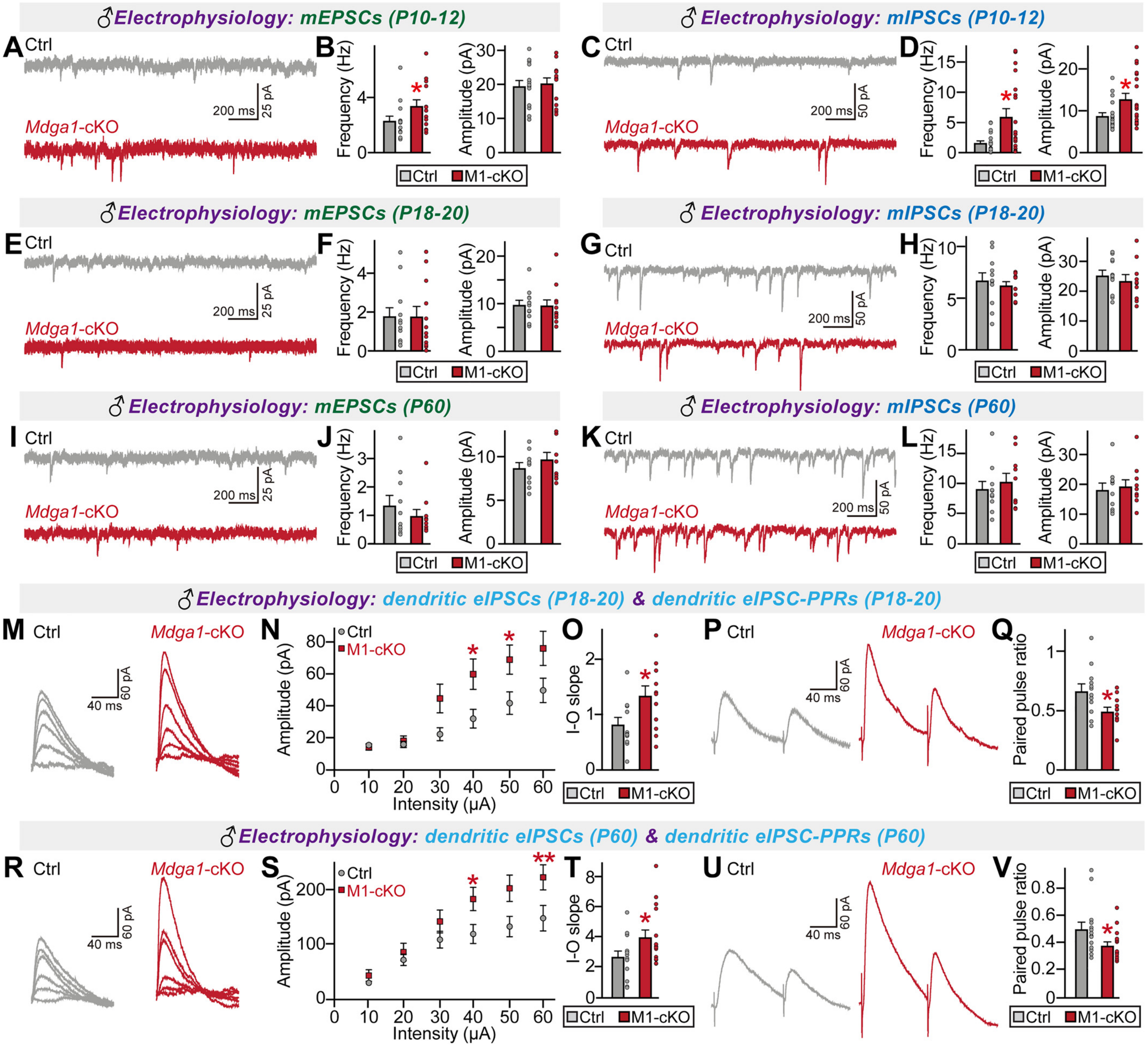
♂Electrophysiology: mEPSCs (P10-12)
♂Electrophysiology: mIPSCs (P10-12)
♂Electrophysiology: mEPSCs (P18-20)
♂Electrophysiology: mIPSCs (P18-20)
♂Electrophysiology: mEPSCs (P60)
♂Electrophysiology: mIPSCs (P60)
♂Electrophysiology: dendritic eIPSCs (P18-20) & dendritic eIPSC-PPRs (P18-20)
♂Electrophysiology: dendritic eIPSCs (P60) & dendritic eIPSC-PPRs (P60)
Ctrl
Mdga1-cKO
M1-cKO
Frequency (Hz)
Amplitude (pA)
Intensity (μA)
I-O slope
Paired pulse ratio
200 ms
40 ms
25 pA
50 pA
60 pA

Figure EV5. Analysis of electrophysiological properties in the hippocampal CA1 pyramidal neurons of various aged male *Mdga1*-cKO mice.

(**A**, **B**) Representative traces (**A**) and quantification (**B**) of mEPSC frequency and amplitude recorded from CA1 pyramidal neurons of P10–12 male mice. *Mdga1*-cKO neurons showed significantly increased mEPSC frequency, while amplitude remained unchanged. Data are means ± SEMs (control, $n = 15/6$; *Mdga1*-cKO, $n = 14/6$; $^*P = 0.0459$, Mann–Whitney *U* test). (**C**, **D**) Representative traces (**C**) and quantification (**D**) of mIPSCs from P10–12 CA1 pyramidal neurons. *Mdga1*-cKO mice exhibited increased mIPSC frequency and amplitude. Data are means ± SEMs (control, $n = 19/6$; *Mdga1*-cKO, $n = 19/6$; (frequency) $^*P = 0.0233$, (amplitude) $^*P = 0.0215$; Mann–Whitney *U* test). (**E**, **F**) Representative traces (**E**) and quantification (**F**) of mEPSCs recorded from CA1 pyramidal neurons of P18–20 male mice. No significant differences were observed between genotypes. Data are means ± SEMs (control, n = 12/4; *Mdga1*-cKO, $n = 12/4$). (**G**, **H**) Representative traces (**G**) and quantification (**H**) of mIPSCs recorded from CA1 pyramidal neurons of P18–20 male mice. No significant differences were observed between genotypes. Data are means ± SEMs (control, $n = 11/4$; *Mdga1-cKO*, n = 10/4). (**I**, **J**) Representative traces (**I**) and quantification (**J**) of mEPSCs recorded from CA1 pyramidal neurons of P60 male mice. No significant differences were observed between genotypes. Data are means ± SEMs ($n = 10/4$ per group). (**K**, **L**) Representative traces (**K**) and quantification (**L**) of mIPSCs recorded from CA1 pyramidal neurons of P60 male mice. No significant differences were detected between genotypes. Data are means ± SEMs ($n = 10/4$ per group). (**M–O**) Representative traces (**M**) and averages of dendritic eIPSCs (**N**, **O**) from CA1 pyramidal neurons of male control and *Mdga1*-cKO mice at P18–20. *Mdga1*-cKO mice exhibited a significant increase in eIPSC amplitude. Data are presented as means ± SEMs (control, $n = 12/4$; *Mdga1*-cKO, $n = 12/4$; (**N**) (40 μA) $^*P = 0.0402$, (50 μA) $^*p = 0.0449$; (**O**) $^*p = 0.0242$; Mann–Whitney *U* test). (**P**, **Q**) Representative traces (**P**) and averages of paired-pulse ratios (PPRs) of dendritic IPSCs (**Q**) from CA1 pyramidal neurons of male control and *Mdga1*-cKO mice at P18–20. *Mdga1*-cKO mice showed a significant reduction in PPRs. Data are presented as means ± SEMs (control, $n = 13/4$; *Mdga1*-cKO, $n = 13/4$; $^*P = 0.0387$; Mann–Whitney *U* test). (**R–T**) Representative traces (**R**) and averages of dendritic eIPSCs (**S**, **T**) from CA1 pyramidal neurons of male control and *Mdga1*-cKO mice at P60. eIPSC amplitude was significantly increased in *Mdga1*-cKO mice. Data are presented as means ± SEMs (control, $n = 13/5$; *Mdga1*-cKO, $n = 16/5$; (**S**) (40 μA) $^*P = 0.0435$, (60 μA) $^{**}P = 0.0066$; (**T**) $^*P = 0.0499$; Mann–Whitney *U* test). (**U**, **V**) Representative traces (**U**) and averages of dendritic eIPSC-PPRs (**V**) from CA1 pyramidal neurons of male control and *Mdga1*-cKO mice at P60. *Mdga1*-cKO mice showed significantly decreased dendritic eIPSC-PPRs. Data are presented as means ± SEMs (control, $n = 14/5$; *Mdga1*-cKO, $n = 16/5$; $^*P = 0.0267$; Mann–Whitney *U* test).

 © The Author(s)

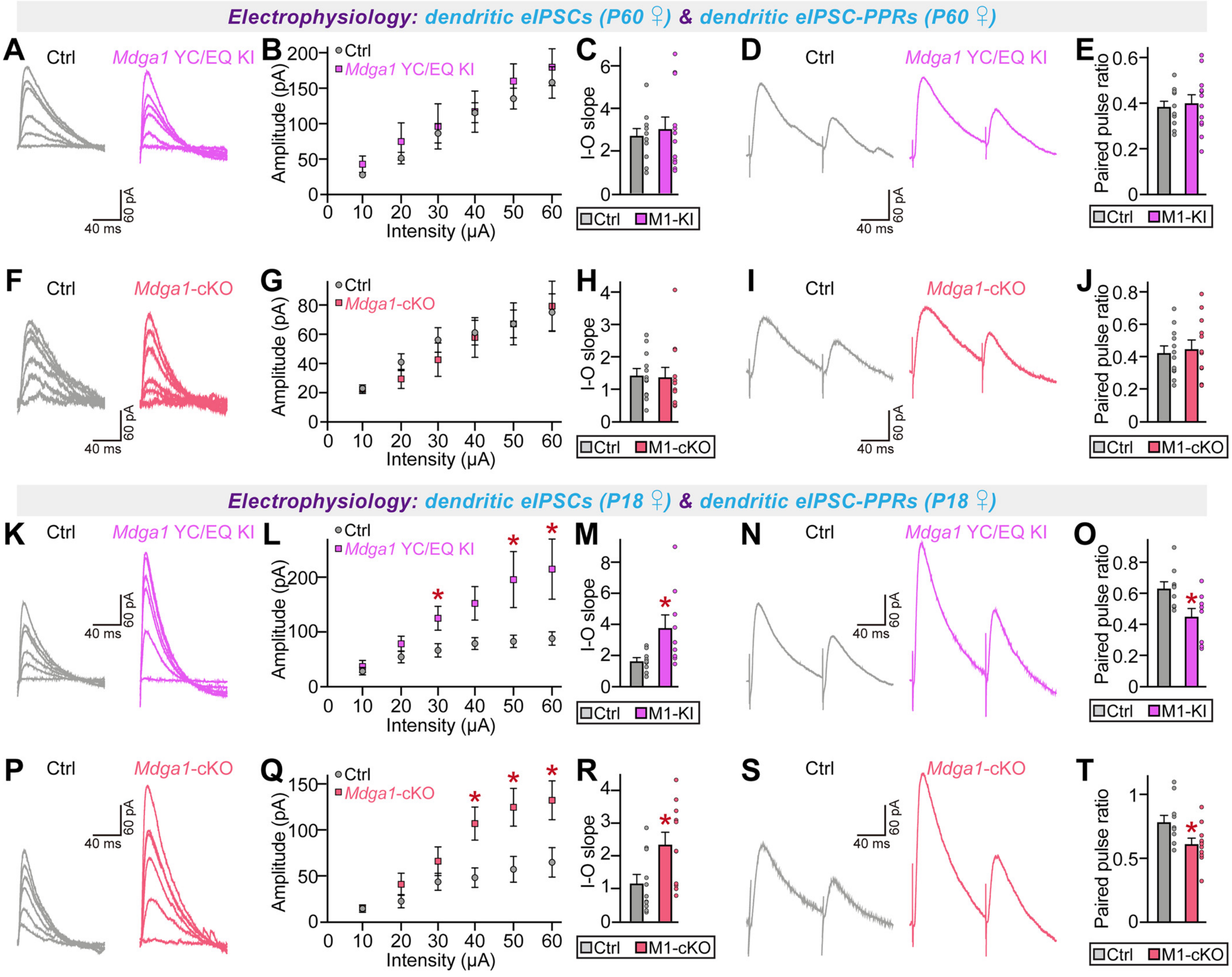

Figure EV6. Analysis of synchronous GABAergic evoked synaptic transmission in the hippocampal CA1 pyramidal neurons of adult female *Mdga1*-cKO and *Mdga1*$^{Y636C/E751Q}$ KI mice.

(**A–E**) Representative traces (**A**, **D**), input-output (I-O) curves (**B**, **C**), and paired-pulse ratios (PPRs) (**E**) of dendritic eIPSCs recorded from CA1 pyramidal neurons in adult female control and *Mdga1*$^{Y636C/E751Q}$ KI mice. No significant differences were observed. Data are presented as means ± SEMs ($n = 12$ cells/group). (**F–J**) Representative traces (**F**, **I**), I-O curves (**G**, **H**), and PPRs (**J**) of dendritic eIPSCs recorded from CA1 pyramidal neurons in adult female control and *Mdga1*-cKO mice. No significant differences were observed. Data are presented as means ± SEMs ($n = 12$ cells/group). (**K–O**) Representative traces (**K**, **N**), I-O curves (**L**, **M**), and PPRs (**O**) of dendritic eIPSCs recorded from CA1 pyramidal neurons in P18 female control and *Mdga1*$^{Y636C/E751Q}$ KI mice. *Mdga1*$^{Y636C/E751Q}$ KI mice showed significantly increased eIPSC amplitude (**M**) and decreased PPRs (**O**). Data are presented as means ± SEMs ($n = 9$ cells/group; (**L**) (30 µA) $^*P = 0.0333$, (50 µA) $^*P = 0.0333$, (60 µA) $^*P = 0.0151$; (**M**) $^*P = 0.0142$; (**O**) $^*P = 0.0188$; Mann–Whitney *U* test). (**P–T**) Representative traces (**P**, **S**), I-O curves (**Q**, **R**), and PPRs (**T**) of dendritic eIPSCs recorded from CA1 pyramidal neurons in P18 female control and *Mdga1*-cKO mice. *Mdga1*-cKO mice exhibited significantly increased eIPSC amplitude (**Q**) and decreased PPRs (**T**). Data are presented as means ± SEMs ($n = 11$ cells/group; (**Q**) (40 µA) $^*P = 0.0199$, (50 µA) $^*P = 0.0158$, (60 µA) $^*P = 0.0135$; (**R**) $^*P = 0.0192$; (**T**) $^*P = 0.0225$; Mann–Whitney *U* test).

© The Author(s)
